# DNA barcode library for European Gelechiidae (Lepidoptera) suggests greatly underestimated species diversity

**DOI:** 10.3897/zookeys.921.49199

**Published:** 2020-03-24

**Authors:** Peter Huemer, Ole Karsholt, Leif Aarvik, Kai Berggren, Oleksiy Bidzilya, Jari Junnilainen, Jean-François Landry, Marko Mutanen, Kari Nupponen, Andreas Segerer, Jan Šumpich, Christian Wieser, Benjamin Wiesmair, Paul D.N. Hebert

**Affiliations:** 1 Naturwissenschaftliche Sammlungen, Tiroler Landesmuseen Betriebsges.m.b.H., Innsbruck, Austria Tiroler Landesmuseen Betriebsges.m.b.H. Innsbruck Austria; 2 Zoological Museum, Natural History Museum of Denmark, Copenhagen, Denmark Natural History Museum of Denmark Copenhagen Denmark; 3 Natural History Museum, University of Oslo, Oslo, Norway University of Oslo Oslo Norway; 4 Kristiansand, Norway Unaffiliated Kristiansand Norway; 5 Institute for Evolutionary Ecology of the National Academy of Sciences of Ukraine, Kiev, Ukraine Institute for Evolutionary Ecology, National Academy of Sciences of Ukraine Kiev Ukraine; 6 Finnish Museum of Natural History, Zoology Unit, Helsinki, Finland Finnish Museum of Natural History Helsinki Finland; 7 Canadian National Collection of Insects, Arachnids, and Nematodes, Ottawa Research and Development Centre, Agriculture and Agri-Food Canada, Ottawa, Canada Agriculture and Agri-Food Canada Ottawa Canada; 8 Department of Ecology and Genetics, University of Oulu, Finland University of Oulu Oulu Finland; 9 Espoo, Finland Unaffiliated Espoo Finland; 10 SNSB-Zoological State Collection, Munich, Germany Zoological State Collection Munich Germany; 11 National Museum, Natural History Museum, Department of Entomology, Praha, Czech Republic Natural History Museum Prague Czech Republic; 12 Landesmuseum Kärnten, Klagenfurt, Austria Landesmuseum Kärnten Klagenfurt am Wörthersee Austria; 13 Centre for Biodiversity Genomics, University of Guelph, Guelph, Canada University of Guelph Guelph Canada

**Keywords:** Europe, cryptic diversity, DNA barcoding, revision, species delimitation

## Abstract

For the first time, a nearly complete barcode library for European Gelechiidae is provided. DNA barcode sequences (COI gene – cytochrome *c* oxidase 1) from 751 out of 865 nominal species, belonging to 105 genera, were successfully recovered. A total of 741 species represented by specimens with sequences ≥ 500bp and an additional ten species represented by specimens with shorter sequences were used to produce 53 NJ trees. Intraspecific barcode divergence averaged only 0.54% whereas distance to the Nearest-Neighbour species averaged 5.58%. Of these, 710 species possessed unique DNA barcodes, but 31 species could not be reliably discriminated because of barcode sharing or partial barcode overlap. Species discrimination based on the Barcode Index System (BIN) was successful for 668 out of 723 species which clustered from minimum one to maximum 22 unique BINs. Fifty-five species shared a BIN with up to four species and identification from DNA barcode data is uncertain. Finally, 65 clusters with a unique BIN remained unidentified to species level. These putative taxa, as well as 114 nominal species with more than one BIN, suggest the presence of considerable cryptic diversity, cases which should be examined in future revisionary studies.

## Introduction

**Figure 1.**
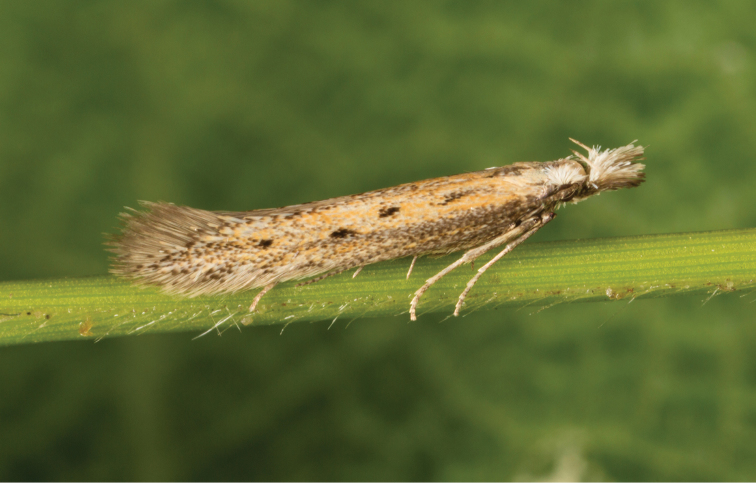
*Megacraspedusteriolensis* is a characteristic example of gelechiid moths only recognised and described during the last few years.

The megadiverse family, Gelechiidae, includes approximately 4,700 known species and perhaps a similar number of undescribed taxa (Karsholt et al. 2013). With a remarkable 865 species reported from Europe and adjacent islands (Huemer and Karsholt 2020), the Gelechiidae are the fourth most diverse family of Lepidoptera after the Noctuidae, Geometridae, and Tortricidae in Europe. Due to their general dull-coloured and inconspicuously patterned wings (Fig. 1), and frequently small size, the Gelechiidae have received little attention from lepidopterists, leading to considerable gaps in knowledge of their taxonomy, systematics, biology, and distribution. In particular, the lack of generic revisions in several diverse groups has created the widespread impression of a “difficult” family which has acted to further limit interest in this group.

Over the last two decades, the Gelechiidae have received increasing attention as a result of two monographs that treated approximately half the known European species (Huemer and Karsholt 1999, 2010) and another on the Central European fauna (Elsner et al. 1999). Unfortunately, these publications, as well as several subsequent revisions (i.e., Bidzilya 2005a, 2005b, Bidzilya and Karsholt 2015, Karsholt and Rutten 2005, Karsholt and Šumpich 2015, Li and Sattler 2012), did not take advantage of new molecular methods, in particular DNA barcoding. On the contrary phylogenetic analysis of higher taxa in Gelechiidae benefitted greatly from molecular analysis (Kaila et al. 2011, Karsholt et al. 2013). However, recent studies on several genera of European Gelechiidae (Huemer et al. 2013, 2014, Huemer and Mutanen 2012, Huemer and Karsholt 2014, Landry et al. 2017) revealed the power of this approach to aid species delimitation in taxonomically difficult groups, even those with a high level of unrecorded species and cryptic diversity. Similar patterns have been analyzed in several other Lepidoptera in different parts of the world, e.g., in another gelechioid group (Mutanen et al. 2011), in Iberian butterflies (Dincă et al. 2015), in North American Noctuoidea (Zahiri et al. 2017), or in the Lepidoptera fauna of Costa Rica (Janzen and Hallwachs 2016). These results motivated the present effort to compile a comprehensive DNA barcode library for the European Gelechiidae fauna, with the aim of simplifying future revisionary studies while also improving their quality.

## Materials and methods

### Checklist of European Gelechiidae

The lack of an updated checklist for European Gelechiidae (see Karsholt 2004-2019) was such a major impediment to the present study that it necessitated the assembly of a new systematic list (Huemer and Karsholt 2020). This list, which includes 865 species of Gelechiidae in 109 genera, provided the basis for selecting the specimens that were analysed in this study.

### Sample material

One major challenge was the difficulty in accessing specimens suitable for molecular analysis, reflecting the rarity of many species. In addition, DNA quality of the specimens was another very important limitation as sequence recovery from older specimens of rare taxa was either partial or failed completely even with protocols that employed high-throughput sequencers to analyze short amplicons. In some cases, efforts were made to recollect taxa that lacked a sequence record.

**Figure 2.**
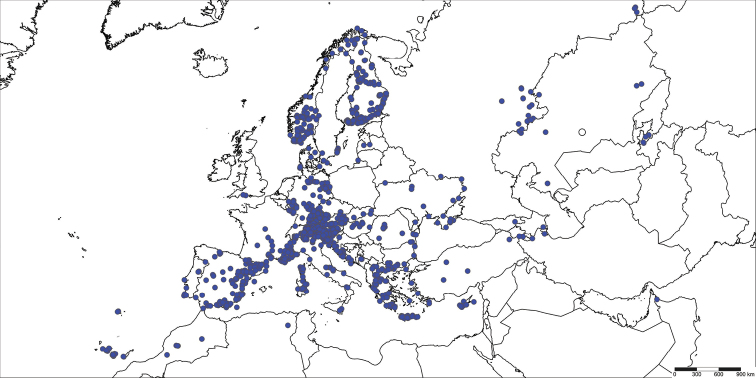
Distribution map of examined material of Gelechiidae (extra-European material partially mapped). SimpleMappr (http://www.simplemappr.net).

Voucher material was obtained from Europe (Fig. 2) except for eleven taxa whose sequences could not be recovered from specimens from this continent or where it seemed important to analyze specimens to clarify taxonomy (e.g., extra-European type-material) (Suppl. material 2, 3). Approximately two-thirds of specimens originated from four nations - Germany (1319), Austria (1157), Italy (906), and Finland (707). The remaining specimens derived from 33 other countries (Fig. 2).

Many institutions and private collectors contributed to the dataset (see below), supplemented by DNA barcodes from earlier studies.

### Abbreviations of private and institutional collections

**BIOUG** Centre for Biodiversity Genomics, Guelph, Canada

**INDO** Inatura, Dornbirn, Austria

**LMK** Landesmuseum Kärnten, Klagenfurt, Austria

**MFSN** Museo Friulano di Storia Natural, Udine, Italy

**MZH** Finnish Museum of Natural History, Helsinki, Finland

**NHM** Natural History Museum, London, United Kingdom

**NHMO** Natural History Museum, University of Oslo, Oslo, Norway

**NHMW** Naturhistorisches Museum, Vienna, Austria

**NMPC** National Museum Prague, Czech Republic

**NMS** Naturmuseum Südtirol, Bozen, Italy

**RCAH** Research Collection Alfred Haslberger, Teisendorf, Germany

**RCER** Research Collection Emily Requena Miret, Gurb, Spain

**RCGB** Research Collection Giorgio Baldizzone, Asti, Italy

**RCGT** Research Collection Giovanni Timossi, Oderzo, Italy

**RCHW** Research Collection Hartmut Wegner, Adendorf, Germany

**RCIB** Research Collection Ian Barton, Cambs, United Kingdom

**RCIR** Research Collection Ignác Richter, Malá Čausa, Slovakia

**RCJD** Research Collection Jordi Dantart, Barcelona, Spain

**RCJJ** Research Collection Jari Junnilainen, Vantaa, Finland

**RCJK** Research Collection Jari-Pekka Kaitila, Vantaa, Finland

**RCJL** Research Collection Gérard Labonne, Montpellier, France

**RCJN** Research Collection Jacques Nel, La Ciotat, France

**RCJS** Research Collection Jan Skyva, Prague, Czech Republic

**RCJSC** Research Collection Jürg Schmid, Illanz, Switzerland

**RCKB** Research Collection Kai Berggren, Kristiansand, Norway

**RCKN** Research Collection Kari and Timo Nupponen, Espoo, Finland

**RCMC** Research Collection Martin Corley, Faringdon, U.K.

**RCOB** Research Collection Oleksiy Bidzilya, Kiev, Ukraine

**RCOR** Research Collection Oliver Rist, Vienna, Austria

**RCPB** Research Collection Peter Buchner, Schwarzau am Steinfeld, Austria

**RCPL** Research Collection Peter Lichtmannecker, Adlkofen, Germany

**RCRH** Research Collection Robert Heckford, Plympton, Plymouth, U.K.

**RCRHE** Research Collection Richard Heindel, Günzburg, Germany

**RCSP** Research Collection Serge Peslier, Perpignan, France

**RCTG** Research Collection Thomas Guggemoos, Ohlstadt, Germany

**RCTM** Research Collection Toni Mayr, Feldkirch, Austria

**RCTV** Research Collection Thierry Varenne, Nice, France

**RCWS** Research Collection Wolfgang Stark, Trübensee, Austria

**RCZT** Research Collection Zdenko Tokár, Šal’a, Slovakia

**TLMF** Tiroler Landesmuseum Ferdinandeum, Innsbruck, Austria

**USNM** Smithsonian Institution, National Museum of Natural History, Washington DC, U.S.A.

**ZMAK** Zoologisches Forschungsmuseum Alexander Koenig, Bonn, Germany

**ZMKU** Taras Shevchenko National University of Kiev, Kiev, Ukraine

**ZMUC** Zoological Museum, Natural History Museum of Denmark, Copenhagen, Denmark

**ZMUO** Zoological Museum, University of Oulu, Finland

**ZSM** Zoologische Staatssammlung, Munich, Germany

### DNA sequencing

A single leg was removed from each specimen and placed in a 96-well lysis plate that was submitted for analysis at the CCDB (Canadian Center for DNA Barcoding, University of Guelph, Canada) where DNA extraction, PCR amplification, and sequencing were performed following standard high-throughput protocols (deWaard et al. 2008). In total, 5986 specimens of European Gelechiidae, initially pre-identified from external and partially genitalia morphology by several colleagues and cross-checked by PH and OK in dubious cases, were successfully sequenced. Details of specimens, including complete voucher data, images, and GenBank accession numbers are available on BOLD (Ratnasingham 2018, Ratnasingham and Hebert 2007) in the public dataset “Lepidoptera (Gelechiidae) of Europe” under the DOI: dx.doi.org/10.5883/DS-GELECHEU.

### Data analysis

Levels of intra- and interspecific variation in the DNA barcode fragment were calculated under the Kimura 2-parameter (K2P) model of nucleotide substitution using analytical tools in BOLD systems v4.0 (http://www.boldsystems.org). Fifty-three Neighbor-Joining trees (Maximum Composite Likelihood method, default settings), most including representatives of a single genus, were constructed using MEGA X (Kumar et. al 2018) (Suppl. material 2 and 3). Node confidences were estimated using 500 bootstrap replicates. For genera with few species, several morphologically closely related genera were included in a single tree. For calculating these trees only sequences ≥ 500 bp were used, except for ten species where only shorter sequences were available (Suppl. material 1). In those cases where the specimens of a single species were assigned to two or more different BINs, they were discriminated by a letter code. Because of the high number of BINs for *Megacraspedusdolosellus* and *M.lanceolellus*, these taxa were figured in two separate NJ trees with BINs separated as single clusters. Species sharing a BIN, but still with a diagnostic barcode were grouped in separate clusters. A three-letter code (ISO 3166-1 alpha-3, https://en.wikipedia.org/wiki/ISO_3166-1_alpha-3) was used to abbreviate country names.

Identification success was assessed by the Barcode Index Number (BIN) system as implemented on BOLD (Ratnasingham and Hebert 2013). This system employs a two-stage algorithm that groups all sequences > 500 bp that meet defined quality criteria into Operational Taxonomic Units (OTUs) and automatically assigns new sequences, irrespective of their previous taxonomy and origin. Concordance or discordance between BINs and morphological species identification was assessed.

## Results

### Overview

DNA barcode sequences were recovered from 5986 specimens representing 751 of the 865 species of Gelechiidae described from Europe (Suppl. material 1). In addition, the analysis revealed 65 putative species whose members were each assigned to a different unique BIN. Most sequences (5476) were compliant with the barcode standard as described in BOLD (http://www.boldsystems.org). Most subsequent analyses only considered the 741 species with sequences ≥ 500bp, but ten additional species with sequences ≥ 300 bp were included in the NJ trees. Sequences from 723 species qualified for BIN analysis.

**Table 1.** 42 Species with Nearest-Neighbour distances of 0–1%.

**Species**	**Mean intra-spec.**	**Max intra-spec.**	**Nearest species**	**Dist. NN**
*Bryotrophaaffinis*	0.17	0.77	*Bryotrophaumbrosella*	0
*Bryotrophaumbrosella*	1.76	3.63	*Bryotrophaaffinis*	0
*Iwarunabiguttella*	0.78	2.02	*Iwarunaklimeschi*	0
*Iwarunaklimeschi*	0	0	*Iwarunabiguttella*	0
*Teleiodesbrevivalva*	0.46	0.46	*Teleiodesvulgella*	0
*Teleiodesitalica*	0.32	0.62	*Teleiodesvulgella*	0
*Teleiodesvulgella*	0.17	0.5	*Teleiodesitalica*	0
*Xenolechiaaethiops*	0.08	0.16	*Xenolechialindae*	0
*Xenolechialindae*	0	0	*Xenolechiaaethiops*	0
*Xenolechiapseudovulgella*	N/A	0	*Xenolechiaaethiops*	0
*Scrobipalpaalterna*	N/A	0	*Scrobipalpalutea*	0.35
*Scrobipalpalutea*	N/A	0	*Scrobipalpaalterna*	0.35
*Acompsiaantirrhinella*	1.39	1.39	*Acompsiatripunctella*	0.46
*Acompsiatripunctella*	2.59	6.4	*Acompsiaantirrhinella*	0.46
*Dirhinosiacervinella*	0.14	0.32	*Dirhinosiainterposita*	0.46
*Dirhinosiainterposita*	0	0	*Dirhinosiacervinella*	0.46
*Monochroaarundinetella*	0.05	0.15	*Monochroasuffusella*	0.47
*Monochroasuffusella*	0.52	1.07	*Monochroaarundinetella*	0.47
*Scrobipalpulapsilella*	0.21	0.64	*Scrobipalpulaseniorum*	0.53
*Scrobipalpulaseniorum*	N/A	0	*Scrobipalpulapsilella*	0.53
*Anacampsisblattariella*	0.48	2.99	*Anacampsispopulella*	0.56
*Anacampsispopulella*	0.22	1.41	*Anacampsisblattariella*	0.56
*Teleiopsisalbifemorella*	0.62	1.42	*Teleiopsisrosalbella*	0.61
*Teleiopsisbagriotella*	0.91	2.66	*Teleiopsisdiffinis*	0.61
*Teleiopsisdiffinis*	1.43	3.26	*Teleiopsisbagriotella*	0.61
*Teleiopsisrosalbella*	0.22	0.46	*Teleiopsisalbifemorella*	0.61
*Thiotrichacoleella*	N/A	0	*Thiotrichasubocellea*	0.67
*Thiotrichasubocellea*	0.74	1.4	*Thiotrichacoleella*	0.67
*Stomopteryxlineolella*	N/A	0	*Stomopteryxnugatricella*	0.77
*Stomopteryxnugatricella*	0	0	*Stomopteryxlineolella*	0.77
*Scrobipalpuladiffluella*	0.54	1.2	*Scrobipalpulatussilaginis*	0.8
*Scrobipalpulatussilaginis*	0.17	0.46	*Scrobipalpuladiffluella*	0.8
*Scrobipalpaarenbergeri*	0.49	0.77	*Scrobipalpamercantourica*	0.92
*Scrobipalpaartemisiella*	0.6	2.5	*Scrobipalpastangei*	0.92
*Scrobipalpamercantourica*	N/A	0	*Scrobipalpaarenbergeri*	0.92
*Scrobipalpasalicorniae*	0.16	0.46	*Scrobipalpasalinella*	0.92
*Scrobipalpasalinella*	0.28	0.92	*Scrobipalpasalicorniae*	0.92
*Scrobipalpastangei*	0.15	0.31	*Scrobipalpaartemisiella*	0.92
*Sattleriamelaleucella*	1.11	1.87	*Sattleriapyrenaica*	0.93
*Sattleriapyrenaica*	2.6	3.65	*Sattleriamelaleucella*	0.93
*Scrobipalpahalymella*	N/A	0	*Scrobipalpastabilis*	0.93
*Scrobipalpastabilis*	N/A	0	*Scrobipalpahalymella*	0.93

### Species delimitation from DNA barcode divergences

Intraspecific DNA barcode variation in the 741 named species with sequences ≥500 bp averaged 0.54%, but this may be an underestimate as sample sizes for 224 taxa were low and only represented by singletons. In respect to the distribution of mean intraspecific DNA barcode variation: 73.1% of sequenced species had variation ranging from 0–1%, 15.8% between 1–2%, 6.3% between 2–3%, and 4.8% > 3%.

Contrastingly, barcode gap analysis resulted in mean distances of 5.58% (maximum 12.75%) to the Nearest Neighbor (NN) with only 5.68% of all species showing a NN distance of 0–1% (Table 2). In this latter group, only four species pairs/triplets (*Dirhinosiacervinella* / *D.interposita*, *Iwarunabiguttella* / *I.klimeschi*, *Teleiodesbrevivalva* / *T.italica* / *T.vulgella*, *Xenolechiaaethiops* / *X.lindae* / *X.pseudovulgella*) shared barcodes so they could not be discriminated on that basis. In eight other cases, shared DNA barcodes meant that assignments were sometimes unreliable, but these species also possessed unique haplotypes (*Acompsiaantirrhinella* / *A.tripunctella*, *Anacampsisblattariella* / *A.populella*, *Bryotrophaaffinis* / *B.umbrosella*, *Sattleriapyrenaica* / *S.melaleucella*, *Scrobipalpaarenbergeri* / *S.mercantourica*, *Stomopteryxlineolella* / *S.nougatricella*, *Thiotrichasubocellea* / *T.coleella*, and partially also *Teleiopsisbagriotella* /*T.diffinis* / *T.paulheberti*). Finally, low distances between *Scrobipalpaalterna* / *S.lutea* and *S.halymella* / *S.stabilis* were only based on a single sequence for each of these species so they may represent additional cases of barcode overlap. On the other hand, five other species pairs with low interspecific divergence could be reliably separated by barcodes (*Monochroaarundinetella* / *M.suffusella*, *Scrobipalpastangei* / *S.artemisiella*, *Scrobipalpasalinella* / *S.salicorniae*, *Scrobipalpula* spp., *Teleiopsisrosalbella* / *T.albifemorella*). Considering all these cases, DNA barcodes showed either incomplete or no resolution for 31 species (4.2%), while species identification was effective for 710 species (95.8%).

**Table 2.** 46 species of European Gelechiidae assigned to multiple (3-22) BINs

Species	no. of BINs
*Aproaeremaanthyllidella*	3
*Aproaeremakarvoneni*	3
*Arogavelocella*	3
*Brachmiadimidiella*	3
*Bryotrophadesertella*	3
*Bryotrophaumbrosella*	3
*Caryocolumalsinella*	3
*Caryocolummarmorea*	3
*Caryocolumtischeriella*	3
*Chionodesfumatella*	3
*Chionodesviduella*	3
*Hypatimarhomboidella*	3
*Isophrictismeridionella*	3
*Megacraspedusbinotella*	3
*Metzneriaaprilella*	3
*Metzneriaartificella*	3
*Neofacultaericetella*	3
*Oxypteryxbaldizzonei*	3
*Parachronistisalbiceps*	3
*Ptocheuusapaupella*	3
*Stomopteryxflavipalpella*	3
*Teleiodesflavimaculella*	3
*Teleiodesluculella*	3
*Teleiopsispaulheberti*	3
*Arogaflavicomella*	4
*Caryocolumamaurella*	4
*Caryocolumfibigerium*	4
*Caryocolumperegrinella*	4
*Caryocolumvicinella*	4
*Ephysterispromptella*	4
*Gelechiasabinella*	4
*Isophrictisanthemidella*	4
*Megacraspedusimparellus*	4
*Metzneriametzneriella*	4
*Mirificarmacytisella*	4
*Athripsamoenella*	5
*Isophrictiskefersteiniellus*	5
*Megacraspedusbrachypteris*	5
*Monochroanomadella*	5
*Sattleriapyrenaica*	5
*Acompsiatripunctella*	6
*Caryocolumschleichi*	6
*Oxypteryxlibertinella*	7
*Stomopteryxremissella*	8
*Megacraspeduslanceolellus*	20
*Megacraspedusdolosellus*	22

### Species delimitation with Barcode Index Number (BIN) system

In total, 5877 sequences were assigned to a BIN. These records were assigned to 992 BINs that belong to 788 putative taxa (Suppl. material 2 and 3). Among these, 723 corresponded with named species, while another 65 belong to a unique BIN that is currently unidentified, but many likely represent additional, unrecognised species. Specimens from another 114 named species were assigned to more than one BIN; members of 68 species were placed in two BINs, while BIN counts for the other 46 species ranged from three to 22 (Table 2).

Altogether 668 (92.4%) of 723 named species have one or more unique BINs, while 55 species (7.6%) share a BIN with up to four species (Table 3). BIN sharing was particularly frequent in six genera (*Acompsia*, *Dirhinosia*, *Iwaruna*, *Scrobipalpula*, *Teleiopsis*, *Xenolechia*) where species often cannot be discriminated by DNA barcodes. However, most specimens in these taxa have diagnostic barcodes and all possess diagnostic morphological characters.

**Table 3.** Species of European Gelechiidae which share a BIN.

**Species**	**BIN**
*Acompsiaantirrhinella* / *A.pyrenaella* / *A.tripunctella*	BOLD:AAJ5937
*Anacampsisblattariella* / *A.populella*	BOLD:AAD3256
*Aproaeremaalbipalpella* / *A.cincticulella*	BOLD:ACB8811
*Aristoteliabrizella* / *A.confusella*	BOLD:AAJ1682
*Athripspruinosella* / *A.spiraeae*	BOLD:AAD2577
*Caryocolumarenbergeri* / *C.blandulella*	BOLD:AAV7765
*Dirhinosiacervinella* / *D.interposita*	BOLD:ACB0757
*Iwarunabiguttella* / *I.klimeschi* / *I.robineaui*	BOLD:AAU3602
*Metzneriafulva* / *M.torosulella*	BOLD:ADM4637
*Monochroaarundinetella* / *M.suffusella*	BOLD:AAF9390
*Monochroapalustrellus* / *M.saltenella*	BOLD:AAF2711
*Sattleriamelaleucella* / *S.pyrenaica*	BOLD:AAC5037
*Scrobipalpaalterna* / *S.lutea*	BOLD:ADR5476
*Scrobipalpaamseli* / *S.hyssopi*	BOLD:ADL8424
*Scrobipalpaartemisiella* / *S.stangei*	BOLD:AAE9838
*Scrobipalpahalymella* / *S.stabilis*	BOLD:AAV9005
*Scrobipalpasalicorniae* / *S.salinella*	BOLD:AAF1193
*Scrobipalpuladiffluella* / *S.psilella* / *S.ramosella* / *S.seniorum* / *S.tussilaginis*	BOLD:AAF1106
*Stomopteryxlineolella* / *S.mongolica* / *S.nugatricella*	BOLD:ACB3380
*Teleiodesbrevivalva* / *T.italica* / *T.vulgella*	BOLD:AAE9855
*Teleiopsisalbifemorella* / *T.rosalbella*	BOLD:AAB6930
*Teleiopsisbagriotella* / *T.diffinis* / *T.paulheberti*	BOLD:ACE4927
*Teleiopsisbagriotella* / *T.diffinis*	BOLD:ACE6105
*Xenolechiaaethiops* / *X.lindae* / *X.pseudovulgella*	BOLD:AAE1445

### Potential cryptic diversity – unrevised taxa

High levels of ‘intraspecific’ barcode variation often reflect overlooked species, but there is no fixed level of divergence that indicates species status. Furthermore, deep barcode splits can also arise as a result of the inadvertent recovery of pseudogenes, as a consequence of hybridisation, or *Wolbachia* infection (Mally et al. 2018, Werren et al. 2008). In Lepidoptera, 2–3% divergence is occasionally viewed as signalling the need for further integrative analysis (Hausmann et al. 2013), but there is clear evidence that no such threshold values exist (see e.g., Kekkonen et al. 2015). In the present dataset 146 of 741 nominal species possessed a maximum intraspecific divergence of > 2%, 88 species > 3%, while 33 species showed greater than > 5% (Table 4).

**Table 4.** 33 species of European Gelechiidae with a maximum intraspecific barcode divergence > 5%.

Species	Mean intra-spec.	Max intra-spec.
*Megacraspedusdolosellus*	7.49	13.76
*Megacraspeduslanceolellus*	7.37	12.51
*Monochroasepicolella*	5.15	9.78
*Megacraspedusbrachypteris*	4.36	7.82
*Stomopteryxremissella*	2.69	7.47
*Ephysterisdiminutella*	3.87	7.15
*Sophroniasicariellus*	1.34	7.06
*Caryocolumcauligenella*	1.86	7.00
*Acompsiapyrenaella*	3.58	6.92
*Caryocolumsaginella*	2.17	6.86
*Dichomerisrasilella*	3.31	6.67
*Monochroanomadella*	3.72	6.58
*Caryocolumschleichi*	3.93	6.47
*Acompsiatripunctella*	2.59	6.40
*Megacraspedusteriolensis*	3.07	6.38
*Caryocolumfibigerium*	3.41	6.31
*Chionodesfumatella*	2.6	6.30
*Oxypteryxbaldizzonei*	3.9	6.29
*Oxypteryxwilkella*	1.5	6.29
*Dichomerisjuniperella*	2.82	6.24
*Parapodia sinaica*	2.97	5.95
*Megacraspedusbalneariellus*	3.97	5.95
*Mirificarmaburdonella*	5.9	5.9
*Caryocolumperegrinella*	3.56	5.71
*Caryocolumalsinella*	2.11	5.60
*Oxypteryxlibertinella*	2.65	5.48
*Aproaeremasuecicella*	2.43	5.44
*Megacraspedusimparellus*	4.05	5.43
*Isophrictisanthemidella*	2.92	5.3
*Catatinagmatrivittellum*	5.24	5.24
*Pexicopiamalvella*	1.1	5.23
*Acompsiamaculosella*	2.16	5.19
*Ephysterispromptella*	3.31	5.12

In some recently revised taxa with high, geographically structured intraspecific barcode divergence such as *Megacraspedus* (Huemer and Karsholt 2018) or the *Oxypteryxlibertinella* species-group (Huemer et al. 2013), no evidence for cryptic diversity was found. However, even lower ‘intraspecific’ barcode divergence may reflect cases of either allopatric or sympatric speciation, as proven e.g., for the genus *Sattleria* (Huemer and Hebert 2011, Huemer and Timossi 2014). In consequence, several species with unusual genetic pattern need to be carefully re-assessed as they may include additional species. Cryptic diversity was, for example, already suspected for some *Caryocolum* (Huemer et al. 2015) or *Stomopteryxremissella*, but may also be detected in recently revised genera such as *Acompsia* or *Chionodes* (Huemer and Karsholt 2002, Huemer and Sattler 1995).

A further group of unrevised species in our dataset includes 65 unidentified DNA barcode clusters which were assigned to separate BINs (Table 5). Many of these cases are likely to represent undescribed species or alternatively, they may represent described species that currently lack barcode coverage. Altogether 26 genera representing approximately one-quarter of European genera are candidates for additional taxa. In fact, four genera (*Aproaerema*, *Aristotelia*, *Monochroa*, *Scrobipalpa*) are each represented by more than five unidentified clusters. For detailed comments on these cases, see Huemer and Karsholt (2020).

**Table 5.** Unidentified species of European Gelechiidae with unique BINs.

Taxon	BIN
*Anarsia*	BOLD:ADE9567
*Anarsia*	BOLD:ADE9710
*Apatetris*	BOLD:AAV7596
*Apatetris*	BOLD:ABA4360
*Aproaerema*	BOLD:AAT9258
*Aproaerema*	BOLD:ACF7323
*Aproaerema*	BOLD:ADG7311
*Aproaerema*	BOLD:ADL8444
*Aproaerema*	BOLD:ADL9068
*Aproaerema*	BOLD:ADL9069
*Aristotelia*	BOLD:AAU2122
*Aristotelia*	BOLD:AAV7599
*Aristotelia*	BOLD:ABV2430
*Aristotelia*	BOLD:ACC2990
*Aristotelia*	BOLD:ACK0360
*Aristotelia*	BOLD:ADC8189
*Aristotelia*	BOLD:ADK9648
*Aristotelia*	BOLD:ADL8520
*Aristotelia*	BOLD:ADL8769
*Aristotelia*	BOLD:ADL9120
*Aristotelia*	BOLD:ADM4599
*Aristotelia*	BOLD:ADY0927
*Brachmia*	BOLD:ADM5065
*Caulastrocecis*	BOLD:ADM1812
*Caulastrocecis*	BOLD:ADR7056
*Chrysoesthia*	BOLD:ADM8914
*Chrysoesthia*	BOLD:ADN7772
*Dichomeris*	BOLD:ADI2574
*Epidola*	BOLD:ADF2272
*Gelechia*	BOLD:ADF0061
*Gelechiidae*	BOLD:ADO2643
*Isophrictis*	BOLD:ADF3165
*Isophrictis*	BOLD:ADI3246
*Ivanauskiella*	BOLD:ACB0708
*Megacraspedus*	BOLD:ACZ8654
*Megacraspedus*	BOLD:ADY4582
*Mesophleps*	BOLD:AAU3614
*Mesophleps*	BOLD:ADM4492
*Metzneria*	BOLD:ABW1820
*Metzneria*	BOLD:ACB3385
*Metzneria*	BOLD:ADM8252
*Monochroa*	BOLD:ACF6594
*Monochroa*	BOLD:ACS5726
*Monochroa*	BOLD:ACW2532
*Monochroa*	BOLD:ADL7906
*Monochroa*	BOLD:ADL9322
*Monochroa*	BOLD:ADR3927
*Neofriseria*	BOLD:ADR5460
*Ochrodia*	BOLD:ACE0260
*Oxypteryx*	BOLD:ACR9491
*Oxypteryx*	BOLD:ACS7858
*Oxypteryx*	BOLD:ACS7859
*Psamathocrita*	BOLD:ADF0071
*Psamathocrita*	BOLD:ADL7901
*Ptocheuusa*	BOLD:AAV7056
*Scrobipalpa*	BOLD:AAV4547
*Scrobipalpa*	BOLD:ACT3383
*Scrobipalpa*	BOLD:ACT4605
*Scrobipalpa*	BOLD:ADF0070
*Scrobipalpa*	BOLD:ADG5400
*Scrobipalpa*	BOLD:ADL6932
*Scrobipalpa*	BOLD:ADL7117
*Sophronia*	BOLD:ADF5021
*Stomopteryx*	BOLD:ADM5270
*Telphusa*	BOLD:ADM5148

## Discussion

During the past decade, several national DNA barcoding campaigns have led to the development of an increasingly well-parameterised DNA barcode library for European Lepidoptera. However, these projects have mainly focused on the fauna of central and northern Europe. As a consequence, genetic coverage for species in the Mediterranean region remains patchy. Reflecting this fact, continent-wide analysis has only considered a few groups so far, such as Nepticulidae (van Nieukerken pers. comm.), Gracillariidae (Lopez-Vaamonde pers. comm.), Elachistinae (Mutanen et al. 2011), Depressariidae (Buchner pers. comm), Geometridae (Hausmann et al. 2013, Müller et al. 2019), and Papilionoidea (Dincă pers. comm.). By contrast, for most families either few DNA barcodes exist, or comprehensive genetic analysis is not available.

**Figure 3.**
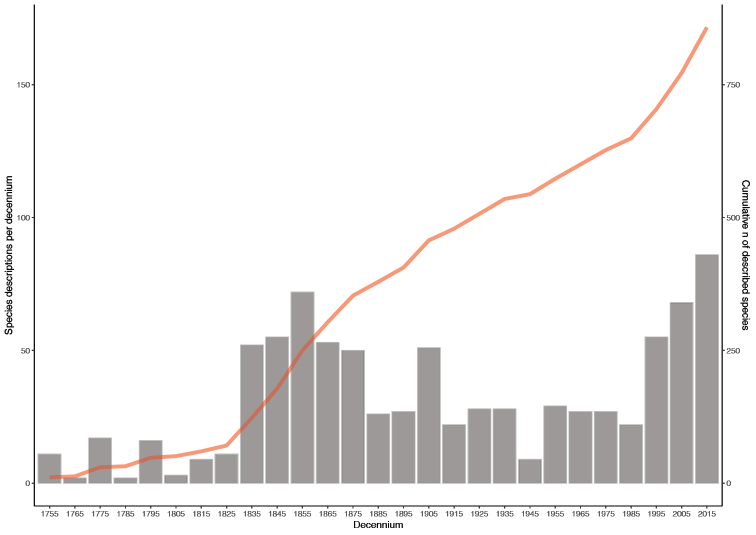
Periods of descriptions of European Gelechiidae.

The current DNA barcode library makes it clear that the Gelechiidae is a particularly good example of the serious gaps in the knowledge of European biodiversity. Nearly a quarter of current fauna has been described since 1990 (Fig. 3). This gap between European gelechiid diversity and adequate coverage in published alpha-taxonomy is most probably a result of: 1) the small number of gelechiid experts, 2) the lack of adequate vouchers for phenotypic and molecular study 3) the frequently cryptic morphology making them less attractive to non-expert workers, and 4) the infrequent consideration of molecular data to assess taxonomic boundaries.

In the present study, DNA sequences revealed a high level of possible cryptic diversity in European Gelechiidae, despite extensive revisionary work over the last decades (see e.g., Huemer and Karsholt 1999, 2010). Although almost 96% of all 741 species possessed unique barcodes, intraspecific divergences exceeded 2% in nearly a fifth of currently recognised species, and 33 of these cases of divergence values exceeded 5%, values that likely signal overlooked species.

The intraspecific DNA barcode variation is reflected in some taxa as allopatric divergence, but in other cases, it reflects sympatric deep splits. However, few of these species have received detailed taxonomic assessment such as the recent comprehensive study on *Megacraspedus* (Huemer and Karsholt 2018). In many other unrevised genera/species-groups a significant increase in species diversity is likely. The major gaps in taxonomic treatment of European Gelechiidae are further demonstrated by the large number of unidentified genetic clusters revealed by the present investigation as many of these 65 putative taxa are likely to represent undescribed species.

## Conclusions

By providing coverage for 751 species of European Gelechiidae, the current DNA barcode library represents the largest release in terms of species diversity for any family of Lepidoptera on this continent. The results reveal unexpected genetic diversity in many taxa as well as numerous unidentified taxa. This indicates that the alpha-taxonomy of this family, still requires serious attention despite one-quarter of the known species described after 1990. The current results indicate that the Gelechiidae remain one of the most taxonomically challenging families of Lepidoptera in the World as complete coverage of even European fauna will require extensive effort. However, the DNA barcode library generated in this study will allow these revisionary studies to target groups that are particularly problematic, accelerating the documentation of the fauna.

## Supplementary materials

Supplementary material 1 Barcoded species Data type: species data https://binary.pensoft.net/file/392662This dataset is made available under the Open Database License (http://opendatacommons.org/licenses/odbl/1.0/). The Open Database License (ODbL) is a license agreement intended to allow users to freely share, modify, and use this Dataset while maintaining this same freedom for others, provided that the original source and author(s) are credited.Peter Huemer, Ole Karsholt, Leif Aarvik, Kai Berggren, Oleksiy Bidzilya, Jari Junnilainen, Jean-François Landry, Marko Mutanen, Kari Nupponen, Andreas Segerer, Jan Šumpich, Christian Wieser, Benjamin Wiesmair, Paul D.N. Hebert

Supplementary material 2 NJ trees 1–53 Data type: species data https://binary.pensoft.net/file/392663This dataset is made available under the Open Database License (http://opendatacommons.org/licenses/odbl/1.0/). The Open Database License (ODbL) is a license agreement intended to allow users to freely share, modify, and use this Dataset while maintaining this same freedom for others, provided that the original source and author(s) are credited.Peter Huemer, Ole Karsholt, Leif Aarvik, Kai Berggren, Oleksiy Bidzilya, Jari Junnilainen, Jean-François Landry, Marko Mutanen, Kari Nupponen, Andreas Segerer, Jan Šumpich, Christian Wieser, Benjamin Wiesmair, Paul D.N. Hebert

Supplementary material 3 Specimen data to NJ trees 1–53 Data type: specimen data https://binary.pensoft.net/file/392664This dataset is made available under the Open Database License (http://opendatacommons.org/licenses/odbl/1.0/). The Open Database License (ODbL) is a license agreement intended to allow users to freely share, modify, and use this Dataset while maintaining this same freedom for others, provided that the original source and author(s) are credited.Peter Huemer, Ole Karsholt, Leif Aarvik, Kai Berggren, Oleksiy Bidzilya, Jari Junnilainen, Jean-François Landry, Marko Mutanen, Kari Nupponen, Andreas Segerer, Jan Šumpich, Christian Wieser, Benjamin Wiesmair, Paul D.N. Hebert
