# Supplementary material for: DNA barcode library for European Gelechiidae (Lepidoptera) suggests greatly underestimated species diversity
Source: Zookeys. 2020 Mar 24;921:141–57. doi: 10.3897/zookeys.921.49199 (PMC7109146; doi:10.3897/zookeys.921.49199)
Supplement: Supplementary material 1 [file zookeys-921-141-s001.pdf]

Supplementary material 1

Barcoded species

Checklist after Huemer & Karsholt (2020)

BC = DNA barcode sequence ≥ 500 bp available; (BC) DNA barcode sequence <500 bp and ≥250 bp available

**Taxon**	**Barcode**
**Gelechiidae Stainton, 1854**	
**Anacampsinae Bruand d'Uzelle, 1851**	
**Anacampsini Bruand d'Uzelle, 1851**	
***Stomopteryx* Heinemann, 1870**	BC
Stomopteryx detersella (Zeller, 1847)	BC
Stomopteryx bolschewickiella (Caradja, 1920)	
Stomopteryx nugatricella Rebel, 1893	BC
Stomopteryx mongolica Piskunov, 1975	BC
Stomopteryx lineolella (Eversmann, 1844)	BC
Stomopteryx basalis (Staudinger, 1876)	BC
Stomopteryx deverrae (Walsingham, 1905)	BC
Stomopteryx flavoclavella Zerny, 1935	BC
Stomopteryx remissella (Zeller, 1847)	BC
Stomopteryx spathulella Nel, Varenne & Labonne, 2019	
Stomopteryx orthogonella (Staudinger, 1871)	
Stomopteryx orthogonella (Staudinger, 1871)	
Stomopteryx flavipalpella Jäckh, 1959	BC
Stomopteryx hungaricella Gozmány, 1957	BC
Stomopteryx lusitaniella Corley & Karsholt, 2014	BC
Stomoptery jeppeseni Karsholt & Šumpich, 2018	BC
Stomopteryx alpinella Nel & Varenne, 2016	BC
Stomopteryx schizogynae (Walsingham, 1908)	
***Aproaerema* Durrant, 1897**	
Aproaerema patruella (Mann, 1857)	BC
Aproaerema coronillella (Treitschke, 1833)	BC
Aproaerema incognitana (Gozmány, 1957)	BC

Aproaerema sangiella (Stainton, 1863)	BC
Aproaerema cinctella (Clerck, 1759)	BC
Aproaerema cinctelloides (Nel & Varenne, 2012)	BC
Aproaerema larseniella (Gozmány, 1957)	BC
Aproaerema wormiella (Wolff, 1958)	BC
Aproaerema azosterella (Herrich-Schäffer, 1854)	BC
Aproaerema ochrofasciella (Toll, 1936)	BC
Aproaerema taeniolella (Zeller, 1839)	BC
Aproaerema montanata (Gozmány, 1957)	
Aproaerema albifrontella (Heinemann, 1870)	BC
Aproaerema cincticulella (Bruand, 1851)	BC
Aproaerema vinella Bankes, 1898	BC
Aproaerema buvati (Nel, 1995)	BC
Aproaerema linella (Chrétien, 1904)	BC
Aproaerema albipalpella (Herrich-Schäffer, 1854)	BC
Aproaerema suecicella (Wolff, 1958)	BC
Aproaerema captivella (Herrich-Schäffer, 1854)	BC
Aproaerema polychromella (Rebel, 1902)	BC
Aproaerema karvoneni (Hackman, 1950)	BC
Aproaerema semicostella (Staudinger, 1871)	BC
Aproaerema steppicolella (Junnilainen, 2010)	BC
Aproaerema cottiennella (Nel, 2012)	
Aproaerema genistae (Walsingham, 1908)	BC
Aproaerema thaumalea (Walsingham, 1905)	BC
Aproaerema anthyllidella (Hübner, 1813)	BC
Aproaerema lerauti Vives, 2001	BC
Aproaerema mercedella Walsingham, 1908	BC
***Iwaruna* Gozmány, 1957**	
Iwaruna heringi Gozmány, 1957	
Iwaruna biguttella (Duponchel, 1843)	BC
Iwaruna klimeschi Wolff, 1958	BC
Iwaruna robineaui Nel, 2008	BC
***Anacampsis* Curtis, 1827**	
Anacampsis populella (Clerck, 1759)	BC

Anacampsis blattariella (Hübner, 1796)	BC
Anacampsis timidella (Wocke, 1887)	BC
Anacampsis scintillella (Fischer v. Röslerstamm, 1841)	BC
Anacampsis temerella (Lienig & Zeller, 1846)	BC
Anacampsis trifoliella (Constant, 1890)	BC
Anacampsis fuscella (Eversmann, 1844)	BC
Anacampsis hirsutella (Constant, 1885)	
Anacampsis obscurella ([Denis & Schiffermüller], 1775)	BC
Anacampsis malella Amsel, 1959	
***Mesophleps* Hübner, 1825**	
Mesophleps corsicella (Herrich-Schäffer, 1856)	BC
Mesophleps silacella (Hübner, 1796)	BC
Mesophleps oxycedrella (Millière, 1871)	BC
Mesophleps trinotella Herrich-Schäffer, 1856	BC
Mesophleps ochracella (Turati, 1926)	BC
**Chelariini Le Marchand, 1947**	
***Nothris* Hübner, 1825**	
Nothris congressariella (Bruand, 1858)	BC
Nothris lemniscellus (Zeller, 1839)	BC
Nothris gregerseni Karsholt & Šumpich, 2015	BC
Nothris verbascella ([Denis & Schiffermüller], 1775)	BC
Nothris sulcella Staudinger, 1879	BC
Nothris radiata (Staudinger, 1879)	BC
Nothris skyvai Karsholt & Šumpich, 2015	BC
***Neofaculta* Gozmány, 1955**	
Neofaculta ericetella (Geyer, 1832)	BC
Neofaculta infernella (Herrich-Schäffer, 1854)	BC
Neofaculta taigana Ponomarenko, 1998	BC
***Hypatima* Hübner, 1825**	
Hypatima rhomboidella (Linnaeus, 1758)	BC
***Anarsia* Zeller, 1839**	
Anarsia lineatella Zeller, 1839	BC
Anarsia innoxiella Gregersen & Karsholt, 2017	BC
Anarsia spartiella (Schrank, 1802)	BC

Anarsia bilbainella (Rössler, 1877)	BC
Anarsia eleagnella Kuznetsov, 1957	BC
Anarsia dejoannisi Réal, 1994	
Anarsia leberonella Réal, 1994	BC
Anarsia sibirica Park & Ponomarenko, 1996	BC
Anarsia stepposella Ponomarenko, 2002	
Anarsia acaciae Walsingham, 1896	BC
Anarsia balioneura Meyrick, 1921	
**Dichomeridinae Hampson, 1918**	
***Dichomeris* Hübner, 1818**	
Dichomeris acuminatus (Staudinger, 1876)	BC
Dichomeris cisti (Staudinger, 1859)	BC
Dichomeris limbipunctellus (Staudinger, 1859)	BC
Dichomeris neatodes Meyrick, 1923	BC
Dichomeris helianthemi (Walsingham, 1903)	BC
Dichomeris castellana (Schmidt, 1941)	
Dichomeris juniperella (Linnaeus, 1761)	BC
Dichomeris marginella (Fabricius, 1781)	BC
Dichomeris ustalella (Fabricius, 1794)	BC
Dichomeris derasella ([Denis & Schiffermüller], 1775)	BC
Dichomeris limosellus (Schläger, 1849)	BC
Dichomeris nitiellus (Costantini, 1923)	BC
Dichomeris rasilella (Herrich-Schäffer, 1854)	BC
Dichomeris barbella ([Denis & Schiffermüller], 1775)	BC
Dichomeris alacella (Zeller, 1839)	BC
Dichomeris latipennella (Rebel, 1937)	BC
***Anasphaltis* Meyrick, 1925**	
Anasphaltis renigerellus (Zeller, 1839)	BC
***Acompsia* Hübner, 1825**	
Acompsia cinerella (Clerck, 1759)	BC
Acompsia pyrenaella Huemer & Karsholt, 2002	BC
Acompsia antirrhinella Millière, 1866	BC
Acompsia baldizzonei Pinzari, Nel & Pinzari, 2016	BC
Acompsia maculosella (Stainton, 1851)	BC

Acompsia dimorpha Petry, 1904	BC
Acompsia subpunctella Svensson, 1966	BC
Acompsia delmastroella Huemer, 1998	BC
Acompsia muellerrutzi Wehrli, 1925	
Acompsia caucasella Huemer & Karsholt, 2002	
Acompsia minorella Rebel, 1899	BC
Acompsia tripunctella ([Denis & Schiffermüller], 1775)	BC
Acompsia ponomarenkoae Huemer & Karsholt, 2002	BC
Acompsia schmidtiellus (Heyden, 1848)	BC
***Brachmia* Hübner, 1825**	
Brachmia dimidiella ([Denis & Schiffermüller], 1775)	BC
Brachmia blandella (Fabricius, 1798)	BC
Brachmia procursella Rebel, 1903	BC
Brachmia inornatella (Douglas, 1850)	BC
***Helcystogramma* Zeller, 1877**	
Helcystogramma lineolella (Zeller, 1839)	BC
Helcystogramma triannulella (Herrich-Schäffer, 1854)	BC
Helcystogramma lutatella (Herrich-Schäffer, 1854)	BC
Helcystogramma rufescens (Haworth, 1828)	BC
Helcystogramma albinervis (Gerasimov, 1929)	BC
Helcystogramma arulensis (Rebel, 1929)	BC
Helcystogramma klimeschi Ponomarenko & Huemer, 2001	
Helcystogramma flavescens Junnilainen, 2010	BC
Helcystogramma convolvuli (Walsingham, 1908)	
Helcystogramma lamprostoma (Zeller, 1847)	BC
***Pseudosophronia* Corley, 2001**	
Pseudosophronia exustellus (Zeller, 1847)	BC
Pseudosophronia cosmella (Constant, 1885)	BC
**Apatetrinae Le Marchand, 1947**	
**Pexicopiini Hodges, 1986**	
***Harpagidia* Ragonot, 1895**	
Harpagidia magnetella (Staudinger, 1871)	BC
***Pectinophora* Busck, 1917**	
Pectinophora gossypiella (Saunders, 1844)	BC

***Pexicopia* Common, 1958**	
Pexicopia malvella (Hübner, 1805)	BC
***Platyedra* Meyrick, 1895**	
Platyedra subcinerea (Haworth, 1828)	BC
***Sitotroga* Heinemann, 1870**	
Sitotroga psacasta Meyrick, 1908	BC
Sitotroga cerealella (Olivier, 1789)	BC
**Apatetrini Le Marchand, 1947**	
***Dactylotula* Cockerell, 1888**	
Dactylotula altithermella (Walsingham, 1903)	
Dactylotula kinkerella (Snellen, 1876)	BC
***Apatetris* Staudinger, 1879**	
Apatetris agenjoi Gozmány, 1954	BC
Apatetris mediterranella Nel & Varenne, 2012	BC
***Catatinagma* Rebel, 1903**	
Catatinagma trivittellum Rebel, 1903	BC
Catatinagma kraterella Junnilainen & Nupponen, 2010	BC
***Coloptilia* Fletcher, 1940**	
Coloptilia conchylidella (Hofmann, 1898)	BC
***Chrysoesthia* Hübner, 1825**	
Chrysoesthia drurella (Fabricius, 1775)	BC
Chrysoesthia eppelsheimi (Staudinger, 1885)	BC
Chrysoesthia verrucosa Tokár, 1999	BC
Chrysoesthia sexguttella (Thunberg, 1794)	BC
Chrysoesthia halimionella Bidzilya & Budashkin, 2015	BC
Chrysoesthia atriplicella (Amsel, 1939)	
Chrysoesthia gaditella (Staudinger, 1859)	BC
Chrysoesthia aletris (Walsingham, 1919)	
Chrysoesthia boseae (Walsingham, 1908)	BC
Chrysoesthia falkovitshi Lvovsky & Piskunov, 1989	(BC)
Chrysoesthia hispanica Karsholt & Vives, 2014	BC
***Metanarsia* Staudinger, 1871**	
Metanarsia modesta Staudinger, 1871	BC
Metanarsia onzella Christoph, 1887	

Metanarsia guberlica Nupponen, 2010	BC
Metanarsia incertella (Herrich-Schäffer, 1861)	BC
***Oecocecis* Guenée, 1870**	
Oecocecis guyonella Guenée, 1870	
**Thiotrichinae Karsholt, Mutanen, Lee & Kaila, 2013**	
***Thiotricha* Meyrick, 1886**	
Thiotricha majorella (Rebel, 1910)	BC
Thiotricha subocellea (Stephens, 1834)	BC
Thiotricha coleella (Constant, 1885)	
Thiotricha wollastoni (Walsingham, 1884)	BC
***Palumbina* Rondani, 1876**	
Palumbina guerinii (Stainton, 1858)	BC
**Anomologinae Meyrick, 1926**	
***Bryotropha* Heinemann, 1870**	
Bryotropha sabulosella (Rebel, 1905)	BC
Bryotropha domestica (Haworth, 1828)	BC
Bryotropha vondermuhlli Nel & Brusseaux, 2003	BC
Bryotropha rossica Anikin & Piskunov, 1996	BC
Bryotropha azovica Bidzilia, 1997	BC
Bryotropha arabica Amsel, 1952	BC
Bryotropha patockai Elsner & Karsholt, 2003	BC
Bryotropha purpurella (Zetterstedt, 1839)	BC
Bryotropha tachyptilella (Rebel, 1916)	BC
Bryotropha italica Karsholt & Rutten, 2005	BC
Bryotropha politella (Stainton, 1851)	BC
Bryotropha aliterrella (Rebel, 1935)	BC
Bryotropha nupponeni Karsholt & Rutten, 2005	BC
Bryotropha satschkovi Anikin & Piskunov, 2018	
Bryotropha terrella ([Denis & Schiffermüller], 1775)	BC
Bryotropha sattleri Nel, 2003	BC
Bryotropha desertella (Douglas, 1850)	BC
Bryotropha wolschrijni Karsholt & Rutten, 2005	BC
Bryotropha heckfordi Karsholt & Rutten, 2005	BC
Bryotropha figulella (Staudinger, 1859)	BC

Bryotropha plantariella (Tengström, 1848)	BC
Bryotropha galbanella (Zeller, 1839)	BC
Bryotropha boreella (Douglas, 1851)	BC
Bryotropha sutteri Karsholt & Rutten, 2005	BC
Bryotropha gallurella Amsel, 1952	BC
Bryotropha hendrikseni Karsholt & Rutten, 2005	BC
Bryotropha pallorella Amsel, 1952	BC
Bryotropha hulli Karsholt & Rutten, 2005	BC
Bryotropha plebejella (Zeller, 1847)	BC
Bryotropha dryadella (Zeller, 1850)	BC
Bryotropha basaltinella (Zeller, 1839)	BC
Bryotropha affinis (Haworth, 1828)	BC
Bryotropha umbrosella (Zeller, 1839)	BC
Bryotropha similis (Stainton, 1854)	BC
Bryotropha senectella (Zeller, 1839)	BC
***Epidola* Staudinger, 1859**	
Epidola stigma Staudinger, 1859	BC
Epidola barcinonella Millière, 1867	BC
Epidola semitica Amsel, 1942	BC
Epidola nuraghella Hartig, 1939	BC
Epidola melitensis Amsel, 1955	
***Aristotelia* Hübner, 1825**	
Aristotelia decurtella (Hübner, 1813)	BC
Aristotelia decoratella (Staudinger, 1879)	BC
Aristotelia leonhardi Krone, 1907	
Aristotelia ericinella (Zeller, 1839)	BC
Aristotelia subdecurtella (Stainton, 1859)	BC
Aristotelia subericinella (Duponchel, 1843)	BC
Aristotelia billii Varenne & Nel, 2013	BC
Aristotelia montarcella Schmidt, 1941	BC
Aristotelia heliacella (Herrich-Schäffer, 1854)	BC
Aristotelia pancaliella (Staudinger, 1871)	(BC)
Aristotelia baltica Sulcs & Sulcs, 1983	BC
Aristotelia brizella (Treitschke, 1833)	BC

Aristotelia brizelloidea Amsel, 1935	
Aristotelia confusella Bidzilya & Budashkin, 2015	BC
Aristotelia staticella Millière, 1876	BC
Aristotelia mirandella Chrétien, 1908	
Aristotelia frankeniae Walsingham, 1898	BC
Aristotelia calastomella (Christoph, 1873)	BC
Aristotelia mirabilis (Christoph, 1888)	BC
***Caulastrocecis* Chrétien, 1931**	BC
Caulastrocecis pudicellus (Mann, 1861)	
Caulastrocecis gypsella (Constant, 1893)	BC
Caulastrocecis furfurella (Staudinger, 1871)	BC
Caulastrocecis cryptoxena (Gozmány, 1952)	BC
Caulastrocecis perexigella Junnilainen, 2010	BC
Caulastrocecis interstratella (Christoph, 1873)	BC
***Paranarsia* Ragonot, 1895**	
Paranarsia joannisiella Ragonot, 1895	BC
***Megacraspedus* Zeller, 1839**	
Megacraspedus lanceolellus (Zeller, 1850)	BC
Megacraspedus bengtssoni Huemer & Karsholt, 2018	BC
Megacraspedus junnilaineni Huemer & Karsholt, 2018	BC
Megacraspedus uzunsyrtus Bidzilya & Budashkin, 2015	BC
Megacraspedus similellus Huemer & Karsholt, 2018	BC
Megacraspedus tokari Huemer & Karsholt, 2018	BC
Megacraspedus dolosellus (Zeller, 1839)	BC
Megacraspedus neli Huemer & Karsholt, 2018	BC
Megacraspedus faunierensis Huemer & Karsholt, 2018	BC
Megacraspedus gredosensis Huemer & Karsholt, 2018	BC
Megacraspedus cuencellus Caradja, 1920	BC
Megacraspedus bidentatus Huemer & Karsholt, 2018	BC
Megacraspedus fuscus Huemer & Karsholt, 2018	BC
Megacraspedus trineae Huemer & Karsholt, 2018	BC
Megacraspedus tristictus Walsingham, 1910	BC
Megacraspedus alfacarellus Wehrli, 1926	BC
Megacraspedus pusillus Walsingham, 1903	BC

Megacraspedus skoui Huemer & Karsholt, 2018	BC
Megacraspedus spinophallus Huemer & Karsholt, 2018	BC
Megacraspedus occidentellus Huemer & Karsholt, 2018	BC
Megacraspedus granadensis Huemer & Karsholt, 2018	
Megacraspedus heckfordi Huemer & Karsholt, 2018	BC
Megacraspedus tenuiuncus Huemer & Karsholt, 2018	BC
Megacraspedus lativalvellus Amsel, 1954	
Megacraspedus dejectella (Staudinger, 1859)	BC
Megacraspedus devorator Huemer & Karsholt, 2018	BC
Megacraspedus binotella (Duponchel, 1843)	BC
Megacraspedus brachypteris Huemer & Karsholt, 2018	BC
Megacraspedus barcodiellus Huemer & Karsholt, 2018	BC
Megacraspedus bilineatella Huemer & Karsholt, 1996	BC
Megacraspedus andreneli Varenne & Nel, 2014	BC
Megacraspedus sumpichi Huemer & Karsholt, 2018	BC
Megacraspedus gallicus Huemer & Karsholt, 2018	BC
Megacraspedus ribbeella (Caradja, 1920)	BC
Megacraspedus numidellus (Chrétien, 1915)	BC
Megacraspedus albovenata Junnilainen, 2010	BC
Megacraspedus longipalpella Junnilainen, 2010	BC
Megacraspedus niphorrhoa (Meyrick, 1926)	BC
Megacraspedus fallax (Mann, 1867)	BC
Megacraspedus balneariellus (Chrétien, 1907)	BC
Megacraspedus podolicus (Toll, 1942)	BC
Megacraspedus knudlarseni Huemer & Karsholt, 2018	BC
Megacraspedus imparellus (Fischer v. Röslerstamm, 1843)	BC
Megacraspedus multispinella Junnilainen & Nupponen, 2010	BC
Megacraspedus cerussatellus Rebel, 1930	BC
Megacraspedus attritellus Staudinger, 1871	BC
Megacraspedus lagopellus (Herrich-Schäffer, 1860)	BC
Megacraspedus argyroneurellus Staudinger, 1871	BC
Megacraspedus ibericus Huemer & Karsholt, 2018	BC
Megacraspedus squalida Meyrick, 1926	BC
Megacraspedus pentheres Walsingham, 1920	

Megacraspedus teriolensis Huemer & Karsholt, 2018	BC
Megacraspedus korabicus Huemer & Karsholt, 2018	BC
Megacraspedus quadristictus Lhomme, 1946	BC
Megacraspedus eburnellus Huemer & Karsholt, 2001	BC
Megacraspedus skulei Huemer & Karsholt, 2018	BC
Megacraspedus peyerimhoffi Le Cerf, 1925	BC
Megacraspedus peslieri Huemer & Karsholt, 2018	BC
***Dirhinosia* Rebel, 1905**	
Dirhinosia cervinella (Eversmann, 1844)	BC
Dirhinosia arnoldiella (Rebel, 1905)	BC
Dirhinosia interposita Bidzilya & Budashkin, 2015	BC
***Psamathocrita* Meyrick, 1925**	
Psamathocrita osseella (Stainton, 1860)	BC
Psamathocrita argentella Pierce & Metcalfe, 1942	BC
Psamathocrita dalmatinella Huemer & Tokár, 2000	BC
***Chimericorsa* Varenne, Huemer & Nel, 2017**	
Chimericorsa nioloensis Varenne, Huemer & Nel, 2017	BC
***Spiniphallellus* Bidzilya & Karsholt, 2008**	
Spiniphallellus desertus Bidzilya & Karsholt, 2008	BC
Spiniphallellus chrysotosella Junnilainen, 2016	BC
***Deltpophora* Janse, 1950**	
Deltophora maculata (Staudinger, 1879)	BC
Deltophora stictella (Rebel, 1927)	BC
Deltophora gielisia Hull, 1995	(BC)
***Ivanauskiella* Ivinskis & Piskunov, 1980**	
Ivanauskiella psamathias (Meyrick, 1891)	BC
Ivanauskiella occitanica (Nel & Varenne, 2013)	BC
***Ptocheuusa* Heinemann, 1870**	
Ptocheuusa paupella (Zeller, 1847)	BC
Ptocheuusa inopella (Zeller, 1839)	BC
Ptocheuusa abnormella (Herrich-Schäffer, 1854)	BC
Ptocheuusa minimella (Rebel, 1936)	BC
Ptocheuusa asterisci (Walsingham, 1903)	
Ptocheuusa scholastica (Walsingham, 1903)	

Ptocheuusa guimarensis (Walsingham, 1908)	
Ptocheuusa sublutella Christoph, 1873	
Ptocheuusa cinerella (Chrétien, 1908)	
***Gladiovalva* Sattler, 1960**	
Gladiovalva rumicivorella (Millière, 1881)	BC
Gladiovalva aizpuruai Vives, 1990	BC
Gladiovalva badidorsella (Rebel, 1935)	BC
***Ornativalva* Gozmány, 1955**	
Ornativalva heluanensis (Debski, 1913)	BC
Ornativalva ornatella Sattler, 1967	BC
Ornativalva tamariciella (Zeller, 1850)	BC
Ornativalva pseudotamariciella Sattler, 1967	BC
Ornativalva antipyramis (Meyrick, 1925)	(BC)
Ornativalva plutelliformis (Staudinger, 1859)	BC
Ornativalva sieversi (Staudinger, 1871)	
Ornativalva mixolitha (Meyrick, 1918)	BC
***Atremaea* Staudinger, 1871**	
Atremaea lonchoptera Staudinger, 1871	BC
***Amblypalpis* Ragonot, 1886**	
Amblypalpis olivierella Ragonot, 1887	
***Parapodia* Joannis, 1912**	
Parapodia sinaica (Frauenfeld, 1859)	BC
***Isophrictis* Meyrick, 1917**	
Isophrictis robinella (Chrétien, 1907)	
Isophrictis meridionella (Herrich-Schäffer, 1854)	BC
Isophrictis constantina (Baker, 1888)	
Isophrictis cerdanica Nel, 1995	
Isophrictis lineatellus (Zeller, 1850)	BC
Isophrictis kefersteiniellus (Zeller, 1850)	BC
Isophrictis striatella ([Denis & Schiffermüller], 1775)	BC
Isophrictis corsicella Amsel, 1936	BC
Isophrictis invisella (Constant, 1885)	
Isophrictis anthemidella (Wocke, 1871)	BC
Isophrictis impugnata Gozmány, 1957	

***Pyncostola* Meyrick, 1917**	
Pyncostola bohemiella (Nickerl, 1864)	BC
***Metzneria* Zeller, 1839**	
Metzneria paucipunctella (Zeller, 1839)	BC
Metzneria tenuiella (Mann, 1864)	BC
Metzneria neuropterella (Zeller, 1839)	BC
Metzneria aestivella (Zeller, 1839)	BC
Metzneria lappella (Linnaeus, 1758)	BC
Metzneria castiliella (Möschler, 1866)	BC
Metzneria littorella (Douglas, 1850)	BC
Metzneria riadella Englert, 1974	BC
Metzneria diffusella Englert, 1974	BC
Metzneria fulva Labonne, Huemer, Thibault & Nel, 2019	BC
Metzneria torosulella (Rebel, 1893)	BC
Metzneria ehikeella Gozmány, 1954	BC
Metzneria metzneriella (Stainton, 1851)	BC
Metzneria hilarella Caradja, 1920	BC
Metzneria staehelinella Englert, 1974	BC
Metzneria artificella (Herrich-Schäffer, 1861)	BC
Metzneria agraphella (Ragonot, 1895)	BC
Metzneria aprilella (Herrich-Schäffer, 1854)	BC
Metzneria subflavella Englert, 1974	BC
Metzneria filia Piskunov, 1979	
Metzneria intestinella (Mann, 1864)	BC
Metzneria santolinella (Amsel, 1936)	BC
Metzneria tristella Rebel, 1901	BC
Metzneria campicolella (Mann, 1857)	BC
***Apodia* Heinemann, 1870**	
Apodia bifractella (Duponchel, 1843)	BC
Apodia martinii Petry, 1911	BC
***Pragmatodes* Walsingham, 1908**	
Pragmatodes fruticosella Walsingham, 1908	
Pragmatodes melagonella (Constant, 1895)	BC
Pragmatodes albagonella (Varenne & Nel, 2010)	BC

Pragmatodes cyrneogonella (Nel & Varenne, 2012)	BC
Pragmatodes parvulata (Gozmány, 1953)	BC
***Argolamprotes* Benander, 1945**	
Argolamprotes micella ([Denis & Schiffermüller], 1775)	BC
***Monochroa* Heinemann, 1870**	
Monochroa rumicetella (Hofmann, 1868)	BC
Monochroa rebeli (Hering, 1927)	BC
Monochroa sepicolella (Herrich-Schäffer, 1854)	BC
Monochroa rectifasciella (Fuchs, 1902)	BC
Monochroa tenebrella (Hübner, 1817)	BC
Monochroa scutatella (Müller-Rutz, 1920)	BC
Monochroa dellabeffai (Rebel, 1932)	BC
Monochroa servella (Zeller, 1839)	BC
Monochroa conspersella (Herrich-Schäffer, 1854)	BC
Monochroa tetragonella (Stainton, 1885)	BC
Monochroa elongella (Heinemann, 1870)	BC
Monochroa inflexella Svensson, 1992	BC
Monochroa sperata Huemer & Karsholt, 2010	BC
Monochroa lutulentella (Zeller, 1839)	BC
Monochroa aenigma Anikin & Piskunov, 2018	
Monochroa saltenella (Benander, 1928)	BC
Monochroa palustrellus (Douglas, 1850)	BC
Monochroa divisella (Douglas, 1850)	BC
Monochroa lucidella (Stephens, 1834)	BC
Monochroa simplicella (Lienig & Zeller, 1846)	BC
Monochroa moyses Uffen, 1991	BC
Monochroa arundinetella (Stainton, 1858)	BC
Monochroa suffusella (Douglas, 1850)	BC
Monochroa cytisella (Curtis, 1837)	BC
Monochroa ferrea (Frey, 1870)	BC
Monochroa nomadella (Zeller, 1868)	BC
Monochroa bronzella Karsholt, Nel, Fournier, Varenne & Huemer, 2013	BC
Monochroa hornigi (Staudinger, 1883)	BC
Monochroa niphognatha (Gozmány, 1953)	BC

***Oxypteryx* Rebel, 1911**	
Oxypteryx nigromaculella (Millière, 1872)	BC
Oxypteryx wilkella (Linnaeus, 1758)	BC
Oxypteryx ochricapilla (Rebel, 1903)	BC
Oxypteryx superbella (Zeller, 1839)	BC
Oxypteryx mirusella (Huemer & Karsholt, 2013)	BC
Oxypteryx baldizzonei (Karsholt & Huemer, 2013)	BC
Oxypteryx occidentella (Huemer & Karsholt, 2011)	BC
Oxypteryx libertinella (Zeller, 1872)	BC
Oxypteryx gemerensis (Elsner, 2013)	BC
Oxypteryx deserta (Piskunov, 1990)	
Oxypteryx unicolorella (Duponchel, 1843)	BC
Oxypteryx atrella ([Denis & Schiffermüller], 1775)	BC
Oxypteryx nigritella (Zeller, 1847)	BC
Oxypteryx immaculatella (Douglas, 1850)	BC
Oxypteryx plumbella (Heinemann, 1870)	BC
Oxypteryx isostacta (Meyrick, 1926)	BC
Oxypteryx helotella (Staudinger, 1859)	BC
Oxypteryx parahelotella (Nel, 1995)	BC
Oxypteryx graecatella (Šumpich & Skyva, 2012)	BC
**Gelechiinae Stainton, 1954**	
**Gelechiini Stainton, 1954**	
***Xystophora* Wocke, 1876**	
Xystophora carchariella (Zeller, 1839)	BC
Xystophora pulveratella (Herrich-Schäffer, 1854)	BC
***Athrips* Billberg, 1820**	
Athrips spiraeae (Staudinger, 1871)	BC
Athrips pruinosella (Lienig & Zeller, 1846)	BC
Athrips rancidella (Herrich-Schäffer, 1854)	BC
Athrips thymifoliella (Constant, 1893)	BC
Athrips amoenella (Frey, 1882)	BC
Athrips nigricostella (Duponchel, 1842)	BC
Athrips tetrapunctella (Thunberg, 1794)	BC
Athrips mouffetella (Linnaeus, 1758)	BC

Athrips asarinella (Chrétien, 1930)	
Athrips medjella (Chrétien, 1900)	
Athrips patockai (Povolný, 1979)	(BC)
Athrips polymaculella Park, 1991	BC
Athrips stepposa Bidzilya, 2005	BC
Athrips aquila Junnilainen, 2010	BC
Athrips bidzilyai Junnilainen, 2010	BC
Athrips fagoniae (Walsingham, 1904)	BC
***Neofriseria* Sattler, 1960**	
Neofriseria peliella (Treitschke, 1835)	BC
Neofriseria singula (Staudinger, 1876)	BC
Neofriseria pseudoterrella (Rebel, 1928)	(BC)
Neofriseria baungaardiella Huemer & Karsholt, 1999	BC
Neofriseria hitadoella Karsholt & Vives, 2014	BC
Neofriseria kuznetzovae Bidzilya, 2002	BC
Neofriseria caucasicella Sattler, 1960	
Neofriseria mongolinella Piskunov, 1987	BC
***Prolita* Leraut, 1993**	
Prolita sexpunctella (Fabricius, 1794)	BC
Prolita solutella (Zeller, 1839)	BC
***Sophronia* Hübner, 1825**	
Sophronia semicostella (Hübner, 1813)	BC
Sophronia gelidella Nordman, 1941	BC
Sophronia consanguinella Herrich-Schäffer, 1854	BC
Sophronia illustrella (Hübner, 1796)	BC
Sophronia grandii Hering, 1933	BC
Sophronia chilonella (Treitschke, 1833)	BC
Sophronia finitimella Rebel, 1905	(BC)
Sophronia acaudella Rebel, 1903	
Sophronia curonella Standfuss, 1884	
Sophronia humerella ([Denis & Schiffermüller], 1775)	BC
Sophronia sicariellus (Zeller, 1839)	BC
Sophronia santolinae Staudinger, 1863	BC
***Mirificarma* Gozmány, 1955**	

Mirificarma rhodoptera (Mann, 1866)	BC
Mirificarma minimella Huemer & Karsholt, 2001	BC
Mirificarma denotata Pitkin, 1984	BC
Mirificarma maculatella (Hübner, 1796)	BC
Mirificarma aflavella (Amsel, 1935)	BC
Mirificarma flavella (Duponchel, 1844)	BC
Mirificarma eburnella ([Denis & Schiffermüller], 1775)	BC
Mirificarma fasciata Pitkin, 1984	BC
Mirificarma lentiginosella (Zeller, 1839)	BC
Mirificarma pederskoui Huemer & Karsholt, 1999	BC
Mirificarma cytisella (Treitschke, 1833)	BC
Mirificarma monticolella (Rebel, 1931)	BC
Mirificarma interrupta (Curtis, 1827)	BC
Mirificarma burdonella (Rebel, 1930)	BC
Mirificarma cabezella (Chrétien, 1925)	BC
Mirificarma ulicinella (Staudinger, 1859)	BC
Mirificarma mulinella (Zeller, 1839)	BC
***Aroga* Busck, 1914**	
Aroga velocella (Zeller, 1839)	BC
Aroga flavicomella (Zeller, 1839)	BC
Aroga eatoni Corley & Goodey, 2014	
Aroga pascuicola (Staudinger, 1871)	BC
Aroga aristotelis (Millière, 1876)	BC
Aroga corsa Varenne & Nel, 2019	
Aroga temporariella Sattler, 1960	BC
Aroga balcanicola Huemer & Karsholt, 1999	BC
***Filatima* Busck, 1939**	
Filatima angustipennis Sattler, 1961	
Filatima pallipalpella (Snellen, 1884)	BC
Filatima spurcella (Duponchel, 1843)	BC
Filatima transsilvanella Kovács & Kovács, 2002	BC
Filatima algarbiella Corley, 2014	
Filatima tephritidella (Duponchel, 1844)	BC
Filatima textorella (Chrétien, 1908)	BC

Filatima djakovica Anikin & Piskunov, 1996	
Filatima incomptella (Herrich-Schäffer, 1854)	BC
Filatima ukrainica Piskunov, 1971	BC
Filatima zagulajevi Anikin & Piskunov, 1996	(BC)
***Chionodes* Hübner, 1825**	
Chionodes lugubrella (Fabricius, 1794)	BC
Chionodes tragicella (Heyden, 1865)	BC
Chionodes soella Huemer & Sattler, 1995	BC
Chionodes luctuella (Hübner, 1793)	BC
Chionodes aprilella Huemer & Sattler, 1995	BC
Chionodes violacea (Tengström, 1848)	BC
Chionodes mongolica Piskunov, 1979	
Chionodes holosericella (Herrich-Schäffer, 1854)	BC
Chionodes praeclarella (Herrich-Schäffer, 1854)	BC
Chionodes caucasicella Huemer & Sattler, 1995	
Chionodes nubilella (Zetterstedt, 1839)	BC
Chionodes continuella (Zeller, 1839)	BC
Chionodes perpetuella (Herrich-Schäffer, 1854)	BC
Chionodes apolectella (Walsingham, 1900)	BC
Chionodes distinctella (Zeller, 1839)	BC
Chionodes hayreddini Koçak, 1986	BC
Chionodes hinnella (Rebel, 1935)	
Chionodes bastuliella (Rebel, 1931)	BC
Chionodes electella (Zeller, 1839)	BC
Chionodes viduella (Fabricius, 1794)	BC
Chionodes nebulosella (Heinemann, 1870)	BC
Chionodes fumatella (Douglas, 1850)	BC
Chionodes ignorantella (Herrich-Schäffer, 1854)	BC
***Gelechia* Hübner, 1825**	
Gelechia rhombella ([Denis & Schiffermüller], 1775)	BC
Gelechia scotinella Herrich-Schäffer, 1854	BC
Gelechia senticetella (Staudinger, 1859)	BC
Gelechia obscuripennis (Frey, 1880)	BC
Gelechia sabinellus (Zeller, 1839)	BC

Gelechia atlanticella (Amsel, 1955)	BC
Gelechia nervosella (Zerny, 1927)	BC
Gelechia sororculella (Hübner, 1817)	BC
Gelechia jakovlevi Krulikovsky, 1905	BC
Gelechia muscosella Zeller, 1839	BC
Gelechia cuneatella Douglas, 1852	BC
Gelechia aspoecki Huemer, 1992	BC
Gelechia asinella (Hübner, 1796)	BC
Gelechia hippophaella (Schrank, 1802)	BC
Gelechia basipunctella Herrich-Schäffer, 1854	BC
Gelechia nigra (Haworth, 1828)	BC
Gelechia turpella ([Denis & Schiffermüller], 1775)	BC
Gelechia rhombelliformis Staudinger, 1871	BC
Gelechia sirotina Omelko, 1986	
Gelechia sestertiella Herrich-Schäffer, 1854	BC
Gelechia mediterranea Huemer, 1991	BC
Gelechia dujardini Huemer, 1991	BC
***Psoricoptera* Stainton, 1854**	
Psoricoptera speciosella Teich, 1893	BC
Psoricoptera gibbosella (Zeller, 1839)	BC
***Agnippe* Chambers, 1872**	
Agnippe echinuloides Bidzilya & Li, 2010	BC
Agnippe lunaki (Rebel, 1941)	
Agnippe pseudolella (Christoph, 1888)	BC
***Holcophora* Staudinger, 1871**	
Holcophora statices Staudinger, 1871	BC
Holcophora inderskella (Caradja, 1920)	
Holcophora obtusipalpis (Walsingham, 1905)	BC
**Gnorimoschemini Povolný, 1964**	
***Gnorimoschema* Busck, 1900**	
Gnorimochema soffneri (Riedl, 1965)	BC
Gnorimoschema herbichii (Nowicki, 1864)	BC
Gnorimoschema bodillum Karsholt & Nielsen, 1974	(BC)
Gnorimoschema nupponeni Huemer & Karsholt, 2010	BC

Gnorimoschema robustella (Staudinger, 1871)	BC
Gnorimoschema steueri Povolný, 1975	BC
Gnorimoschema epithymella (Staudinger, 1859)	BC
Gnorimoschema nordlandicolella (Strand, 1902)	BC
Gnorimoschema nilsi Huemer, 1996	BC
Gnorimoschema valesiella (Staudinger, 1877)	BC
Gnorimoschema streliciella (Herrich-Schäffer, 1854)	BC
Gnorimoschema hoefneri (Rebel, 1909)	BC
***Scrobipalpopsis* Povolný, 1967**	
Scrobipalpopsis petasitis (Pfaffenzeller, 1867)	BC
***Tecia* Povolný, 1973**	
Tecia solanivora (Povolný, 1973)	BC
***Scrobipalpa* Janse, 1951**	
Scrobipalpa aptatella (Walker, 1864)	BC
Scrobipalpa kasyi Povolný, 1968	BC
Scrobipalpa notata (Povolný, 2001)	BC
Scrobipalpa acuminatella (Sircom, 1850)	BC
Scrobipalpa skulei Huemer & Karsholt, 2010	BC
Scrobipalpa hungariae (Staudinger, 1871)	
Scrobipalpa adaptata (Povolný, 2001)	BC
Scrobipalpa brahmiella (Heyden, 1862)	BC
Scrobipalpa vasconiella (Rössler, 1877)	BC
Scrobipalpa dorsolutea Huemer & Karsholt, 2010	BC
Scrobipalpa amseli Povolný, 1966	BC
Scrobipalpa hyssopi Nel, 2003	BC
Scrobipalpa montanella (Chrétien, 1910)	BC
Scrobipalpa corleyi Huemer & Karsholt, 2010	BC
Scrobipalpa chrysanthemella (Hofmann, 1867)	BC
Scrobipalpa proclivella (Fuchs, 1886)	BC
Scrobipalpa frugifera Povolný, 1969	
Scrobipalpa oleksiyella Huemer & Karsholt, 2010	BC
Scrobipalpa smithi Povolný & Bradley, 1964	BC
Scrobipalpa occulta (Povolný, 2002)	BC
Scrobipalpa grisea Povolný, 1969	BC

Scrobipalpa usingeri Povolný, 1969	
Scrobipalpa clintoni Povolný, 1968	BC
Scrobipalpa reiprichi Povolný, 1984	BC
Scrobipalpa obsoletella (Fischer v. Röslerstamm, 1841)	BC
Scrobipalpa feralella (Zeller, 1872)	BC
Scrobipalpa halonella (Herrich-Schäffer, 1854)	BC
Scrobipalpa perinii (Klimesch, 1951)	BC
Scrobipalpa phagnalella (Constant, 1895)	BC
Scrobipalpa tokari Huemer & Karsholt, 2010	BC
Scrobipalpa karadaghi (Povolný, 2001)	BC
Scrobipalpa heimi Huemer & Karsholt, 2010	BC
Scrobipalpa acuta (Povolný, 2001)	
Scrobipalpa soffneri Povolný, 1964	BC
Scrobipalpa jariorum Huemer & Karsholt, 2010	BC
Scrobipalpa murinella (Duponchel, 1843)	BC
Scrobipalpa wiltshirei Povolný, 1966	BC
Scrobipalpa caucasica (Povolný, 2001)	
Scrobipalpa pauperella (Heinemann, 1870)	BC
Scrobipalpa spumata (Povolný, 2001)	BC
Scrobipalpa arenbergeri Povolný, 1973	BC
Scrobipalpa mercantourica Varenne & Nel, 2018	BC
Scrobipalpa nana Povolný, 1973	BC
Scrobipalpa heretica Povolný, 1973	
Scrobipalpa bigoti Povolný, 1973	BC
Scrobipalpa dorsoflava (Povolný, 1996)	BC
Scrobipalpa magnificella Povolný, 1967	BC
Scrobipalpa abstrusa Huemer & Karsholt, 2010	BC
Scrobipalpa superstes Povolný, 1977	BC
Scrobipalpa remota Povolný, 1972	
Scrobipalpa plesiopicta Povolný, 1969	BC
Scrobipalpa bradleyi Povolný, 1971	BC
Scrobipalpa selectella (Caradja, 1920)	
Scrobipalpa alterna (Falkovitsh & Bidzilya, 2006)	BC
Scrobipalpa lutea Povolný, 1977	BC

Scrobipalpa griseoflava Bidzilya & Budashkin, 2011	BC
Scrobipalpa niveifacies Povolný, 1977	BC
Scrobipalpa indignella (Staudinger, 1879)	BC
Scrobipalpa punctata (Povolný, 1996)	BC
Scrobipalpa lagodes (Meyrick, 1926)	
Scrobipalpa deluccae Povolný, 1966	
Scrobipalpa atriplicella (Fischer von Röslerstamm, 1841)	BC
Scrobipalpa suaedella (Richardson, 1893)	BC
Scrobipalpa solitaria Povolný, 1969	BC
Scrobipalpa dagmaris Povolný, 1987	
Scrobipalpa suasella (Constant, 1895)	BC
Scrobipalpa hendrikseni Huemer & Karsholt, 2010	BC
Scrobipalpa halimifolia Bidzilya & Budashkin, 2011	BC
Scrobipalpa traganella (Chrétien, 1915)	BC
Scrobipalpa bazae Povolný, 1977	BC
Scrobipalpa artemisiella (Treitschke, 1833)	BC
Scrobipalpa stangei (Hering, 1889)	BC
Scrobipalpa suaedivorella (Chrétien, 1915)	BC
Scrobipalpa bryophiloides Povolný, 1966	BC
Scrobipalpa algeriensis Povolný & Bradley, 1964	BC
Scrobipalpa deutschi Huemer & Karsholt, 2010	BC
Scrobipalpa disjectella (Staudinger, 1859)	BC
Scrobipalpa fontanensis Varenne & Nel, 2017	BC
Scrobipalpa mixta Huemer & Karsholt, 2010	
Scrobipalpa achtubica Anikin & Piskunov, 2018	
Scrobipalpa rebeli (Preissecker, 1914)	BC
Scrobipalpa gallicella (Constant, 1885)	BC
Scrobipalpa ustulatella (Staudinger, 1871)	BC
Scrobipalpa postulatella Huemer & Karsholt, 2010	BC
Scrobipalpa filia Povolný, 1969	
Scrobipalpa nitentella (Fuchs, 1902)	BC
Scrobipalpa costella (Humphreys & Westwood, 1845)	BC
Scrobipalpa hyoscyamella (Stainton, 1869)	BC
Scrobipalpa portosanctana (Stainton, 1859)	BC

Scrobipalpa vicaria (Meyrick, 1921)	BC
Scrobipalpa ocellatella (Boyd, 1858)	BC
Scrobipalpa pulchra Povolný, 1967	BC
Scrobipalpa gecko (Walsingham, 1911)	
Scrobipalpa hannemanni Povolný, 1966	BC
Scrobipalpa erichi Povolný, 1964	BC
Scrobipalpa divisella (Rebel, 1936)	BC
Scrobipalpa voltinella (Chrétien, 1898)	BC
Scrobipalpa corsicamontes Varenne & Nel, 2013	
Scrobipalpa suaedicola (Mabille, 1906)	BC
Scrobipalpa monochromella (Constant, 1895)	BC
Scrobipalpa samadensis (Pfaffenzeller, 1870)	BC
Scrobipalpa salinella (Zeller, 1847)	BC
Scrobipalpa spergulariella (Chrétien, 1910)	
Scrobipalpa salicorniae (Hering, 1889)	BC
Scrobipalpa halimioniella Huemer & Karsholt, 2010	BC
Scrobipalpa thymelaeae (Amsel, 1939)	BC
Scrobipalpa halymella (Millière, 1864)	BC
Scrobipalpa camphorosmella Nel, 1999	BC
Scrobipalpa stabilis Povolný, 1977	BC
Scrobipalpa instabilella (Douglas, 1846)	BC
Scrobipalpa peterseni (Povolný, 1965)	
Scrobipalpa ergasima (Meyrick, 1916)	BC
***Turcopalpa* Povolný, 1973**	
Turcopalpa glaseri Povolný, 1973	BC
***Scrobipalpula* Povolný, 1964**	
Scrobipalpula psilella (Herrich-Schäffer, 1854)	BC
Scrobipalpula ramosella (Müller-Rutz, 1934)	BC
Scrobipalpula seniorum Povolný, 2000	BC
Scrobipalpula diffluella (Frey, 1870)	BC
Scrobipalpula tussilaginis (Stainton, 1867)	BC
***Phthorimaea* Meyrick, 1902**	
Phthorimaea operculella (Zeller, 1873)	BC
***Tuta* Kieffer & Jørgensen, 1910**	

Tuta absoluta (Meyrick, 1917)	BC
***Keiferia* Busck, 1939**	
Keiferia lycopersicella (Walsingham, 1897)	
***Ephysteris* Meyrick, 1908**	
Ephysteris promptella (Staudinger, 1859)	BC
Ephysteris tenuisaccus Nupponen, 2010	
Ephysteris deserticolella (Staudinger, 1871)	BC
Ephysteris insulella (Heinemann, 1870)	BC
Ephysteris brachyptera Karsholt & Sattler, 1998	
Ephysteris diminutella (Zeller, 1839)	BC
Ephysteris inustella (Zeller, 1847)	BC
Ephysteris olympica Povolný, 1968	BC
Ephysteris iberica Povolný, 1977	BC
***Ochrodia* Povolný, 1966**	
Ochrodia subdiminutella (Stainton, 1867)	BC
***Vladimirea* Povolný, 1967**	
Vladimirea glebicolorella (Erschoff, 1874)	
***Microlechia* Turati, 1924**	
Microlechia rhamnifoliae (Amsel & Hering, 1931)	BC
Microlechia chretieni Turati, 1924	BC
Microlechia klimeschi (Povolný, 1972)	BC
Microlechia karsholti (Nupponen, 2010)	BC
***Cosmardia* Povolný, 1965**	
Cosmardia moritzella (Treitschke, 1835)	BC
***Lutilabria* Povolný, 1965**	
Lutilabria lutilabrella (Mann, 1857)	BC
Lutilabria volgensis Anikin & Piskunov, 1996	BC
Lutilabria prolata Junnilainen & Nupponen, 2010	BC
***Klimeschiopsis* Povolný, 1967**	
Klimeschiopsis kiningerella (Duponchel, 1843)	BC
Klimeschiopsis discontinuella (Rebel, 1899)	BC
Klimeschiopsis maritimaealpina Nel & Varenne, 2011	BC
Klimeschiopsis terroris (Hartig, 1938)	BC
***Caryocolum* Gregor & Povolný, 1954**	

Caryocolum fischerella (Treitschke, 1833)	BC
Caryocolum tischeriella (Zeller, 1839)	BC
Caryocolum alsinella (Zeller, 1868)	BC
Caryocolum viscariella (Stainton, 1855)	BC
Caryocolum albifaciella (Heinemann, 1870)	BC
Caryocolum vicinella (Douglas, 1851)	BC
Caryocolum bosalella (Rebel, 1936)	BC
Caryocolum sciurella (Walsingham, 1908)	BC
Caryocolum amaurella (Hering, 1924)	BC
Caryocolum crypticum Huemer, Karsholt & Mutanen, 2014	BC
Caryocolum tredosella Nel & Requena, 2017	
Caryocolum oculatella (Thomann, 1930)	BC
Caryocolum leucofasciatum Huemer, 1989	BC
Caryocolum petryi (Hofmann, 1899)	BC
Caryocolum baischi Huemer & Karsholt, 2010	BC
Caryocolum repentis Huemer & Luquet, 1992	BC
Caryocolum siculum Bella, 2008	BC
Caryocolum inflativorella (Klimesch, 1938)	
Caryocolum saginella (Zeller, 1868)	BC
Caryocolum cauligenella (Schmid, 1863)	BC
Caryocolum trauniella (Zeller, 1868)	BC
Caryocolum peregrinella (Herrich-Schäffer, 1854)	BC
Caryocolum delphinatella (Constant, 1890)	BC
Caryocolum provinciella (Stainton, 1869)	BC
Caryocolum mucronatella (Chrétien, 1900)	BC
Caryocolum leucomelanella (Zeller, 1839)	BC
Caryocolum mazeli Huemer & Nel, 2005	BC
Caryocolum leucothoracellum (Klimesch, 1953)	BC
Caryocolum schleichi (Christoph, 1872)	BC
Caryocolum arenariella (Benander, 1937)	BC
Caryocolum marmorea (Haworth, 1828)	BC
Caryocolum pullatella (Tengström, 1848)	BC
Caryocolum stramentella (Rebel, 1935)	BC
Caryocolum hispanicum Huemer, 1988	

Caryocolum confluens Huemer, 1988	BC
Caryocolum srnkai Huemer & Karsholt, 2011	BC
Caryocolum gallagenellum Huemer, 1989	BC
Caryocolum fraternella (Douglas, 1851)	BC
Caryocolum klosi (Rebel, 1917)	BC
Caryocolum interalbicella (Herrich-Schäffer, 1854)	BC
Caryocolum laceratella (Zeller, 1868)	BC
Caryocolum dauphini Grange & Nel, 2012	BC
Caryocolum blandella (Douglas, 1852)	BC
Caryocolum blandelloides Karsholt, 1981	BC
Caryocolum horoscopa (Meyrick, 1926)	BC
Caryocolum jaspidella (Chrétien, 1908)	BC
Caryocolum proxima (Haworth, 1828)	BC
Caryocolum blandulella (Tutt, 1887)	BC
Caryocolum arenbergeri Huemer, 1989	BC
Caryocolum tricolorella (Haworth, 1812)	BC
Caryocolum fibigerium Huemer, 1988	BC
Caryocolum junctella (Douglas, 1851)	BC
Caryocolum cassella (Walker, 1864)	BC
Caryocolum moehringiae (Klimesch, 1954)	BC
Caryocolum petrophila (Preissecker, 1914)	BC
Caryocolum huebneri (Haworth, 1828)	BC
Caryocolum kroesmanniella (Herrich-Schäffer, 1854)	BC
***Tila* Povolný, 1965**	
Tila capsophilella (Chrétien, 1900)	BC
***Pogochaetia* Staudinger, 1879**	
Pogochaetia solitaria Staudinger, 1879	BC
***Agonochaetia* Povolný, 1967**	
Agonochaetia terrestrella (Zeller, 1872)	BC
Agonochaetia intermedia Sattler, 1968	BC
Agonochaetia quartana Povolný, 1990	
***Canarischema* Karsholt, 2017**	
Canarischema fuerteventura Karsholt, 2017	BC
***Sattleria* Povolný, 1965**	

Sattleria melaleucella (Constant, 1865)	BC
Sattleria arcuata Pitkin & Sattler, 1991	BC
Sattleria pyrenaica (Petry, 1904)	BC
Sattleria taurandi Nel & Varenne, 2019	
Sattleria karsholti Huemer & Hebert, 2011	BC
Sattleria cottiella Huemer & Hebert, 2011	BC
Sattleria marguareisi Huemer & Sattler, 1992	BC
Sattleria izoardi Huemer & Sattler, 1992	BC
Sattleria graiaeella Huemer & Hebert, 2011	BC
Sattleria dolomitica Huemer, 2014	
Sattleria basistrigella Huemer, 1997	BC
Sattleria triglavica Povolný, 1987	BC
Sattleria dinarica Huemer, 2014	BC
Sattleria haemusi Huemer, 2014	BC
Sattleria dzieduszyckii (Nowicki, 1864)	BC
Sattleria angustispina Pitkin & Sattler, 1991	
Sattleria breviramus Pitkin & Sattler, 1991	BC
Sattleria sophiae Timossi, 2014	BC
Sattleria styriaca Pitkin & Sattler, 1991	BC
**Litini Bruand d'Uzelle 1859**	
***Schneidereria* Weber, 1957**	
Schneidereria pistaciella Weber, 1957	BC
***Teleiodes* Sattler, 1960**	
Teleiodes vulgella ([Denis & Schiffermüller], 1775)	BC
Teleiodes italica Huemer, 1992	BC
Teleiodes brevivalva Huemer, 1992	BC
Teleiodes wagae (Nowicki, 1860)	BC
Teleiodes saltuum (Zeller, 1878)	BC
Teleiodes kaitilai Junnilainen, 2010	BC
Teleiodes luculella (Hübner, 1813)	BC
Teleiodes flavimaculella (Herrich-Schäffer, 1854)	BC
Teleiodes albidorsella Huemer & Karsholt, 1999	
Teleiodes albiluculella Huemer & Karsholt, 2001	BC
***Neotelphusa* Janse, 1958**	

Neotelphusa sequax (Haworth, 1828)	BC
Neotelphusa huemeri (Nel, 1998)	BC
Neotelphusa traugotti (Huemer & Karsholt, 2001)	(BC)
Neotelphusa cisti (Stainton, 1869)	BC
***Carpatolechia* Capuse, 1964**	
Carpatolechia decorella (Haworth, 1812)	BC
Carpatolechia aenigma (Sattler, 1983)	BC
Carpatolechia fugitivella (Zeller, 1839)	BC
Carpatolechia fugacella (Zeller, 1839)	BC
Carpatolechia minor (Kasy, 1978)	
Carpatolechia filipjevi (Lvovsky & Piskunov, 1993)	BC
Carpatolechia alburnella (Zeller, 1839)	BC
Carpatolechia notatella (Hübner, 1813)	BC
Carpatolechia proximella (Hübner, 1796)	BC
Carpatolechia intermediella Huemer & Karsholt, 1999	
Carpatolechia epomidella (Tengström, 1869)	BC
***Pseudotelphusa* Janse, 1958**	
Pseudotelphusa scalella (Scopoli, 1763)	BC
Pseudotelphusa istrella (Mann, 1866)	BC
Pseudotelphusa occidentella Huemer & Karsholt, 1999	BC
Pseudotelphusa paripunctella (Thunberg, 1794)	BC
Pseudotelphusa tessella (Linnaeus, 1758)	BC
***Istrianis* Meyrick, 1918**	
Istrianis myricariella (Frey, 1870)	BC
Istrianis arenicolella (Caradja, 1920)	BC
Istrianis pseudomyricariella Bidzilya & Karsholt, 2015	
Istrianis nilssoni Bidzilya & Karsholt, 2015	BC
Istrianis brucinella (Mann, 1872)	
Istrianis femoralis (Staudinger, 1876)	BC
Istrianis piskunovi Bidzilya & Karsholt, 2015	
***Streyella* Janse, 1958**	
Streyella canariensis (Walsingham, 1908)	BC
Streyella anguinella (Herrich-Schäffer, 1861)	BC
***Teleiopsis* Sattler, 1960**	

Teleiopsis terebinthinella (Herrich-Schäffer, 1856)	BC
Teleiopsis latisacculus Pitkin, 1988	BC
Teleiopsis diffinis (Haworth, 1828)	BC
Teleiopsis lunariella (Walsingham, 1908)	BC
Teleiopsis bagriotella (Duponchel, 1840)	BC
Teleiopsis laetitiae Schmid, 2011	BC
Teleiopsis lindae Schmid, 2011	BC
Teleiopsis albifemorella (Hofmann, 1867)	BC
Teleiopsis paulheberti Huemer & Mutanen, 2012	BC
Teleiopsis rosalbella (Fologne, 1862)	BC
***Xenolechia* Meyrick, 1895**	
Xenolechia aethiops (Humphreys & Westwood, 1845)	BC
Xenolechia lindae Huemer & Karsholt, 1999	BC
Xenolechia pseudovulgella Huemer & Karsholt, 1999	BC
***Altenia* Sattler, 1960**	
Altenia perspersella (Wocke, 1862)	BC
Altenia scriptella (Hübner, 1796)	BC
Altenia elsneriella Huemer & Karsholt, 1999	BC
Altenia mersinella (Staudinger, 1879)	BC
Altenia wagneriella (Rebel, 1926)	BC
Altenia modesta (Danilevsky, 1955)	BC
***Recurvaria* Haworth, 2828**	
Recurvaria nanella ([Denis & Schiffermüller], 1775)	BC
Recurvaria leucatella (Clerck, 1759)	BC
Recurvaria thomeriella (Chrétien, 1901)	BC
Recurvaria costimaculella Huemer & Karsholt, 2001	
***Coleotechnites* Chambers, 1880**	
Coleotechnites piceaella (Kearfott, 1903)	BC
***Exoteleia* Wallengren, 1881**	
Exoteleia dodecella (Linnaeus, 1758)	BC
Exoteleia succinctella (Zeller, 1872)	BC
***Stenolechia* Meyrick, 1894**	
Stenolechia gemmella (Linnaeus, 1758)	BC
***Parastenolechia* Kanazawa, 1985**	

Parastenolechia nigrinotella (Zeller, 1847)	BC
***Stenolechiodes* Elsner, 1996**	
Stenolechiodes pseudogemmellus Elsner, 1996	BC
Stenolechiodes macrolepiellus Huemer & Karsholt, 1999	BC
***Parachronistis* Meyrick, 1925**	
Parachronistis albiceps (Zeller, 1839)	BC
***Schistophila* Chrétien, 1899**	
Schistophila laurocistella Chrétien, 1899	BC
**unplaced genus**	
"*Telphusa*" *cistiflorella* (Constant, 1890)	BC
