# Supplementary material for: DNA barcode library for European Gelechiidae (Lepidoptera) suggests greatly underestimated species diversity
Source: Zookeys. 2020 Mar 24;921:141–57. doi: 10.3897/zookeys.921.49199 (PMC7109146; doi:10.3897/zookeys.921.49199)
Supplement: Supplementary material 2 [file zookeys-921-141-s002.pdf]

Supplementary material 2

NJ trees 1–53

A-V = different intraspecific clusters (BINs); n= number of sequenced specimens; country abbreviations according to ISO 3166-1 alpha-3 (https://en.wikipedia.org/wiki/ISO_3166-1_alpha-39)

NJ tree 1
Acompsia tripunctella A (BOLD:AAB3415) n=26: AUT, FRA, GER, ITA, SVN
Acompsia tripunctella B (BOLD:AAB3417) n=3: FRA, ITA
Acompsia tripunctella C (BOLD:AAB3414) n=15: AUT, GER, ITA
Acompsia tripunctella D (BOLD:AAB3416) n=1: CHE
Acompsia tripunctella D (BOLD:AAB3416)n=5: ITA
Acompsia tripunctella E (BOLD:ADL1239) n=1: GER
Acompsia dimorpha (BOLD:AAJ5944) n=1: FRA
Acompsia ponomarenkoae (BOLD:ABA5285) n=3: MKD
Acompsia antirrhinella (BOLD:AAJ5937) n=2: ESP, FRA
Acompsia pyrenaella A (BOLD:AAJ5937) n=1: ESP
Acompsia tripunctella F (BOLD:AAJ5937) n=3: AUT, ITA
Acompsia maculosella A (BOLD:AAD5140) n=3: AUT
Acompsia pyrenaella B (BOLD:AAJ3058) n=4: AND, ESP, FRA
Acompsia pyrenaella C (BOLD:ACA9634) n=3: ESP
Acompsia maculosella B (BOLD:AAD5139) n=9: AUT, GER
Acompsia delmastroella (BOLD:AAJ3063) n=3: FRA, ITA
Acompsia baldizzonei (BOLD:ADR9697) n=3: AUT, ITA
Acompsia subpunctella (BOLD:AAL7813) n=3: FIN
Acompsia cinerella (BOLD:AAD0078) n=36: AND, AUT, FIN, FRA, GER, ITA, MKD, NOR
Acompsia schmidtiellus (BOLD:AAK0920) n=9: GER, ITA

NJ tree 2
Psoricoptera gibbosella (BOLD:AAD0608) n=31: AUT, CHE, DNK, FIN, GER, ITA, NOR
Psoricoptera speciosella (BOLD:ABY7503) n=16: AUT, FIN, GER, NOR
Holcophora statices (BOLD:ADM2267) n=1: HUN
Agnippe echinuloides (BOLD:ACB0598) n=1: RUS
Agnippe pseudolella (BOLD:AAW4777) n=2: KGZ

NJ tree 3
Agonochaetia terrestrella A (BOLD:ADR6815) n=3: CHE
Agonochaetia terrestrella B (BOLD:ABV4431) n=1: ROU
Agonochaetia intermedia (BOLD:ABV4430) n=4: AUT, RUS
Tila capsophilella (BOLD:ACL7124) n=2: AUT
Pogochaetia solitaria (BOLD:ACA9790) n=5: AUT, ITA
Canarischema fuerteventura (BOLD:ACZ6649) n=2: ESP

NJ tree 4
Altenia scriptella A (BOLD:AAF3288) n=12: AUT, GER, ITA
Altenia scriptella B (BOLD:ACB4200) n=5: GER, HRV, MKD, NOR

Altenia wagneriella (BOLD:ADL0602) n=1: MKD
Altenia modesta (BOLD:ADK9159) n=2: ITA
Altenia mersinella (missing) n=1: CYP
Altenia elsneriella (BOLD:ADL8920) n=3: GRE, HRV, MKD
Altenia perspersella (BOLD:AAE2251) n=4: FIN, NOR

NJ tree 5
Anacampsis blattariella A (BOLD:AAC9810) n=28: AUT, FIN, GER, ITA, NOR
Anacampsis blattariella B (BOLD:AAD3256) n=3: AUT
Anacampsis populella A (BOLD:AAD3256) n=1: FIN
Anacampsis populella A (BOLD:AAD3256) n=21: AUT, FIN, GER, ITA, NOR
Anacampsis scintillella A (BOLD:ACF5668) n=2: ESP
Anacampsis scintillella B (BOLD:AAP3199) n=16: AUT, GER, ITA
Anacampsis temerella (BOLD:AAE5852) n=4: FIN, NOR
Anacampsis obscurella A (BOLD:ACX8131) n=3: HRV, ITA
Anacampsis obscurella B (BOLD:AAP3198) n=6: AUT, GER
Anacampsis timidella (BOLD:AAU3897) n=7: AUT, GER, ITA
Anacampsis trifoliella (BOLD:ADS0030) n=1: ITA
Anacampsis fuscella (BOLD:AAL9303)n=4: FIN, GRE

NJ tree 6
Anarsia innoxiella (BOLD:ABZ2446) n=23: AUT, CZE, FIN, GER, ITA
Anarsia lineatella (BOLD:AAD7849) n=8: AUT, DNK, ESP, GER, ITA
Anarsia sp. 1 (BOLD:ADE9567) n=1: GRE
Anarsia eleagnella (BOLD:ADL9428) n=1: HUN
Anarsia acaciae (BOLD:ADA2395) n=1: MAR
Anarsia leberonella (BOLD:AAO2944) n=6: FRA, GRE, ITA
Anarsia sp. 2 (BOLD:ADE9710) n=1: CYP
Anarsia sibirica (BOLD:AAY5340) n=1: RUS
Anarsia spartiella (BOLD:ABA2603) n=4: GER, MKD
Anarsia bilbainella A (BOLD:ADU3193) n=1: ESP
Anarsia bilbainella B (BOLD:AAJ2343) n=8: FRA, ITA

NJ tree 7
Dichomeris limosellus (BOLD:AAV6621) n=10: AUT, FIN, GER, ROU
Dichomeris nitiellus (BOLD:AAV6622) n=2: ITA
Dichomeris barbella (BOLD:ADC7243) n=1: ROU
Dichomeris ustalella (BOLD:AAM5583) n=10: AUT, BGR, DNK, GER, ITA
Dichomeris derasella (BOLD:AAE7499) n=22: AUT, FIN, GER, ITA, RUS
Dichomeris acuminatus (BOLD:AAB6409) n=4: GRE, ITA
Dichomeris neatodes (BOLD:ADL5756) n=4: CYP, GRE, HRV
Dichomeris limbipunctellus (BOLD:ADL7272) n=2: ITA
Dichomeris helianthemi (BOLD:AAV6610) n=3: ESP
Dichomeris castellana (BOLD:ADM0361) n=1: ESP
Dichomeris sp. 1 (BOLD:ADI2574) n=1: ESP
Dichomeris juniperella A (BOLD:AAF5665) n=9: AUT, FIN, GER, ITA, NOR
Dichomeris juniperella B (BOLD:AAU1435) n=3: ITA
Dichomeris marginella (BOLD:AAC4900) n=8: DNK, ESP, GER
Dichomeris latipennella (BOLD:AAF1370) n=25: AUT, FIN, GER, ITA, NOR
Dichomeris alacella (BOLD:AAF1347) n=31 : AUT, ESP, FIN, FRA, GER, ITA, NOR
Dichomeris rasilella A (BOLD:AAI8300) n=3: AUT, FIN

Dichomeris rasilella B (BOLD:AAQ3487) n=1: RUS
Anasphaltis renigerellus (BOLD:AAV9730) n=4: AUT, ITA

NJ tree 8
Apatetris sp.1 (BOLD:AAV7596) n=6: ESP
Coloptilia conchylidella (missing) n=1: TUR
Catatinagma trivittellum A (BOLD:ADL7614) n=1: MKD
Catatinagma trivittellum B (BOLD:ADM2953) n=1: FRA
Catatinagma kraterella (BOLD:ACB0721) n=1: RUS
Apatetris mediterranella A (BOLD:ADM1409) n=1: GRE
Apatetris mediterranella B (BOLD:ABA4709) n=3: ITA
Apatetris agenjoi (BOLD:ADM1885) n=1: ESP
Apatetris sp.2 (BOLD:AAV7596) n=7: BGR, HRV, ITA, MKD
Dactylotula kinkerella A (BOLD:ACH7599) n=1: GER
Dactylotula kinkerella B (BOLD:ADL7709) n=4: CZE, ESP, FRA

NJ tree 9
Apodia bifractella (BOLD:AAF8159) n=19: AUT, BGR, ESP, GER, ITA
Apodia martinii (BOLD:AAF8160) n=7: AUT, FIN, GER, ITA
Argolamprotes micella (BOLD:AAD2506) n=17: AUT, FIN, GER, ITA, NOR
Pragmatodes sp.1 (BOLD:ACF6594) n=4: BGR
Pragmatodes albagonella (BOLD:ADM0124) n=3: AUT, FRA
Pragmatodes cyrneogonella (BOLD:ACW2356) n=3: FRA, ITA
Pragmatodes melagonella (BOLD:ACE2136) n=1: FRA
Pragmatodes parvulata (BOLD:ACB9409) n=13: AUT, GER, ITA, ROU

NJ tree 10
Iwaruna biguttella (BOLD:AAU3602) n=10: ESP, FRA, GRE, HRV
Iwaruna klimeschi (BOLD:AAU3602) n=5: CZE, ITA, MKD, SVN
Iwaruna robineaui (BOLD:AAU3602) n=1 : FRA
Aproaerema albipalpella (BOLD:ACB8811) n=1: FRA
Aproaerema cincticulella (BOLD:ACB8811) n=9: GER
Aproaerema sangiella (BOLD:AAE8758) n=30: AUT, FIN, FRA, GER, ITA, MKD, NOR
Aproaerema buvati (BOLD:ABY3626) n=3: ESP, FRA
Aproaerema cinctella A (BOLD:AAD7223) n=18: AUT, FIN, NOR
Aproaererna cinctella B (BOLD:ADY6734) n=20: AUT, GER, ITA, MKD
Aproaererna cinctelloides (BOLD:ABX8391) n=7: FRA, GER, GRE, MKD
Aproaerema coronillella A (BOLD:ACF4181) n=46: AUT, GER, ITA
Aproaerema coronillella B (BOLD:AAE2580) n=35: AUT, GER, HRV, ITA
Aproaerema wormiella (BOLD:AAK1466) n=19: AUT, FIN, FRA, GER, ROU
Aproaerema larseniella (BOLD:AAK1468) n=8: AUT, GER
Aproaerema sp.1 (BOLD:ADL8444) n=3: FRA, GRE, HRV
Aproaerema taeniolella (BOLD:AAE8756) n=35: AUT, ESP, FIN, FRA, GER, GRE, ITA, NOR
Aproaerema patruella (BOLD:AAE2579) n=38: AUT, FRA, GER, ITA
Aproaerema incognitana (missing) n=3: ITA
Aproaerema thaumalea (BOLD:ADO5854) n=1: MOR
Aproaerema vinella (BOLD:ABV9469) n=14: AUT, GER
Aproaerema steppicolella (BOLD:ADR4159) n=1: RUS
Aproaerema karvoneni A (BOLD:ACE7715) n=2: CHE, FRA
Aproaerema karvoneni B (BOLD:ABZ6645) n=5: FIN
Aproaerema karvoneni C (BOLD:AAE8761) n=9: FIN, NOR

Aproaerema ochrofasciella (BOLD:AAZ7074) n=2: AUT, ITA
Aproaerema sp.2 (BOLD:ADL9068) n=1: MNE
Aproaerema sp.3 (BOLD:AAT9258) n=1: ESP
Aproaerema semicostella (BOLD:AAW5087) n=1: RUS
Aproaerema sp.4 (BOLD:ADL9069) n=2: GRE, ITA
Aproaerema azosterella (BOLD:ADH5263) n=2: FRA, GRE
Aproaerema genistae (BOLD:ADL6169) n=2: ESP
Aproaerema sp.5 (BOLD:ADG7311) n=1 : ESP
Aproaerema sp.6 (BOLD:ACF7323) n=1: ROU
Aproaerema polychromella (BOLD:ABV2027) n=1: ITA
Aproaerema captivella (BOLD:AAV8424) n=2: ITA
Aproaerema suecicella A (BOLD:AAZ7929) n=2: AUT, DNK
Aproaerema suecicella B (BOLD:AAU4349) n=7: ESP, FRA
Aproaerema albifrontella (BOLD:AAH3893) n=14: AUT, GER, ITA
Aproaerema lerauti (BOLD:AAV9841) n=1: ESP
Aproaerema anthyllidella A (BOLD:AAD2266) n=76: AUT, CHE, DNK, ESP, FIN, FRA, GER, GRE, HRV, ITA, NOR, PRT
Aproaerema anthyllidella B (BOLD:ADR9287) n=1: ESP
Aproaerema anthyllidella C (BOLD:AAD2267) n=34: AUT, DNK, FlN, FRA, GER, NOR, PRT
Aproaerema mercedella (BOLD:ADM9690) n=1: ESP

NJ tree 11
Aristotelia heliacella (BOLD:AAE6658) n=16: AUT, CHE, FIN, FRA, ITA
Aristotelia decurtella A (BOLD:AAJ9936) n=5: AUT, FRA, ITA
Aristotelia decurtella B (BOLD:ABA3199) n=6: HUN, ITA, MKD, ROU
Aristotelia sp.1 (BOLD:ABV2430) n=2: ESP
Aristotelia pancaliella (missing) n=1: TUR
Aristotelia baltica (BOLD:AAZ7113) n=1: LAT
Aristotelia sp.2 (BOLD:AAV7599) n=2: ESP
Aristotelia sp.3 (BOLD:ACK0360) n=4: BGR, ITA
Aristotelia sp.4 (BOLD:ADK9648) n=1: SVN
Aristotelia subericinella (BOLD:ADR8914) n=2: AUT
Aristotelia sp.5 (BOLD:ADC8189) n=1: ROU
Aristotelia billii (BOLD:AAW5816) n=3: BGR, FRA, GRE
Aristotelia sp. 6 (BOLD:ACC2990) n=3: GRE, ITA
Aristotelia sp.7 (BOLD:AAU2122) n=5: ESP
Aristotelia sp.8 (BOLD:ADY0927) n=1: ESP
Aristotelia decoratella (BOLD:AAV9850) n=1: ESP
Aristotelia sp.9 (BOLD:ADM4599) n=2: ITA
Aristotelia ericinella (BOLD:AAD9272) n=11: AUT, FIN, GER, NOR
Aristotelia calastomella (BOLD:ACB0626) n=4: HUN, RUS
Aristotelia subdecurtella A (BOLD:AAP7516) n=2: FIN
Aristotelia subdecurtella B (BOLD:AAP7515) n=8: AUT, FIN, GER, ITA
Aristotelia frankeniae (BOLD:AAV9851) n=2: ESP
Aristotelia montarcella (BOLD:ADN3894) n=1: ESP
Aristotelia sp.10 (BOLD:ADL8769) n=1: ESP
Aristotelia sp.11 (BOLD:ADL8520) n=2: HRV, ITA
Aristotelia sp.12 (BOLD:ADL9120) n=1: ITA
Aristotelia mirabilis (BOLD:ACB0949) n=1: RUS
Aristotelia staticella (BOLD:ADS0089) n=1: FRA
Aristotelia confusella (BOLD:AAJ1682) n=3: BGR, UKR

Aristotelia brizella (BOLD:AAJ1682) n=11: FIN, GER, GRE, ITA, MKD, NOR

NJ tree 12
Aroga velocella A (BOLD:AAC7813) n=19: AUT, BGR, FIN, GER, ITA, NOR
Aroga velocella B (BOLD:ACJ5010) n=3: GER
Aroga velocella C (BOLD:ACJ5279) n=1: GER
Aroga pascuicola (BOLD:ADL6412) n=2: FRA, ITA
Aroga aristotelis (BOLD:AAK0049) n=1: FRA
Aroga temporariella (BOLD:ADL7754) n=2: ESP, FRA
Aroga balcanicola (BOLD:AAZ9708) n=1: GRE
Aroga flavicomella A (BOLD:ADF8457) n=2: AUT, ITA
Aroga flavicomella B (BOLD:ADF8456) n=1: GER
Aroga flavicomella B (BOLD:ADF8456) n=1: AUT
Aroga flavicomella B (BOLD:ADF8456) n=1: GER
Aroga flavicomella C (BOLD:ADF8328) n=2: AUT
Aroga flavicomella D (BOLD:AAV9856) n=2: ITA

NJ tree 13
Athrips pruinosella (BOLD:AAD2577) n=29: AUT, FIN, FRA, GER, NOR, RUS
Athrips spiraeae (BOLD:AAD2577) n=3: RUS
Athrips bidzilyai (BOLD:ACB0731) n=2: RUS
Athrips mouffetella (BOLD:AAC9993) n=23: AUT, FIN, GER, ITA, NOR
Athrips patockai (BOLD:ACG8916) n=1: SVK
Athrips polymaculella (BOLD:ACB0649) n=3: RUS
Athrips aquila (BOLD:ADO3593) n=1: RUS
Athrips rancidella A (BOLD:AAD2618) n=15: AUT, ESP, GER
Athrips rancidella B (BOLD:ADE9737) n=1: GRE
Athrips tetrapunctella (BOLD:AAF5922) n=3: FIN
Athrips stepposa (BOLD:ADO4433) n=1: RUS
Athrips nigricostella (BOLD:AAY7867) n=2: AUT, HUN
Athrips amoenella A (BOLD:ACR3436) n=1: ITA
Athrips amoenella B (BOLD:ADK9309) n=3: AUT
Athrips amoenella C (BOLD:AAU5392) n=2: FIN
Athrips amoenella D (BOLD:ACF0469) n=1: AUT
Athrips amoenella E (BOLD:ABW2239) n=4: ITA, NOR
Athrips thymifoliella (BOLD:ADO4434) n=1 : ESP
Athrips fagoniae (BOLD:ADM8377) n=1: ESP
Xystophora carchariella (BOLD:ADM1649) n=1: SVK
Xystophora pulveratella (BOLD:AAE4677) n=10: FIN, HUN, ITA, NOR

NJ tree 14
Gladiovalva rumicivorella (BOLD:AAY0789) n=2: ITA
Gladiovalva badidorsella (BOLD:ADL9737) n=1: ESP
Gladiovalva aizpuruai (BOLD:ADL9920) n=1: RUS
Ornativalva mixolitha (BOLD:ADR2046) n=1: MAR
Ornativalva ornatella (BOLD:ADM9491) n=1: KAZ
Ornativalva plutelliformis (BOLD:ABW9166) n=3: BGR, MKD
Ornativalva heluanensis (BOLD:ACE0451) n=3: GRE
Ornativalva tamaricella (BOLD:ACE0451) n=3: ESP, HRV, ITA
Ornativalva antipyramis (missing) n=1: ESP
Atremaea lonchoptera (BOLD:ACR3301) n=2: ITA

NJ tree 15
Helcystogramma rufescens (BOLD:AAC1177) n=29: AUT, BGR, FIN, GER, ITA, NOR
Helcystogramma albinervis (BOLD:ACC3445) n=5: AUT, HUN
Helcystogramma flavescens (BOLD:ADR8962) n=1: RUS
Helcystogramma lineolella (BOLD:AAK4655) n=7: AUT, FIN, GER, NOR
Helcystogramma lutatella (BOLD:AAI6980) n=27: AUT, ESP, FIN, FRA, GER, ITA, NOR, ROU
Helcystogramma trianullela (BOLD:ACX0424) n=6: AUT, GER, ITA
Helcystogramma arulensis (BOLD:AAU3156) n=10: AUT, ITA
Helcystogramma lamprostoma (BOLD:AAV6609) n=3: ESP, GRE
Brachmia procursella (BOLD:ADK8841) n=1: AUT
Brachmia inornatella (BOLD:AAI8301) n=10: AUT, FIN, GER, SWE
Brachmia sp.1 (BOLD:ADM5065) n=1: GRE
Brachmia dimidiella A (BOLD:AAU2903) n=1: ITA
Brachmia dimidiella B (BOLD:ACK6536) n=2: GER
Brachmia dimidiella C (BOLD:AAF6101) n=5: AUT, FIN, MKD
Brachmia blandella (BOLD:AAD2457) n=26: AUT, FIN, GER, ITA
Pseudosophronia cosmella (BOLD:ADR7943) n=1: ESP
Pseudosophronia exustellus (BOLD:AAV2780) n=3: ESP

NJ tree 16
Bryotropha affinis (BOLD:AAC3323) n=50: AUT, DNK, FIN, GER, ITA, NOR, SWE
Bryotropha umbrosella A (BOLD:AAC3323) n=8: FIN, SWE
Bryotropha umbrosella B (BOLD:AAJ8980) n=11: FIN, NOR
Bryotropha similis (BOLD:AAA2297) n=58: AUT, FIN, GER, ITA, NOR
Bryotropha plantariella (BOLD:AAA5914) n=4: FIN, NOR
Bryotropha basaltinella (BOLD:AAH5446) n=14: ESP, GER, NOR
Bryotropha hulli A (BOLD:ADM2092) n=4: GRE
Bryotropha hulli B (BOLD:ADM5964) n=2: GRE
Bryotropha sutteri (BOLD:ADL8606) n=2: ITA, MKD
Bryotropha hendrikseni (BOLD:ADF1189) n=2: BGR
Bryotropha plebejella (BOLD:ADF2534) n=3: ESP, GRE
Bryotropha gallurella (BOLD:ADL8617) n=3: ESP, FRA, ITA
Bryotropha pallorella (BOLD:ADL7409) n=2: ESP
Bryotropha dryadella (BOLD:AAW7301) n=2: ESP, ITA
Bryotropha senectella (BOLD:AAD3661) n=63: AUT, FIN, GER, ITA, NOR
Bryotropha figulella (BOLD:ADL9820) n=5: ITA
Bryotropha boreella (BOLD:AAE0932) n=9: AUT, FIN, GER
Bryotropha galbanella (BOLD:AAE0931) n=25: AUT, FIN, GER, ITA, NOR
Bryotropha vondermuhlli (BOLD:ADI8418) n=2: ESP
Bryotropha domestica (BOLD:AAE8511) n=4: FRA, GER, GRE
Bryotropha arabica (BOLD:ADL7143) n=2: BGR, GRE
Bryotropha purpurella (BOLD:AAI8516) n=4: FIN, NOR
Bryotropha aliterrella (missing) n=1: ESP
Bryotropha nupponeni (BOLD:ADO3225) n=1: RUS
Bryotropha politella (BOLD:AAW7300) n=1: FRA
Bryotropha tachyptilella (BOLD:ADL7141) n=3: GRE, ROU, SVK
Bryotropha italica (BOLD:ADF3309) n=1: ITA
Bryotropha azovica (BOLD:ADL8607) n=2: BGR
Bryotropha sabulosella (BOLD:ADR2629) n=1 : GRE
Bryotropha patockai (BOLD:ADR2630) n=1: CZE

Bryotropha sattleri (BOLD:ADM8851) n=1: ITA
Bryotropha desertella A (BOLD:AAB9592) n=9: AUT, FIN, GER, HRV, NOR
Bryotropha desertella B (BOLD:ADJ4208) n=3: AUT, GRE
Bryotropha desertella C (BOLD:ADC7722) n=1 : NOR
Bryotropha heckfordi (BOLD:ADM9910) n=1: ESP
Bryotropha wolschrijni (BOLD:ADN0112) n=1 : ESP
Bryotropha terrella A (BOLD:ACP8843) n=2: AUT
Bryotropha terrella B (BOLD:AAB9591) n=57: AUT, FIN, GER, GRE, ITA, NOR

NJ tree 17
Carpatolechia fugitivella (BOLD:AAA7652) n=33: AUT, FIN, GER, ITA, NOR
Carpatolechia decorella (BOLD:AAK2790) n=22: AUT, BGR, FIN, FRA. GER, HRV, ITA, NOR
Carpatolechia notatella (BOLD:AAD6612) n=15: AUT, FIN, GER, ITA, NOR
Carpatolechia proximella (BOLD:AAA6352) n=33: AUT, FIN, GER, ITA, NOR
Carpatolechia filipjevi (BOLD:ADO3061) n=1: RUS
Carpatolechia alburnella (BOLD:AAF1850) n=20: AUT, FIN, GER, ITA, NOR
Carpatolechia fugacella (BOLD:AAN0741) n=20: AUT, GER, ITA
Carpatolechia epomidella (BOLD:AAF1847) n=3: FIN
Carpatolechia intermediella (BOLD:ADR7611) n=1: ESP
Neotelphusa cisti (BOLD:ADJ0429) n=1: FRA
Neotelphusa sequax (BOLD:AAL5674) n=24: AUT, FIN, GER, ITA, SVN
Neotelphusa huemeri (BOLD:ADM2537) n=1 : ESP
Carpatolechia aenigma (BOLD:AAV7729) n=4: ITA, ROU

NJ tree 18
Caryocolum viscariella (BOLD:AAI6568) n=14: AUT, FIN, FRA, ITA, NOR
Caryocolum alsinella A {BOLD:AAV7764) n=1: ITA
Caryocolum alsinella B (BOLD:ADG5831) n=3: ITA, MKD, NOR
Caryocolum alsinella C (BOLD:AAV0572) n=14: AUT, BGR, DNK, FIN, HUN, ITA
Caryocolum albifaciella (BOLD:AAK2832) n=4: AUT, ITA
Caryocolum bosalella (BOLD:ADL6231) n=2: ITA
Caryocolum vicinella A (BOLD:ABA3050) n=2: GER, ITA
Caryocolum vicinella B (BOLD:ACY9466) n=1: NOR
Caryocolum vicinella C (BOLD:AAL6797) n=3: FIN
Caryocolum vicinella D (BOLD:ACC3292) n=13: AUT, GER. ITA
Caryocolum petryi (BOLD:AAM0549) n=8: AUT, CHE, FIN, SVN
Caryocolum crypticum (BOLD:ACJ5624) n=3: ITA
Caryocolum baischi (BOLD:ADN9692) n=1: GRE
Caryocolum mucronatella (BOLD:AAW4102) n=7: AUT, GER, ITA
Caryocolum amaurella A (BOLD:AAE9473) n=3: FIN, NOR
Caryocolum amaurella B (BOLD:ACE5885) n=2: AUT
Caryocolum amaurella C (BOLD:ADL1729) n=2: AUT, NOR
Caryocolum amaurella D (BOLD:ABA2355) n=2: MKD
Caryocolum amaurella E (BOLD:AAE9474) n=12: FIN, NOR
Caryocolum siculum (BOLD:ABV4977) n=1: ITA
Caryocolum repentis (BOLD:AAV7766) n=7: AUT, GER, ITA
Caryocolum sciurella (BOLD:ADL5956) n=2: ESP, PRT
Caryocolum fischerella (B0 LD:AAI6565) n=8: AUT, FIN, GER
Caryocolum tischeriella A (BOLD:ADR7700) n=1: FRA
Caryocolum tischeriella B (BOLD:ADR2929) n=13: AUT, GER, ITA, NOR
Caryocolum tischeriella B (BOLD:ADR2929) n=2: AUT, ITA

Caryocolum tischeriella C (BOLD:AAI6566) n=S: FIN, ITA, MKD
Caryocolum saginella A (BOLD:AAI6574) n=2: ITA
Caryocolum cauligenella A (BOLD:AAU3080) n=1: ESP
Caryocolum cauligenella B (BOLD:AAP7513) n=7: AUT, FIN, ITA
Caryocolum saginella B (BOLD:ADM2047) n=9: AUT
Caryocolum leucofasciatum (BOLD:ADL9319) n=1: ESP
Caryocolum oculatella (BOLD:ABV4976) n=1: AUT
Caryocolum pullatella A (BOLD:AAC1598) n=8: AUT, ITA, MKD
Caryocolum pullatella B (BOLD:AAC1599) n=8: FIN, NOR
Caryocolum marmorea A (BOLD:ADM7016) n=1: ITA
Caryocolum marmorea B (BOLD:ACJ5938) n=3: ITA
Caryocolum marmorea C (BOLD:AAK2828) n=4: DNK, ESP, FRA
Caryocolum delphinatella (BOLD:AAK2827) n=5: FRA, ITA
Caryocolum provinciella (BOLD:ADO2699) n=1: ESP
Caryocolum trauniella (BOLD:AAO3810) n=3: AUT, SVN
Caryocolum peregrinella A (BOLD:AAC6817) n=6: AUT, ITA
Caryocolum peregrinella B (BOLD:AAC6818) n=8: GRE, MKD, SVN
Caryocolum peregrinella C (BOLD:AAC6816) n=4: FRA, ITA
Caryocolum peregrinella D (BOLD:ACK3061) n=2: ESP
Caryocolum leucomelanella A (BOLD:AAM3503) n=20: AUT, FRA, GER, HRV, ITA, MKD
Caryocolum leucomelanella B (BOLD:ACI3418) n=3: AUT, GER
Caryocolum schleichi A (BOLD:AAE9478) n=7: ITA
Caryocolum schleichi B (BOLD:AAU1854) n=1: ESP
Caryocolum schleichi C (BOLD:ACB5067) n=7: CHE, FRA, GER
Caryocolum schleichi D (BOLD:ACL3018) n=3: AUT, ITA
Caryocolum schleichi E (BOLD:ADH6455) n=1: AZE
Caryocolum schleichi F (BOLD:ACT3307) n=4: BGR, GRE, ITA
Caryocolum arenariella (BOLD:AAE9479) n=5: FIN, MKD, SWE
Caryocolum leucothoracellum (BOLD:AAO3805) n=6: FRA, ITA
Caryocolum mazeli (BOLD:ABY3315) n=3: FRA, ITA
Caryocolum gallagenellum (BOLD:ABV4975) n=1: FRA
Caryocolum confluens (BOLD:ABW9623) n=1: GRE
Caryocolum fraternella (BOLD:AAI6571) n=8: FIN, FRA, NOR
Caryocolum junctella A (BOLD:ACY8911) n=5: GER, NOR
Caryocolum junctella B (BOLD:AAQ1185) n=6: AUT, FIN
Caryocolum jaspidella (BOLD:ABW6542) n=2: PRT
Caryocolum fibigerium A (BOLD:AAO2674) n=2: ITA
Caryocolum fibigerium B (BOLD:ADK9243) n=1: ITA
Caryocolum fibigerium C (BOLD:ACC2659) n=3: GRE, MKD
Caryocolum fibigerium D (BOLD:AAU3076) n=5: ESP, FRA
Caryocolum tricolorella (BOLD:AAF1506) n=7: FIN, GER
Caryocolum laceratella (BOLD:AAO4665) n=1: SVN
Caryocolum dauphini (BOLD:ACE2159) n=2: FRA
Caryocolum klosi A (BOLD:ABW0326) n=1: FRA
Caryocolum klosi B (BOLD:AAY7823) n=3: AUT, GER
Caryocolum interalbicella (BOLD:AAI6572) n=7: AUT, CHE, ITA
Caryocolum huebneri (BOLD:ABW6646) n=1: AUT
Caryocolum petrophila (BOLD:AAO3809) n=12: CHE, FIN, ITA, MKD, SVN
Caryocolum moehringiae (BOLD:ACR3961) n=13: AUT, GER
Caryocolum proxima (BOLD:AAH4687) n=9: AUT, ITA
Caryocolum arenbergeri (BOLD:AAV7765) n=1: ESP

Caryocolum blandulella (BOLD:AAV7765) n=5: DNK, GRE, SWE
Caryocolum kroesmanniella (BOLD:AAl6573) n=S: FIN, FRA, GER
Caryocolum blandella (BOLD:AAI6556) n=9: FIN, GER, NOR
Caryocolum horoscopa (BOLD:ADN1280) n= 1: TUR
Caryocolum blandelloides (BOLD:AAK2834) n=8: FIN, GER, GRE, NOR
Caryocolum cassella (BOLD:ACX4318) n=20: AUT, FIN, GER, ITA, NOR

NJ tree 19
Caulastrocecis cryptoxena (BOLD:ABW9600) n=5: AUT, SVK, UKR
Caulastrocecis sp.1 (BOLD:ADR7056) n=1: ITA
Caulastrocecis gypsella (BOLD:ADG7214) n=2: FRA
Caulastrocecis perexigella (BOLD:ACB0591) n=4: RUS
Caulastrocecis pudicella (missing) n=1: SVK
Caulastrocecis interstratella (BOLD:ACB0451) n=1: RUS
Caulastrocecis sp. 2 (BOLD:ADM1812) n=3: GRE, HRV
Paranarsia joannisiella (BOLD:AAV3498) n=3: AUT, ITA

NJ tree 20
Psamathocrita dalmatinella (BOLD:ACW1421) n=5: ESP, FRA, HRV
Psamathocrita sp.1 (BOLD:ADL7901) n=1: HRV
Psamathocrita osseella (BOLD:ACI0510) n=4: BGR, HRV
Psamathocrita argentella (BOLD:ACG2666) n=7: FRA, GBR, HUN, ITA
Psamathocrita sp.2 (BOLD:ADF0071) n=1: CYP
Chimericorsa nioloensis (BOLD:ACZ3221) n=1: FRA
Dirhinosia arnoldiella (BOLD:ADL8839) n=1: GRE
Dirhinosia cervinella (BOLD:ACB0757) n=7: BGR, RUS, UKR
Dirhinosia interposita (BOLD:ACB0757) n=3: UKR

NJ tree 21
Chionodes fumatella A (BOLD:AAC7811) n=39: AUT, FIN, FRA, GER, ITA, MKD, NOR, ROU
Chionodes fumatella B (BOLD:ADL0221) n=6: AUT, CHE, ITA
Chionodes fumatella C (BOLD:AAl4278) n=6: FIN
Chionodes ignorantella (BOLD:AAP7487) n=7: FIN, GER, NOR
Chionodes violacea (BOLD:AAJ0091) n=5: FIN
Chionodes viduella A (BOLD:ACF2998) n=5: CHE, FIN, GER, NOR
Chionodes viduella B (BOLD:ACF2999) n=1: FRA
Chionodes viduella C (BOLD:AAJ0096) n=11 : AUT, FIN, NOR
Chionodes nebulosella (BOLD:AAE2228) n=11: AUT, BGR, GER, ITA
Chionodes continuella (BOLD:AAB4514) n=10: FIN, NOR
Chionodes distinctella A (BOLD:AAE9035) n=3: FIN
Chionodes distinctella B (BOLD:AAE9035) n=50: AUT, FIN, GER, ITA, MKD, NOR, RUS
Chionodes aprilella (BOLD:ACB1026) n=1: RUS
Chionodes holosericella (BOLD:ABY6213) n=23: AUT, CHE, FIN, GER, ITA, NOR, RUS
Chionodes electella (BOLD:AAD4848) n=27: AUT, FIN, GER, ITA, NOR
Chionodes soella (BOLD:ADN6193) n=1: RUS
Chionodes tragicella (BOLD:AAJ0097) n=12: AUT, FIN, GER, ITA
Chionodes perpetuella (BOLD:AAJ4702) n=9: AUT, ITA
Chionodes nubilella A (BOLD:AAE9042) n=1: NOR
Chionodes nubilella B (BOLD:ACF2229) n=1: NOR
Chionodes apolectella (BOLD:ADL8627) n=1: FRA
Chionodes bastuliella (missing) n=1: ESP

Chionodes hayreddini (BOLD:ACJ9735) n=8: AUT, ITA
Chionodes luctuella A (BOLD:ABY8068) n=22: AUT, GER, ITA
Chionodes luctuella B (BOLD:AAD2579) n=5: FIN, NOR
Chionodes praeclarella (BOLD:AAY8727) n=8: AUT, ITA
Chionodes lugubrella (BOLD:AAB8866) n=19: AUT, FIN, FRA, ITA, NOR

NJ tree 22
Chrysoesthia sexguttella (BOLD:AAD8505) n=9: AUT, FIN, HRV, NOR, RUS
Chrysoesthia sp.1 (BOLD:ACS7804) n=1: UKR
Chrysoesthia sp.2 (BOLD:ADN7772) n=2: ESP
Chrysoesthia boseae (BOLD:ADL6012) n=2: ESP
Chrysoesthia eppelsheimi (BOLD:ADR6879) n=1: GRE
Chrysoesthia verrucosa (BOLD:ADL9791) n=1: CZE
Chrysoesthia sp. 3 (BOLD:ADM8914) n=1: ESP
Chrysoesthia gaditella (BOLD:ADM0528) n=2: ESP
Chrysoesthia falkovitchi (missing) n=2: UKR
Chrysoesthia drurella A (BOLD:AAR9709) n=2: AUT
Chrysoesthia drurella B (BOLD:AAF2806) n=12: AUT, FIN, GER, ITA, NOR
Chrysoesthia hispanica (BOLD:ADM2746) n=2: ESP
Metanarsia incertella (BOLD:ADL7455) n=1 : ESP
Metanarsia guberlica (BOLD:ACB1007) n=1: RUS
Metanarsia modesta A (BOLD:ACI1984) n=1: ARM
Metanarsia modesta B (missing) n=3: UKR

NJ tree 23
Klimeschiopsis kiningerella A (BOLD:AAF3126) n=16: AUT, FIN, GER, NOR, ROU
Klimeschiopsis kiningerella B (BOLD:AAP5746) n=3: ITA
Klimeschiopsis discontinuella (BOLD:AAX0326) n=3: ITA
Klimeschiopsis maritimaealpina (BOLD:ACA9638) n=3: FRA, ITA
Klimeschiopsis terroris (BOLD:AAV6681) n=2: ESP
Lutilabria lutilabrella A (BOLD:ADL2704) n=2: SVK
Lutilabria lutilabrella B (BOLD:ABW4984) n=1: SVN
Lutilabria volgensis (BOLD:ACB0680) n=1: RUS
Lutilabria prolata (BOLD:ACT0912) n=1: UKR
Cosmardia moritzella (BOLD:AAJ5578) n=9: AUT, FIN, GER, GRE, ITA, NOR

NJ tree 24
Ivanauskiella occitanica (BOLD:ABW5477) n=5: FRA, HRV, ITA
Ivanauskiella psamathias (BOLD:ADD9916) n=2: BGR, RUS
Ivanauskiella sp.1 (BOLD:ACB0708) n=2: RUS
Deltophora stictella (BOLD:ADM8283) n=3: ESP, FRA, ITA
Deltophora gielisia (missing) n=1: ESP
Deltophora maculata (BOLD:ADF1116) n=2: GRE
Spiniphallellus desertus (BOLD:ACB3153) n=1: RUS
Spiniphallellus chrysotosella (BOLD:ACW1628) n=1: BGR

NJ tree 25
Microlechia chretieni (BOLD:AAU3189) n=4: ESP
Microlechia rhamnifoliae (BOLD:ADN2422) n=1: CYP
Microlechia klimeschi (missing) n=1: ESP

Microlechia karsholti (missing) n=1: RUS
Ephysteris iberica (BOLD:AAV6506) n=2: ESP
Ephysteris deserticolella (BOLD:ACT4402) n=1: UKR
Ephysteris promptella A (BOLD:ACR6646) n=3: GRE
Ephysteris promptella B (BOLD:AAU3945) n=1: ESP
Ephysteris promptella C (BOLD:AAU3944) n=1 : ESP
Ephysteris promptella D (BOLD:ADY9845) n=2: HRV, UKR
Ephysteris olympica (BOLD:ADM1235) n=1: FRA
Ephysteris diminutella A (BOLD:ADK8538) n=4: HRV, ITA
Ephysteris diminutella B (BOLD:ABA7116) n=4: MKD, RUS
Ephysteris inustella (BOLD:ACR2218) n=9: FRA, ITA
Ochrodia sp.1 (BOLD:ACE0260) n=1 : GRE
Ochrodia subdiminutella (BOLD:AAU1967) n=5: ESP

NJ tree 26
Epidola sp.1 (BOLD:ADF2272) n=4: ESP, GRE, HRV
Epidola nuraghella (BOLD:ADM4659) n=3: ITA
Epidola semitica (BOLD:ADM1116) n=2: GRE
Epidola barcinonella (BOLD:ADR9309) n=1: ESP
Epidola stigma (BOLD:ABX1734) n=2: FRA, PRT

NJ tree 27
Filatima spurcella (BOLD:AAV9861) n=6: AUT, HRV, ITA, ROU
Filatima pallipalpella (BOLD:AAY8466) n=1: RUS
Filatima transsilvanella (BOLD:ADN6902) n=1: ROU
Filatima tephritidella (BOLD:ADL6299) n=1: BGR
Filatima ukrainica (BOLD:ADL8238) n=1: LTU
Filatima incomptella (BOLD:AAJ0374) n=4: FIN, NOR
Filatima textorella (BOLD:ADR1852) n=1: FRA
Filatima zagulajevi (missing) n=1: RUS

NJ tree 28
Gelechia sororculella (BOLD:AAC8633) n=29: AUT, FIN, GER, ITA, MKD, NOR
Gelechia jakovlevi (BOLD:AAE6376) n=3: FIN
Gelechia cuneatella (BOLD:AAF5086) n=7: AUT, FIN, GER, NOR
Gelechia nervosella (BOLD:ADL8973) n=3: ESP
Gelechia senticetella A (BOLD:AAE6366) n=3: AUT, GER, ITA
Gelechia senticetella B (BOLD:ADF0597) n=3: GRE
Gelechia obscuripennis (BOLD:ABY8998) n=9: AUT, BGR, ITA, MKD
Gelechia asinella (BOLD:AAY9300) n=4: AUT, ITA
Gelechia atlanticella (BOLD:ADN6612) n=1: ESP
Gelechia sp.1 (BOLD:ADF0061) n=1: GRE
Gelechia sabinella (BOLD AAD7093) n=34: AUT, ESP, FIN, GER, ITA, NORGelechia aspoecki (BOLD:AAU0134) n=2: ESP, FRA
Gelechia turpella (BOLD:AAI7030) n=9: AUT, FIN, GER, ITA
Gelechia basipunctella A (BOLD:AAY1122) n=2: AUT, ITA
Gelechia basipunctella B (BOLD:ADI5669) n=3: AUT, GER
Gelechia nigra (BOLD:AAF5081) n=20: AUT, FIN, GER, ITA, NOR
Gelechia muscosella (BOLD:AAC8632) n=32: AUT, FIN, GER, ITA, NOR
Gelechia sestertiella (BOLD:AAI7031) n=18: AUT, FIN, GER, ITA, LVA
Gelechia dujardini (BOLD:ADK9995) n=2: HRV

Gelechia mediterranea (BOLD:ADE9654) n=2: GRE
Gelechia rhombelliformis (BOLD:AAE6371) n=7: AUT, GER
Gelechia rhombella (BOLD:AAE6372) n=16: AUT, FIN, GER, ITA, NOR
Gelechia hippophaella (BOLD:AAG0519) n=7: AUT, FIN
Gelechia scotinella A (BOLD:ACQ9801) n=3: MKD, ROU, SWE
Gelechia scotinella B (BOLD:ACQ9800) n=6: GER, GRE
Gelechia scotinella C (BOLD:AAI7032) n=22: AUT, FIN, GER, ITA

NJ tree 29
Gnorimoschema hoefneri (BOLD:AAF8265) n=6: AUT, ITA, SVN
Gnorimoschema streliciella (BOLD:AAF8271) n=6: FIN, NOR
Gnorimoschema nilsi (BOLD:AAL3826) n=1: AUT
Gnorimoschema nordlandicolella (BOLD:AAA7774) n=3: FIN
Gnorimoschema valesiella (BOLD:AAA7775) n=8: ESP, FIN, ITA, NOR
Gnorimoschema steueri (BOLD:ADE0307) n=1: ITA
Gnorimoschema nupponeni (BOLD:ACB3130) n=4: RUS
Gnorimoschema robustella (BOLD:ACB0967) n=1: RUS
Gnorimoschema soffneri (BOLD:ADM1971) n=2: GRE, HUN
Gnorimoschema bodillum (missing) n=1: DNK
Gnorimoschema herbichii A (BOLD:AAL5587) n=2: FIN
Gnorimoschema herbichii B (BOLD:AAA6744) n=4: DNK, FIN, NOR
Gnorimoschema epithymella (BOLD:AAE7091) n=12: AUT, FIN, ITA, NOR
Scrobipalpopsis petasitis (BOLD:AAE1641) n=16: AUT, CHE, FIN, GER, ITA
Tecia solanivora (BOLD:ACP2393) n=1: ESP

NJ tree 30
Pexicopia malvella A (BOLD:AAD9025) n=26: AUT, FIN, GER, GRE, NOR
Pexicopia malvella B (BOLD:ACB8817) n=3: GER
Pectinophora gossypiella (BOLD:AAH4802) n=1: PRT
Platyedra subcinerea (BOLD:AAD8749) n=9: AUT, GER, ITA
Harpagidia magnetella (BOLD:ADM0785) n=1: ARM
Sitotroga cerealella (BOLD:AAD0546) n=1: GER
Sitotroga psacasta (BOLD:ADF3395) n=1: ESP

NJ tree 31
Neofaculta infernella (BOLD:AAC1363) n=63: AUT, CHE, DNK, FIN, GER, ITA, NOR
Neofaculta taigana (BOLD:AAC1362) n=20: FIN, NOR
Neofaculta ericetella A (BOLD:AAU3509) n=1: ESP
Neofaculta ericetella B (BOLD:ADM8824) n=2: FRA, ITA
Neofaculta ericetella C (BOLD:AAD1501) n=33: AUT, ESP, FIN, GER, ITA, NOR
Hypatima rhomboidella A (BOLD:AAD1449) n=5: AUT, FIN, GER
Hypatima rhomboidella B (BOLD:ABY9559) n=3: AUT, GER, ITA
Hypatima rhomboidella C (BOLD:AAD1448) n=14: AUT, FIN, GER, ITA, NOR
Nothris sulcella (BOLD:ADH7559) n=2: BGR, GRE
Nothris radiata (BOLD:ADL7080) n=2: MKD, TUR
Nothris lemniscellus (BOLD:AAI2382) n=19: AUT, FRA, GER, ITA, SVN
Nothris gregerseni A (BOLD:ACY8316) n=1: SWE
Nothris gregerseni B (BOLD:AAI2383) n=6: AUT, CZE, FIN, RUS
Nothris congressariella (BOLD:AAK4334) n=3: ITA
Nothris skyvai (BOLD:ADL6473) n=1: HRV
Nothris verbascella (BOLD:AAE5563) n=24: ARM, AUT, FIN, GER, GRE, ITA, MKD, NOR

NJ tree 32
Isophrictis kefersteiniellus A (BOLD:ADR3447) n=2: HRV
Isophrictis kefersteiniellus B (BOLD:AAU3278) n=2: ESP
Isophrictis kefersteiniellus C (BOLD:ADR3447) n=3: BGR, GRE, HRV
Isophrictis kefersteiniellus D (BOLD:ADM8862) n=1: GRE
Isophrictis kefersteiniellus E (BOLD:ADF4024) n=1: ESP
Isophrictis lineatella (BOLD:ADL8364) n=3: ESP, ITA
Isophrictis corsicella (BOLD:ADL8363) n=1: ITA
Isophrictis sp.1 (BOLD:ADI3246) n=1: GRE
Isophrictis meridionella A (BOLD:ADF3165) n=1: BGR
Isophrictis meridionella B (BOLD:ADM8251) n=1: ESP
Isophrictis meridionella C (BOLD:AAU3277) n=2: ESP
Isophrictis meridionella D (BOLD:ADM0460) n=4: ESP
Isophrictis anthemidella A (BOLD:AAI8202) n=1: FIN
Isophrictis anthemidella A (BOLD:AAI8202) n=2: FIN
Isophrictis anthemidella B (BOLD:ADS1791) n=1: GER
Isophrictis anthemidella C (BOLD:ADL5673) n=1: ITA
Isophrictis anthemidella D (BOLD:AAU3266) n=5: GER
Isophrictis striatella (BOLD:AAD5050) n=32: AUT, FIN, GER, ITA, MKD, NOR
Parapodia sinaica A (BOLD:ADL8290) n=1: CYP
Parapodia sinaica B (BOLD:ADL6186) n=3: ESP

NJ tree 33
Istrianis arenicolella (BOLD:ADO3473) n=2: FRA
Istrianis myricariella (BOLD:ADM0985) n=2: FRA, GRE
Istrianis nilssoni (BOLD:ADR4954) n=1: GRE
Istrianis femoralis (BOLD:ADF1591) n=2: GRE, SVN
Streyella canariensis (BOLD:ADL7266) n=2: ESP
Streyella anguinella (BOLD:ADM1557) n=2: ESP, SVN

NJ tree 34
Scrobipalpula tussilaginis (BOLD:AAF1106) n=13: AUT, DNK, GER, ITA
Scrobipalpula diffluella (BOLD:AAF1106) n=12: AUT, CHE, FIN, GER, ITA, NOR
Scrobipalpula ramosella (BOLD:AAF1106) n=1: CHE
Scrobipalpula seniorum (BOLD:AAF1106) n=1: MKD
Scrobipalpula psilella (BOLD:AAF1106) n=9: AUT, CHE, FIN, GER, NOR, SWE
Tuta absoluta (BOLD:AAJ8033) n=3: FRA, NOR, PRT
Phthorimaea operculella (BOLD:AAB9396) n=1 : ITA

NJ tree 35
Megacraspedus lanceolellus (18 BINs) n=41: AND, ESP, FRA, ITA
Megacraspedus bengtssoni (BOLD:ACM1097) n=4: ESP
Megacraspedus faunierensis (BOLD:AAJ3164) n=4: ITA
Megacraspedus gredosensis (BOLD:ADI8272) n=1: ESP
Megacraspedus sumpichi (BOLD:ACM1096) n=5: ESP
Megacraspedus bidentatus (BOLD:ADA0203) n=2: ESP, FRA
Megacraspedus cuencellus (BOLD:ACC5029) n=2: ESP
Megacraspedus fuscus (BOLD:ADM1005) n=1: ESP
Megacraspedus sp.1 (BOLD:ACZ8654) n=1: ESP
Megacraspedus trineae (BOLD:ADF0469) n=2: PRT

Megacraspedus tristictus (BOLD:ADA0606) n=1: FRA
Megacraspedus heckfordi (BOLD:ACA9758) n=4: ESP
Megacraspedus tenuiuncus (BOLD:ADF1915) n=1: ESP
Megacraspedus pusillus (BOLD:ACZ8007) n=1: ESP
Megacraspedus skoui (BOLD:ACT1624) n=1 : ESP
Megacraspedus spinophallus A (BOLD:ACT2894) n=1: ESP
Megacraspedus spinophallus B (BOLD:AAU1828) n=9: ESP
Megacraspedus binotella A (BOLD:ACM0962) n=4: AUT, GER
Megacraspedus binotella B (BOLD:ADl7972) n=1: AUT
Megacraspedus binotella C (BOLD:ACW5732) n=1: AUT
Megacraspedus devorator (BOLD ADB7270) n=2: BGR, ROU
Megacraspedus barcodiellus (BOLD:ABA2916) n=3: MKD
Megacraspedus brachypteris A (BOLD:ADR2636) n=1: BGR
Megacraspedus brachypteris B (BOLD:ABA3165) n=3: MKD
Megacraspedus brachypteris C (BOLD:ACS7816) n=1: MKD
Megacraspedus brachypteris D (BOLD:ACZ3665) n=1: MKD
Megacraspedus brachypteris E (BOLD:ADJ3544) n=2: MKD
Megacraspedus tokari (BOLD:ADS0140) n=2: HRV
Megacraspedus similellus (BOLD:ADB8685) n=3: BGR
Megacraspedus uzunsyrtus (BOLD:ACS7353) n=2: UKR
Megacraspedus dolosellus (22 BINs) n=51: AUT, BGR, GRE, HRV, HUN, ITA, MKD, ROU, RUS, SVN, UKR
Megacraspedus quadristictus (BOLD:AAU1830) n=6: ESP
Megacraspedus korabicus (BOLD ABA2917) n=3: MKD
Megacraspedus teriolensis A (BOLD:ADF1918) n=1: GRE
Megacraspedus teriolensis B (BOLD:ABW5890) n=5: HRV. ITA, SVN
Megacraspedus multispinella (BOLD:ACM0852) n=1: RUS
Megacraspedus attritellus A (BOLD:ACE2700) n=3: RUS
Megacraspedus attritellus B (BOLD:ACB0770) n=1: RUS
Megacraspedus cerussatellus (BOLD:ACA8764) n=1: BGR
Megacraspedus imparellus A (BOLD:ADR9079) n=1: GRE
Megacraspedus imparellus B (BOLD:ACB3182) n=1: RUS
Megacraspedus imparellus C (BOLD:ADB7271) n=1: GRE
Megacraspedus imparellus D (BOLD:ABW9450) n=2: AUT. HUN
Megacraspedus lagopellus (BOLD:ACB0458) n=2: RUS
Megacraspedus skulei (BOLD:ACM0982) n=6: ESP
Megacraspedus eburnellus (BOLD:AAJ3176) n=2: ITA
Megacraspedus sp.2 (BOLD:ADY4582) n=1: ITA
Megacraspedus peyerimhoffi (BOLD:ACC5030) n=4: ESP
Megacraspedus ibericus (BOLD:ACZ7298) n=1: ESP
Megacraspedus peslieri (BOLD:ADM8362) n=1: FRA
Megacraspedus squalida (BOLD:AAU3336) n=6: ESP
Megacraspedus andreneli A (BOLD:ADG6163) n=1: FRA
Megacraspedus andreneli B (BOLD:ACS0692) n=1: FRA
Megacraspedus bilineatella (BOLD:ABU7227) n=1: ITA
Megacraspedus gallicus (BOLD:ACF7111) n=6: ESP, FRA.
Megacraspedus ribbeella (BOLD:ACZ9288) n=2: ESP
Megacraspedus numidellus (BOLD:ADA0605) n=1: ESP
Megacraspedus albovenata (BOLD:ACE2688) n=2: RUS
Megacraspedus podolicus (BOLD:ADB8683) n= 1: RUS
Megacraspedus balneariellus A (BOLD:ADR2637) n=1: FRA

Megacraspedus balneariellus B (BOLD:ADB9039) n=2: HRV
Megacraspedus fallax (BOLD:ACB0437) n=3: RUS
Megacraspedus longipalpella (BOLD:ACM1349) n=1: RUS
Megacraspedus niphorrhoa (BOLD:ACB3210) n=5: KAZ, RUS

NJ tree 36
Megacraspedus dolosellus A (BOLD:ABA2915) n=10: MKD
Megacraspedus dolosellus B (BOLD:ADB8790) n=2: MKD
Megacraspedus dolosellus C (BOLD:ADR5927) n=1: MKD
Megacraspedus dolosellus D (BOLD:ADB8686) n=4: HRV
Megacraspedus dolosellus E (BOLD:AAG0031) n=4: HUN, ROU
Megacraspedus dolosellus F (BOLD:ACS9604) n=2: AUT
Megacraspedus dolosellus G (BOLD:ADR7287) n=1: HRV
Megacraspedus dolosellus H (BOLD:AAV7561) n=4: SVN
Megacraspedus dolosellus I (BOLD:ACZ3281) n=1: RUS
Megacraspedus dolosellus J (BOLD:ACZ3530) n=3: RUS
Megacraspedus dolosellus K (BOLD:ADA0140) n=1: UKR
Megacraspedus dolosellus L (BOLD:ADB8789) n=1: BGR
Megacraspedus dolosellus M (BOLD:ADB8684) n=1: BGR
Megacraspedus dolosellus N (BOLD:ACZ9025) n=1: BGR
Megacraspedus dolosellus O (BOLD:ACR2396) n=5: BGR
Megacraspedus dolosellus P (BOLD:ACQ6924) n=2: GRE
Megacraspedus dolosellus Q (BOLD:ACS7817) n=1: GRE
Megacraspedus dolosellus R (BOLD:ACA9065) n=3: BGR
Megacraspedus dolosellus S (BOLD:AAO3318) n=1: ITA
Megacraspedus dolosellus T (BOLD:AAO3319) n=1: ITA
Megacraspedus dolosellus U (BOLD:AAX3311) n=1: ITA
Megacraspedus dolosellus V (BOLD:ACZ7902) n=1: ITA

NJ tree 37
Megacraspedus lanceolellus A (BOLD:ADF2263) n=3: ESP
Megacraspedus lanceolellus B (BOLD:ACM1006) n=2: ESP
Megacraspedus lanceolellus C (BOLD:ADB7571) n=1: ESP
Megacraspedus lanceolellus D (BOLD:ADF2194) n=1: ESP
Megacraspedus lanceolellus E (BOLD:ACZ8269) n=1: ESP
Megacraspedus lanceolellus F (BOLD:ADF2256) n=2: ESP
Megacraspedus lanceolellus G (BOLD:ADF1916) n=1: ESP
Megacraspedus lanceolellus H (BOLD:AAU1829) n=5: ESP
Megacraspedus lanceolellus I (BOLD:ACZ2607) n=2: ESP
Megacraspedus lanceolellus J (BOLD:ADJ0160) n=2: ESP
Megacraspedus lanceolellus K (BOLD:ADM7770) n=3: FRA
Megacraspedus lanceolellus L (BOLD:ABA3648) n=2: FRA
Megacraspedus lanceolellus M (BOLD:ABA3649) n=1: FRA
Megacraspedus lanceolellus M (BOLD:ABA3649) n=1: FRA
Megacraspedus lanceolellus N (BOLD:ACZ2656) n=1: ITA
Megacraspedus lanceolellus O (BOLD:ACA9759) n=2: AND
Megacraspedus lanceolellus P (BOLD:ACZ8656) n=2: ESP
Megacraspedus lanceolellus Q (missing) n=1: ESP
Megacraspedus lanceolellus R (BOLD:ACZ9024) n=2: ESP
Megacraspedus lanceolellus S (BOLD:ACZ2657) n=1: ITA
Megacraspedus lanceolellus T (BOLD:ACZ3381) n=1: ITA

Megacraspedus lanceolellus U (BOLD:AAU2834) n=4: ITA

NJ tree 38
Mesophleps silacella (BOLD:AAF2204) n=12: AUT, FIN, FRA, GER, ITA, SWE
Mesophleps corsicella (BOLD:AAU3613) n=3: ESP, GRE
Mesophleps ochraceella (BOLD:AAV4323) n=7: ESP, GRE
Mesophleps oxycedrella (BOLD:AAX3833) n=2: ESP, GER
Mesophleps sp. 1 (BOLD:ADM4492) n=2: GRE
Mesophleps sp. 2 (BOLD:AAU3614) n=2: ESP
Mesophleps trinotella (BOLD:AAV7800) n=10: AUT, ESP, GRE, HUN, ITA, MKD

NJ tree 39
Metzneria metzneriella A (BOLD:AAD6784) n=20: AUT, GER, ITA, MKD, NOR
Metzneria metzneriella B (BOLD:ACF0151) n=14: AUT, FIN, HRV, ITA, NOR
Metzneria metzneriella C (BOLD:AAV4652) n=1: ESP
Metzneria metzneriella D (BOLD:ABW0199) n=2: ITA
Metzneria tristella (BOLD:ABX1726) n=1: FRA
Metzneria santolinella (BOLD:AAH4048) n=5: ESP, FIN, FRA
Metzneria hilarella (BOLD:AAV4651) n=4: ESP, FRA
Metzneria staehelinella (BOLD:ACW2108) n=1: BGR
Metzneria intestinella (BOLD:ACW1685) n=1: GRE
Metzneria sp.1 (BOLD:ADM8252) n=2: ESP
Metzneria ehikeella A (BOLD:AAN2252) n=5: DNK, FIN, GER, MKD
Metzneria ehikeella B (BOLD:ACW7184) n=1: GER
Metzneria paucipunctella (BOLD:AAH4275) n=9: AUT, FRA, ESP
Metzneria aestivella (BOLD:AAK7524) n=14: AUT, BGR, GRE, HRV, ITA
Metzneria expositoi (BOLD:AAK7524) n=1: ESP
Metzneria castiliella (BOLD:ABW1526) n=4: ESP, GRE
Metzneria torosulella (BOLD:ADM4637) n=2: ESP, PRT
Metzneria fulva (BOLD:ADM4637) n=1: FRA
Metzneria sp.2 (BOLD:ACB3385) n=1: RUS
Metzneria diffusella (BOLD:ABX1375) n=2: FRA
Metzneria neuropterella A (BOLD:AAH3862) n=4: FIN, ITA, MKD, ROU
Metzneria neuropterella B (BOLD:AAH3861) n=11: AUT, ESP, FIN, GER, ITA, NOR
Metzneria sp.3 (BOLD:ABW1820) n=1: ESP
Metzneria subflavella (BOLD:ADM8253) n=1: FRA
Metzneria aprilella A (BOLD:ABW0030) n=3: HRV, ITA
Metzneria aprilella B (BOLD:ABW0031) n=6: BGR, GRE, ITA
Metzneria aprilella C (BOLD:AAB4322) n=16: AUT, FIN, GER, ITA, NOR
Metzneria lappella (BOLD:AAB4321) n=23: AUT, ESP, FIN, GER, ITA, NOR
Metzneria agraphella (BOLD:ADM1031) n=1: GRE
Metzneria riadella (BOLD ABW1819) n=2: ESP
Metzneria littorella (BOLD:ADL7669) n=5: HRV, MNE
Metzneria tenuiella (BOLD:ADF5115) n=2: BGR, ESP
Metzneria artificella A (BOLD:ABW7147) n=4 AUT, ITA
Metzneria artificella B (BOLD:ADM5891) n=1: GRE
Metzneria artificella C (BOLD:ABW0128) n=2: ESP
Metzneria campicolella (BOLD:ADE9585) n=3 ESP, GRE, HRV
Pyncostola bohemiella (BOLD:ACZ1855) n=1 : AUT

NJ tree 40

Mirificarma lentiginosella A (BOLD:AAO0969) n=8: AUT, GER
Mirificarma lentiginosella B (BOLD:ABY8674) n=5: GER, ITA, SWE
Mirificarma pederskoui (BOLD:ADR3027) n=1: ESP
Mirificarma burdonella A (BOLD:ADO2415) n=1: FRA
Mirificarma burdonella B (BOLD:ADL7124) n=1: FRA
Mirificarma interrupta (BOLD:AAU3669) n=6: ESP, FRA, GER
Mirificarma mulinella (BOLD:AAK6246) n=3: GER, NOR
Mirificarma cabezella (BOLD:ADM0327) n=1: ESP
Mirificarma ulicinella A (BOLD:ADR8143) n=1: FRA
Mirificarma ulicinella B (BOLD:ADN6104) n=1: PRT
Mirificarma fasciata (BOLD:ADL7123) n=1: ESP
Mirificarma monticolella A (BOLD:ADL6256) n=1: BGR
Mirificarma monticolella B (BOLD:ADL8109) n=1: ITA
Mirificarma cytisella A (BOLD:AAH2272) n=2: GER
Mirificarma cytisella B (BOLD:ADR9770) n=1: AUT
Mirificarma cytisella C (BOLD:ACW6477) n=1: GER
Mirificarma cytisella D (BOLD:AAH2271) n=3: ITA
Mirificarma aflavella (BOLD:ADO1004) n=1: GRE
Mirificarma flavella (BOLD:ACH5726) n=2: CYP, MKD
Mirificarma minimella (BOLD:ADF2273) n=2: GRE
Mirificarma rhodoptera (BOLD:ADM2199) n=1: GRE
Mirificarma eburnella (BOLD:ABW9460) n=3: AUT, GRE
Mirificarma denotata (BOLD:ADN5001) n=1: PRT
Mirificarma maculatella (BOLD:AAH7250) n=9: AUT, FRA, GER, ITA, MKD

NJ tree 41
Monochroa lutulentella (BOLD:AAE4780) n=20: AUT, FIN, GER, ITA, MKD, NOR
Monochroa inflexella (BOLD:ACX1099) n=1: AUT
Monochroa sp.1 (BOLD:ACS5726) n=3: AUT, ITA
Monochroa sperata (BOLD:AAX4741) n=1: ITA
Monochroa sp.2 (BOLD:ADL7906) n=1: BGR
Monochroa niphognatha (BOLD:AAZ9444) n=2: DNK, FIN
Monochroa cytisella (BOLD:AAE1922) n=17: AUT, FIN, GER, GRE, ITA, NOR
Monochroa divisella (BOLD:AAY8956) n=2: ITA
Monochroa suffusella (BOLD:AAY8956) n=15: AUT, FIN, GER, ITA, NOR
Monochroa arundinetella (BOLD:AAF9390) n=11: FIN, GER, NOR
Monochroa elongella (BOLD:AAP7522) n=11 : AUT, FIN, GER, NOR
Monochroa lucidella A (BOLD:ABX6146) n=14: FIN, GBR, GER, NOR
Monochroa lucidella B (BOLD:AAE4765) n=1: GBR
Monochroa sp.3 (BOLD:ADR3927) n=1: HVR
Monochroa simplicella (BOLD:AAQ2222) n=4: AUT, GER, RUS
Monochroa hornigi (BOLD:AAK6885) n=35: AUT, FIN, GER, ITA, NOR, SVN
Monochroa moyses (BOLD:ABW4950) n=3: AUT, ITA
Monochroa servella A (BOLD:AAF9070) n=18: AUT, FIN, GER, ITA, SVN
Monochroa servella B (BOLD:AAG0040) n=6: GER
Monochroa tetragonella (BOLD:AAG1037) n=6: FIN, NOR
Monochroa conspersella (BOLD:AAF3517) n=25: AUT, FIN, GER, NOR
Monochroa rumicetella A (BOLD:ABU9493) n=3: ESP, FIN, NOR
Monochroa rumicetella B (BOLD:AAX4743) n=2: FIN
Monochroa rebeli (BOLD:ADL7650) n=1: ESP
Monochroa sepicolella A (BOLD:AAU2813) n=5: AUT, GER, ITA, ROU

Monochroa sepicolella B (BOLD:AAF8604) n=4: FIN, NOR
Monochroa tenebrella A (BOLD:AAG1078) n=12: AUT, FIN, GER, ITA, NOR
Monochroa tenebrella B (BOLD:ADV2423) n=2: BGR, ITA
Monochroa dellabeffai (BOLD:ADR9993) n=1: ITA
Monochroa scutatella (BOLD:ACA9731) n=2: ITA
Monochroa uralensis (missing) n=2: UKR
Monochroa saltenella (BOLD:AAF2711) n=1: NOR
Monochroa palustrellus (BOLD:AAF2711) n=3: FIN
Monochroa sp.4 (BOLD:ACW2532) n=2: HRV
Monochroa ferrea (BOLD:AAF2721) n=7: CHE, FIN, GER
Monochroa sp.5 (BOLD:ADL9322) n=1: ESP
Monochroa bronzella (BOLD:ACA9721) n=2: ITA
Monochroa nomadella A (BOLD:ACU4919) n=7: AUT
Monochroa nomadella B (BOLD:AAU1638) n=2: ITA
Monochroa nomadella C (BOLD:ADR2662) n=3: HRV
Monochroa nomadella D (BOLD:AAG0220) n=5: ITA
Monochroa nomadella E (BOLD:ACB0708) n=1: RUS

NJ tree 42
Neofriseria peliella A (BOLD:AAC7812) n=26: AUT, ESP, FIN, GER, NOR
Neofriseria peliella B (BOLD:ABZ1425) n=15: AUT, FIN, GER, ITA, MKD
Neofriseria singula (BOLD:AAN4153) n=9: BGR, DNK, GER, ITA, SWE
Neofriseria pseudoterrella (missing) n=1: ESP
Neofriseria kuznetzovae (BOLD:ACB1068) n=2: RUS
Neofriseria baungaardiella (BOLD:ADL8355) n=1: ARM
Neofriseria sp.1 (BOLD:ADR5460) n=1 : FRA
Neofriseria hitadoella (BOLD:ADN2521) n=1: MAR
Neofriseria mongolinella (BOLD:ADL7713) n=1: RUS
Prolita solutella (BOLD:AAK9355) n=18: AUT, ESP, GER, HUN, MKD, ROU, SWE
Prolita sexpunctella (BOLD:ABZ5400) n=24: AUT, CHE, ESP, FIN, GER, ITA, MKD, NOR

NJ tree 43
Oxypteryx atrella (BOLD:AAD5702) n=26: AUT, BGR, FIN, GER, ITA, MKD, NOR
Oxypteryx plumbella (BOLD:AAT9257) n=15: AUT, BGR, CHE, GER, GRE, ITA, MKD, SWE
Oxypteryx unicolorella (BOLD:AAD1198) n=36: AUT, FIN, GER, ITA, NOR
Oxypteryx sp.1 (BOLD:ACS7858) n=1: BGR
Oxypteryx isostacta (BOLD:ADO2410) n=1: CYP
Oxypteryx nigritella (BOLD:ADF1166) n=1: GRE
Oxypteryx immaculatella (BOLD:AAV7057) n=9: DNK, ESP, FRA, GRE, ITA
Oxypteryx nigromaculella A (BOLD:ADE9941) n=1: GRE
Oxypteryx nigromaculella B (BOLD:AAW5089) n=3: BGR, MKD
Oxypteryx graecatella (BOLD:ADL8101) n=1: MKD
Oxypteryx sp.2 (BOLD:ACS7859) n=2: CYP
Oxypteryx sp.3 (BOLD:ACR9491) n=2: ITA
Oxypteryx parahelotella (BOLD:ADR3424) n=1: ESP
Oxypteryx helotella (BOLD:AAP5437) n=5: ESP, GRE, ITA
Oxypteryx superbella (BOLD:AAF7565) n=8: FIN, GER, ITA
Oxypteryx ochricapilla (BOLD:AAP5737) n=6: AUT, ITA
Oxypteryx wilkella A (BOLD:AAF7554) n=23: AUT, FIN, GER, HUN, ITA, MKD, NOR
Oxypteryx wilkella B (BOLD:ACF6810) n=2: HUN, ITA
Oxypteryx baldizzonei A (BOLD:ABA2902) n=4: ITA

Oxypteryx baldizzonei B (BOLD:ACE7450) n=1: HRV
Oxypteryx baldizzonei C (BOLD:ACE2075) n=2: SVN
Oxypteryx mirusella (BOLD:ADS0158) n=2: FRA
Oxypteryx occidentella (BOLD:AAI5125) n=5: ITA
Oxypteryx libertinella A (BOLD:AAB5293) n=10: FRA, ITA
Oxypteryx libertinella B (BOLD:ADL5809) n=1: AUT
Oxypteryx libertinella C (BOLD:ACE5789) n=1: AUT
Oxypteryx libertinella D (BOLD:ACF5421) n=2: ITA
Oxypteryx libertinella E (BOLD:AAB5294) n=11: ITA
Oxypteryx libertinella F (BOLD:ADR5121) n=2: ITA
Oxypteryx libertinella G (BOLD:ABZ1714) n=1: AUT
Oxypteryx libertinella G (BOLD:ABZ1714) n=18: AUT, CHE, ITA

NJ tree 44
Thiotricha subocellea (BOLD:AAD7105) n=21: AUT, ESP, FIN, FRA, GER, ITA, NOR
Thiotricha coleella (missing) n=1: FRA
Thiotricha majorella (BOLD:AAO4372) n=7: AUT, ITA, MKD
Thiotricha wollastoni (BOLD:ADN0105) n=1: PRT
Palumbina guerinii (BOLD:AAV1433) n=4: ESP, GRE, ITA

NJ tree 45
Pseudotelphusa paripunctella (BOLD:AAD0741) n=33: AUT, CHE, DNK, FIN, GER, ITA, NOR
Pseudotelphusa occidentella (BOLD:ACA9489) n=1: ESP
Pseudotelphusa istrella (BOLD:ADC6829) n=2: ITA, ROU
Pseudotelphusa scalella (BOLD:AAM0076) n=11: AUT, GER, GRE, ITA
Pseudotelphusa tessella A (BOLD:AAM0070) n=6: AUT, GER
Pseudotelphusa tessella B (BOLD:ABY8568) n=7: AUT, GER, ITA
Xenolechia aethiops (BOLD:AAE1445) n=8: ESP, FRA, GRE
Xenolechia lindae (BOLD:AAE1445) n=1: GRE
Xenolechia pseudovulgella (BOLD:AAE1445) n=1: GRE

NJ tree 46
Ptocheuusa paupella A (BOLD:AAV2188) n=8: AUT, ESP, ITA
Ptocheuusa paupella B (BOLD:ACW1528) n=1: BGR
Ptocheuusa paupella C (BOLD:ACW2460) n=6: FRA, GRE, ITA
Ptocheuusa minimella (BOLD:ADM0559) n=1: GRE
Ptocheuusa inopella A (BOLD:ACW1360) n=1: SWE
Ptocheuusa inopella B (BOLD:ADF5122) n=2: ESP
Ptocheuusa inopella C (BOLD:AAV2187) n=3: ESP
Ptocheuusa sp.1 (BOLD:AAV7056) n=2: ESP
Ptocheuusa abnormella (BOLD:ABW9416) n=5: AUT, SVN

NJ tree 47
Exoteleia dodecella A1 (BOLD:AAA8390) n=9: AUT
Exoteleia dodecella A2 (BOLD:AAA8390) n=54: AUT, BGR, CHE, FIN, FRA, GER, ITA, NOR, SVN
Exoteleia succinctella (BOLD:AAD7714) n=14: AUT, GER, ITA
Stenolechiodes pseudogemmellus (BOLD:AAP7208) n=4: AUT, ITA
Stenolechiodes macrolepiellus (BOLD:AAJ5420) n=1: GRE
Coleotechnites piceaella (BOLD:AAA5953) n=2: AUT, GER
Parachronistis albiceps A (BOLD:ACA9804) n=5: AUT, GER, ITA

Parachronistis albiceps B (BOLD:AAD5736) n=2: AUT
Parachronistis albiceps C (BOLD:ACR4410) n=14: FIN, GER, ITA, NOR
Parastenolechia nigrinotella (BOLD:AAL3237) n=4: FRA, ITA, SVN
Schistophila laurocistella (BOLD:ADO2170) n=1: PRT
Stenolechia gemmella (BOLD:AAH9891) n=12: AUT, FIN, GER, ITA, NOR

NJ tree 48
Sattleria sophiae (BOLD:ACC4919) n=7: ITA
Sattleria triglavica (missing) n=1: SVN
Sattleria breviramus (BOLD:AAF1661) n=5: FRA
Sattleria graiaeella (BOLD:AAJ6133) n=3: ITA
Sattleria basistrigella (BOLD:AAM0044) n=6: CHE
Sattleria styriaca A (BOLD:ADZ0690) n=1: AUT
Sattleria styriaca B (BOLD:AAM0043) n=2: AUT
Sattleria haemusi (BOLD:ACC2506) n=3: BGR, MKD
Sattleria karsholti (BOLD:AAC5038) n=2: ITA
Sattleria dzieduszyckii (BOLD:AAV8914) n=1: ROU
Sattleria marguareisi (BOLD:ABY9734) n=3: FRA
Sattleria izoardi (BOLD:ABY9733) n=4: FRA
Sattleria cottiella (BOLD:AAE4405) n=5: ITA
Sattleria arcuata (BOLD:ADR2111) n=1: ESP
Sattleria pyrenaica A (BOLD:ADR7396) n=1: FRA
Sattleria pyrenaica B (BOLD:ADR7395) n=1: AND
Sattleria pyrenaica C (BOLD:ADR7394) n=1: ESP
Sattleria pyrenaica D (BOLD:ADR9382) n=1: ESP
Sattleria melaleucella (BOLD:AAC5037) n=16: AUT, CHE, FRA, ITA
Sattleria pyrenaica E (BOLD:AAC5037) n=2: AND, ESP

NJ tree 49
Scrobipalpa samadensis (BOLD:AAE9840) n=30: AUT, FIN, FRA, ITA, MKD, NOR
Scrobipalpa sp.1 (BOLD:AAV4547) n=4: ESP, HRV, ITA
Scrobipalpa halimoniella (BOLD:ACT0672) n=4: FRA, UKR
Scrobipalpa salinella (BOLD:AAF1193) n=8: ESP, FRA, ITA
Scrobipalpa salicorniae (BOLD:AAF1193) n=12: AUT, DNK, FIN, NOR
Scrobipalpa monochromella (BOLD:ACT1265) n=3: FRA, UKR
Scrobipalpa halymella (BOLD:AAV9005) n=1: FRA
Scrobipalpa stabilis {BOLD:AAV9005) n=1: ESP
Scrobipalpa thymelaeae (BOLD:ADL6008) n=2: BGR, ESP
Scrobipalpa sp.2 (BaOLD:ADF0070) n=1: GRE
Scrobipalpa instabilella (BOLD:AAH9719) n=6: DNK, FRA. GER
Scrobipalpa camphorosmella (missing) n=1: FRA
Scrobipalpa voltinella (BOLD:ADR2108) n=1: ESP
Scrobipalpa sp.3 (BOLD:ACT3383) n=1: UKR
Scrobipalpa kasyi (BOLD:ADL7116) n=2: BGR, GRE
Scrobipalpa bazae (BOLD:ADM0987) n=1: ESP
Scrobipalpa feralella (BOLD:AAH9731) n=5: AUT, ITA
Scrobipalpa pauperella (BOLD:AAF1201) n=27: AUT, FIN, GER, HRV, ITA
Scrobipalpa nitentella (BOLD:AAE9842) n=8: AUT, ESP, FIN, NOR
Scrobipalpa karadaghi (BOLD:ADR5475) n=1: RUS
Scrobipalpa tokari (BOLD:ADO1512) n=1: HRV
Scrobipalpa soffneri (BOLD:ACB0552) n=1: BGR

Scrobipalpa sp.4 (BOLD:ADL6932) n=1: FRA
Scrobipalpa dorsolutea (BOLD:ADN0121) n=1: RUS
Scrobipalpa sp.5 (BOLD:ADG5400) n=1: ESP
Scrobipalpa halonella (BOLD:ADJ3259) n=4: AUT
Scrobipalpa arenbergeri (BOLD:ACU4764) n=4: AUT, ITA, HRV
Scrobipalpa mercantourica (BOLD:ACU4764) n=1: FRA
Scrobipalpa montanella (BOLD:ADM1198) n=1: FRA
Scrobipalpa adaptata (BOLD:ABV8845) n=1: AUT
Scrobipalpa murinella A (BOLD:AAH9720) n=6: FIN, FRA, NOR
Scrobipalpa murinella B (BOLD:ACY8255) n=2: FIN, NOR
Scrobipalpa artemisiella A (BOLD:ADF1391) n=1: GER
Scrobipalpa artemisiella B (BOLD:AAE9838) n=29: AUT, ESP, FIN, FRA, GER, ITA, MKD
Scrobipalpa stangei (BOLD:AAE9838) n=4: FIN, NOR
Scrobipalpa suaedella (BOLD:ADN4306) n=1: ESP
Scrobipalpa algeriensis (BOLD:ADO1302) n=1: ESP
Scrobipalpa solitaria (BOLD:ADM1805) n=2: RUS
Scrobipalpa wiltshirei (BOLB:AAU2739) n=2: ESP
Scrobipalpa bradleyi (BOLD:AAU3155) n=3: ESP, FRA
Scrobipalpa phagnalella (BOLD:ADL5975) n=1: GRE
Scrobipalpa skulei (BOLD:ADR6061) n=1: HRV
Scrobipalpa caucasica (BOLD:ADL8263) n=1: GEO
Scrobipalpa superstes (BOLD:ACY4407) n=7: GRE, ITA
Scrobipalpa chrysanthemella (BOLD:ACA2604) n=11 : AUT, GER ITA
Scrobipalpa alterna (BOLD:ADR5476) n=1: RUS
Scrobipalpa lutea (BOLD:ADR5476) n=1: RUS
Scrobipalpa pulchra (BOLD:AAY7979) n=1: KZA
Scrobipalpa argenteonigra (BOLD:ADL6109) n=1: AZB
Scrobipalpa corleyi (BOLD:ABW6601) n=1: PRT
Scrobipalpa reiprichi A (BOLD:ACX8455) n=4: AUT, ITA
Scrobipalpa reiprichi B (BOLD:ACY6331) n=2: NOR
Scrobipalpa amseli (BOLD:ADL8424) n=1: ESP
Scrobipalpa hyssopi (BOlD:ADL8424) n= : FRA
Scrobipalpa vasconiella (BOLD:AAV7794) n=2: ESP
Scrobipalpa hendrikseni (BOLD:ADN4379) n=1: GRE
Scrobipalpa brahmiella (BOLD:ABA3381) n=2: FRA, MKD
Scrobipalpa suasella (BOLD:AAV7795) n=3: ESP, FRA
Scrobipalpa proclivella (BOLD:AAJ5816) n=7: AUT, FIN, GER, ITA
Scrobipalpa sp.6 (BOLD:ADL7117) n=1: ESP
Scrobipalpa gallicella (BOLD:ADG6801) n=2: FRA
Scrobipalpa jariorum (BOLD:ADG6980) n=1: BGR
Scrobipalpa hannemanni (BOLD:ADN4381) n=1: RUS
Scrobipalpa dorsoflava (BOLD:ADR6356) n=1: RUS
Scrobipalpa sp.7 (BOLD:ACT4605) n=1: UKR
Scrobipalpa bryophiloides A (BOLD:AAQ3427) n=2: RUS, UKR
Scrobipalpa bryophiloides B (BOLD:AAQ3428) n=1: RUS
Scrobipalpa heimi (missing) n=2: RUS
Scrobipalpa ustulatella (BOLD:ACL6706) n=1: RUS
Scrobipalpa deutschi (BOLD:ADN0123) n=1: RUS
Scrobipalpa rebeli (BOLD:ACL2449) n=2: ITA
Scrobipalpa disjectella (BOLD:ADL6933) n=3: ESP
Scrobipalpa fontanensis (BOLD:ADJ0760) n=1: FRA

Scrobipalpa postulatella (BOLD:ADL5759) n=4: ESP
Scrobipalpa grisea (missing) n=2: RUS
Scrobipalpa hyoscyamella (BOLD:ADN4380) n=1: AUT
Scrobipalpa suaedicola (BOLD:ADN1641} n=1: ESP
Scrobipalpa suaedivorella (BOLD:ADL7707) n=1: ESP
Scrobipalpa clintoni (BOLD:ABW1704) n=4: CHE, NOR
Scrobipalpa plesiopicta (BOLD:ADR5858) n=1: RUS
Scrobipalpa abstrusa (BOLD:AAW5451) n=1: RUS
Scrobipalpa costella (BOLD:AAF1186) n=1: DNK
Scrobipalpa atriplicella (BOLD:AAA9252) n=25: AUT, FIN, GER. ITA, NOR
Scrobipalpa ocellatella A (BOLD:AAG9138) n=2: ESP, GER
Scrobipalpa ocellatella B (BOLD:ADL5612) n=6: AUT, GER
Scrobipalpa indignella (missing) n=1: RUS
Scrobipalpa magnificella (BOLD:ADF7371) n=1: RUS
Scrobipalpa traganella (BOLD:ACS9476) n=1: ESP
Scrobipalpa bigoti (BOLD:AAV7801) n=2: ESP
Scrobipalpa occulta (BOLD:ADR5477) n=1: RUS
Scrobipalpa smithi (BOLD:ADM9516) n=1: ESP
Scrobipalpa oleksiyella (BOLD:ADN8885) n=1: RUS
Scrobipalpa obsoletella (BOLD:AAF1200) n=8: FIN, ITA, NOR
Scrobipalpa notata (BOLD:ADR6354) n=1: RUS
Scrobipalpa spumata (missing) n=1: RUS
Scrobipalpa acuminatella (BOLD:AAC1644) n=46: AUT, FIN, GER, ITA, MKD, NOR
Scrobipalpa divisella (BOLD:ADR3668) n=1: ESP
Scrobipalpa niveifacies (BOLD:ABW9337} n=1: PRT
Scrobipalpa halimifolia (BOLD:ACT1207) n=2: UKR
Scrobipalpa perinii (BOLD:ACI9032) n=3: ITA
Scrobipalpa griseoflava (BOLD:ACT2401) n=2: UKR
Scrobipalpa ergasima (BOLD:ABW0932) n=2: BGR, PRT
Scrobipalpa aptatella (BOLD:AAW5797) n=1: ARE
Scrobipalpa erichi (missing) n=1 : AUT
Scrobipalpa vicaria (BOLD:ACK7387) n=1: ESP
Scrobipalpa portosanctana (BOLD:ADL5974) n=1: HRV

NJ tree 50
Sophronia humerella (BOLD:AAF4746) n=15: AUT, FIN, GER, ITA
Sophronia santolinae (BOLD:ADR9214) n=1: ESP
Sophronia sp.1 (BOLD:ADF5021) n=1: BGR
Sophronia chilonella (BOLD:AAJ3742) n=5: AUT, BGR, GER, NOR
Sophronia sicariellus A (missing) n=1: GER
Sophronia sicariellus B (BOLD:AAJ3723) n=14: AUT, FIN, GER, ITA, MKD, NOR
Sophronia ascalis (BOLD:ACU4059) n=5: AUT, ITA
Sophronia finitimella (missing) n=1: GRE
Sophronia semicostella A (BOLD:AAF4748) n=5: AUT, FIN, NOR
Sophronia semicostella B (BOLD:AAF4747) n=14: AUT, ESP, FRA, GER, ITA
Sophronia illustrella (BOLD:ACZ2459) n=1: AUT
Sophronia consanguinella (BOLD:ADL7320) n=1: CZE
Sophronia gelidella (BOLD:AAF4739) n=3: FIN

NJ tree 51
Stomopteryx remissella A (BOLD:AAI0050) n=34: AUT, GER, ITA

Stomopteryx remissella B (BOLD:ACC3131) n=1: GRE
Stomopteryx remissella C (BOLD:AAO2655) n=7: ITA
Stomopteryx remissella D (BOLD:ADM8516) n=1: GRE
Stomopteryx remissella E (BOLD:AAU0792) n=2: ITA
Stomopteryx remissella F (BOLD:ACW1714) n=1: MKD
Stomopteryx remissella G (BOLD:AAI0051) n=5: CHE, ITA
Stomopteryx remissella H (BOLD:ADR2152) n=5: FRA, HRV
Stomopteryx alpinella (BOLD:ADF6169) n=1: FRA
Stomopteryx basalis (BOLD:ACW1398) n=2: BGR, HRV
Stomopteryx deverrae (BOLD:ADM5101) n=2: DZA, MAR
Stomopteryx mongolica (BOLD:ACB3380) n=1: RUS
Stomopteryx lineolella (BOLD:ACB3380) n=1: RUS
Stomopteryx nugatricella (BOLD:ACB3380) n=2: ESP
Stomopteryx flavoclavella A (BOLD:ADM5394) n=1 : MAR
Stomopteryx flavoclavella B (BOLD:ADL6782) n=2: ESP
Stomopteryx sp.1 (BOLD:ADM5270) n=1: GRE
Stomopteryx detersella (BOLD:ADL5887) n=1: MKD
Stomopteryx flavipalpella A (BOLD:AAV8359) n=4: FRA, ITA
Stomopteryx flavipalpella B (BOLD:ADM8466) n=2: ESP
Stomopteryx flavipalpella C (BOLD:ADM8515) n=1: ITA
Stomopteryx jeppeseni (BOLD:ADR8328) n=1: ESP
Stomopteryx lusitaniella (BOLD:ADN1459) n=1: GRE
Stomopteryx hungaricella (BOLD:ABW0260) n=5: FRA, ITA

NJ tree 52
Teleiodes brevivalvus (BOLD:AAE9855) n=2: ITA
Teleiodes italica (BOLD:AAE9855) n=7: ITA
Teleiodes vulgella (BOLD:AAE9855) n=28: AUT, FIN, GER, NOR
Teleiodes saltuum A (BOLD:AAF1120) n=8: AUT, DNK, GER, SWE
Teleiodes saltuum A (BOLD:AAF1120) n=4: AUT, ITA
Teleiodes saltuum B (BOLD:ABZ0224) n=5: AUT, GER, ITA
Teleiodes kaitilai (BOLD:ACB0610) n=1: RUS
Teleiodes traugotti (missing) n=1: ESP
Teleiodes wagae (BOLD:AAV7088) n=12: AUT, FIN, GER, HRV, SVN
Teleiodes albiluculella (BOLD:ADO2279) n=1: GRE
Teleiodes flavimaculella A (BOLD:AAH9847) n=9: AUT, FIN, GER, HRV
Teleiodes flavimaculella B (BOLD:AAP5747) n=1: AUT
Teleiodes flavimaculella C (BOLD:AAD2633) n=7: AUT, GER
Teleiodes luculella A (BOLD:ADM4854) n=2: CYP
Teleiodes luculella B (BOLD:ACU4420) n=2: AUT, GER
Teleiodes luculella C (BOLD:AAD2632) n=26: AUT, ESP, FIN, GER, ITA, NOR
Schneidereria pistaciella (BOLD:ADM2090) n=1: HRV

NJ tree 53
Teleiopsis rosalbella (BOLD:AAB6930) n=8: GER, ITA
Teleiopsis albifemorella (BOLD:AAB6930) n=15: AUT, ITA, SVN
Teleiopsis albifemorella (BOLD:AAB6930) n=13: AUT, GER
Teleiopsis paulheberti A (BOLD:ACE6399) n=15: ITA
Teleiopsis paulheberti B (BOLD:ABZ5927) n=4: FRA
Teleiopsis diffinis A (BOLD:ACE4927) n=1: GER
Teleiopsis paulheberti C (BOLD:ACE4927) n=1: FRA

Teleiopsis bagriotella A (BOLD:ACE4927) n=1: AUT
Teleiopsis diffinis A (BOLD:ACE4927) n=8: AND, AUT, ESP, ITA
Teleiopsis diffinis B (BOLD:ACE6105) n=21: AUT, FIN, ITA, NOR, ROU
Teleiopsis bagriotella B (BOLD:ACE6105) n=13: AUT, ITA, MKD
Teleiopsis bagriotella B (BOLD:ACE6105) n=4: ITA
Teleiopsis laetitiae (BOLD:ABZ5926) n=10: AUT, CHE, GRE, ITA, MKD
Teleiopsis lunariella (BOLD:ADL7992) n=1 : ESP
Teleiopsis latisacculus (missing): n=1: GRE
Teleiopsis terebinthinella (BOLD:AAK0911) n=2: ITA

NJ tree 1

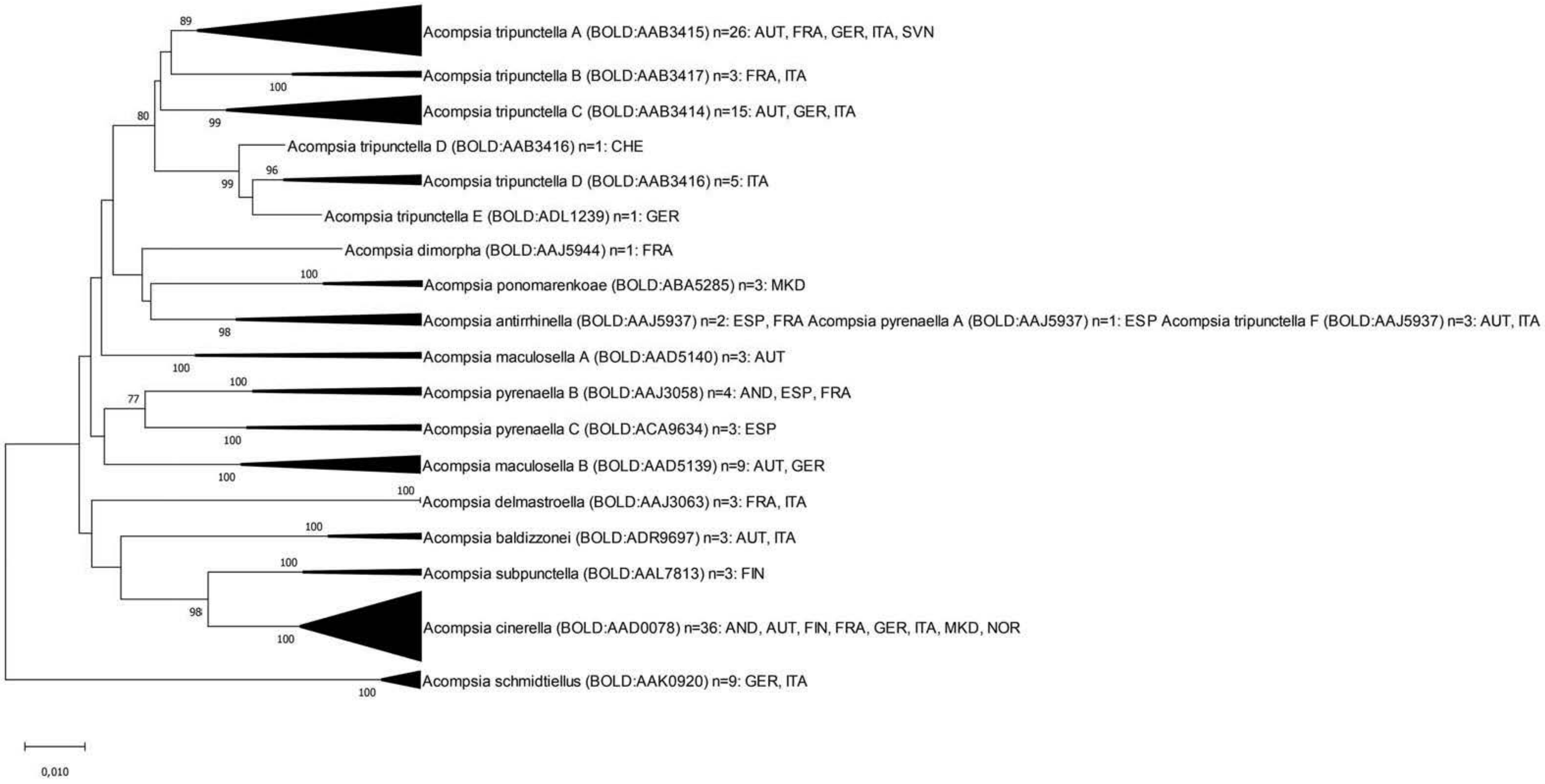

Acompsia tripunctella A (BOLD:AAB3415) n=26: AUT, FRA, GER, ITA, SVN
Acompsia tripunctella B (BOLD:AAB3417) n=3: FRA, ITA
Acompsia tripunctella C (BOLD:AAB3414) n=15: AUT, GER, ITA
Acompsia tripunctella D (BOLD:AAB3416) n=1: CHE
Acompsia tripunctella D (BOLD:AAB3416) n=5: ITA
Acompsia tripunctella E (BOLD:ADL1239) n=1: GER
Acompsia dimorpha (BOLD:AAJ5944) n=1: FRA
Acompsia ponomarenkoae (BOLD:ABA5285) n=3: MKD
Acompsia antirrhinella (BOLD:AAJ5937) n=2: ESP, FRA Acompsia pyrenaella A (BOLD:AAJ5937) n=1: ESP Acompsia tripunctella F (BOLD:AAJ5937) n=3: AUT, ITA
Acompsia maculosella A (BOLD:AAD5140) n=3: AUT
Acompsia pyrenaella B (BOLD:AAJ3058) n=4: AND, ESP, FRA
Acompsia pyrenaella C (BOLD:ACA9634) n=3: ESP
Acompsia maculosella B (BOLD:AAD5139) n=9: AUT, GER
Acompsia delmastroella (BOLD:AAJ3063) n=3: FRA, ITA
Acompsia baldizzonei (BOLD:ADR9697) n=3: AUT, ITA
Acompsia subpunctella (BOLD:AAL7813) n=3: FIN
Acompsia cinerella (BOLD:AAD0078) n=36: AND, AUT, FIN, FRA, GER, ITA, MKD, NOR
Acompsia schmidtiellus (BOLD:AAK0920) n=9: GER, ITA
89
100
99
80
96
99
100
98
100
100
77
100
100
100
100
100
98
100
100
0,010

NJ tree2

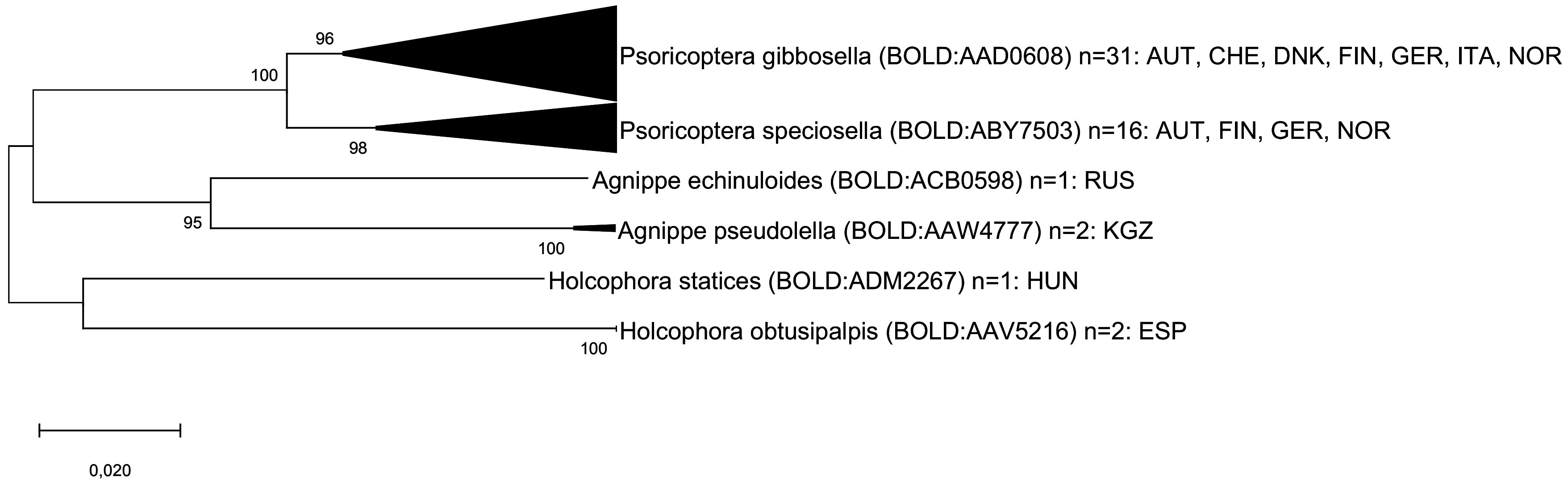

Psoricoptera gibbosella (BOLD:AAD0608) n=31: AUT, CHE, DNK, FIN, GER, ITA, NOR
Psoricoptera speciosella (BOLD:ABY7503) n=16: AUT, FIN, GER, NOR
Agnippe echinuloides (BOLD:ACB0598) n=1: RUS
Agnippe pseudolella (BOLD:AAW4777) n=2: KGZ
Holcophora statices (BOLD:ADM2267) n=1: HUN
Holcophora obtusipalpis (BOLD:AAV5216) n=2: ESP
96
100
98
95
100
100
0,020

NJ tree 3

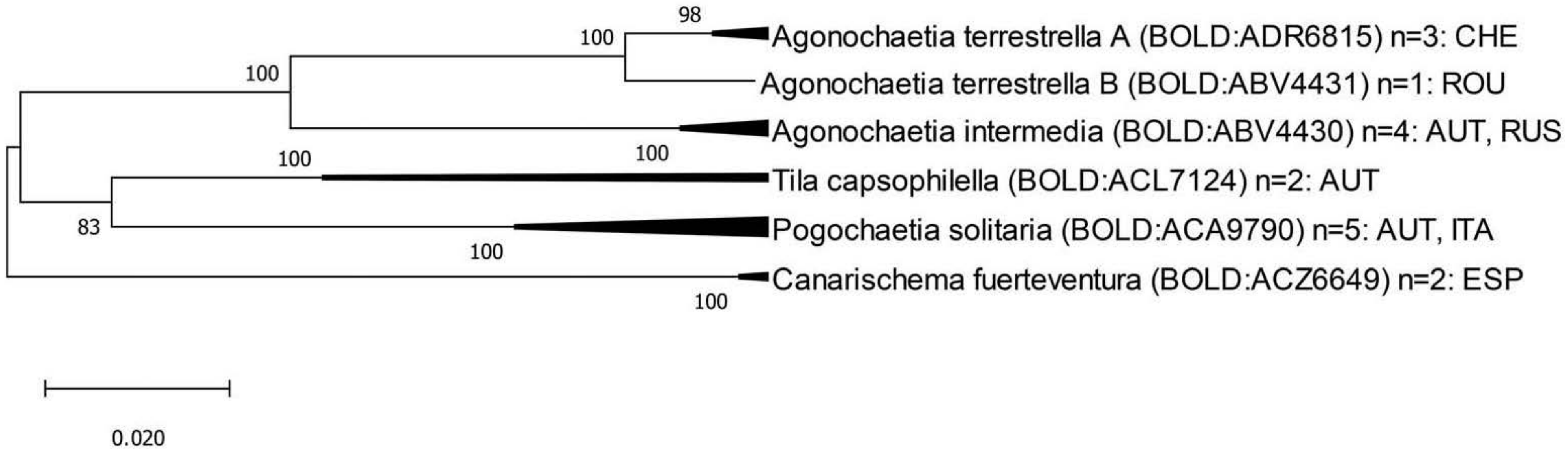
98
100
100
Agonochaetia terrestrella A (BOLD:ADR6815) n=3: CHE
Agonochaetia terrestrella B (BOLD:ABV4431) n=1: ROU
Agonochaetia intermedia (BOLD:ABV4430) n=4: AUT, RUS
100
100
Tila capsophilella (BOLD:ACL7124) n=2: AUT
83
Pogochaetia solitaria (BOLD:ACA9790) n=5: AUT, ITA
100
Canarischema fuerteventura (BOLD:ACZ6649) n=2: ESP
100
0.020

NJ tree 4

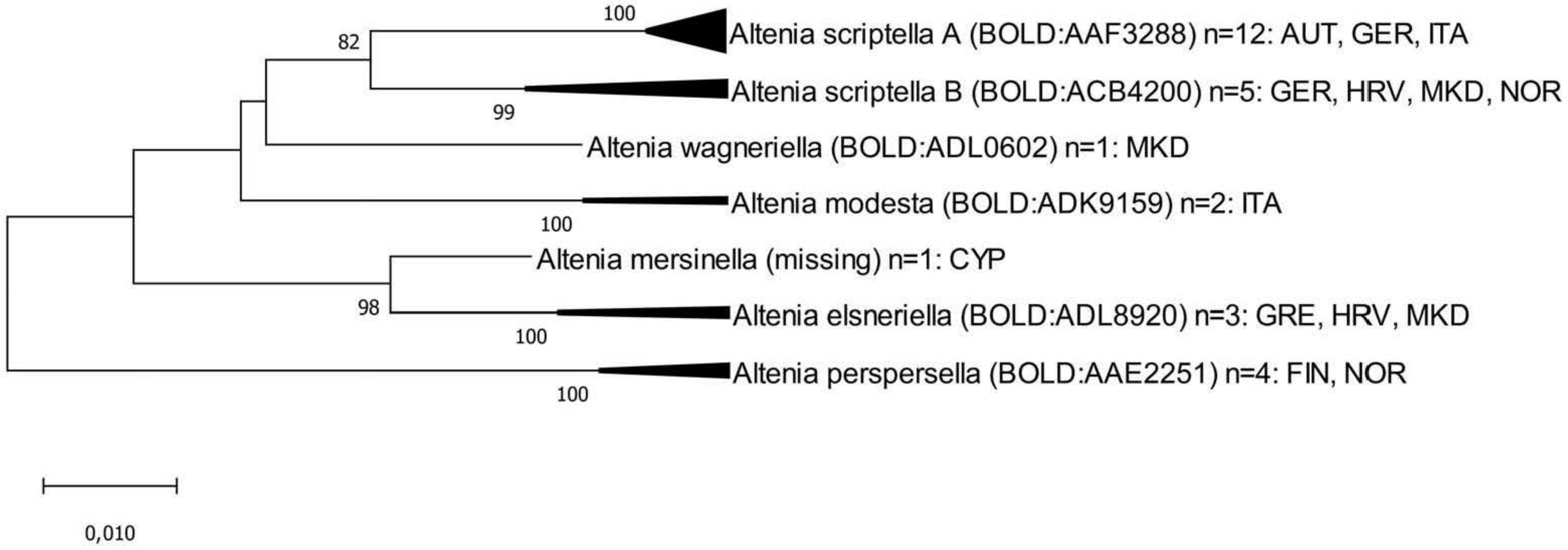

100
82
Altenia scriptella A (BOLD:AAF3288) n=12: AUT, GER, ITA
Altenia scriptella B (BOLD:ACB4200) n=5: GER, HRV, MKD, NOR
99
Altenia wagneriella (BOLD:ADL0602) n=1: MKD
Altenia modesta (BOLD:ADK9159) n=2: ITA
100
Altenia mersinella (missing) n=1: CYP
98
Altenia elsneriella (BOLD:ADL8920) n=3: GRE, HRV, MKD
100
Altenia perspersella (BOLD:AAE2251) n=4: FIN, NOR
100
0,010

NJ tree 5

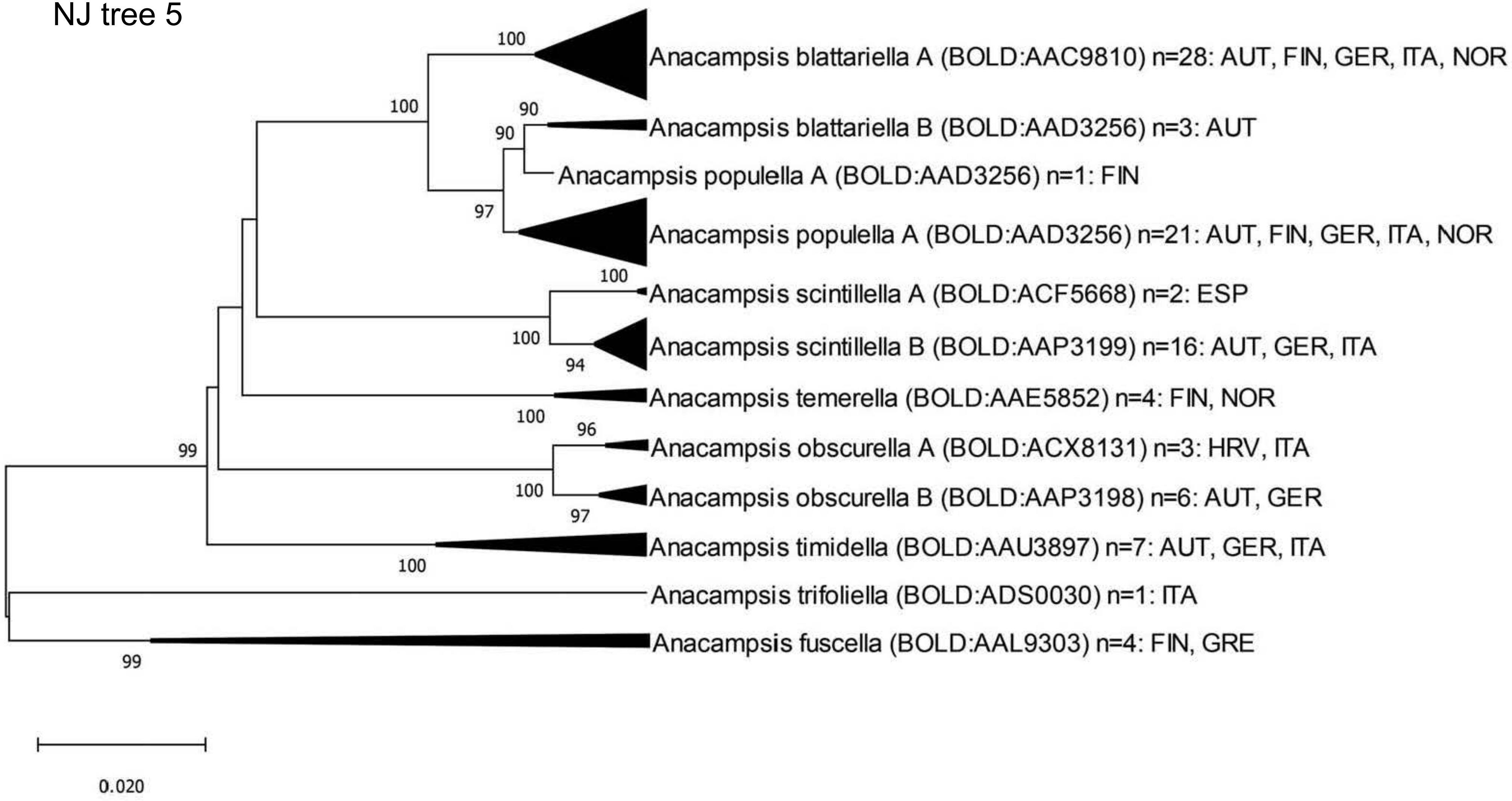

100
Anacampsis blattariella A (BOLD:AAC9810) n=28: AUT, FIN, GER, ITA, NOR
100
90
90
Anacampsis blattariella B (BOLD:AAD3256) n=3: AUT
Anacampsis populella A (BOLD:AAD3256) n=1: FIN
97
Anacampsis populella A (BOLD:AAD3256) n=21: AUT, FIN, GER, ITA, NOR
100
Anacampsis scintillella A (BOLD:ACF5668) n=2: ESP
100
94
Anacampsis scintillella B (BOLD:AAP3199) n=16: AUT, GER, ITA
Anacampsis temerella (BOLD:AAE5852) n=4: FIN, NOR
100
96
99
Anacampsis obscurella A (BOLD:ACX8131) n=3: HRV, ITA
100
Anacampsis obscurella B (BOLD:AAP3198) n=6: AUT, GER
97
Anacampsis timidella (BOLD:AAU3897) n=7: AUT, GER, ITA
100
Anacampsis trifoliella (BOLD:ADS0030) n=1: ITA
Anacampsis fuscella (BOLD:AAL9303) n=4: FIN, GRE
99
0.020

NJ tree 6

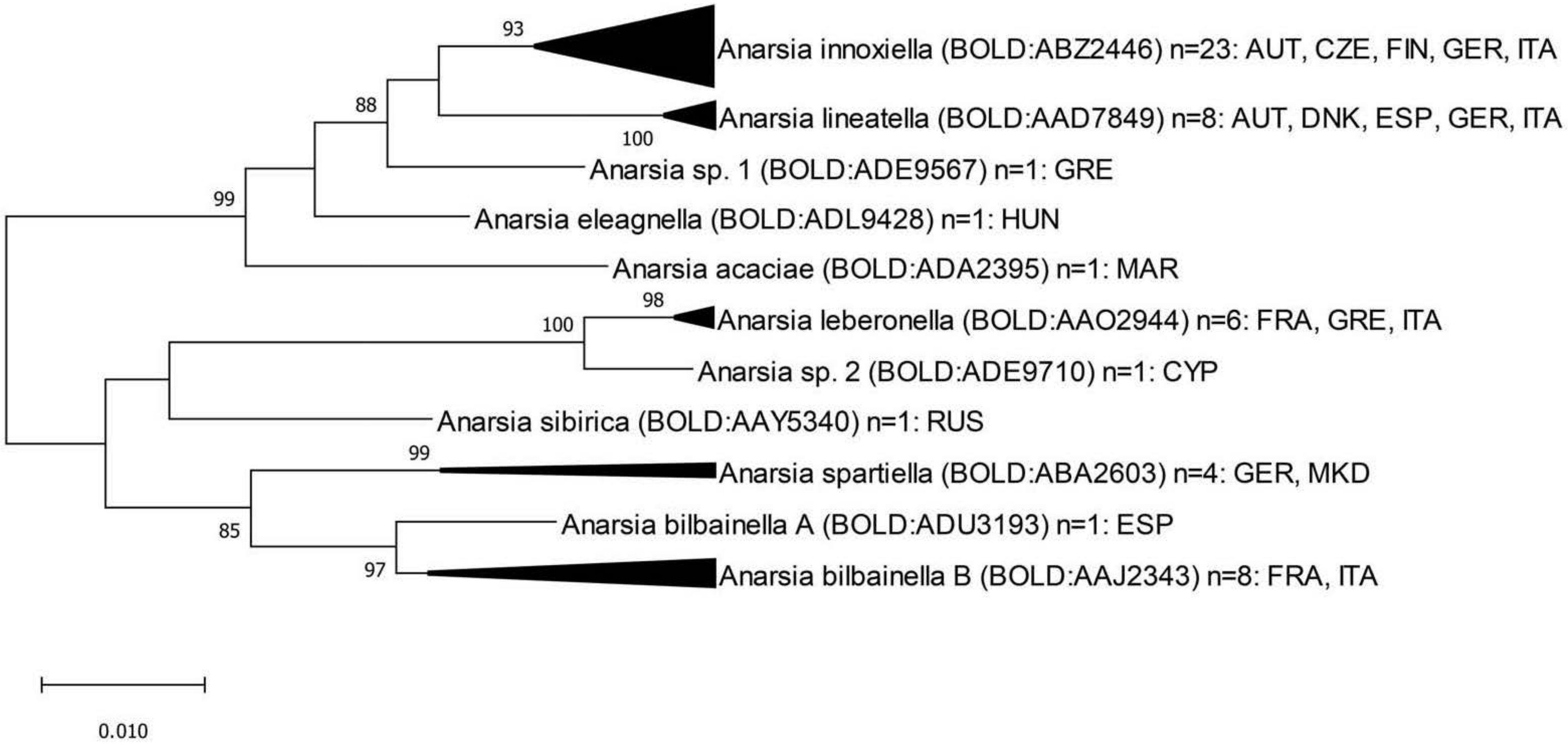
93
Anarsia innoxiella (BOLD:ABZ2446) n=23: AUT, CZE, FIN, GER, ITA
88
Anarsia lineatella (BOLD:AAD7849) n=8: AUT, DNK, ESP, GER, ITA
100
Anarsia sp. 1 (BOLD:ADE9567) n=1: GRE
99
Anarsia eleagnella (BOLD:ADL9428) n=1: HUN
Anarsia acaciae (BOLD:ADA2395) n=1: MAR
98
100
Anarsia leberonella (BOLD:AAO2944) n=6: FRA, GRE, ITA
Anarsia sp. 2 (BOLD:ADE9710) n=1: CYP
Anarsia sibirica (BOLD:AAY5340) n=1: RUS
99
Anarsia spartiella (BOLD:ABA2603) n=4: GER, MKD
Anarsia bilbainella A (BOLD:ADU3193) n=1: ESP
85
97
Anarsia bilbainella B (BOLD:AAJ2343) n=8: FRA, ITA
0.010

NJ tree 7

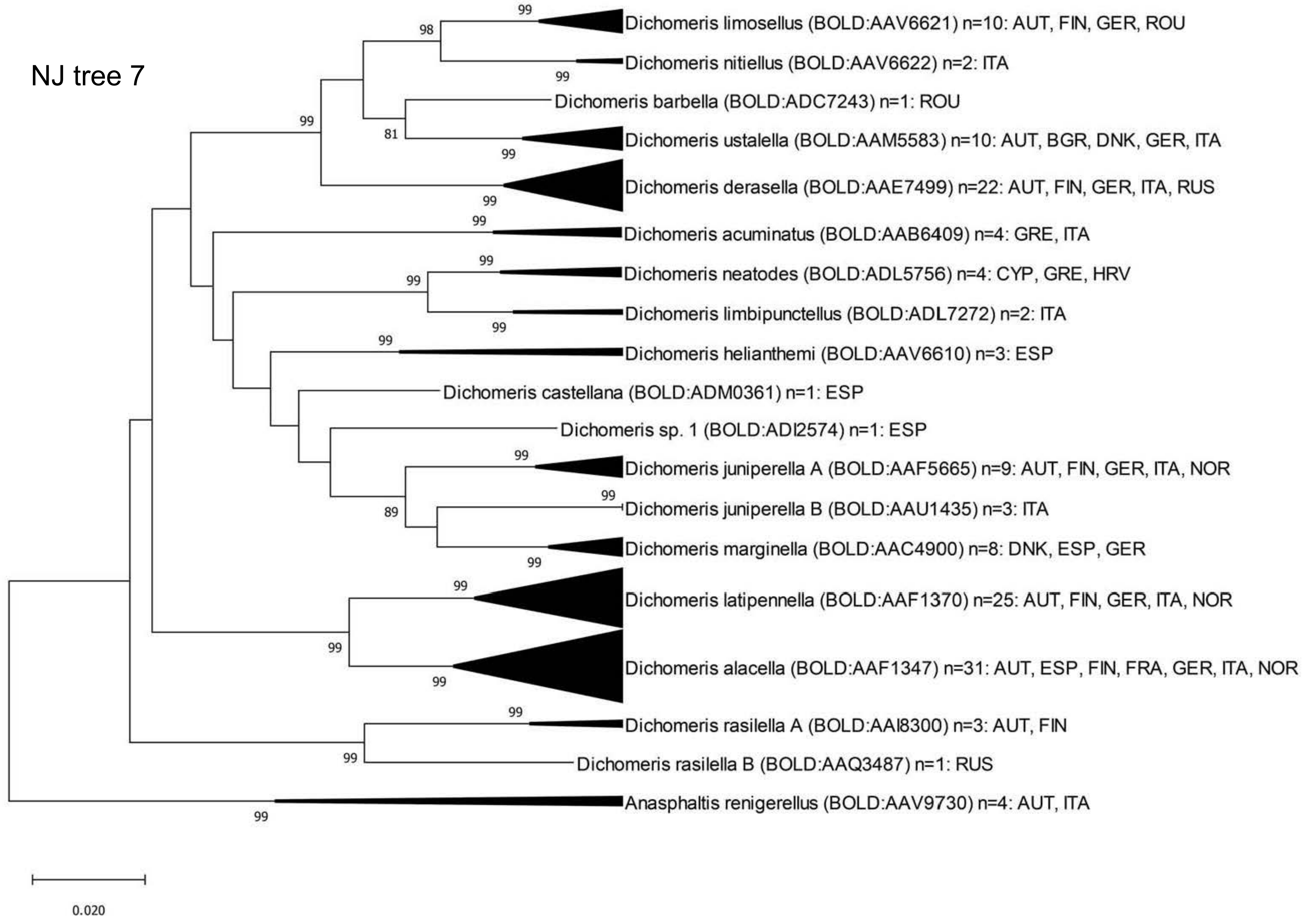

Dichomeris limosellus (BOLD:AAV6621) n=10: AUT, FIN, GER, ROU
Dichomeris nitiellus (BOLD:AAV6622) n=2: ITA
Dichomeris barbella (BOLD:ADC7243) n=1: ROU
Dichomeris ustalella (BOLD:AAM5583) n=10: AUT, BGR, DNK, GER, ITA
Dichomeris derasella (BOLD:AAE7499) n=22: AUT, FIN, GER, ITA, RUS
Dichomeris acuminatus (BOLD:AAB6409) n=4: GRE, ITA
Dichomeris neatodes (BOLD:ADL5756) n=4: CYP, GRE, HRV
Dichomeris limbipunctellus (BOLD:ADL7272) n=2: ITA
Dichomeris helianthemi (BOLD:AAV6610) n=3: ESP
Dichomeris castellana (BOLD:ADM0361) n=1: ESP
Dichomeris sp. 1 (BOLD:ADI2574) n=1: ESP
Dichomeris juniperella A (BOLD:AAF5665) n=9: AUT, FIN, GER, ITA, NOR
Dichomeris juniperella B (BOLD:AAU1435) n=3: ITA
Dichomeris marginella (BOLD:AAC4900) n=8: DNK, ESP, GER
Dichomeris latipennella (BOLD:AAF1370) n=25: AUT, FIN, GER, ITA, NOR
Dichomeris alacella (BOLD:AAF1347) n=31: AUT, ESP, FIN, FRA, GER, ITA, NOR
Dichomeris rasilella A (BOLD:AAI8300) n=3: AUT, FIN
Dichomeris rasilella B (BOLD:AAQ3487) n=1: RUS
Anasphaltis renigerellus (BOLD:AAV9730) n=4: AUT, ITA
99
98
99
99
81
99
99
99
99
99
99
99
99
99
89
99
99
99
99
99
99
99
99
99
0.020

NJ tree 8

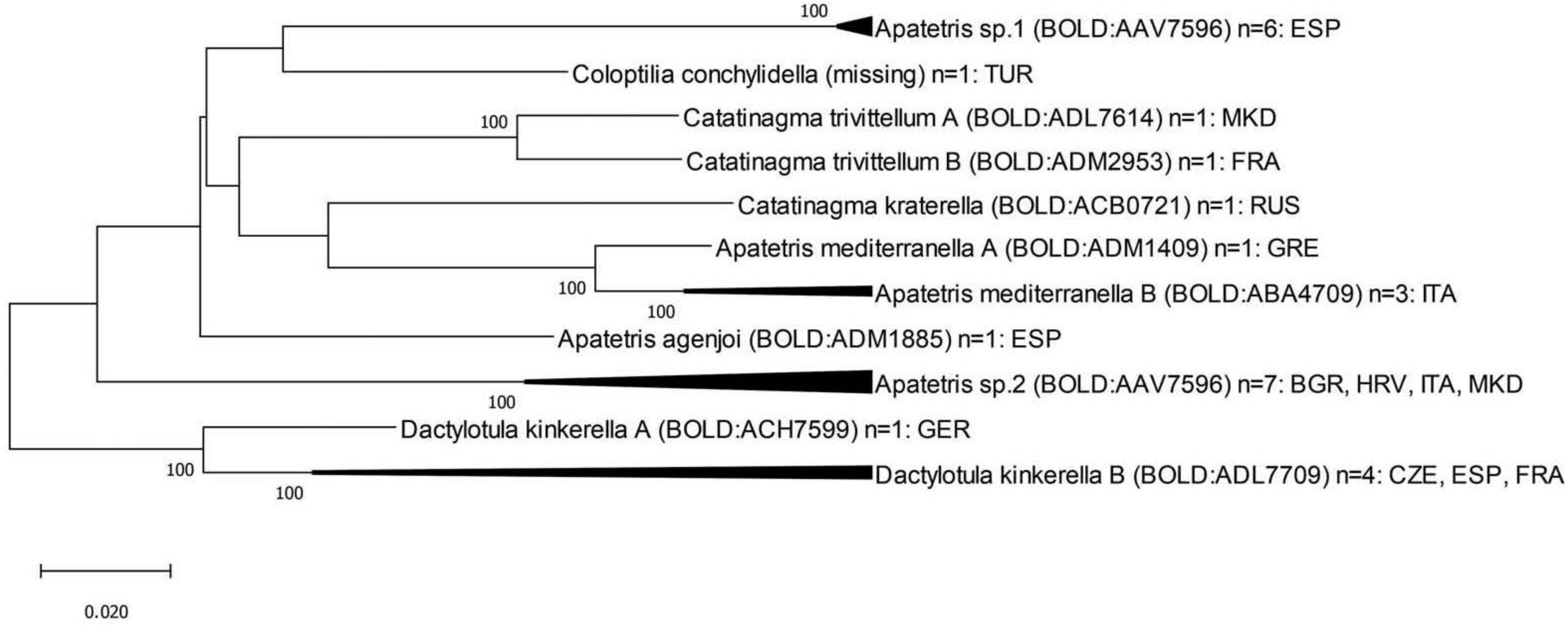
100
Apatetris sp.1 (BOLD:AAV7596) n=6: ESP
Coloptilia conchylidella (missing) n=1: TUR
100
Catatinagma trivittellum A (BOLD:ADL7614) n=1: MKD
Catatinagma trivittellum B (BOLD:ADM2953) n=1: FRA
Catatinagma kraterella (BOLD:ACB0721) n=1: RUS
Apatetris mediterranella A (BOLD:ADM1409) n=1: GRE
100
Apatetris mediterranella B (BOLD:ABA4709) n=3: ITA
100
Apatetris agenjoi (BOLD:ADM1885) n=1: ESP
Apatetris sp.2 (BOLD:AAV7596) n=7: BGR, HRV, ITA, MKD
100
Dactylotula kinkerella A (BOLD:ACH7599) n=1: GER
100
Dactylotula kinkerella B (BOLD:ADL7709) n=4: CZE, ESP, FRA
100
0.020

NJ tree 9

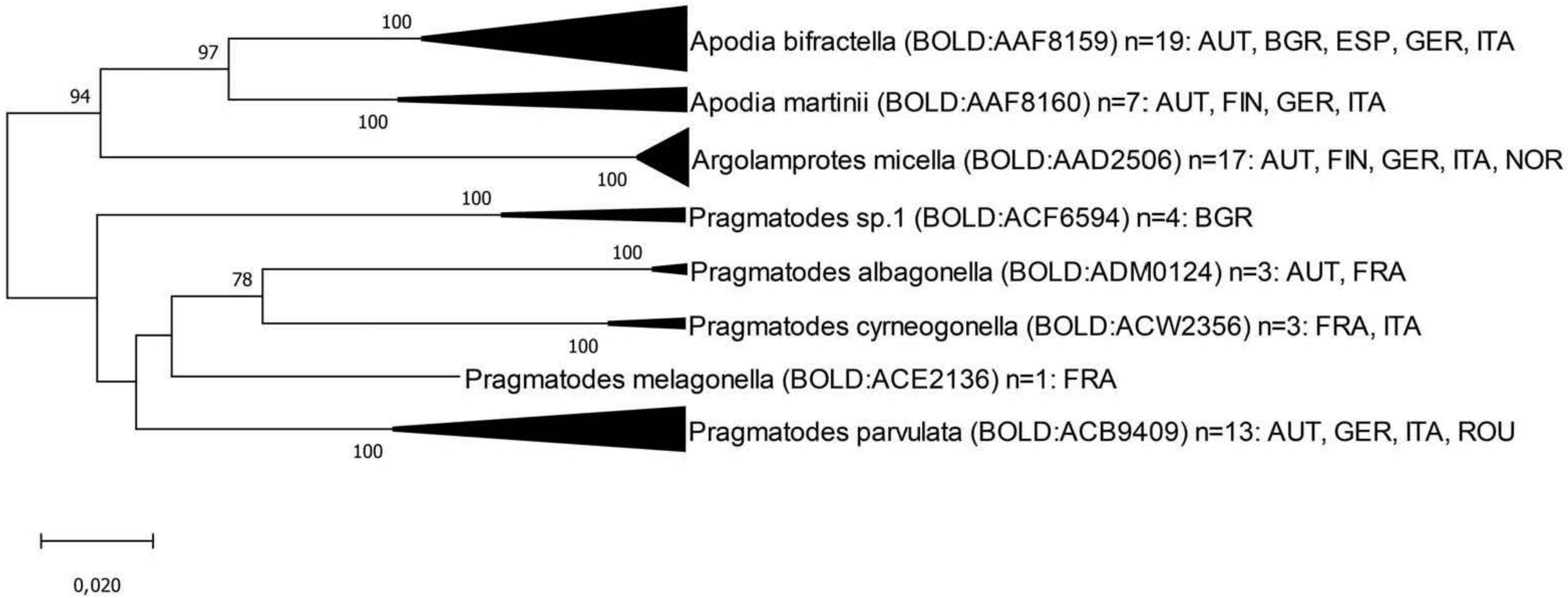

100
97
94
Apodia bifractella (BOLD:AAF8159) n=19: AUT, BGR, ESP, GER, ITA
Apodia martinii (BOLD:AAF8160) n=7: AUT, FIN, GER, ITA
100
Argolamprotes micella (BOLD:AAD2506) n=17: AUT, FIN, GER, ITA, NOR
100
100
Pragmatodes sp.1 (BOLD:ACF6594) n=4: BGR
100
78
Pragmatodes albagonella (BOLD:ADM0124) n=3: AUT, FRA
Pragmatodes cyrneogonella (BOLD:ACW2356) n=3: FRA, ITA
100
Pragmatodes melagonella (BOLD:ACE2136) n=1: FRA
Pragmatodes parvulata (BOLD:ACB9409) n=13: AUT, GER, ITA, ROU
100
0,020

NJ tree 10

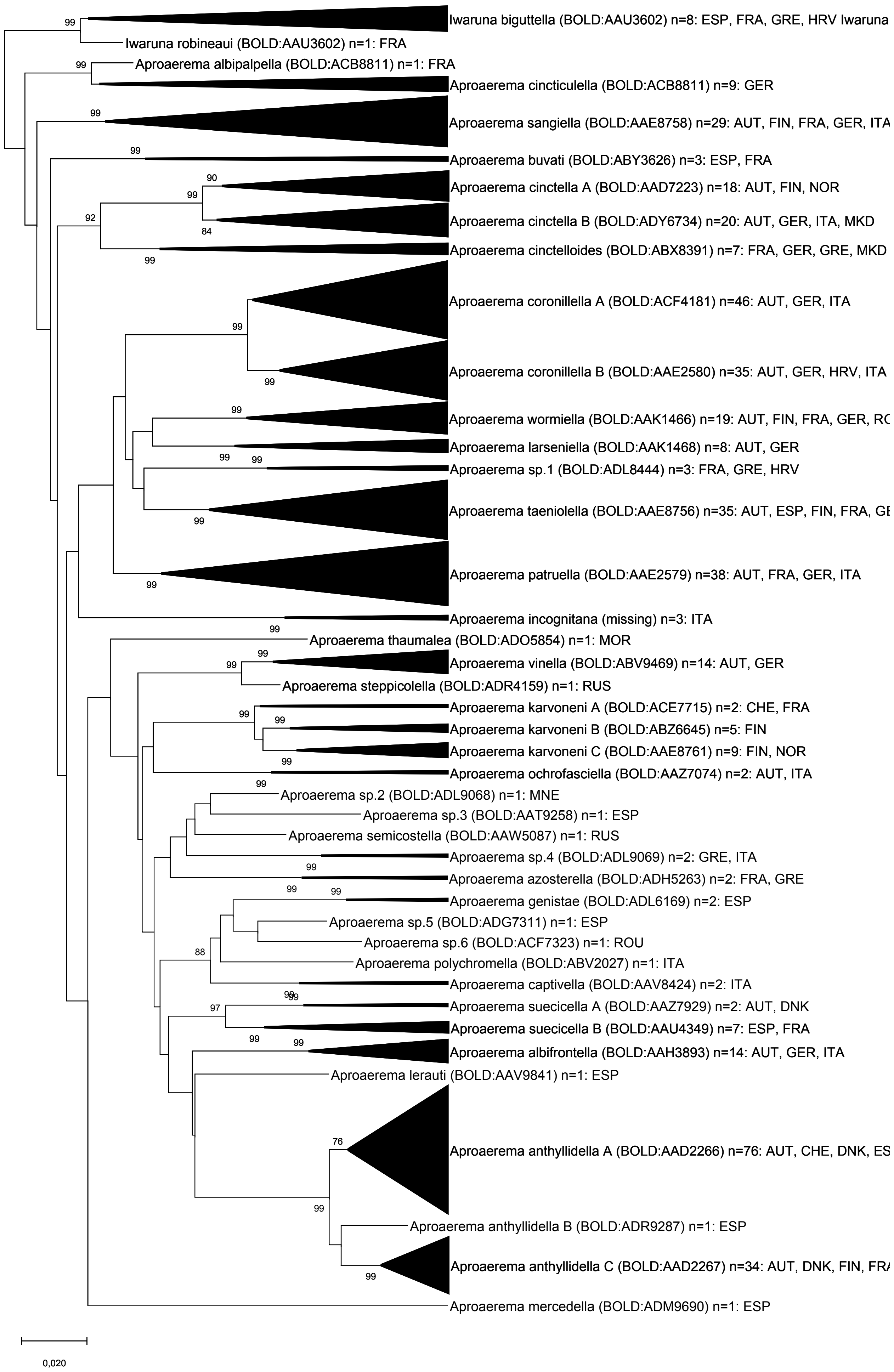

Iwaruna biguttella (BOLD:AAU3602) n=8: ESP, FRA, GRE, HRV Iwaruna
Iwaruna robineaui (BOLD:AAU3602) n=1: FRA
Aproaerema albipalpella (BOLD:ACB8811) n=1: FRA
Aproaerema cincticulella (BOLD:ACB8811) n=9: GER
Aproaerema sangiella (BOLD:AAE8758) n=29: AUT, FIN, FRA, GER, ITA
Aproaerema buvati (BOLD:ABY3626) n=3: ESP, FRA
Aproaerema cinctella A (BOLD:AAD7223) n=18: AUT, FIN, NOR
Aproaerema cinctella B (BOLD:ADY6734) n=20: AUT, GER, ITA, MKD
Aproaerema cinctelloides (BOLD:ABX8391) n=7: FRA, GER, GRE, MKD
Aproaerema coronillella A (BOLD:ACF4181) n=46: AUT, GER, ITA
Aproaerema coronillella B (BOLD:AAE2580) n=35: AUT, GER, HRV, ITA
Aproaerema wormiella (BOLD:AAK1466) n=19: AUT, FIN, FRA, GER, RC
Aproaerema larseniella (BOLD:AAK1468) n=8: AUT, GER
Aproaerema sp.1 (BOLD:ADL8444) n=3: FRA, GRE, HRV
Aproaerema taeniolella (BOLD:AAE8756) n=35: AUT, ESP, FIN, FRA, GE
Aproaerema patruella (BOLD:AAE2579) n=38: AUT, FRA, GER, ITA
Aproaerema incognitana (missing) n=3: ITA
Aproaerema thaumalea (BOLD:ADO5854) n=1: MOR
Aproaerema vinella (BOLD:ABV9469) n=14: AUT, GER
Aproaerema steppicolella (BOLD:ADR4159) n=1: RUS
Aproaerema karvoneni A (BOLD:ACE7715) n=2: CHE, FRA
Aproaerema karvoneni B (BOLD:ABZ6645) n=5: FIN
Aproaerema karvoneni C (BOLD:AAE8761) n=9: FIN, NOR
Aproaerema ochrofasciella (BOLD:AAZ7074) n=2: AUT, ITA
Aproaerema sp.2 (BOLD:ADL9068) n=1: MNE
Aproaerema sp.3 (BOLD:AAT9258) n=1: ESP
Aproaerema semicostella (BOLD:AAW5087) n=1: RUS
Aproaerema sp.4 (BOLD:ADL9069) n=2: GRE, ITA
Aproaerema azosterella (BOLD:ADH5263) n=2: FRA, GRE
Aproaerema genistae (BOLD:ADL6169) n=2: ESP
Aproaerema sp.5 (BOLD:ADG7311) n=1: ESP
Aproaerema sp.6 (BOLD:ACF7323) n=1: ROU
Aproaerema polychromella (BOLD:ABV2027) n=1: ITA
Aproaerema captivella (BOLD:AAV8424) n=2: ITA
Aproaerema suecicella A (BOLD:AAZ7929) n=2: AUT, DNK
Aproaerema suecicella B (BOLD:AAU4349) n=7: ESP, FRA
Aproaerema albifrontella (BOLD:AAH3893) n=14: AUT, GER, ITA
Aproaerema lerauti (BOLD:AAV9841) n=1: ESP
Aproaerema anthyllidella A (BOLD:AAD2266) n=76: AUT, CHE, DNK, ES
Aproaerema anthyllidella B (BOLD:ADR9287) n=1: ESP
Aproaerema anthyllidella C (BOLD:AAD2267) n=34: AUT, DNK, FIN, FR
Aproaerema mercedella (BOLD:ADM9690) n=1: ESP
0,020

NJ tree 11

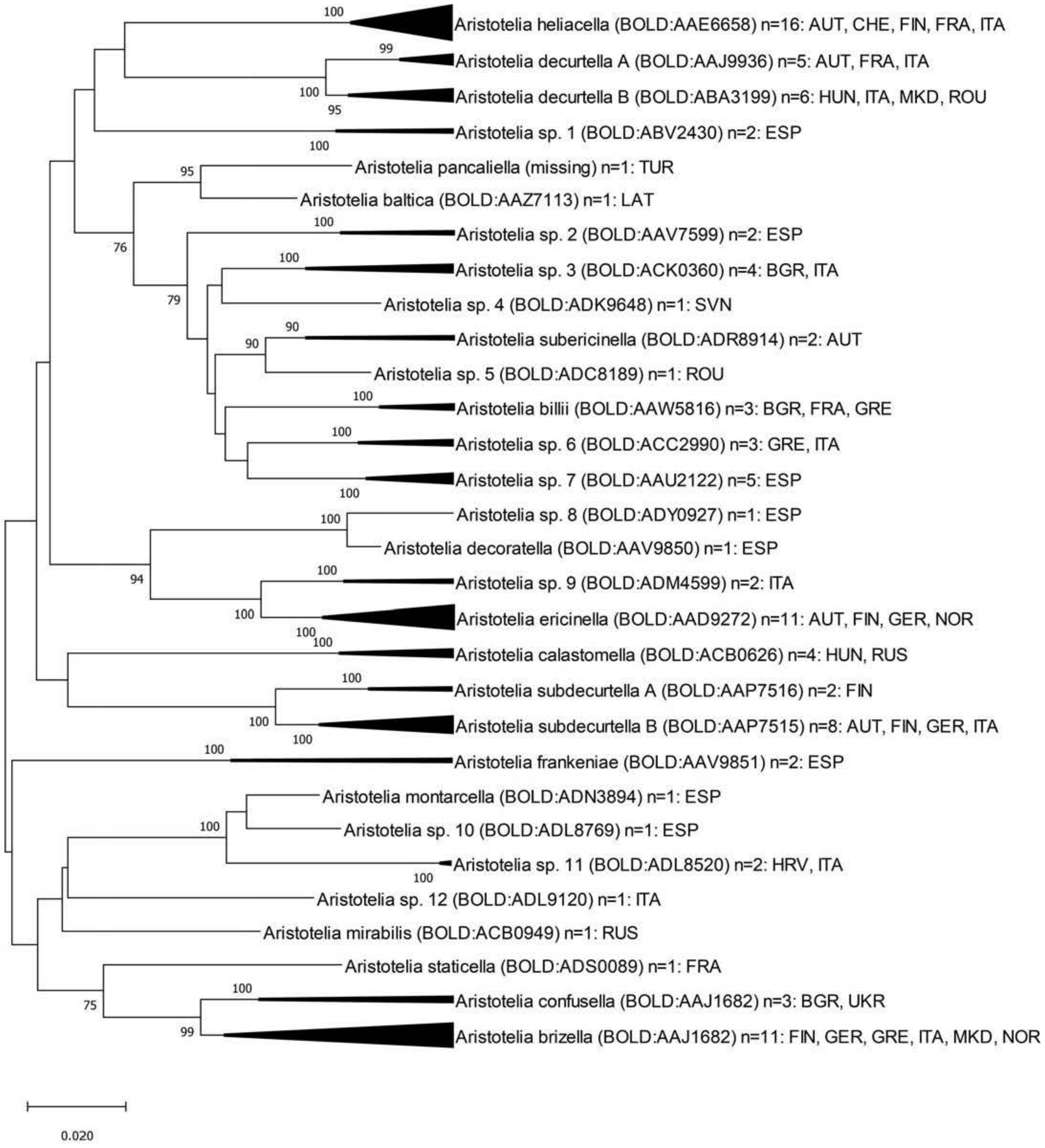

Aristotelia heliacella (BOLD:AAE6658) n=16: AUT, CHE, FIN, FRA, ITA
Aristotelia decurtella A (BOLD:AAJ9936) n=5: AUT, FRA, ITA
Aristotelia decurtella B (BOLD:ABA3199) n=6: HUN, ITA, MKD, ROU
Aristotelia sp. 1 (BOLD:ABV2430) n=2: ESP
Aristotelia pancaliella (missing) n=1: TUR
Aristotelia baltica (BOLD:AAZ7113) n=1: LAT
Aristotelia sp. 2 (BOLD:AAV7599) n=2: ESP
Aristotelia sp. 3 (BOLD:ACK0360) n=4: BGR, ITA
Aristotelia sp. 4 (BOLD:ADK9648) n=1: SVN
Aristotelia subericinella (BOLD:ADR8914) n=2: AUT
Aristotelia sp. 5 (BOLD:ADC8189) n=1: ROU
Aristotelia billii (BOLD:AAW5816) n=3: BGR, FRA, GRE
Aristotelia sp. 6 (BOLD:ACC2990) n=3: GRE, ITA
Aristotelia sp. 7 (BOLD:AAU2122) n=5: ESP
Aristotelia sp. 8 (BOLD:ADY0927) n=1: ESP
Aristotelia decoratella (BOLD:AAV9850) n=1: ESP
Aristotelia sp. 9 (BOLD:ADM4599) n=2: ITA
Aristotelia ericinella (BOLD:AAD9272) n=11: AUT, FIN, GER, NOR
Aristotelia calastomella (BOLD:ACB0626) n=4: HUN, RUS
Aristotelia subdecurtella A (BOLD:AAP7516) n=2: FIN
Aristotelia subdecurtella B (BOLD:AAP7515) n=8: AUT, FIN, GER, ITA
Aristotelia frankeniae (BOLD:AAV9851) n=2: ESP
Aristotelia montarcella (BOLD:ADN3894) n=1: ESP
Aristotelia sp. 10 (BOLD:ADL8769) n=1: ESP
Aristotelia sp. 11 (BOLD:ADL8520) n=2: HRV, ITA
Aristotelia sp. 12 (BOLD:ADL9120) n=1: ITA
Aristotelia mirabilis (BOLD:ACB0949) n=1: RUS
Aristotelia staticella (BOLD:ADS0089) n=1: FRA
Aristotelia confusella (BOLD:AAJ1682) n=3: BGR, UKR
Aristotelia brizella (BOLD:AAJ1682) n=11: FIN, GER, GRE, ITA, MKD, NOR
0.020

NJ tree 12

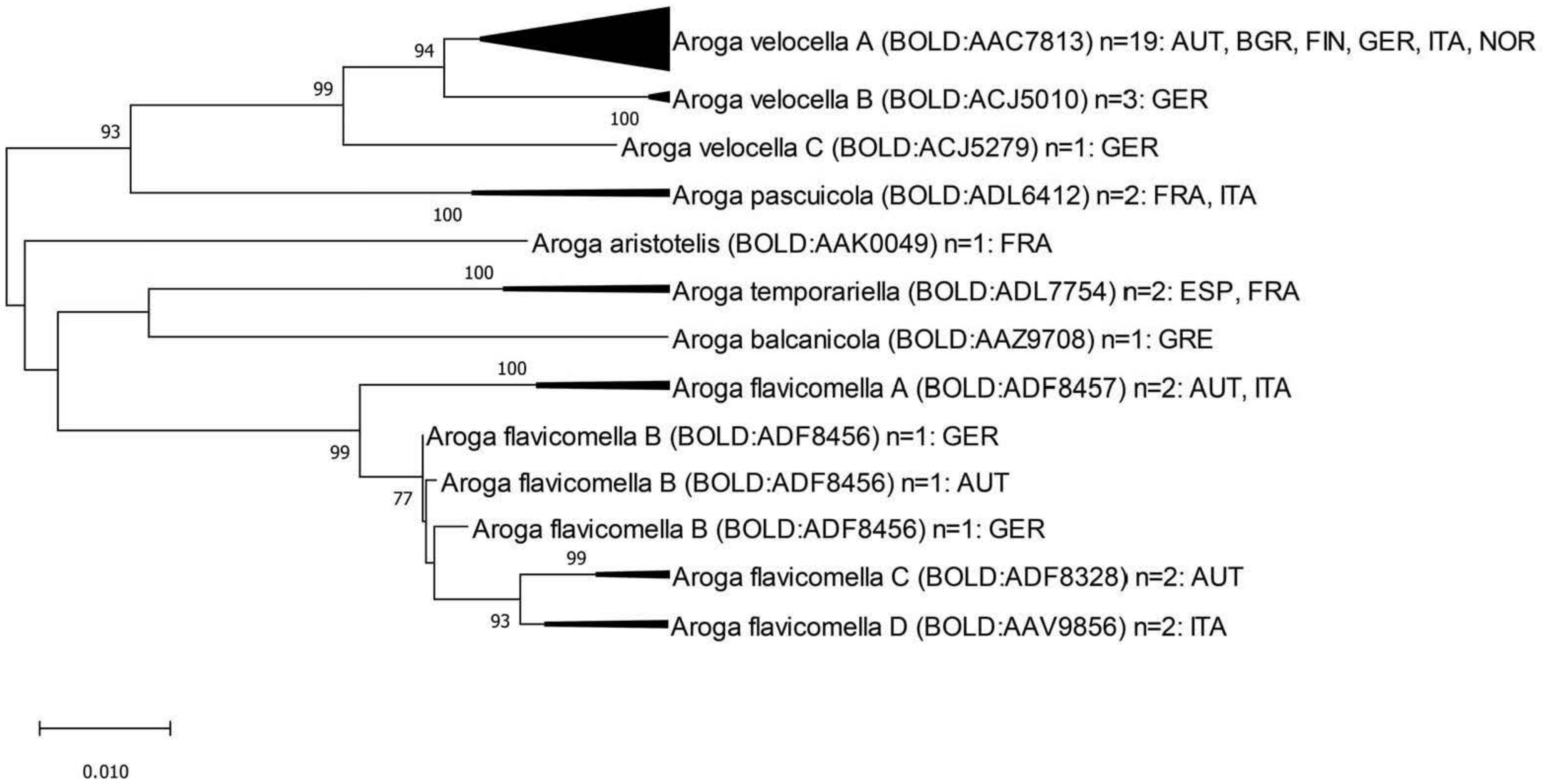
Aroga velocella A (BOLD:AAC7813) n=19: AUT, BGR, FIN, GER, ITA, NOR
Aroga velocella B (BOLD:ACJ5010) n=3: GER
Aroga velocella C (BOLD:ACJ5279) n=1: GER
Aroga pascuicola (BOLD:ADL6412) n=2: FRA, ITA
Aroga aristotelis (BOLD:AAK0049) n=1: FRA
Aroga temporariella (BOLD:ADL7754) n=2: ESP, FRA
Aroga balcanicola (BOLD:AAZ9708) n=1: GRE
Aroga flavicomella A (BOLD:ADF8457) n=2: AUT, ITA
Aroga flavicomella B (BOLD:ADF8456) n=1: GER
Aroga flavicomella B (BOLD:ADF8456) n=1: AUT
Aroga flavicomella B (BOLD:ADF8456) n=1: GER
Aroga flavicomella C (BOLD:ADF8328) n=2: AUT
Aroga flavicomella D (BOLD:AAV9856) n=2: ITA
94
99
93
100
100
100
100
99
77
99
93
0.010

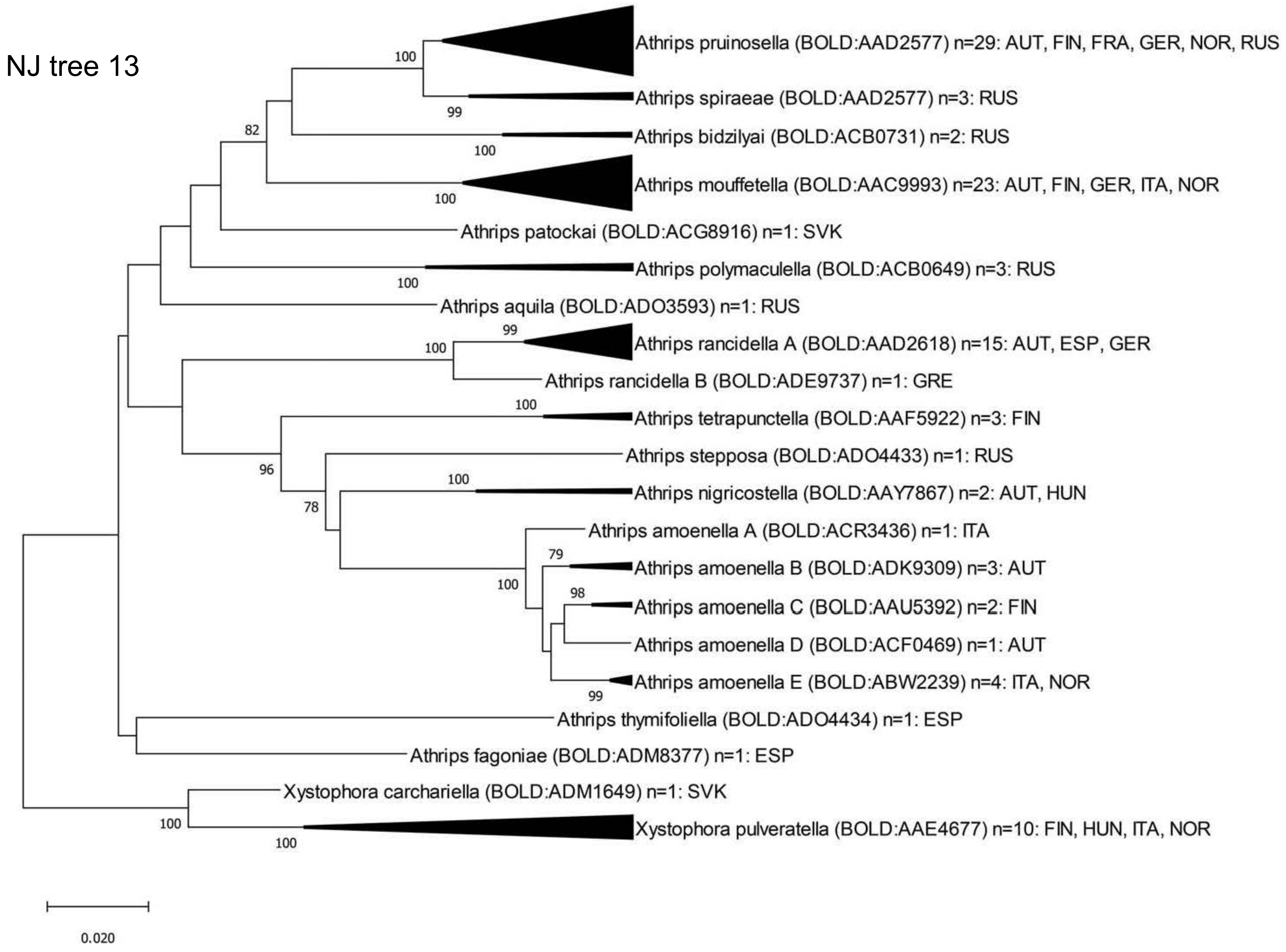
NJ tree 13
Athrips pruinosella (BOLD:AAD2577) n=29: AUT, FIN, FRA, GER, NOR, RUS
Athrips spiraeae (BOLD:AAD2577) n=3: RUS
Athrips bidzilyai (BOLD:ACB0731) n=2: RUS
Athrips mouffetella (BOLD:AAC9993) n=23: AUT, FIN, GER, ITA, NOR
Athrips patockai (BOLD:ACG8916) n=1: SVK
Athrips polymaculella (BOLD:ACB0649) n=3: RUS
Athrips aquila (BOLD:ADO3593) n=1: RUS
Athrips rancidella A (BOLD:AAD2618) n=15: AUT, ESP, GER
Athrips rancidella B (BOLD:ADE9737) n=1: GRE
Athrips tetrapunctella (BOLD:AAF5922) n=3: FIN
Athrips stepposa (BOLD:ADO4433) n=1: RUS
Athrips nigricostella (BOLD:AAY7867) n=2: AUT, HUN
Athrips amoenella A (BOLD:ACR3436) n=1: ITA
Athrips amoenella B (BOLD:ADK9309) n=3: AUT
Athrips amoenella C (BOLD:AAU5392) n=2: FIN
Athrips amoenella D (BOLD:ACF0469) n=1: AUT
Athrips amoenella E (BOLD:ABW2239) n=4: ITA, NOR
Athrips thymifoliella (BOLD:ADO4434) n=1: ESP
Athrips fagoniae (BOLD:ADM8377) n=1: ESP
Xystophora carchariella (BOLD:ADM1649) n=1: SVK
Xystophora pulveratella (BOLD:AAE4677) n=10: FIN, HUN, ITA, NOR
0.020

NJ tree 14

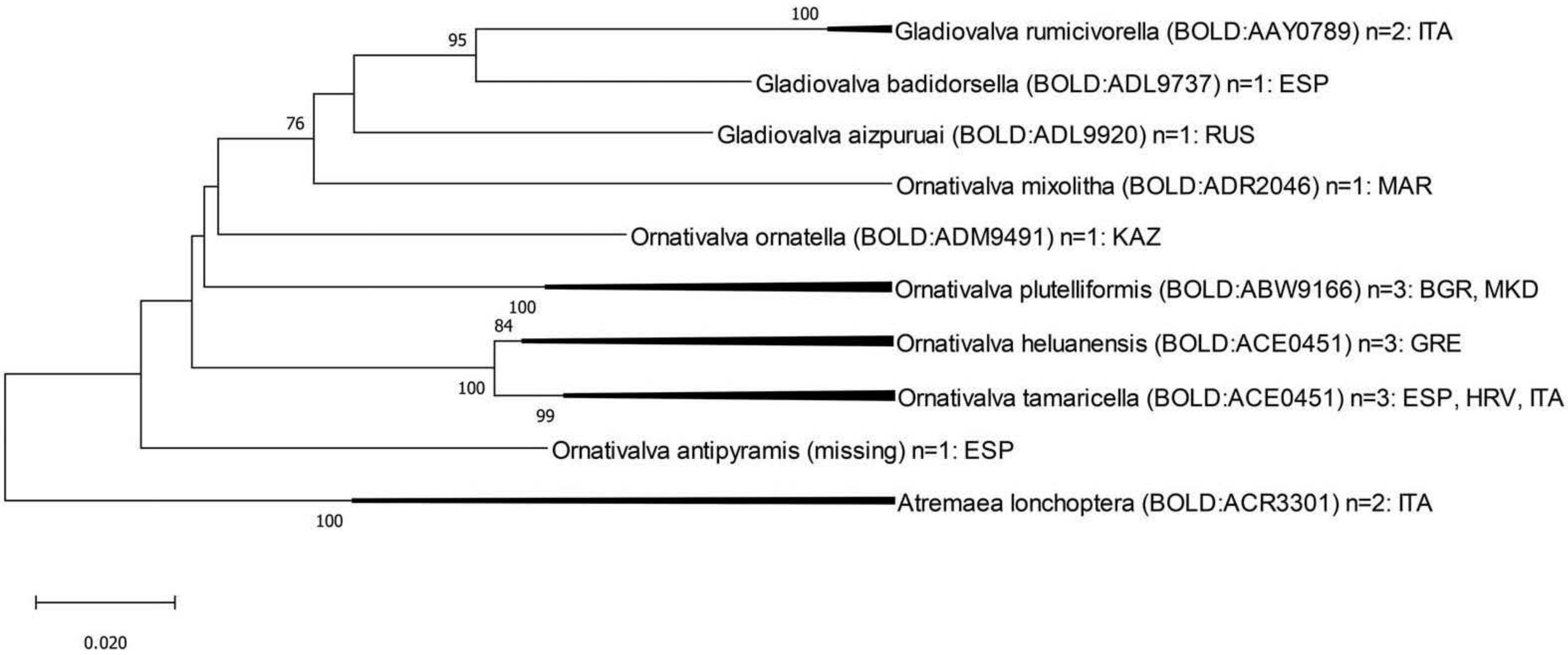

100
95
Gladiovalva rumicivorella (BOLD:AAY0789) n=2: ITA
Gladiovalva badidorsella (BOLD:ADL9737) n=1: ESP
76
Gladiovalva aizpuruai (BOLD:ADL9920) n=1: RUS
Ornativalva mixolitha (BOLD:ADR2046) n=1: MAR
Ornativalva ornatella (BOLD:ADM9491) n=1: KAZ
Ornativalva plutelliformis (BOLD:ABW9166) n=3: BGR, MKD
100
84
Ornativalva heluanensis (BOLD:ACE0451) n=3: GRE
100
Ornativalva tamaricella (BOLD:ACE0451) n=3: ESP, HRV, ITA
99
Ornativalva antipyramis (missing) n=1: ESP
Atremaea lonchoptera (BOLD:ACR3301) n=2: ITA
100
0.020

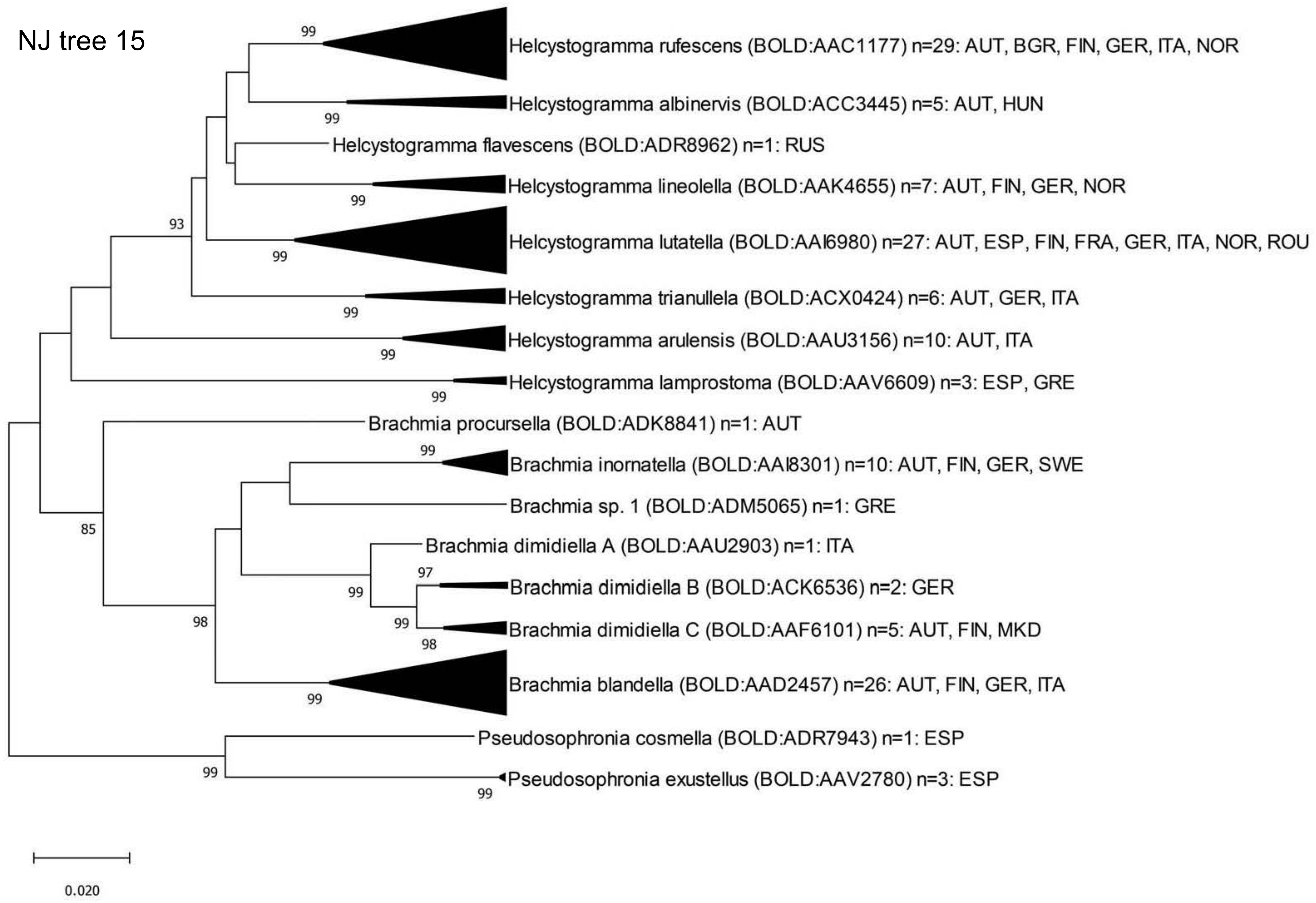
NJ tree 15
Helcystogramma rufescens (BOLD:AAC1177) n=29: AUT, BGR, FIN, GER, ITA, NOR
Helcystogramma albinervis (BOLD:ACC3445) n=5: AUT, HUN
Helcystogramma flavescens (BOLD:ADR8962) n=1: RUS
Helcystogramma lineolella (BOLD:AAK4655) n=7: AUT, FIN, GER, NOR
Helcystogramma lutatella (BOLD:AAI6980) n=27: AUT, ESP, FIN, FRA, GER, ITA, NOR, ROU
Helcystogramma trianullela (BOLD:ACX0424) n=6: AUT, GER, ITA
Helcystogramma arulensis (BOLD:AAU3156) n=10: AUT, ITA
Helcystogramma lamprostoma (BOLD:AAV6609) n=3: ESP, GRE
Brachmia procursella (BOLD:ADK8841) n=1: AUT
Brachmia inornatella (BOLD:AAI8301) n=10: AUT, FIN, GER, SWE
Brachmia sp. 1 (BOLD:ADM5065) n=1: GRE
Brachmia dimidiella A (BOLD:AAU2903) n=1: ITA
Brachmia dimidiella B (BOLD:ACK6536) n=2: GER
Brachmia dimidiella C (BOLD:AAF6101) n=5: AUT, FIN, MKD
Brachmia blandella (BOLD:AAD2457) n=26: AUT, FIN, GER, ITA
Pseudosophronia cosmella (BOLD:ADR7943) n=1: ESP
Pseudosophronia exustellus (BOLD:AAV2780) n=3: ESP
99
99
99
93
99
99
99
99
99
97
85
99
99
98
98
99
99
99
0.020

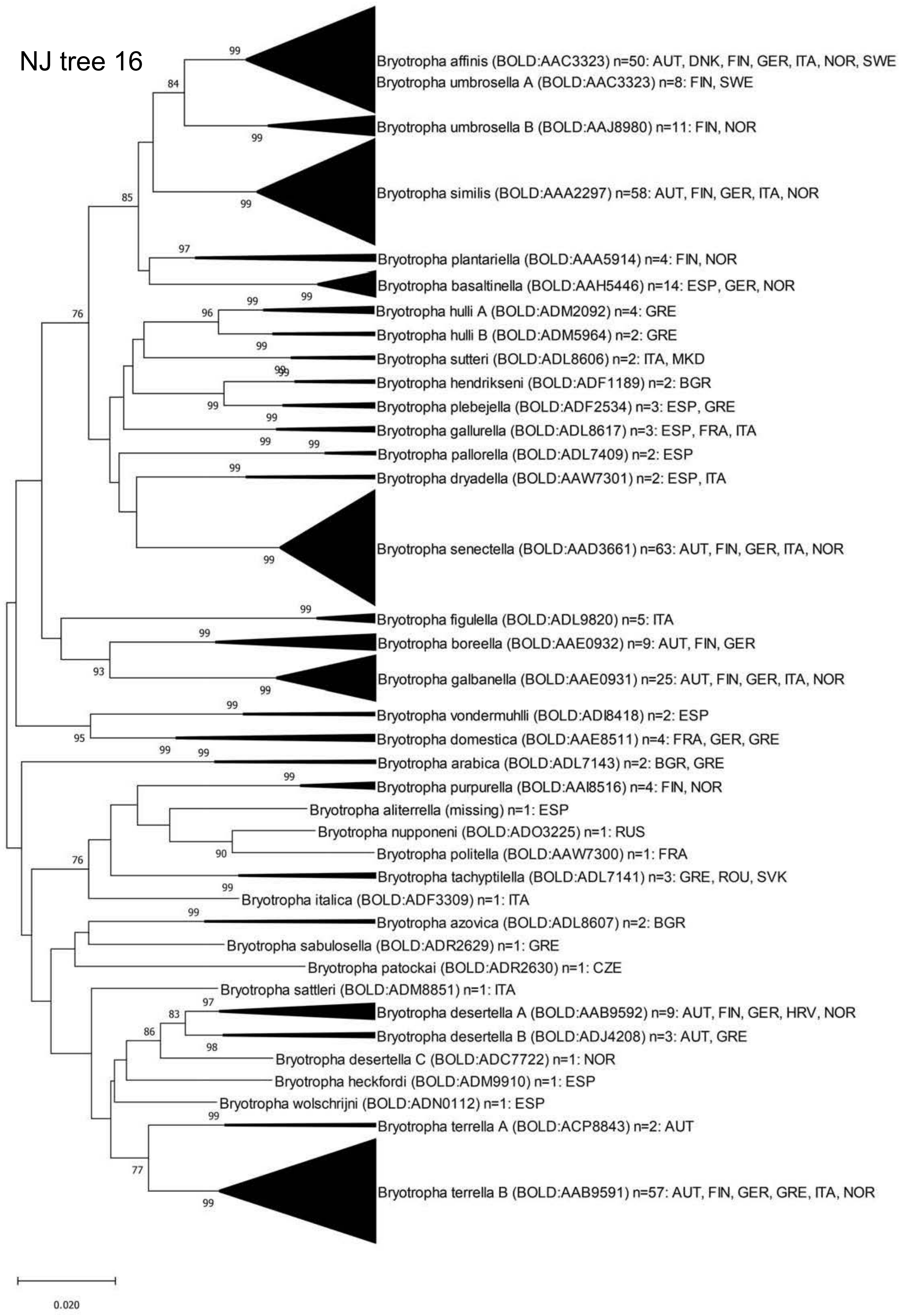

NJ tree 16
Bryotropha affinis (BOLD:AAC3323) n=50: AUT, DNK, FIN, GER, ITA, NOR, SWE
Bryotropha umbrosella A (BOLD:AAC3323) n=8: FIN, SWE
Bryotropha umbrosella B (BOLD:AAJ8980) n=11: FIN, NOR
Bryotropha similis (BOLD:AAA2297) n=58: AUT, FIN, GER, ITA, NOR
Bryotropha plantariella (BOLD:AAA5914) n=4: FIN, NOR
Bryotropha basaltinella (BOLD:AAH5446) n=14: ESP, GER, NOR
Bryotropha hulli A (BOLD:ADM2092) n=4: GRE
Bryotropha hulli B (BOLD:ADM5964) n=2: GRE
Bryotropha sutteri (BOLD:ADL8606) n=2: ITA, MKD
Bryotropha hendrikseni (BOLD:ADF1189) n=2: BGR
Bryotropha plebejella (BOLD:ADF2534) n=3: ESP, GRE
Bryotropha gallurella (BOLD:ADL8617) n=3: ESP, FRA, ITA
Bryotropha pallorella (BOLD:ADL7409) n=2: ESP
Bryotropha dryadella (BOLD:AAW7301) n=2: ESP, ITA
Bryotropha senectella (BOLD:AAD3661) n=63: AUT, FIN, GER, ITA, NOR
Bryotropha figulella (BOLD:ADL9820) n=5: ITA
Bryotropha boreella (BOLD:AAE0932) n=9: AUT, FIN, GER
Bryotropha galbanella (BOLD:AAE0931) n=25: AUT, FIN, GER, ITA, NOR
Bryotropha vondermuhlli (BOLD:ADI8418) n=2: ESP
Bryotropha domestica (BOLD:AAE8511) n=4: FRA, GER, GRE
Bryotropha arabica (BOLD:ADL7143) n=2: BGR, GRE
Bryotropha purpurella (BOLD:AAI8516) n=4: FIN, NOR
Bryotropha aliterrella (missing) n=1: ESP
Bryotropha nupponeni (BOLD:ADO3225) n=1: RUS
Bryotropha politella (BOLD:AAW7300) n=1: FRA
Bryotropha tachyptilella (BOLD:ADL7141) n=3: GRE, ROU, SVK
Bryotropha italica (BOLD:ADF3309) n=1: ITA
Bryotropha azovica (BOLD:ADL8607) n=2: BGR
Bryotropha sabulosella (BOLD:ADR2629) n=1: GRE
Bryotropha patockai (BOLD:ADR2630) n=1: CZE
Bryotropha sattleri (BOLD:ADM8851) n=1: ITA
Bryotropha desertella A (BOLD:AAB9592) n=9: AUT, FIN, GER, HRV, NOR
Bryotropha desertella B (BOLD:ADJ4208) n=3: AUT, GRE
Bryotropha desertella C (BOLD:ADC7722) n=1: NOR
Bryotropha heckfordi (BOLD:ADM9910) n=1: ESP
Bryotropha wolschrijni (BOLD:ADN0112) n=1: ESP
Bryotropha terrella A (BOLD:ACP8843) n=2: AUT
Bryotropha terrella B (BOLD:AAB9591) n=57: AUT, FIN, GER, GRE, ITA, NOR
0.020

NJ tree 17

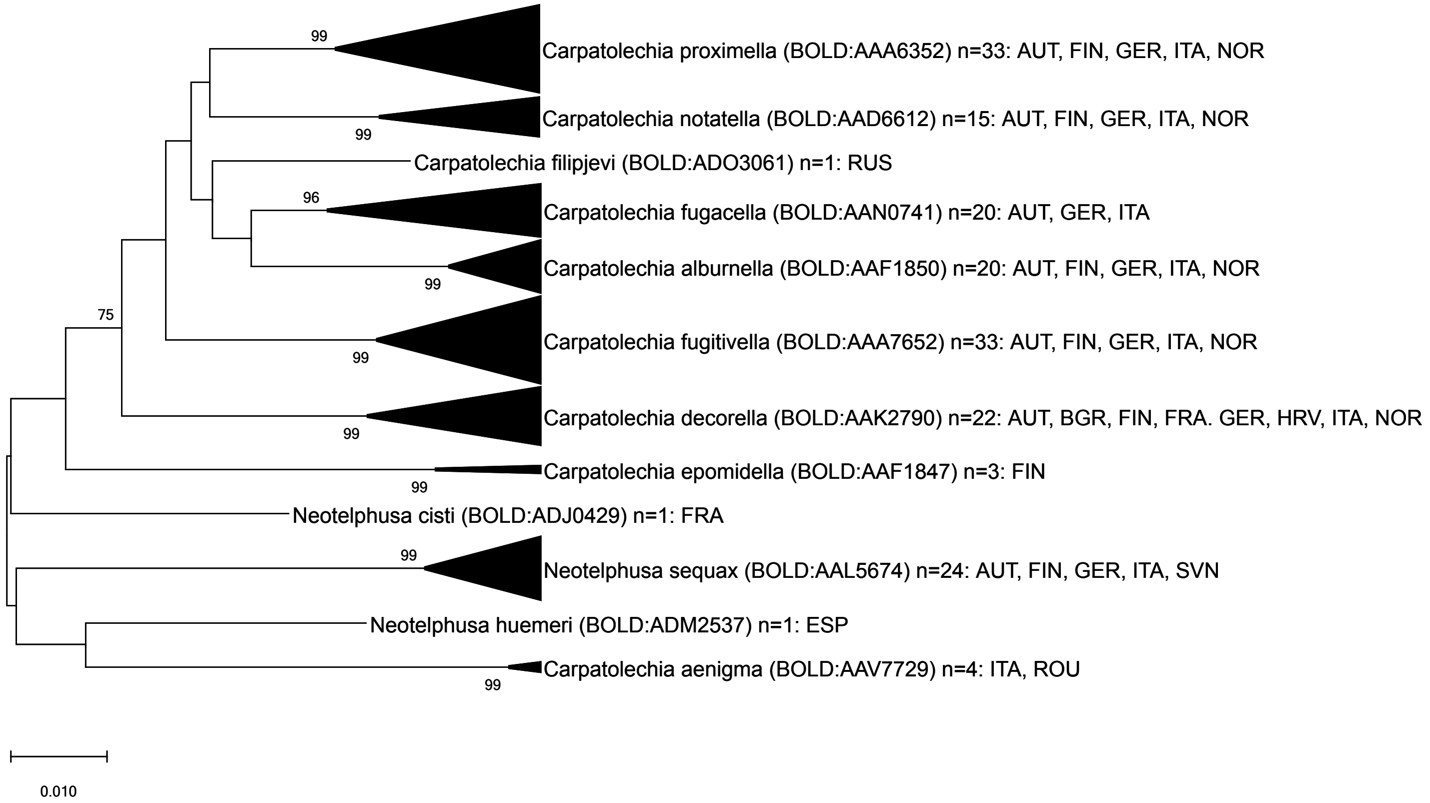
99
Carpatolechia proximella (BOLD:AAA6352) n=33: AUT, FIN, GER, ITA, NOR
99
Carpatolechia notatella (BOLD:AAD6612) n=15: AUT, FIN, GER, ITA, NOR
Carpatolechia filipjevi (BOLD:ADO3061) n=1: RUS
96
Carpatolechia fugacella (BOLD:AAN0741) n=20: AUT, GER, ITA
99
Carpatolechia alburnella (BOLD:AAF1850) n=20: AUT, FIN, GER, ITA, NOR
75
99
Carpatolechia fugitivella (BOLD:AAA7652) n=33: AUT, FIN, GER, ITA, NOR
99
Carpatolechia decorella (BOLD:AAK2790) n=22: AUT, BGR, FIN, FRA. GER, HRV, ITA, NOR
99
Carpatolechia epomidella (BOLD:AAF1847) n=3: FIN
Neotelphusa cisti (BOLD:ADJ0429) n=1: FRA
99
Neotelphusa sequax (BOLD:AAL5674) n=24: AUT, FIN, GER, ITA, SVN
Neotelphusa huemeri (BOLD:ADM2537) n=1: ESP
99
Carpatolechia aenigma (BOLD:AAV7729) n=4: ITA, ROU
0.010

NJ tree 18

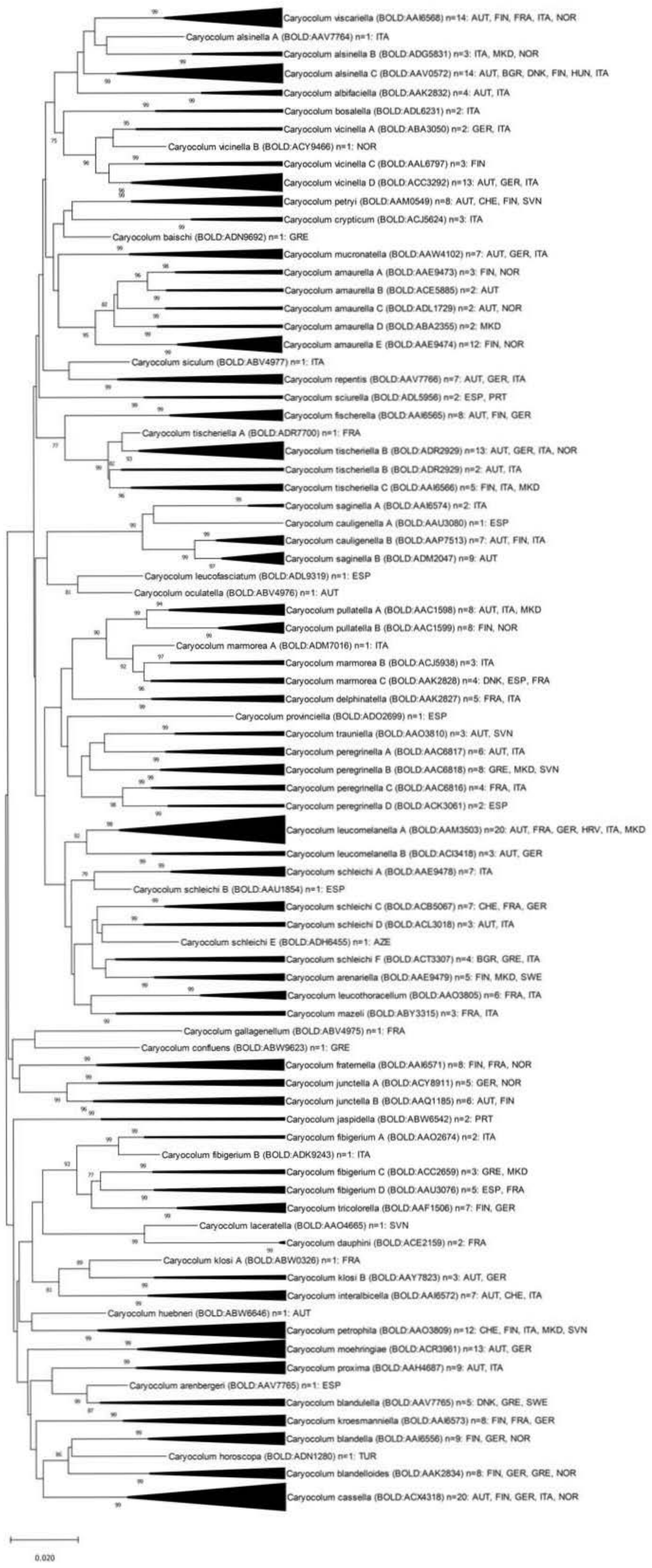

Caryocolum viscariella (BOLD:AAI6568) n=14: AUT, FIN, FRA, ITA, NOR
Caryocolum alsinella A (BOLD:AAV7764) n=1: ITA
Caryocolum alsinella B (BOLD:ADG5831) n=3: ITA, MKD, NOR
Caryocolum alsinella C (BOLD:AAV0572) n=14: AUT, BGR, DNK, FIN, HUN, ITA
Caryocolum albifaciella (BOLD:AAK2832) n=4: AUT, ITA
Caryocolum bosalella (BOLD:ADL6231) n=2: ITA
Caryocolum vicinella A (BOLD:ABA3050) n=2: GER, ITA
Caryocolum vicinella B (BOLD:ACY9466) n=1: NOR
Caryocolum vicinella C (BOLD:AAL6797) n=3: FIN
Caryocolum vicinella D (BOLD:ACC3292) n=13: AUT, GER, ITA
Caryocolum petryi (BOLD:AAM0549) n=8: AUT, CHE, FIN, SVN
Caryocolum crypticum (BOLD:ACJ5624) n=3: ITA
Caryocolum baischi (BOLD:ADN9692) n=1: GRE
Caryocolum mucronatella (BOLD:AAW4102) n=7: AUT, GER, ITA
Caryocolum amaurella A (BOLD:AAE9473) n=3: FIN, NOR
Caryocolum amaurella B (BOLD:ACE5885) n=2: AUT
Caryocolum amaurella C (BOLD:ADL1729) n=2: AUT, NOR
Caryocolum amaurella D (BOLD:ABA2355) n=2: MKD
Caryocolum amaurella E (BOLD:AAE9474) n=12: FIN, NOR
Caryocolum siculum (BOLD:ABV4977) n=1: ITA
Caryocolum repentis (BOLD:AAV7766) n=7: AUT, GER, ITA
Caryocolum sciurella (BOLD:ADL5956) n=2: ESP, PRT
Caryocolum fischerella (BOLD:AAI6565) n=8: AUT, FIN, GER
Caryocolum tischeriella A (BOLD:ADR7700) n=1: FRA
Caryocolum tischeriella B (BOLD:ADR2929) n=13: AUT, GER, ITA, NOR
Caryocolum tischeriella B (BOLD:ADR2929) n=2: AUT, ITA
Caryocolum tischeriella C (BOLD:AAI6566) n=5: FIN, ITA, MKD
Caryocolum saginella A (BOLD:AAI6574) n=2: ITA
Caryocolum cauligenella A (BOLD:AAU3080) n=1: ESP
Caryocolum cauligenella B (BOLD:AAP7513) n=7: AUT, FIN, ITA
Caryocolum saginella B (BOLD:ADM2047) n=9: AUT
Caryocolum leucofasciatum (BOLD:ADL9319) n=1: ESP
Caryocolum oculatella (BOLD:ABV4976) n=1: AUT
Caryocolum pullatella A (BOLD:AAC1598) n=8: AUT, ITA, MKD
Caryocolum pullatella B (BOLD:AAC1599) n=8: FIN, NOR
Caryocolum marmorea A (BOLD:ADM7016) n=1: ITA
Caryocolum marmorea B (BOLD:ACJ5938) n=3: ITA
Caryocolum marmorea C (BOLD:AAK2828) n=4: DNK, ESP, FRA
Caryocolum delphinatella (BOLD:AAK2827) n=5: FRA, ITA
Caryocolum provinciella (BOLD:ADO2699) n=1: ESP
Caryocolum trauniella (BOLD:AAO3810) n=3: AUT, SVN
Caryocolum peregrinella A (BOLD:AAC6817) n=6: AUT, ITA
Caryocolum peregrinella B (BOLD:AAC6818) n=8: GRE, MKD, SVN
Caryocolum peregrinella C (BOLD:AAC6816) n=4: FRA, ITA
Caryocolum peregrinella D (BOLD:ACK3061) n=2: ESP
Caryocolum leucomelanella A (BOLD:AAM3503) n=20: AUT, FRA, GER, HRV, ITA, MKD
Caryocolum leucomelanella B (BOLD:ACI3418) n=3: AUT, GER
Caryocolum schleichi A (BOLD:AAE9478) n=7: ITA
Caryocolum schleichi B (BOLD:AAU1854) n=1: ESP
Caryocolum schleichi C (BOLD:ACB5067) n=7: CHE, FRA, GER
Caryocolum schleichi D (BOLD:ACL3018) n=3: AUT, ITA
Caryocolum schleichi E (BOLD:ADH6455) n=1: AZE
Caryocolum schleichi F (BOLD:ACT3307) n=4: BGR, GRE, ITA
Caryocolum arenariella (BOLD:AAE9479) n=5: FIN, MKD, SWE
Caryocolum leucothoracellum (BOLD:AAO3805) n=6: FRA, ITA
Caryocolum mazeli (BOLD:ABY3315) n=3: FRA, ITA
Caryocolum gallagenellum (BOLD:ABV4975) n=1: FRA
Caryocolum confluens (BOLD:ABW9623) n=1: GRE
Caryocolum fraternella (BOLD:AAI6571) n=8: FIN, FRA, NOR
Caryocolum junctella A (BOLD:ACY8911) n=5: GER, NOR
Caryocolum junctella B (BOLD:AAQ1185) n=6: AUT, FIN
Caryocolum jaspidella (BOLD:ABW6542) n=2: PRT
Caryocolum fibigerium A (BOLD:AAO2674) n=2: ITA
Caryocolum fibigerium B (BOLD:ADK9243) n=1: ITA
Caryocolum fibigerium C (BOLD:ACC2659) n=3: GRE, MKD
Caryocolum fibigerium D (BOLD:AAU3076) n=5: ESP, FRA
Caryocolum tricolorella (BOLD:AAF1506) n=7: FIN, GER
Caryocolum laceratella (BOLD:AAO4665) n=1: SVN
Caryocolum dauphini (BOLD:ACE2159) n=2: FRA
Caryocolum klosi A (BOLD:ABW0326) n=1: FRA
Caryocolum klosi B (BOLD:AAY7823) n=3: AUT, GER
Caryocolum interalbicella (BOLD:AAI6572) n=7: AUT, CHE, ITA
Caryocolum huebneri (BOLD:ABW6646) n=1: AUT
Caryocolum petrophila (BOLD:AAO3809) n=12: CHE, FIN, ITA, MKD, SVN
Caryocolum moehringiae (BOLD:ACR3961) n=13: AUT, GER
Caryocolum proxima (BOLD:AAH4687) n=9: AUT, ITA
Caryocolum arenbergeri (BOLD:AAV7765) n=1: ESP
Caryocolum blandulella (BOLD:AAV7765) n=5: DNK, GRE, SWE
Caryocolum kroesmanniella (BOLD:AAI6573) n=8: FIN, FRA, GER
Caryocolum blandella (BOLD:AAI6556) n=9: FIN, GER, NOR
Caryocolum horoscopa (BOLD:ADN1280) n=1: TUR
Caryocolum blandelloides (BOLD:AAK2834) n=8: FIN, GER, GRE, NOR
Caryocolum cassella (BOLD:ACX4318) n=20: AUT, FIN, GER, ITA, NOR
0.020

NJ tree 19

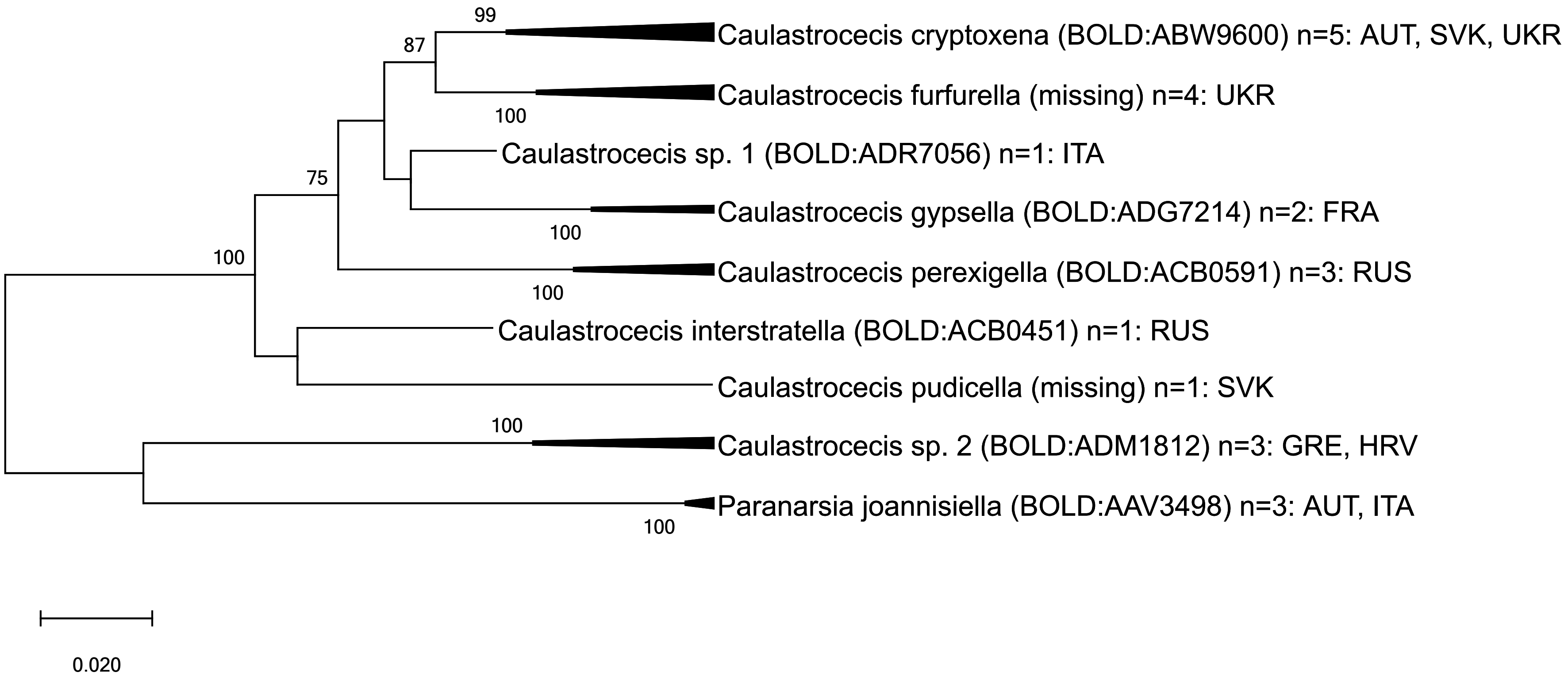

99
87
Caulastrocecis cryptoxena (BOLD:ABW9600) n=5: AUT, SVK, UKR
Caulastrocecis furfurella (missing) n=4: UKR
100
Caulastrocecis sp. 1 (BOLD:ADR7056) n=1: ITA
75
Caulastrocecis gypsella (BOLD:ADG7214) n=2: FRA
100
100
Caulastrocecis perexigella (BOLD:ACB0591) n=3: RUS
100
Caulastrocecis interstratella (BOLD:ACB0451) n=1: RUS
Caulastrocecis pudicella (missing) n=1: SVK
100
Caulastrocecis sp. 2 (BOLD:ADM1812) n=3: GRE, HRV
Paranarsia joannisiella (BOLD:AAV3498) n=3: AUT, ITA
100
0.020

NJ tree 20

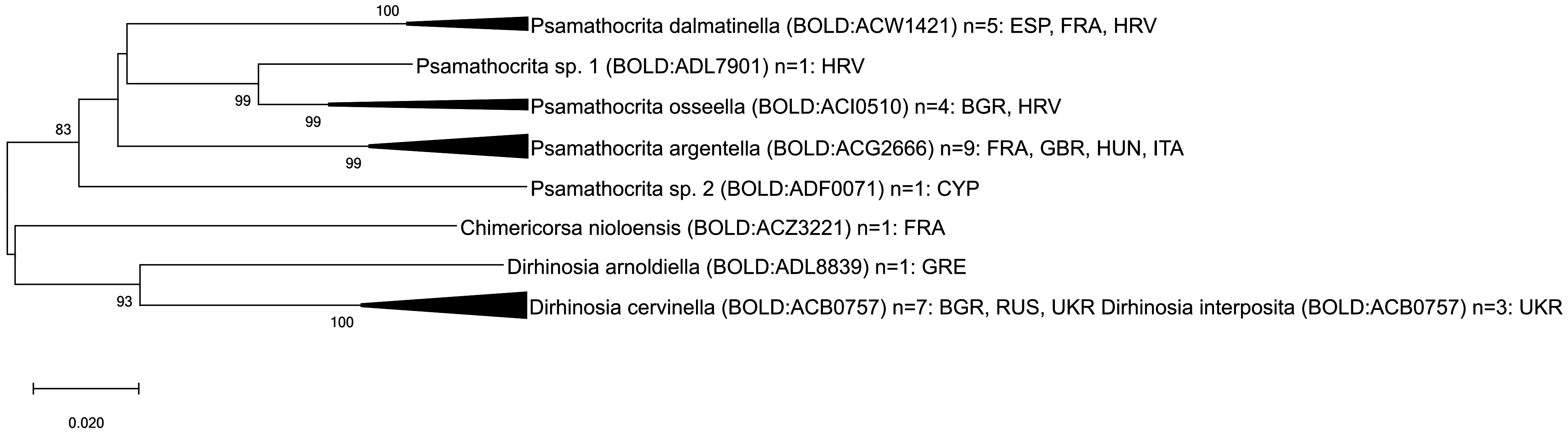

Psamathocrita dalmatinella (BOLD:ACW1421) n=5: ESP, FRA, HRV
Psamathocrita sp. 1 (BOLD:ADL7901) n=1: HRV
Psamathocrita osseella (BOLD:ACI0510) n=4: BGR, HRV
Psamathocrita argentella (BOLD:ACG2666) n=9: FRA, GBR, HUN, ITA
Psamathocrita sp. 2 (BOLD:ADF0071) n=1: CYP
Chimericorsa nioloensis (BOLD:ACZ3221) n=1: FRA
Dirhinosia arnoldiella (BOLD:ADL8839) n=1: GRE
Dirhinosia cervinella (BOLD:ACB0757) n=7: BGR, RUS, UKR Dirhinosia interposita (BOLD:ACB0757) n=3: UKR
100
99
99
99
83
93
100
0.020

NJ tree 21

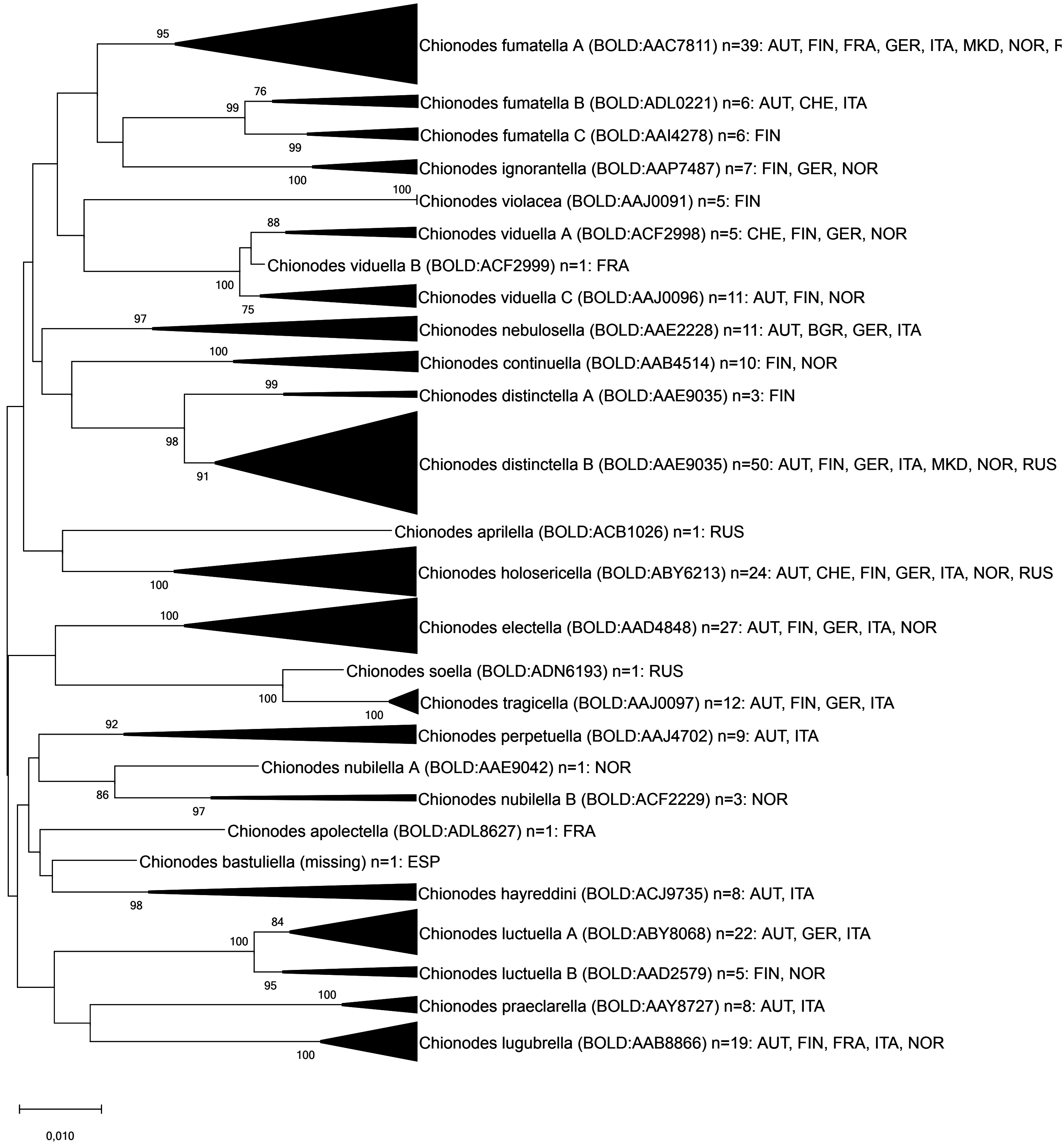

95
Chionodes fumatella A (BOLD:AAC7811) n=39: AUT, FIN, FRA, GER, ITA, MKD, NOR, F
76
99
Chionodes fumatella B (BOLD:ADL0221) n=6: AUT, CHE, ITA
99
Chionodes fumatella C (BOLD:AAI4278) n=6: FIN
100
Chionodes ignorantella (BOLD:AAP7487) n=7: FIN, GER, NOR
100
Chionodes violacea (BOLD:AAJ0091) n=5: FIN
88
Chionodes viduella A (BOLD:ACF2998) n=5: CHE, FIN, GER, NOR
Chionodes viduella B (BOLD:ACF2999) n=1: FRA
100
75
Chionodes viduella C (BOLD:AAJ0096) n=11: AUT, FIN, NOR
97
Chionodes nebulosella (BOLD:AAE2228) n=11: AUT, BGR, GER, ITA
100
Chionodes continuella (BOLD:AAB4514) n=10: FIN, NOR
99
Chionodes distinctella A (BOLD:AAE9035) n=3: FIN
98
91
Chionodes distinctella B (BOLD:AAE9035) n=50: AUT, FIN, GER, ITA, MKD, NOR, RUS
Chionodes aprilella (BOLD:ACB1026) n=1: RUS
100
Chionodes holosericella (BOLD:ABY6213) n=24: AUT, CHE, FIN, GER, ITA, NOR, RUS
100
Chionodes electella (BOLD:AAD4848) n=27: AUT, FIN, GER, ITA, NOR
Chionodes soella (BOLD:ADN6193) n=1: RUS
100
100
Chionodes tragicella (BOLD:AAJ0097) n=12: AUT, FIN, GER, ITA
92
Chionodes perpetuella (BOLD:AAJ4702) n=9: AUT, ITA
Chionodes nubilella A (BOLD:AAE9042) n=1: NOR
86
97
Chionodes nubilella B (BOLD:ACF2229) n=3: NOR
Chionodes apolectella (BOLD:ADL8627) n=1: FRA
Chionodes bastuliella (missing) n=1: ESP
98
Chionodes hayreddini (BOLD:ACJ9735) n=8: AUT, ITA
84
100
Chionodes luctuella A (BOLD:ABY8068) n=22: AUT, GER, ITA
95
Chionodes luctuella B (BOLD:AAD2579) n=5: FIN, NOR
100
Chionodes praeclarella (BOLD:AAY8727) n=8: AUT, ITA
100
Chionodes lugubrella (BOLD:AAB8866) n=19: AUT, FIN, FRA, ITA, NOR
0,010

NJ tree 22

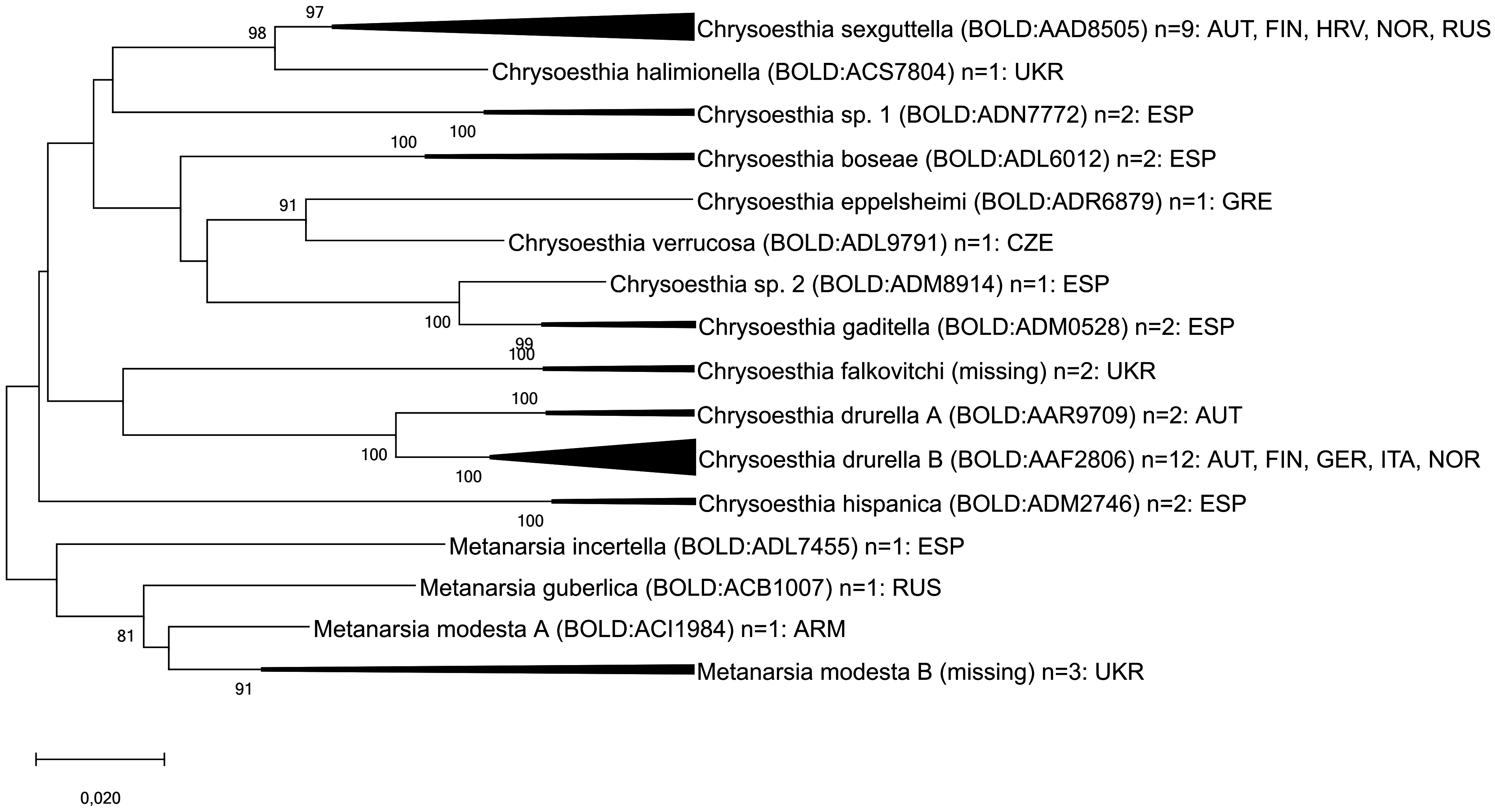
97
98
Chrysoesthia sexguttella (BOLD:AAD8505) n=9: AUT, FIN, HRV, NOR, RUS
Chrysoesthia halimionella (BOLD:ACS7804) n=1: UKR
Chrysoesthia sp. 1 (BOLD:ADN7772) n=2: ESP
100
100
Chrysoesthia boseae (BOLD:ADL6012) n=2: ESP
91
Chrysoesthia eppelsheimi (BOLD:ADR6879) n=1: GRE
Chrysoesthia verrucosa (BOLD:ADL9791) n=1: CZE
Chrysoesthia sp. 2 (BOLD:ADM8914) n=1: ESP
100
Chrysoesthia gaditella (BOLD:ADM0528) n=2: ESP
99
100
Chrysoesthia falkovitchi (missing) n=2: UKR
100
Chrysoesthia drurella A (BOLD:AAR9709) n=2: AUT
100
Chrysoesthia drurella B (BOLD:AAF2806) n=12: AUT, FIN, GER, ITA, NOR
100
Chrysoesthia hispanica (BOLD:ADM2746) n=2: ESP
100
Metanarsia incertella (BOLD:ADL7455) n=1: ESP
Metanarsia guberlica (BOLD:ACB1007) n=1: RUS
81
Metanarsia modesta A (BOLD:ACI1984) n=1: ARM
Metanarsia modesta B (missing) n=3: UKR
91
0,020

NJ tree 23

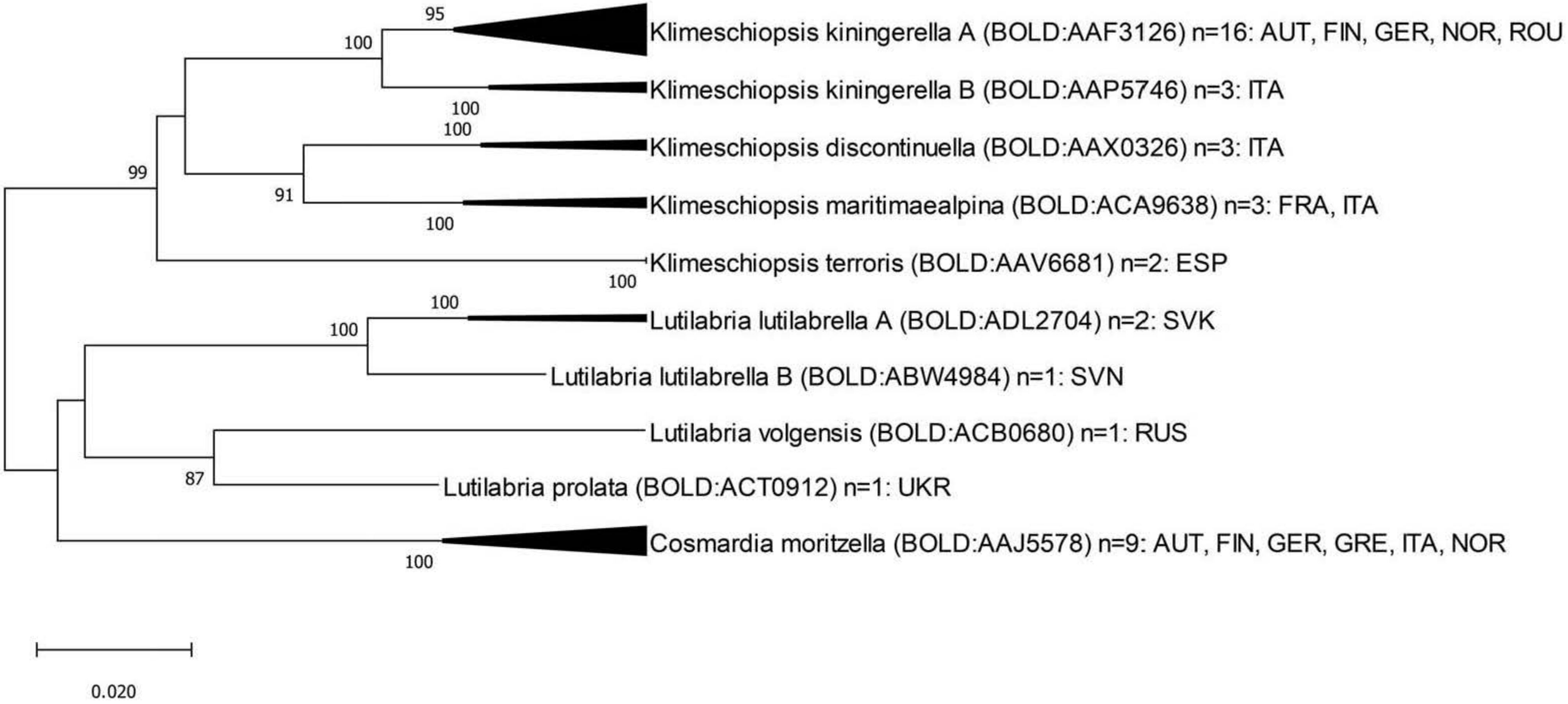

95
100
Klimeschiopsis kiningerella A (BOLD:AAF3126) n=16: AUT, FIN, GER, NOR, ROU
Klimeschiopsis kiningerella B (BOLD:AAP5746) n=3: ITA
100
100
Klimeschiopsis discontinuella (BOLD:AAX0326) n=3: ITA
99
91
Klimeschiopsis maritimaealpina (BOLD:ACA9638) n=3: FRA, ITA
100
Klimeschiopsis terroris (BOLD:AAV6681) n=2: ESP
100
100
Lutilabria lutilabrella A (BOLD:ADL2704) n=2: SVK
100
Lutilabria lutilabrella B (BOLD:ABW4984) n=1: SVN
Lutilabria volgensis (BOLD:ACB0680) n=1: RUS
87
Lutilabria prolata (BOLD:ACT0912) n=1: UKR
Cosmardia moritzella (BOLD:AAJ5578) n=9: AUT, FIN, GER, GRE, ITA, NOR
100
0.020

NJ tree 24

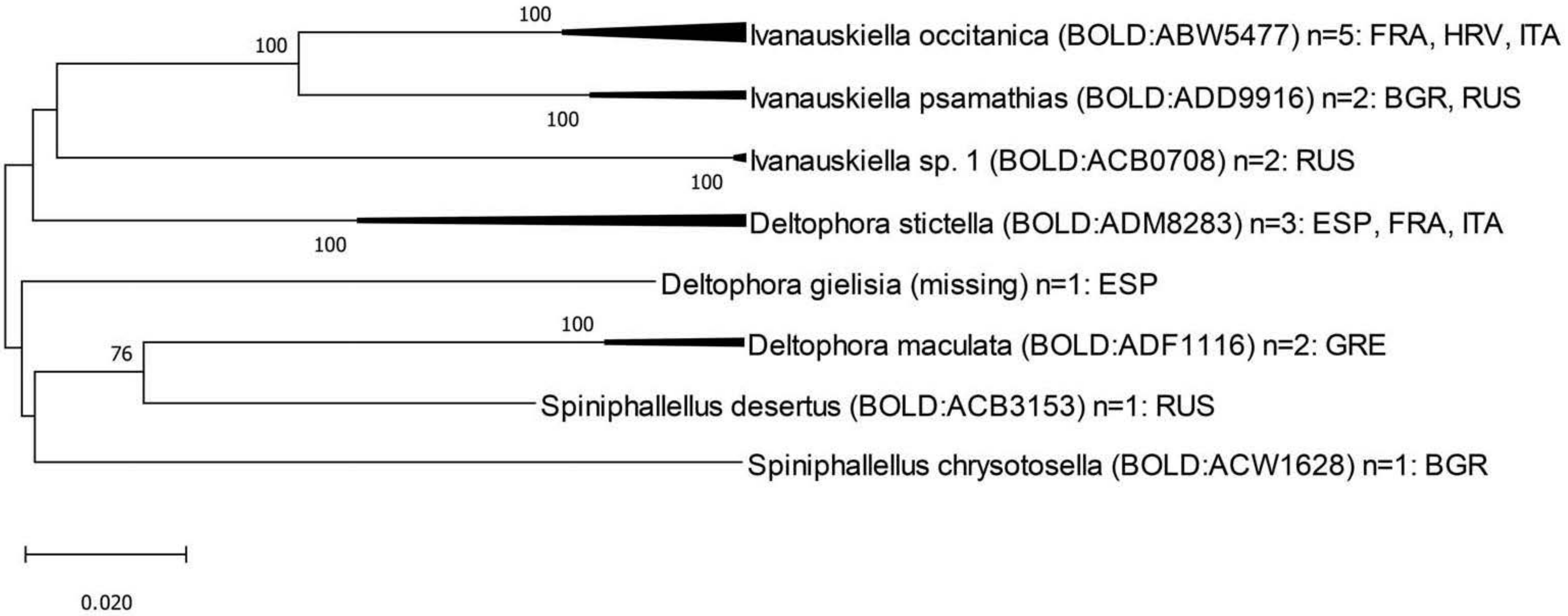
100
100
Ivanauskiella occitanica (BOLD:ABW5477) n=5: FRA, HRV, ITA
Ivanauskiella psamathias (BOLD:ADD9916) n=2: BGR, RUS
100
Ivanauskiella sp. 1 (BOLD:ACB0708) n=2: RUS
100
Deltophora stictella (BOLD:ADM8283) n=3: ESP, FRA, ITA
100
Deltophora gielisia (missing) n=1: ESP
100
Deltophora maculata (BOLD:ADF1116) n=2: GRE
76
Spiniphallellus desertus (BOLD:ACB3153) n=1: RUS
Spiniphallellus chrysotosella (BOLD:ACW1628) n=1: BGR
0.020

NJ tree 25

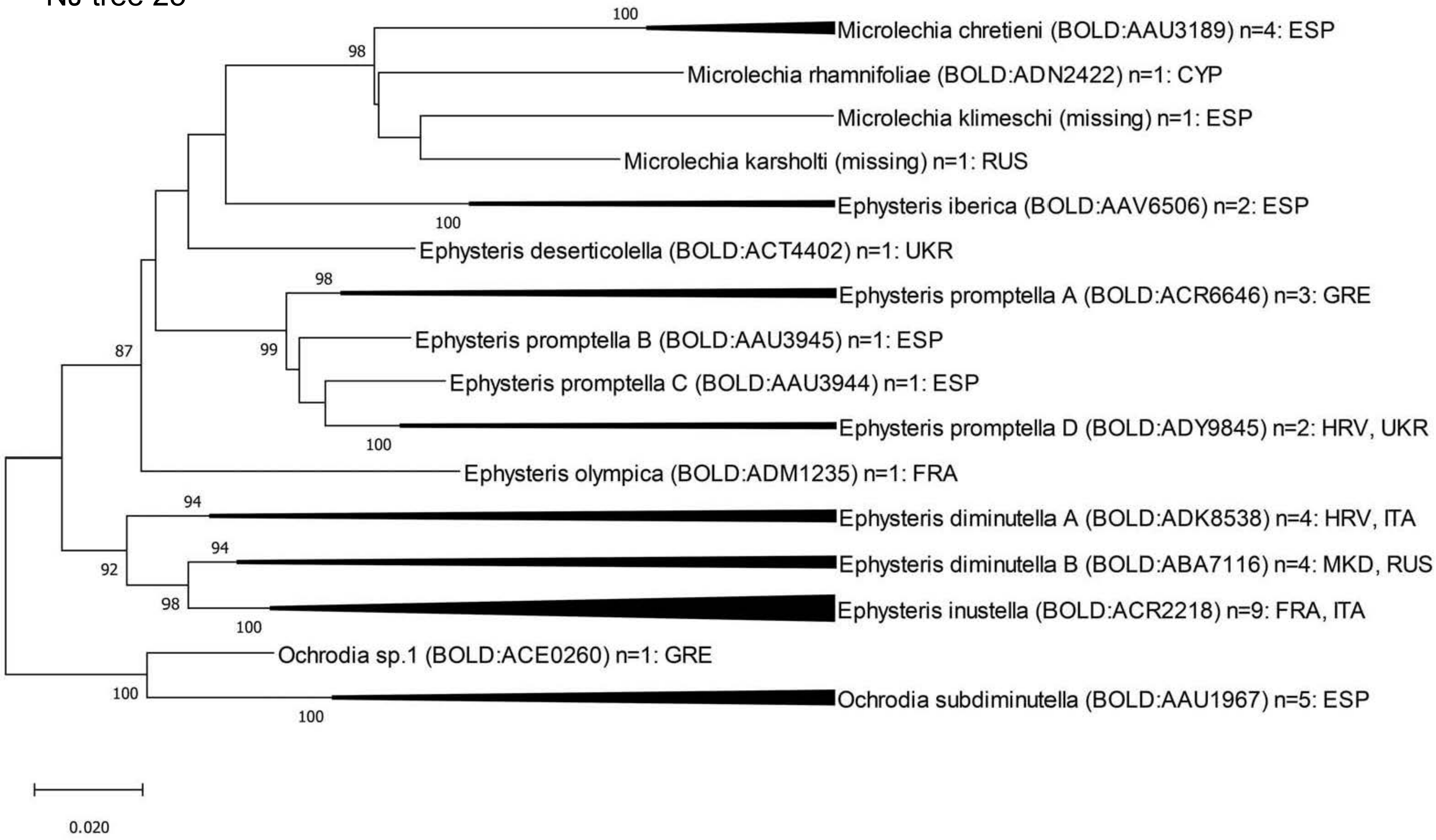

Microlechia chretieni (BOLD:AAU3189) n=4: ESP
Microlechia rhamnifoliae (BOLD:ADN2422) n=1: CYP
Microlechia klimeschi (missing) n=1: ESP
Microlechia karsholti (missing) n=1: RUS
Ephysteris iberica (BOLD:AAV6506) n=2: ESP
Ephysteris deserticolella (BOLD:ACT4402) n=1: UKR
Ephysteris promptella A (BOLD:ACR6646) n=3: GRE
Ephysteris promptella B (BOLD:AAU3945) n=1: ESP
Ephysteris promptella C (BOLD:AAU3944) n=1: ESP
Ephysteris promptella D (BOLD:ADY9845) n=2: HRV, UKR
Ephysteris olympica (BOLD:ADM1235) n=1: FRA
Ephysteris diminutella A (BOLD:ADK8538) n=4: HRV, ITA
Ephysteris diminutella B (BOLD:ABA7116) n=4: MKD, RUS
Ephysteris inustella (BOLD:ACR2218) n=9: FRA, ITA
Ochrodia sp.1 (BOLD:ACE0260) n=1: GRE
Ochrodia subdiminutella (BOLD:AAU1967) n=5: ESP
100
98
100
87
98
99
100
94
92
94
98
100
100
100
0.020

NJ tree 26

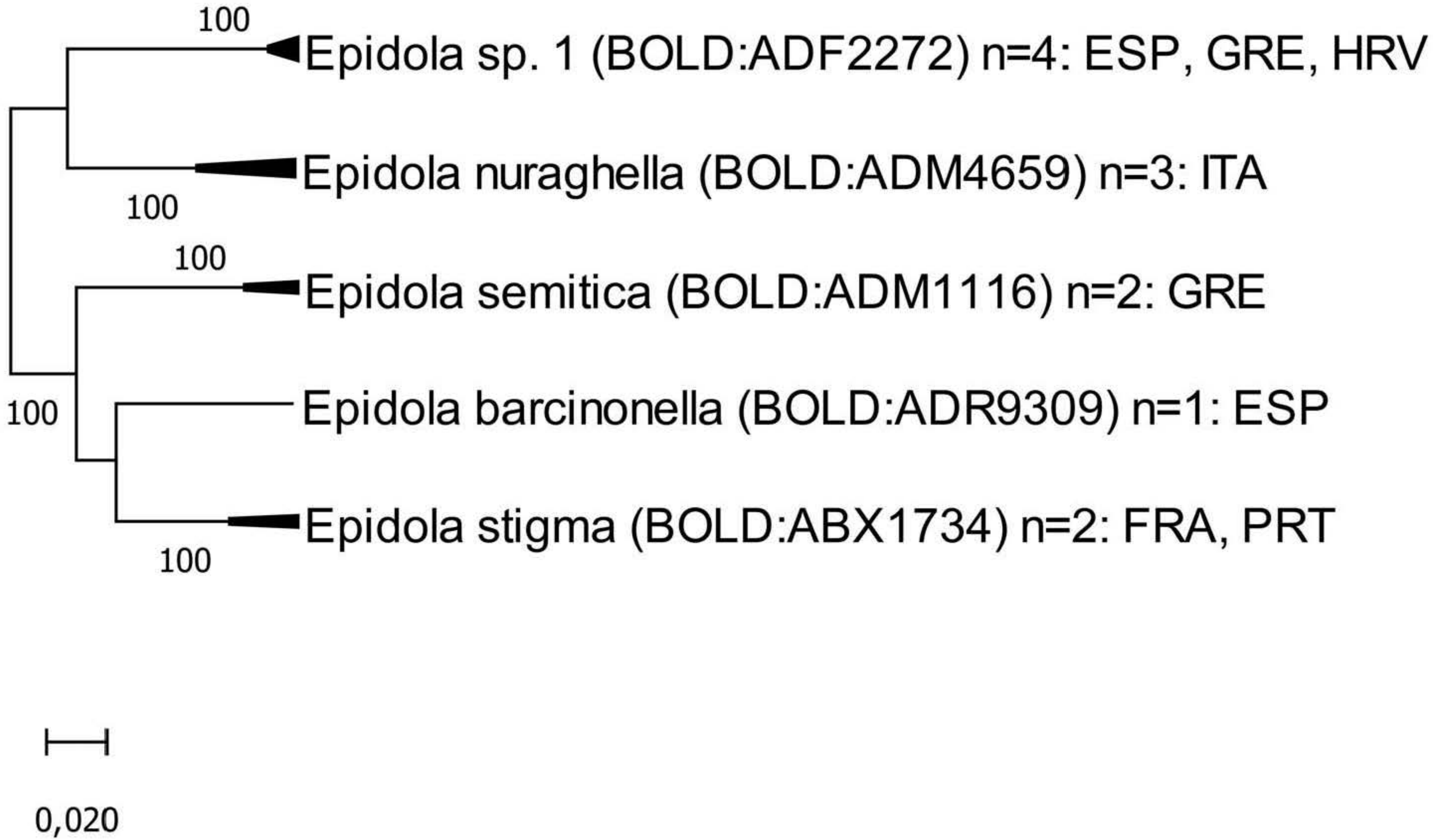
100
Epidola sp. 1 (BOLD:ADF2272) n=4: ESP, GRE, HRV
Epidola nuraghella (BOLD:ADM4659) n=3: ITA
100
100
Epidola semitica (BOLD:ADM1116) n=2: GRE
100
Epidola barcinonella (BOLD:ADR9309) n=1: ESP
Epidola stigma (BOLD:ABX1734) n=2: FRA, PRT
100
0,020

NJ tree 27

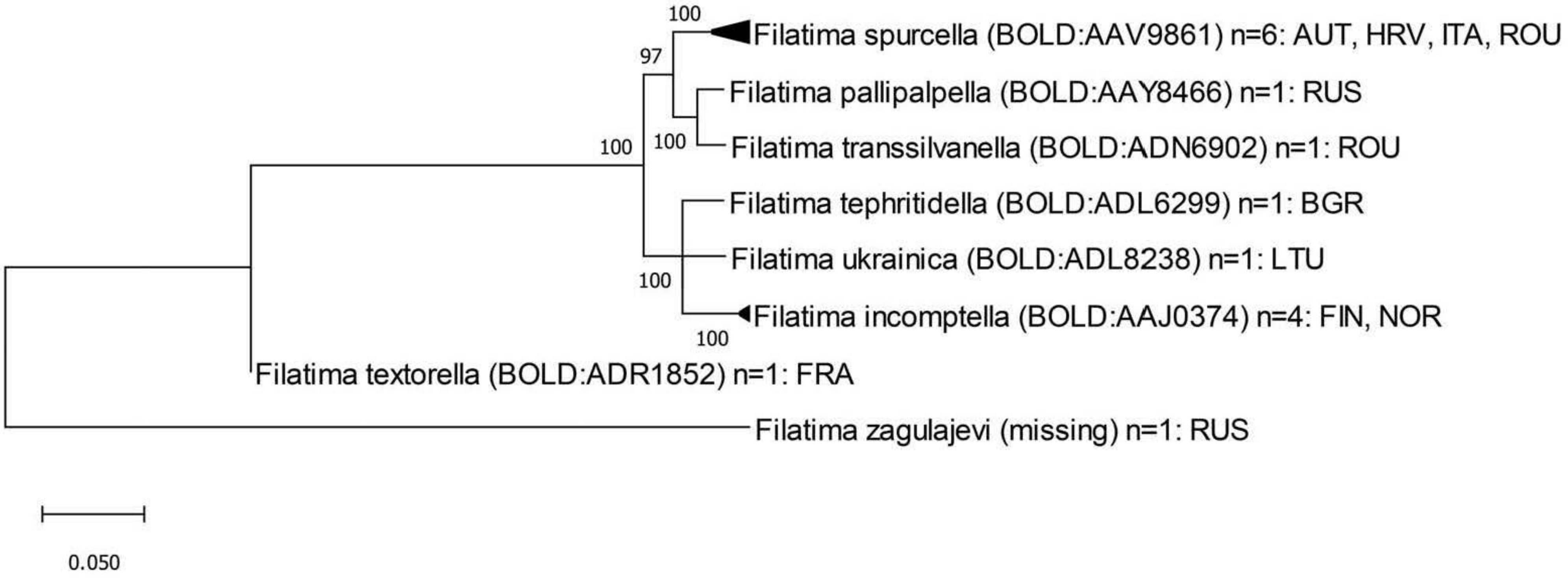

100
Filatima spurcella (BOLD:AAV9861) n=6: AUT, HRV, ITA, ROU
97
Filatima pallipalpella (BOLD:AAY8466) n=1: RUS
100
100
Filatima transsilvanella (BOLD:ADN6902) n=1: ROU
Filatima tephritidella (BOLD:ADL6299) n=1: BGR
Filatima ukrainica (BOLD:ADL8238) n=1: LTU
100
Filatima incomptella (BOLD:AAJ0374) n=4: FIN, NOR
100
Filatima textorella (BOLD:ADR1852) n=1: FRA
Filatima zagulajevi (missing) n=1: RUS
0.050

NJ tree 28

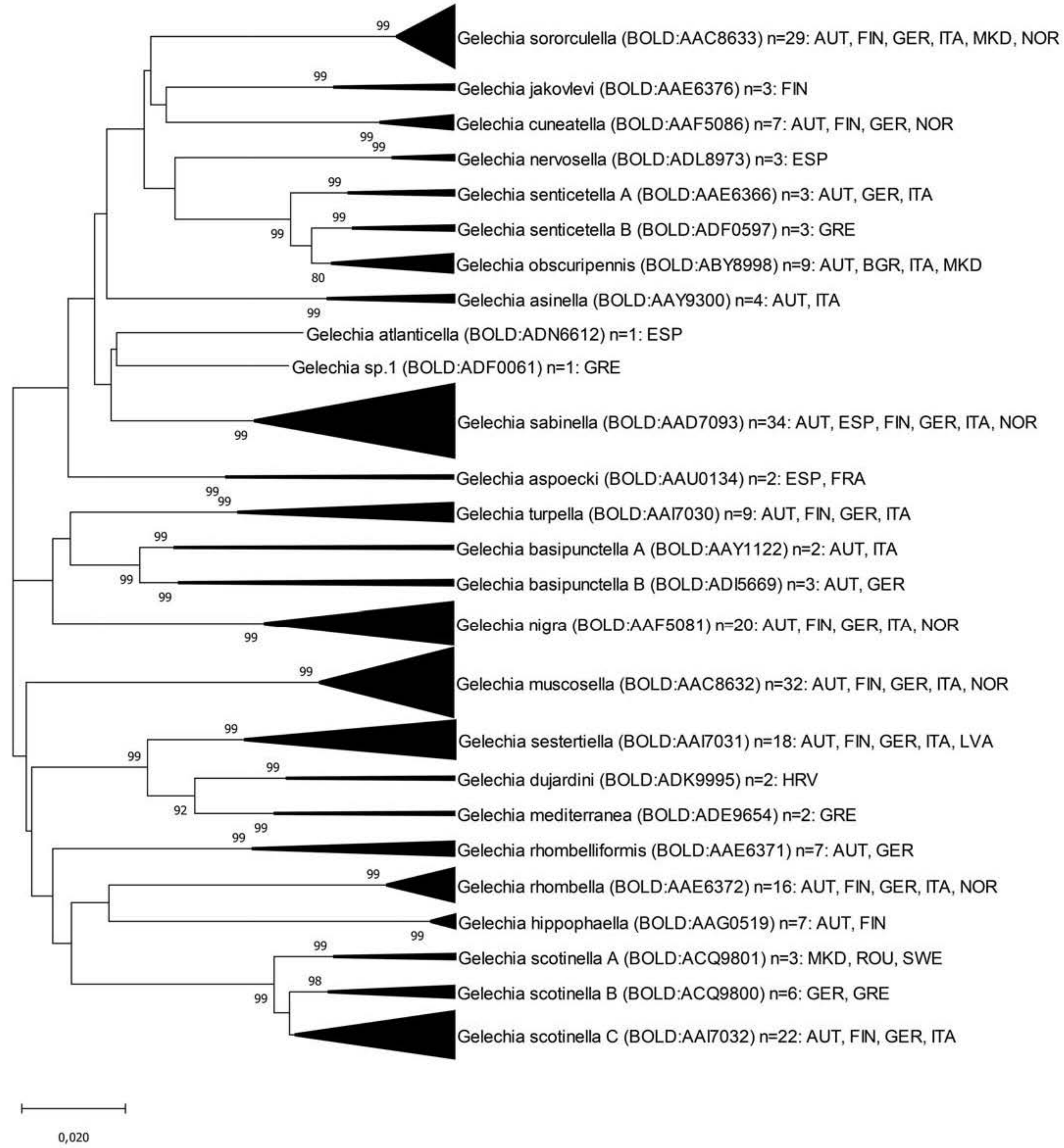

Gelechia sororculella (BOLD:AAC8633) n=29: AUT, FIN, GER, ITA, MKD, NOR
Gelechia jakovlevi (BOLD:AAE6376) n=3: FIN
Gelechia cuneatella (BOLD:AAF5086) n=7: AUT, FIN, GER, NOR
Gelechia nervosella (BOLD:ADL8973) n=3: ESP
Gelechia senticetella A (BOLD:AAE6366) n=3: AUT, GER, ITA
Gelechia senticetella B (BOLD:ADF0597) n=3: GRE
Gelechia obscuripennis (BOLD:ABY8998) n=9: AUT, BGR, ITA, MKD
Gelechia asinella (BOLD:AAY9300) n=4: AUT, ITA
Gelechia atlanticella (BOLD:ADN6612) n=1: ESP
Gelechia sp.1 (BOLD:ADF0061) n=1: GRE
Gelechia sabinella (BOLD:AAD7093) n=34: AUT, ESP, FIN, GER, ITA, NOR
Gelechia aspoecki (BOLD:AAU0134) n=2: ESP, FRA
Gelechia turpella (BOLD:AAI7030) n=9: AUT, FIN, GER, ITA
Gelechia basipunctella A (BOLD:AAY1122) n=2: AUT, ITA
Gelechia basipunctella B (BOLD:ADI5669) n=3: AUT, GER
Gelechia nigra (BOLD:AAF5081) n=20: AUT, FIN, GER, ITA, NOR
Gelechia muscosella (BOLD:AAC8632) n=32: AUT, FIN, GER, ITA, NOR
Gelechia sestertiella (BOLD:AAI7031) n=18: AUT, FIN, GER, ITA, LVA
Gelechia dujardini (BOLD:ADK9995) n=2: HRV
Gelechia mediterranea (BOLD:ADE9654) n=2: GRE
Gelechia rhombelliformis (BOLD:AAE6371) n=7: AUT, GER
Gelechia rhombella (BOLD:AAE6372) n=16: AUT, FIN, GER, ITA, NOR
Gelechia hippophaella (BOLD:AAG0519) n=7: AUT, FIN
Gelechia scotinella A (BOLD:ACQ9801) n=3: MKD, ROU, SWE
Gelechia scotinella B (BOLD:ACQ9800) n=6: GER, GRE
Gelechia scotinella C (BOLD:AAI7032) n=22: AUT, FIN, GER, ITA
0,020

NJ tree 29

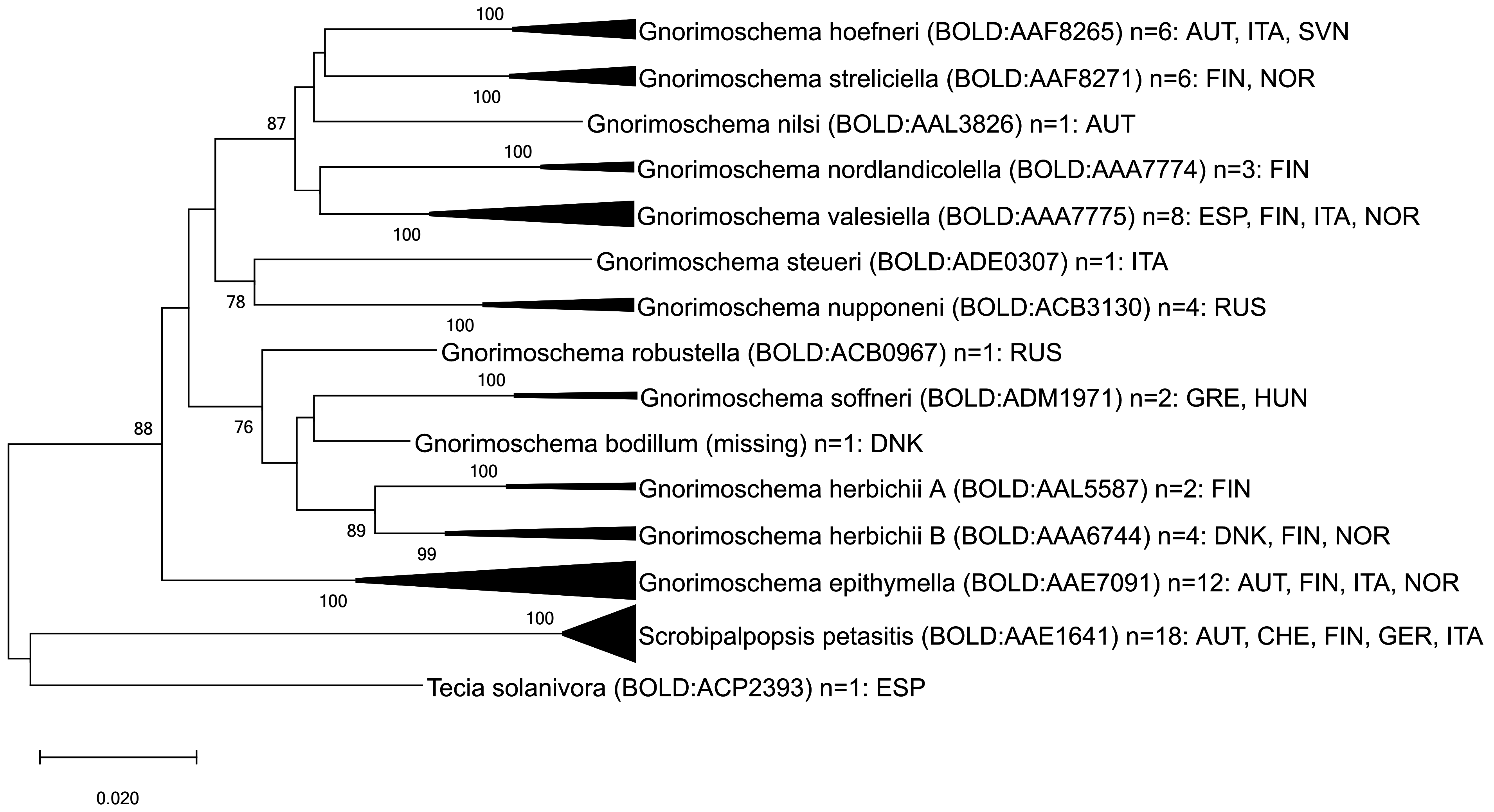
Gnorimoschema hoefneri (BOLD:AAF8265) n=6: AUT, ITA, SVN
Gnorimoschema streliciella (BOLD:AAF8271) n=6: FIN, NOR
Gnorimoschema nilsi (BOLD:AAL3826) n=1: AUT
Gnorimoschema nordlandicolella (BOLD:AAA7774) n=3: FIN
Gnorimoschema valesiella (BOLD:AAA7775) n=8: ESP, FIN, ITA, NOR
Gnorimoschema steueri (BOLD:ADE0307) n=1: ITA
Gnorimoschema nupponeni (BOLD:ACB3130) n=4: RUS
Gnorimoschema robustella (BOLD:ACB0967) n=1: RUS
Gnorimoschema soffneri (BOLD:ADM1971) n=2: GRE, HUN
Gnorimoschema bodillum (missing) n=1: DNK
Gnorimoschema herbichii A (BOLD:AAL5587) n=2: FIN
Gnorimoschema herbichii B (BOLD:AAA6744) n=4: DNK, FIN, NOR
Gnorimoschema epithymella (BOLD:AAE7091) n=12: AUT, FIN, ITA, NOR
Scrobipalpopsis petasitis (BOLD:AAE1641) n=18: AUT, CHE, FIN, GER, ITA
Tecia solanivora (BOLD:ACP2393) n=1: ESP
100
100
87
100
100
78
100
88
76
100
100
89
99
100
100
0.020

NJ tree 30

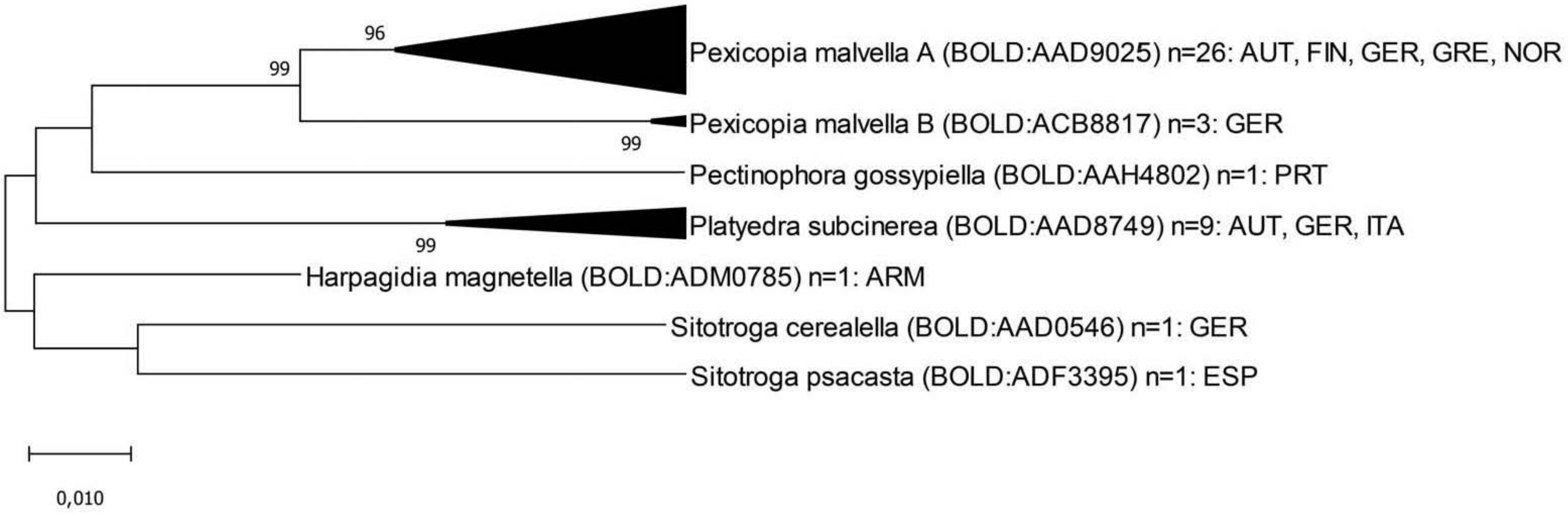
96
99
Pexicopia malvella A (BOLD:AAD9025) n=26: AUT, FIN, GER, GRE, NOR
Pexicopia malvella B (BOLD:ACB8817) n=3: GER
99
Pectinophora gossypiella (BOLD:AAH4802) n=1: PRT
Platyedra subcinerea (BOLD:AAD8749) n=9: AUT, GER, ITA
99
Harpagidia magnetella (BOLD:ADM0785) n=1: ARM
Sitotroga cerealella (BOLD:AAD0546) n=1: GER
Sitotroga psacasta (BOLD:ADF3395) n=1: ESP
0,010

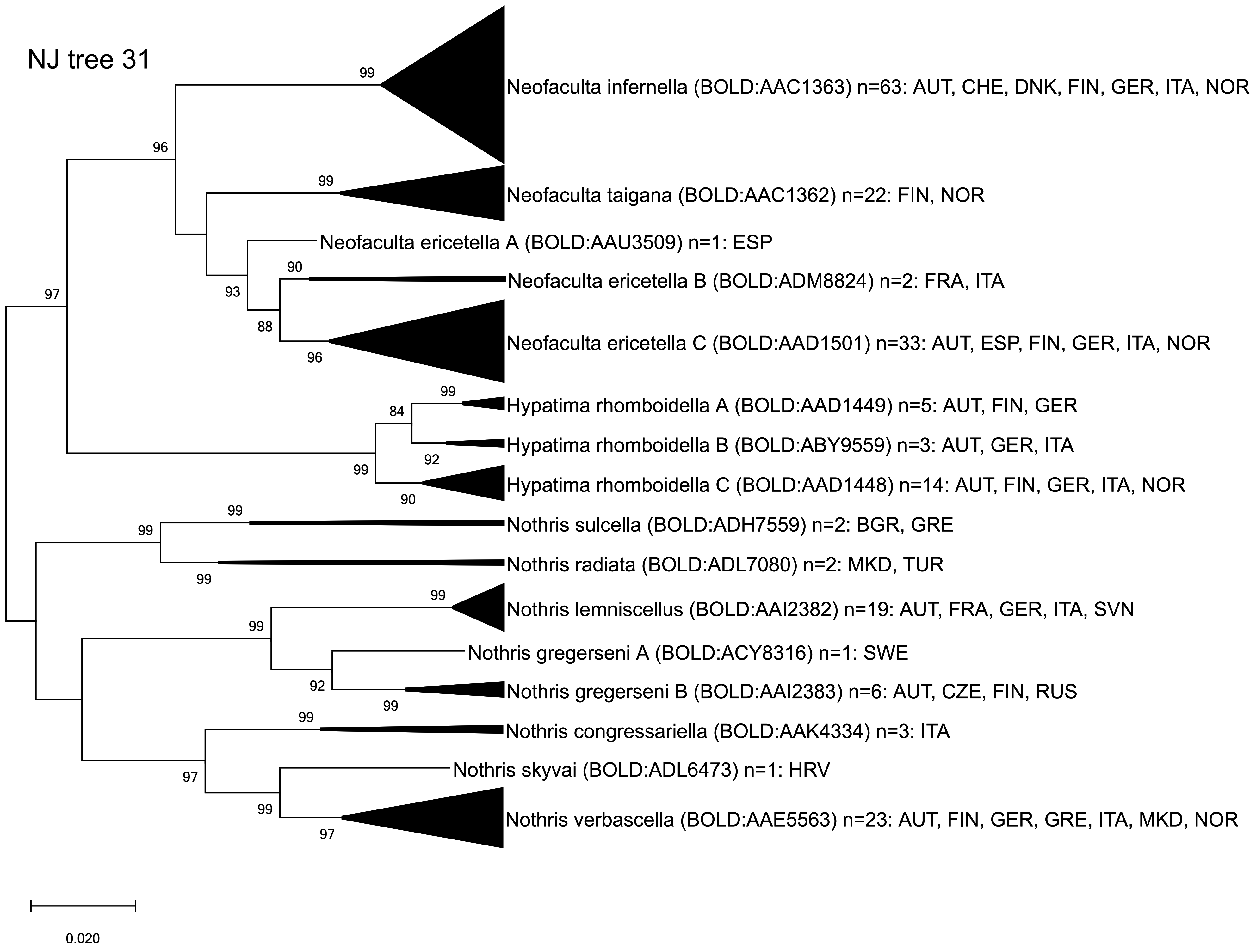
NJ tree 31
99
Neofaculta infernella (BOLD:AAC1363) n=63: AUT, CHE, DNK, FIN, GER, ITA, NOR
96
99
Neofaculta taigana (BOLD:AAC1362) n=22: FIN, NOR
Neofaculta ericetella A (BOLD:AAU3509) n=1: ESP
90
Neofaculta ericetella B (BOLD:ADM8824) n=2: FRA, ITA
97
93
88
Neofaculta ericetella C (BOLD:AAD1501) n=33: AUT, ESP, FIN, GER, ITA, NOR
96
99
84
Hypatima rhomboidella A (BOLD:AAD1449) n=5: AUT, FIN, GER
Hypatima rhomboidella B (BOLD:ABY9559) n=3: AUT, GER, ITA
92
99
Hypatima rhomboidella C (BOLD:AAD1448) n=14: AUT, FIN, GER, ITA, NOR
90
99
99
Nothris sulcella (BOLD:ADH7559) n=2: BGR, GRE
Nothris radiata (BOLD:ADL7080) n=2: MKD, TUR
99
99
Nothris lemniscellus (BOLD:AAI2382) n=19: AUT, FRA, GER, ITA, SVN
99
Nothris gregerseni A (BOLD:ACY8316) n=1: SWE
92
Nothris gregerseni B (BOLD:AAI2383) n=6: AUT, CZE, FIN, RUS
99
99
Nothris congressariella (BOLD:AAK4334) n=3: ITA
97
Nothris skyvai (BOLD:ADL6473) n=1: HRV
99
Nothris verbascella (BOLD:AAE5563) n=23: AUT, FIN, GER, GRE, ITA, MKD, NOR
97
0.020

NJ tree 32

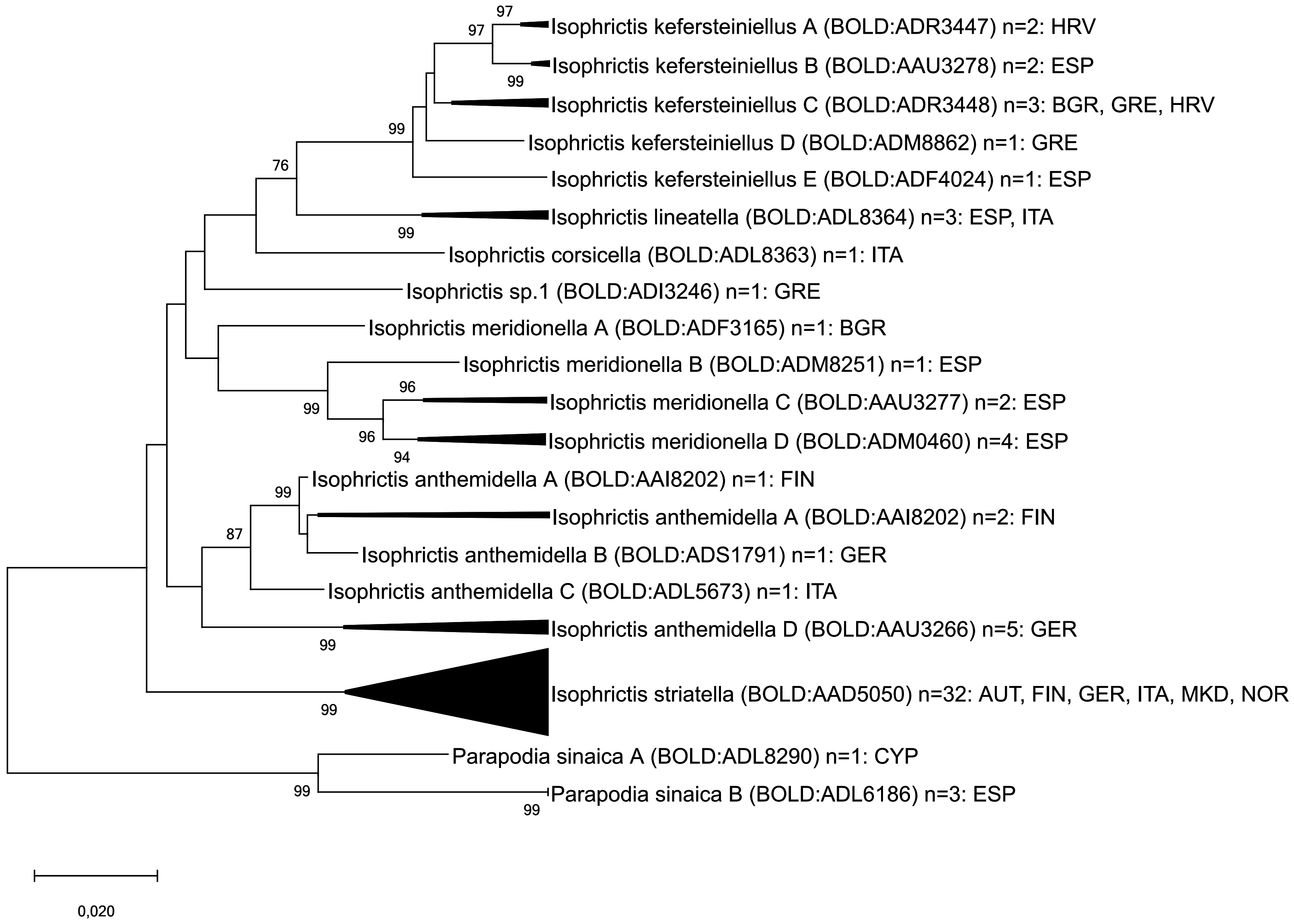

97
97
Isophrictis kefersteiniellus A (BOLD:ADR3447) n=2: HRV
Isophrictis kefersteiniellus B (BOLD:AAU3278) n=2: ESP
99
Isophrictis kefersteiniellus C (BOLD:ADR3448) n=3: BGR, GRE, HRV
99
Isophrictis kefersteiniellus D (BOLD:ADM8862) n=1: GRE
76
Isophrictis kefersteiniellus E (BOLD:ADF4024) n=1: ESP
Isophrictis lineatella (BOLD:ADL8364) n=3: ESP, ITA
99
Isophrictis corsicella (BOLD:ADL8363) n=1: ITA
Isophrictis sp.1 (BOLD:ADI3246) n=1: GRE
Isophrictis meridionella A (BOLD:ADF3165) n=1: BGR
Isophrictis meridionella B (BOLD:ADM8251) n=1: ESP
96
99
Isophrictis meridionella C (BOLD:AAU3277) n=2: ESP
96
Isophrictis meridionella D (BOLD:ADM0460) n=4: ESP
94
Isophrictis anthemidella A (BOLD:AAI8202) n=1: FIN
99
Isophrictis anthemidella A (BOLD:AAI8202) n=2: FIN
87
Isophrictis anthemidella B (BOLD:ADS1791) n=1: GER
Isophrictis anthemidella C (BOLD:ADL5673) n=1: ITA
Isophrictis anthemidella D (BOLD:AAU3266) n=5: GER
99
Isophrictis striatella (BOLD:AAD5050) n=32: AUT, FIN, GER, ITA, MKD, NOR
99
Parapodia sinaica A (BOLD:ADL8290) n=1: CYP
99
Parapodia sinaica B (BOLD:ADL6186) n=3: ESP
99
0,020

NJ tree 33

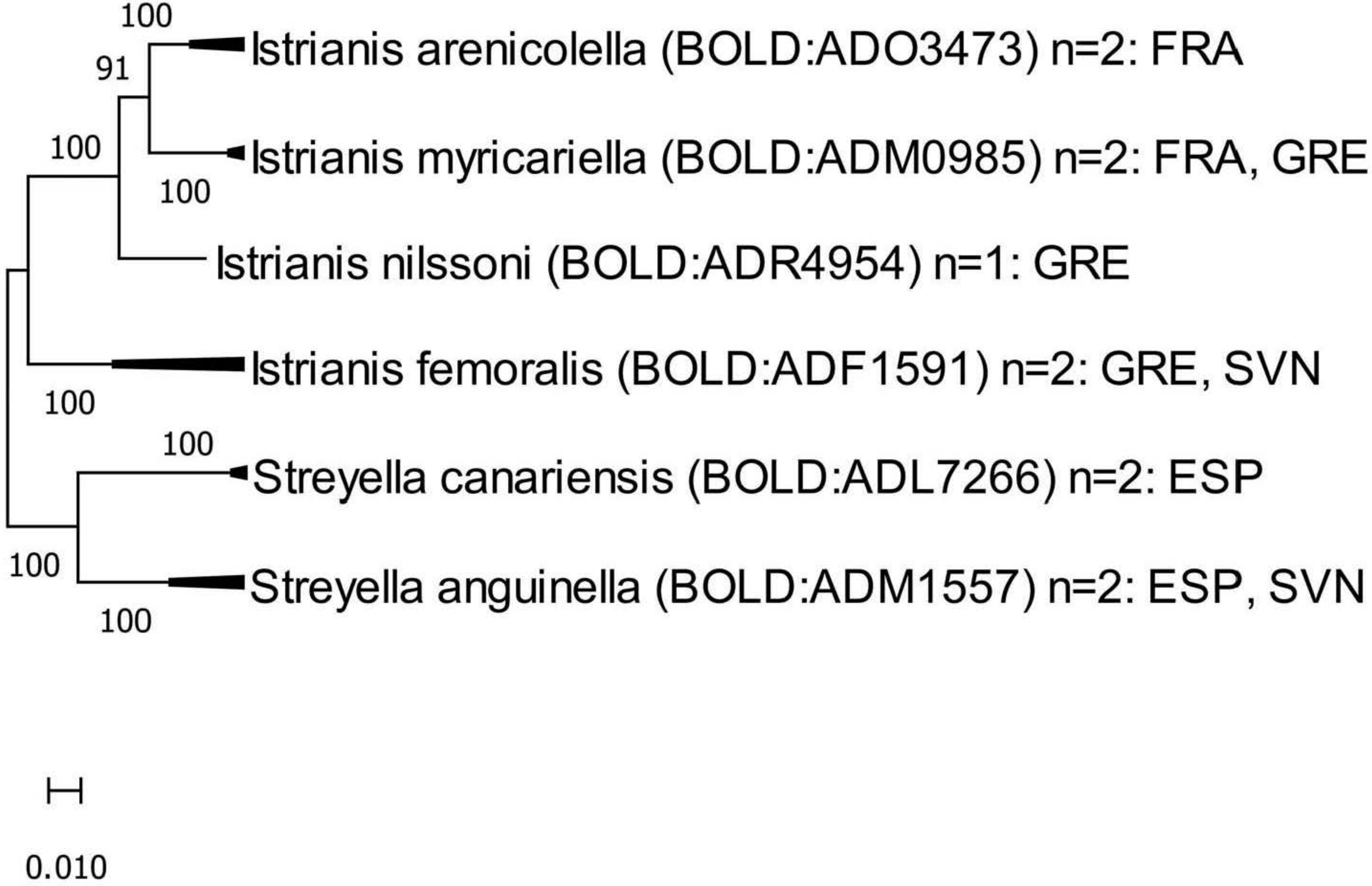

100
91
Istrianis arenicolella (BOLD:ADO3473) n=2: FRA
100
Istrianis myricariella (BOLD:ADM0985) n=2: FRA, GRE
100
Istrianis nilssoni (BOLD:ADR4954) n=1: GRE
Istrianis femoralis (BOLD:ADF1591) n=2: GRE, SVN
100
100
Streyella canariensis (BOLD:ADL7266) n=2: ESP
100
Streyella anguinella (BOLD:ADM1557) n=2: ESP, SVN
100
0.010

NJ tree 34

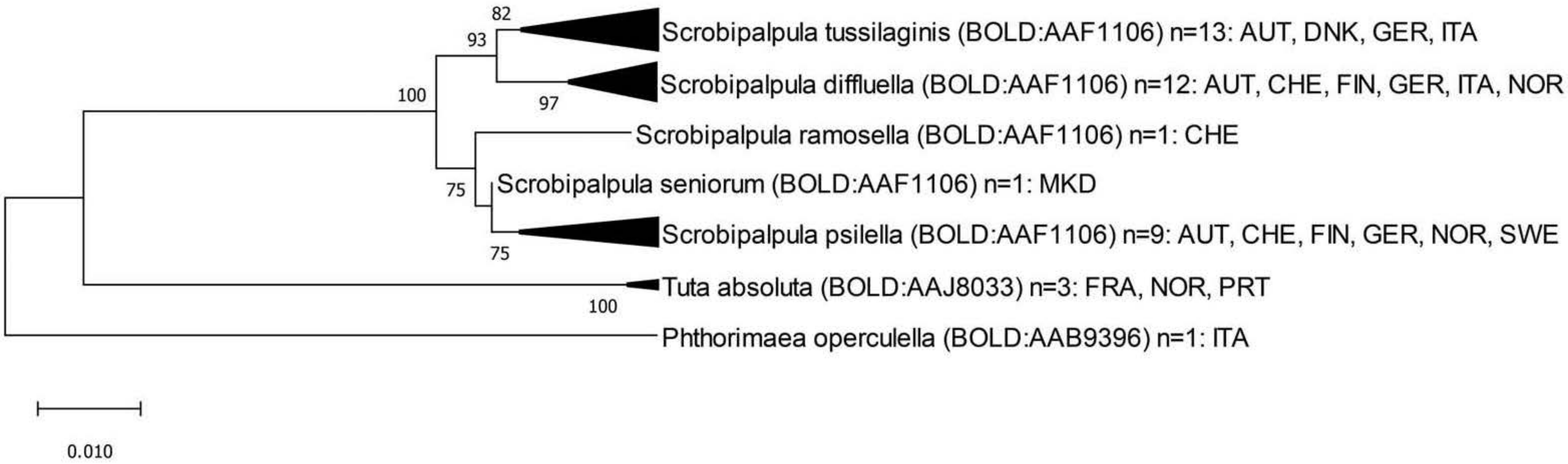

82
93
100
97
75
75
100
Scrobipalpula tussilaginis (BOLD:AAF1106) n=13: AUT, DNK, GER, ITA
Scrobipalpula diffluella (BOLD:AAF1106) n=12: AUT, CHE, FIN, GER, ITA, NOR
Scrobipalpula ramosella (BOLD:AAF1106) n=1: CHE
Scrobipalpula seniorum (BOLD:AAF1106) n=1: MKD
Scrobipalpula psilella (BOLD:AAF1106) n=9: AUT, CHE, FIN, GER, NOR, SWE
Tuta absoluta (BOLD:AAJ8033) n=3: FRA, NOR, PRT
Phthorimaea operculella (BOLD:AAB9396) n=1: ITA
0.010

NJ tree 35

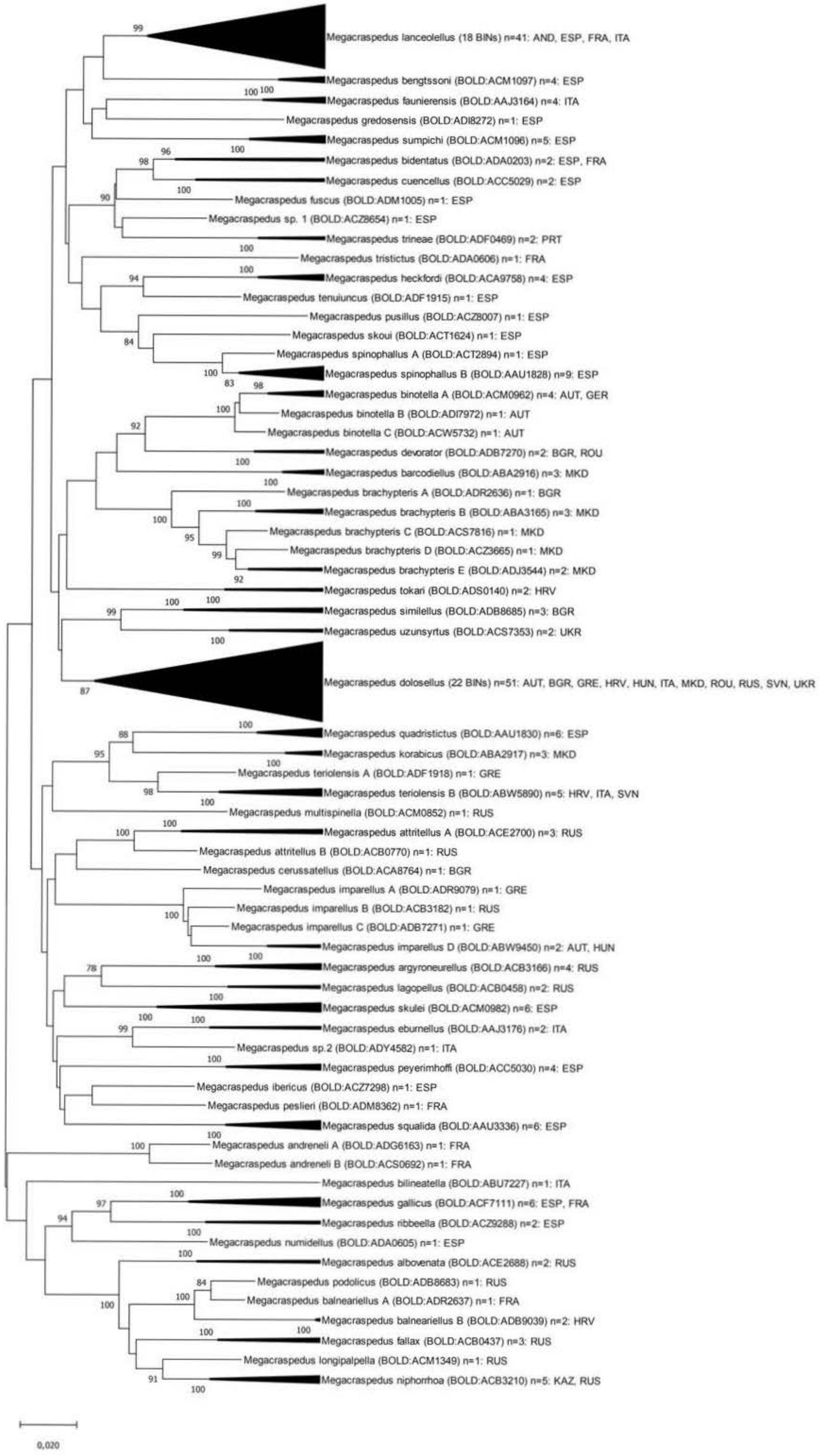
Megacraspedus lanceolellus (18 BINs) n=41: AND, ESP, FRA, ITA
Megacraspedus bengtssoni (BOLD:ACM1097) n=4: ESP
Megacraspedus faunierensis (BOLD:AAJ3164) n=4: ITA
Megacraspedus gredosensis (BOLD:ADI8272) n=1: ESP
Megacraspedus sumpichi (BOLD:ACM1096) n=5: ESP
Megacraspedus bidentatus (BOLD:ADA0203) n=2: ESP, FRA
Megacraspedus cuencellus (BOLD:ACC5029) n=2: ESP
Megacraspedus fuscus (BOLD:ADM1005) n=1: ESP
Megacraspedus sp. 1 (BOLD:ACZ8654) n=1: ESP
Megacraspedus trineae (BOLD:ADF0469) n=2: PRT
Megacraspedus tristictus (BOLD:ADA0606) n=1: FRA
Megacraspedus heckfordi (BOLD:ACA9758) n=4: ESP
Megacraspedus tenuiuncus (BOLD:ADF1915) n=1: ESP
Megacraspedus pusillus (BOLD:ACZ8007) n=1: ESP
Megacraspedus skoui (BOLD:ACT1624) n=1: ESP
Megacraspedus spinophallus A (BOLD:ACT2894) n=1: ESP
Megacraspedus spinophallus B (BOLD:AAU1828) n=9: ESP
Megacraspedus binotella A (BOLD:ACM0962) n=4: AUT, GER
Megacraspedus binotella B (BOLD:ADI7972) n=1: AUT
Megacraspedus binotella C (BOLD:ACW5732) n=1: AUT
Megacraspedus devorator (BOLD:ADB7270) n=2: BGR, ROU
Megacraspedus barcodiellus (BOLD:ABA2916) n=3: MKD
Megacraspedus brachypteris A (BOLD:ADR2636) n=1: BGR
Megacraspedus brachypteris B (BOLD:ABA3165) n=3: MKD
Megacraspedus brachypteris C (BOLD:ACS7816) n=1: MKD
Megacraspedus brachypteris D (BOLD:ACZ3665) n=1: MKD
Megacraspedus brachypteris E (BOLD:ADJ3544) n=2: MKD
Megacraspedus tokari (BOLD:ADS0140) n=2: HRV
Megacraspedus similellus (BOLD:ADB8685) n=3: BGR
Megacraspedus uzunsyrtus (BOLD:ACS7353) n=2: UKR
Megacraspedus dolosellus (22 BINs) n=51: AUT, BGR, GRE, HRV, HUN, ITA, MKD, ROU, RUS, SVN, UKR
Megacraspedus quadristictus (BOLD:AAU1830) n=6: ESP
Megacraspedus korabicus (BOLD:ABA2917) n=3: MKD
Megacraspedus teriolensis A (BOLD:ADF1918) n=1: GRE
Megacraspedus teriolensis B (BOLD:ABW5890) n=5: HRV, ITA, SVN
Megacraspedus multispinella (BOLD:ACM0852) n=1: RUS
Megacraspedus attritellus A (BOLD:ACE2700) n=3: RUS
Megacraspedus attritellus B (BOLD:ACB0770) n=1: RUS
Megacraspedus cerussatellus (BOLD:ACA8764) n=1: BGR
Megacraspedus imparellus A (BOLD:ADR9079) n=1: GRE
Megacraspedus imparellus B (BOLD:ACB3182) n=1: RUS
Megacraspedus imparellus C (BOLD:ADB7271) n=1: GRE
Megacraspedus imparellus D (BOLD:ABW9450) n=2: AUT, HUN
Megacraspedus argyroneurellus (BOLD:ACB3166) n=4: RUS
Megacraspedus lagopellus (BOLD:ACB0458) n=2: RUS
Megacraspedus skulei (BOLD:ACM0982) n=6: ESP
Megacraspedus eburnellus (BOLD:AAJ3176) n=2: ITA
Megacraspedus sp.2 (BOLD:ADY4582) n=1: ITA
Megacraspedus peyerimhoffi (BOLD:ACC5030) n=4: ESP
Megacraspedus ibericus (BOLD:ACZ7298) n=1: ESP
Megacraspedus peslieri (BOLD:ADM8362) n=1: FRA
Megacraspedus squalida (BOLD:AAU3336) n=6: ESP
Megacraspedus andreneli A (BOLD:ADG6163) n=1: FRA
Megacraspedus andreneli B (BOLD:ACS0692) n=1: FRA
Megacraspedus bilineatella (BOLD:ABU7227) n=1: ITA
Megacraspedus gallicus (BOLD:ACF7111) n=6: ESP, FRA
Megacraspedus ribbeella (BOLD:ACZ9288) n=2: ESP
Megacraspedus numidellus (BOLD:ADA0605) n=1: ESP
Megacraspedus albovenata (BOLD:ACE2688) n=2: RUS
Megacraspedus podolicus (BOLD:ADB8683) n=1: RUS
Megacraspedus balneariellus A (BOLD:ADR2637) n=1: FRA
Megacraspedus balneariellus B (BOLD:ADB9039) n=2: HRV
Megacraspedus fallax (BOLD:ACB0437) n=3: RUS
Megacraspedus longipalpella (BOLD:ACM1349) n=1: RUS
Megacraspedus niphorrhoa (BOLD:ACB3210) n=5: KAZ, RUS
0,020

NJ tree 36

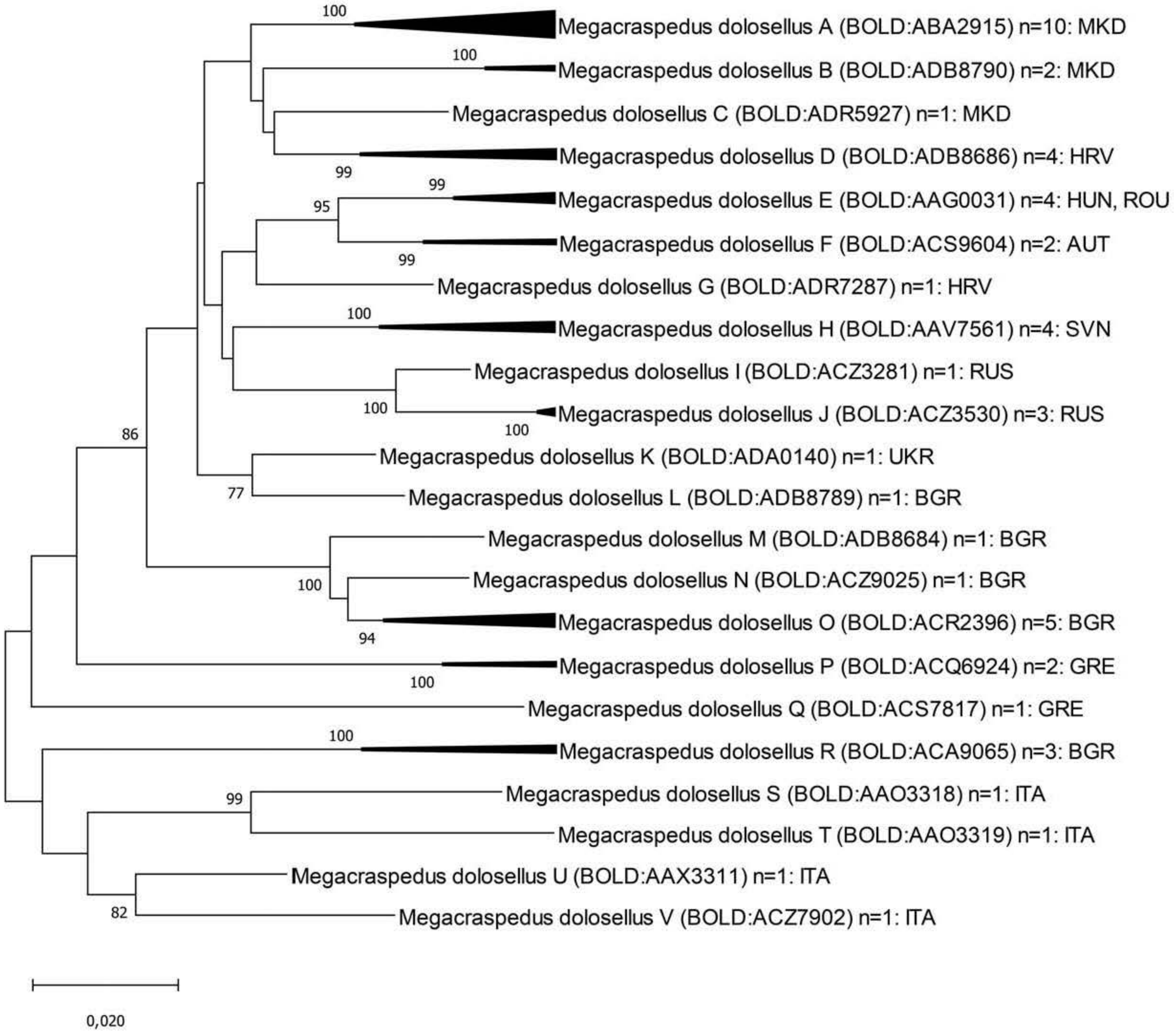

100
Megacraspedus dolosellus A (BOLD:ABA2915) n=10: MKD
100
Megacraspedus dolosellus B (BOLD:ADB8790) n=2: MKD
Megacraspedus dolosellus C (BOLD:ADR5927) n=1: MKD
99
Megacraspedus dolosellus D (BOLD:ADB8686) n=4: HRV
99
95
Megacraspedus dolosellus E (BOLD:AAG0031) n=4: HUN, ROU
99
Megacraspedus dolosellus F (BOLD:ACS9604) n=2: AUT
Megacraspedus dolosellus G (BOLD:ADR7287) n=1: HRV
100
Megacraspedus dolosellus H (BOLD:AAV7561) n=4: SVN
Megacraspedus dolosellus I (BOLD:ACZ3281) n=1: RUS
100
Megacraspedus dolosellus J (BOLD:ACZ3530) n=3: RUS
100
86
Megacraspedus dolosellus K (BOLD:ADA0140) n=1: UKR
77
Megacraspedus dolosellus L (BOLD:ADB8789) n=1: BGR
Megacraspedus dolosellus M (BOLD:ADB8684) n=1: BGR
100
Megacraspedus dolosellus N (BOLD:ACZ9025) n=1: BGR
94
Megacraspedus dolosellus O (BOLD:ACR2396) n=5: BGR
100
Megacraspedus dolosellus P (BOLD:ACQ6924) n=2: GRE
Megacraspedus dolosellus Q (BOLD:ACS7817) n=1: GRE
100
Megacraspedus dolosellus R (BOLD:ACA9065) n=3: BGR
99
Megacraspedus dolosellus S (BOLD:AAO3318) n=1: ITA
Megacraspedus dolosellus T (BOLD:AAO3319) n=1: ITA
Megacraspedus dolosellus U (BOLD:AAX3311) n=1: ITA
82
Megacraspedus dolosellus V (BOLD:ACZ7902) n=1: ITA
0,020

NJ tree 37

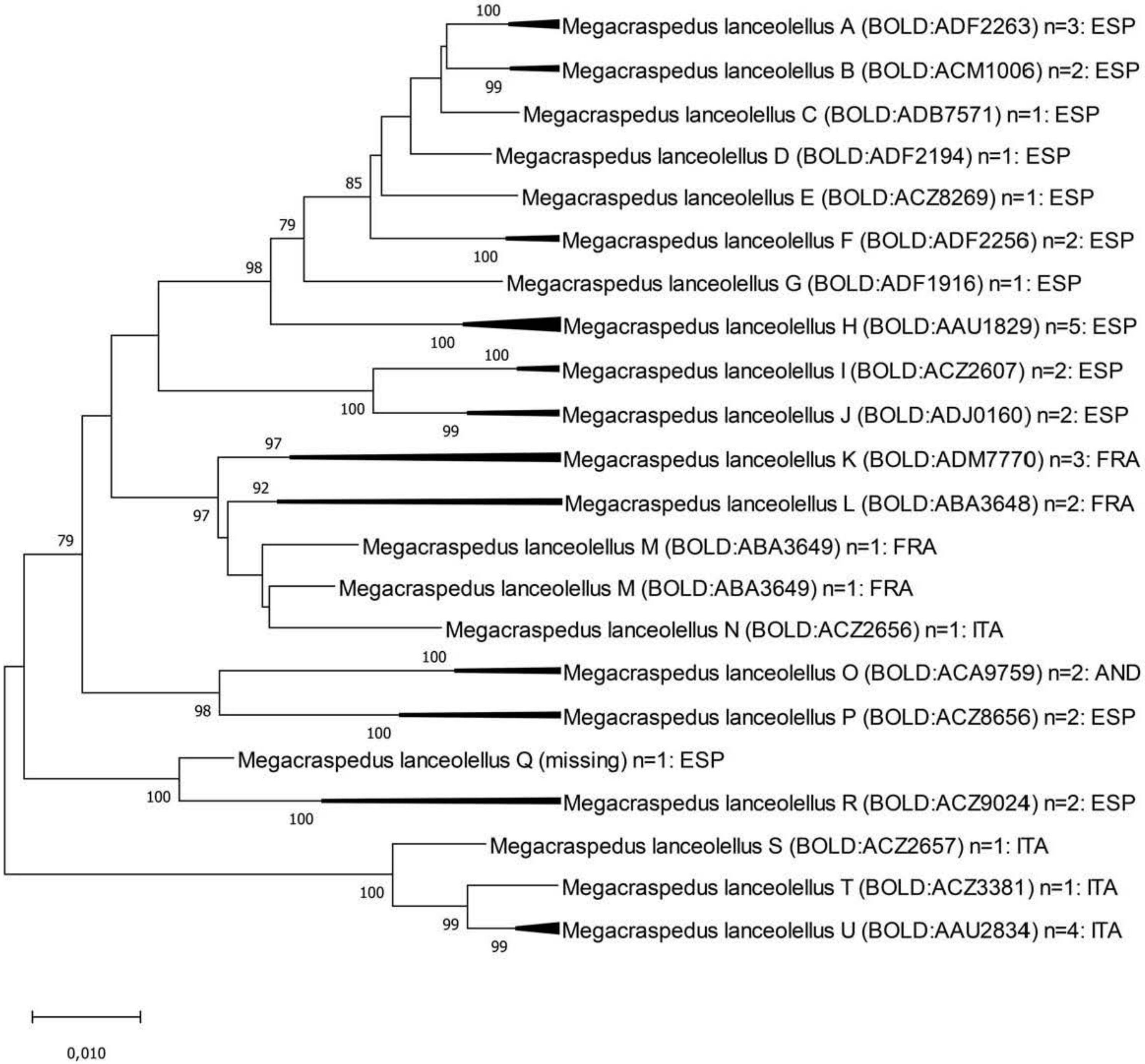
100
Megacraspedus lanceolellus A (BOLD:ADF2263) n=3: ESP
99
Megacraspedus lanceolellus B (BOLD:ACM1006) n=2: ESP
Megacraspedus lanceolellus C (BOLD:ADB7571) n=1: ESP
Megacraspedus lanceolellus D (BOLD:ADF2194) n=1: ESP
85
Megacraspedus lanceolellus E (BOLD:ACZ8269) n=1: ESP
79
Megacraspedus lanceolellus F (BOLD:ADF2256) n=2: ESP
100
98
Megacraspedus lanceolellus G (BOLD:ADF1916) n=1: ESP
Megacraspedus lanceolellus H (BOLD:AAU1829) n=5: ESP
100
100
Megacraspedus lanceolellus I (BOLD:ACZ2607) n=2: ESP
100
Megacraspedus lanceolellus J (BOLD:ADJ0160) n=2: ESP
99
97
Megacraspedus lanceolellus K (BOLD:ADM7770) n=3: FRA
92
Megacraspedus lanceolellus L (BOLD:ABA3648) n=2: FRA
97
79
Megacraspedus lanceolellus M (BOLD:ABA3649) n=1: FRA
Megacraspedus lanceolellus M (BOLD:ABA3649) n=1: FRA
Megacraspedus lanceolellus N (BOLD:ACZ2656) n=1: ITA
100
Megacraspedus lanceolellus O (BOLD:ACA9759) n=2: AND
98
Megacraspedus lanceolellus P (BOLD:ACZ8656) n=2: ESP
100
Megacraspedus lanceolellus Q (missing) n=1: ESP
100
Megacraspedus lanceolellus R (BOLD:ACZ9024) n=2: ESP
100
Megacraspedus lanceolellus S (BOLD:ACZ2657) n=1: ITA
100
Megacraspedus lanceolellus T (BOLD:ACZ3381) n=1: ITA
99
Megacraspedus lanceolellus U (BOLD:AAU2834) n=4: ITA
99
0,010

NJ tree 38

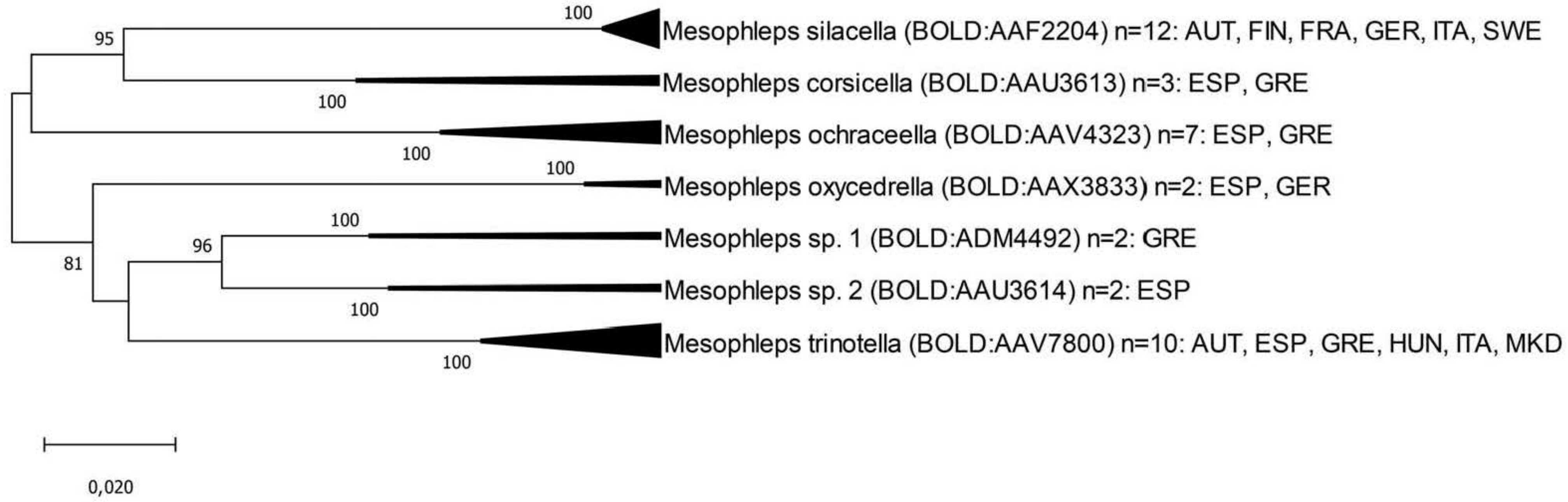
100
95
Mesophleps silacella (BOLD:AAF2204) n=12: AUT, FIN, FRA, GER, ITA, SWE
Mesophleps corsicella (BOLD:AAU3613) n=3: ESP, GRE
100
Mesophleps ochraceella (BOLD:AAV4323) n=7: ESP, GRE
100
100
Mesophleps oxycedrella (BOLD:AAX3833) n=2: ESP, GER
100
96
Mesophleps sp. 1 (BOLD:ADM4492) n=2: GRE
81
Mesophleps sp. 2 (BOLD:AAU3614) n=2: ESP
100
Mesophleps trinotella (BOLD:AAV7800) n=10: AUT, ESP, GRE, HUN, ITA, MKD
100
0,020

NJ tree 39

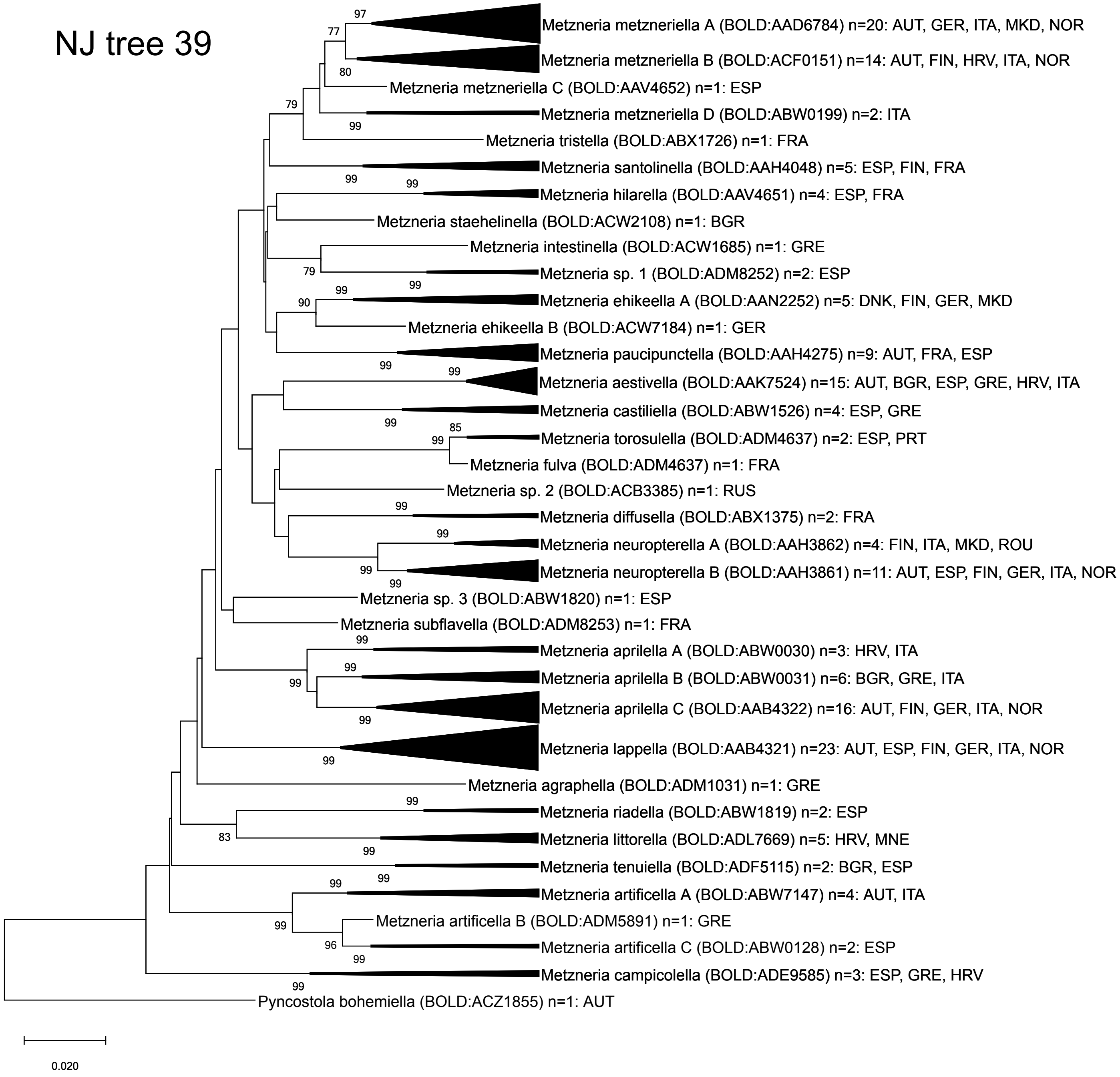
Metzneria metzneriella A (BOLD:AAD6784) n=20: AUT, GER, ITA, MKD, NOR
Metzneria metzneriella B (BOLD:ACF0151) n=14: AUT, FIN, HRV, ITA, NOR
Metzneria metzneriella C (BOLD:AAV4652) n=1: ESP
Metzneria metzneriella D (BOLD:ABW0199) n=2: ITA
Metzneria tristella (BOLD:ABX1726) n=1: FRA
Metzneria santolinella (BOLD:AAH4048) n=5: ESP, FIN, FRA
Metzneria hilarella (BOLD:AAV4651) n=4: ESP, FRA
Metzneria staehelinella (BOLD:ACW2108) n=1: BGR
Metzneria intestinella (BOLD:ACW1685) n=1: GRE
Metzneria sp. 1 (BOLD:ADM8252) n=2: ESP
Metzneria ehikeella A (BOLD:AAN2252) n=5: DNK, FIN, GER, MKD
Metzneria ehikeella B (BOLD:ACW7184) n=1: GER
Metzneria paucipunctella (BOLD:AAH4275) n=9: AUT, FRA, ESP
Metzneria aestivella (BOLD:AAK7524) n=15: AUT, BGR, ESP, GRE, HRV, ITA
Metzneria castiliella (BOLD:ABW1526) n=4: ESP, GRE
Metzneria torosulella (BOLD:ADM4637) n=2: ESP, PRT
Metzneria fulva (BOLD:ADM4637) n=1: FRA
Metzneria sp. 2 (BOLD:ACB3385) n=1: RUS
Metzneria diffusella (BOLD:ABX1375) n=2: FRA
Metzneria neuropterella A (BOLD:AAH3862) n=4: FIN, ITA, MKD, ROU
Metzneria neuropterella B (BOLD:AAH3861) n=11: AUT, ESP, FIN, GER, ITA, NOR
Metzneria sp. 3 (BOLD:ABW1820) n=1: ESP
Metzneria subflavella (BOLD:ADM8253) n=1: FRA
Metzneria aprilella A (BOLD:ABW0030) n=3: HRV, ITA
Metzneria aprilella B (BOLD:ABW0031) n=6: BGR, GRE, ITA
Metzneria aprilella C (BOLD:AAB4322) n=16: AUT, FIN, GER, ITA, NOR
Metzneria lappella (BOLD:AAB4321) n=23: AUT, ESP, FIN, GER, ITA, NOR
Metzneria agraphella (BOLD:ADM1031) n=1: GRE
Metzneria riadella (BOLD:ABW1819) n=2: ESP
Metzneria littorella (BOLD:ADL7669) n=5: HRV, MNE
Metzneria tenuiella (BOLD:ADF5115) n=2: BGR, ESP
Metzneria artificella A (BOLD:ABW7147) n=4: AUT, ITA
Metzneria artificella B (BOLD:ADM5891) n=1: GRE
Metzneria artificella C (BOLD:ABW0128) n=2: ESP
Metzneria campicolella (BOLD:ADE9585) n=3: ESP, GRE, HRV
Pyncostola bohemiella (BOLD:ACZ1855) n=1: AUT
0.020

NJ tree 40

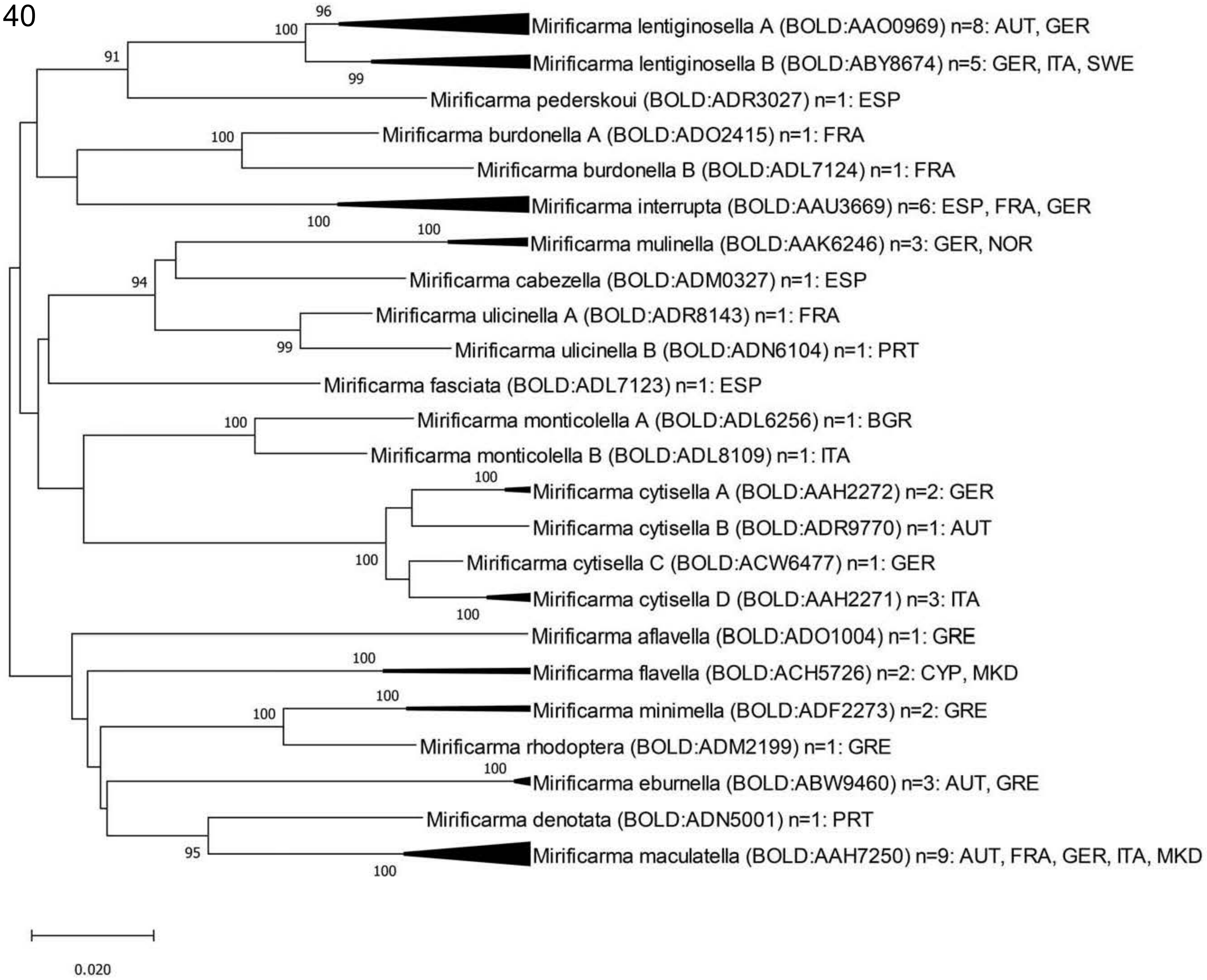

Mirificarma lentiginosella A (BOLD:AAO0969) n=8: AUT, GER
Mirificarma lentiginosella B (BOLD:ABY8674) n=5: GER, ITA, SWE
Mirificarma pederskoui (BOLD:ADR3027) n=1: ESP
Mirificarma burdonella A (BOLD:ADO2415) n=1: FRA
Mirificarma burdonella B (BOLD:ADL7124) n=1: FRA
Mirificarma interrupta (BOLD:AAU3669) n=6: ESP, FRA, GER
Mirificarma mulinella (BOLD:AAK6246) n=3: GER, NOR
Mirificarma cabezella (BOLD:ADM0327) n=1: ESP
Mirificarma ulicinella A (BOLD:ADR8143) n=1: FRA
Mirificarma ulicinella B (BOLD:ADN6104) n=1: PRT
Mirificarma fasciata (BOLD:ADL7123) n=1: ESP
Mirificarma monticolella A (BOLD:ADL6256) n=1: BGR
Mirificarma monticolella B (BOLD:ADL8109) n=1: ITA
Mirificarma cytisella A (BOLD:AAH2272) n=2: GER
Mirificarma cytisella B (BOLD:ADR9770) n=1: AUT
Mirificarma cytisella C (BOLD:ACW6477) n=1: GER
Mirificarma cytisella D (BOLD:AAH2271) n=3: ITA
Mirificarma aflavella (BOLD:ADO1004) n=1: GRE
Mirificarma flavella (BOLD:ACH5726) n=2: CYP, MKD
Mirificarma minimella (BOLD:ADF2273) n=2: GRE
Mirificarma rhodoptera (BOLD:ADM2199) n=1: GRE
Mirificarma eburnella (BOLD:ABW9460) n=3: AUT, GRE
Mirificarma denotata (BOLD:ADN5001) n=1: PRT
Mirificarma maculatella (BOLD:AAH7250) n=9: AUT, FRA, GER, ITA, MKD
96
100
91
99
100
100
100
94
99
100
100
100
100
100
100
100
100
100
100
95
100
0.020

NJ tree 41

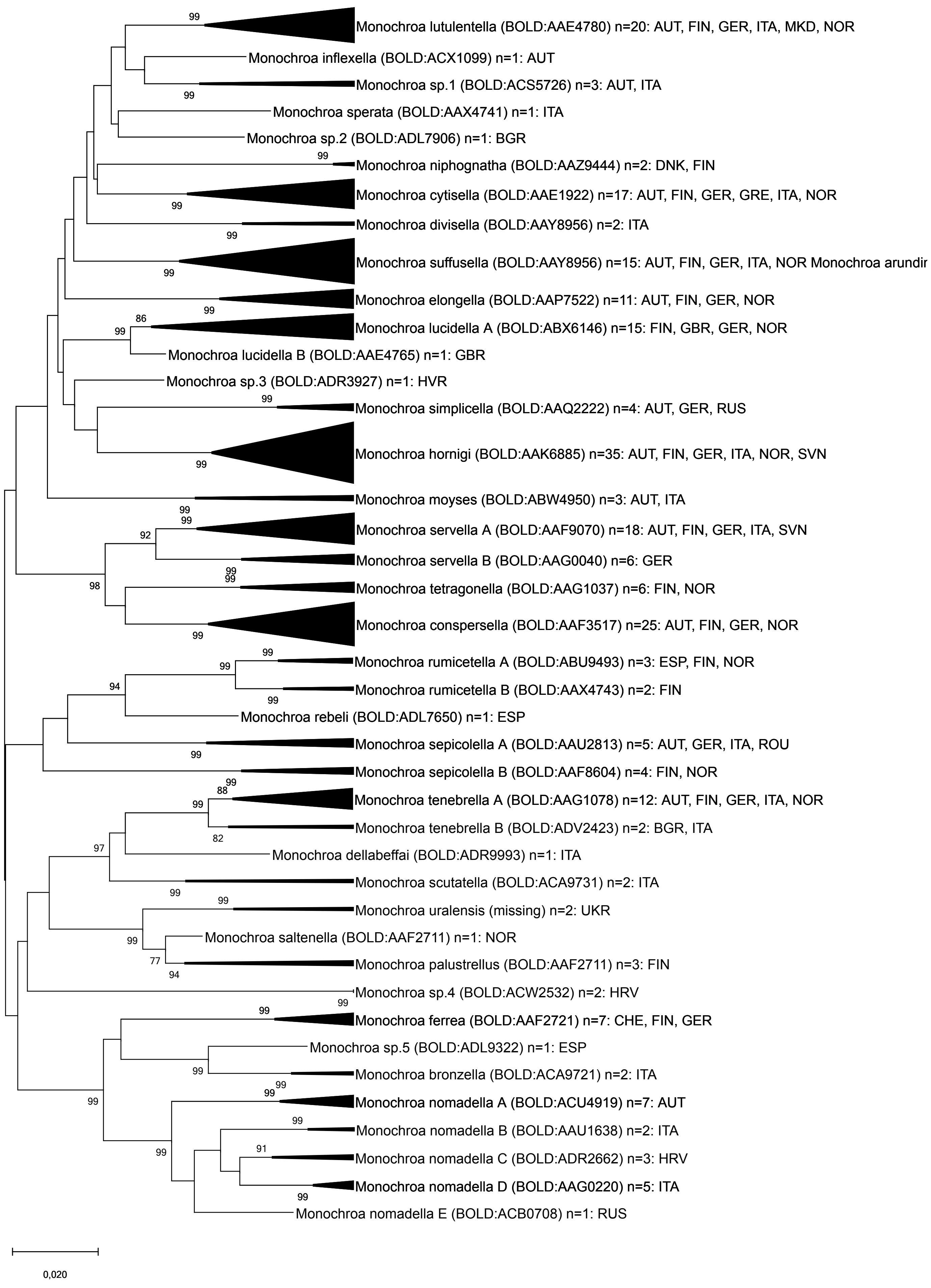
Monochroa lutulentella (BOLD:AAE4780) n=20: AUT, FIN, GER, ITA, MKD, NOR
Monochroa inflexella (BOLD:ACX1099) n=1: AUT
Monochroa sp.1 (BOLD:ACS5726) n=3: AUT, ITA
Monochroa sperata (BOLD:AAX4741) n=1: ITA
Monochroa sp.2 (BOLD:ADL7906) n=1: BGR
Monochroa niphognatha (BOLD:AAZ9444) n=2: DNK, FIN
Monochroa cytisella (BOLD:AAE1922) n=17: AUT, FIN, GER, GRE, ITA, NOR
Monochroa divisella (BOLD:AAY8956) n=2: ITA
Monochroa suffusella (BOLD:AAY8956) n=15: AUT, FIN, GER, ITA, NOR Monochroa arundir
Monochroa elongella (BOLD:AAP7522) n=11: AUT, FIN, GER, NOR
Monochroa lucidella A (BOLD:ABX6146) n=15: FIN, GBR, GER, NOR
Monochroa lucidella B (BOLD:AAE4765) n=1: GBR
Monochroa sp.3 (BOLD:ADR3927) n=1: HVR
Monochroa simplicella (BOLD:AAQ2222) n=4: AUT, GER, RUS
Monochroa hornigi (BOLD:AAK6885) n=35: AUT, FIN, GER, ITA, NOR, SVN
Monochroa moyses (BOLD:ABW4950) n=3: AUT, ITA
Monochroa servella A (BOLD:AAF9070) n=18: AUT, FIN, GER, ITA, SVN
Monochroa servella B (BOLD:AAG0040) n=6: GER
Monochroa tetragonella (BOLD:AAG1037) n=6: FIN, NOR
Monochroa conspersella (BOLD:AAF3517) n=25: AUT, FIN, GER, NOR
Monochroa rumicetella A (BOLD:ABU9493) n=3: ESP, FIN, NOR
Monochroa rumicetella B (BOLD:AAX4743) n=2: FIN
Monochroa rebeli (BOLD:ADL7650) n=1: ESP
Monochroa sepicolella A (BOLD:AAU2813) n=5: AUT, GER, ITA, ROU
Monochroa sepicolella B (BOLD:AAF8604) n=4: FIN, NOR
Monochroa tenebrella A (BOLD:AAG1078) n=12: AUT, FIN, GER, ITA, NOR
Monochroa tenebrella B (BOLD:ADV2423) n=2: BGR, ITA
Monochroa dellabeffai (BOLD:ADR9993) n=1: ITA
Monochroa scutatella (BOLD:ACA9731) n=2: ITA
Monochroa uralensis (missing) n=2: UKR
Monochroa saltenella (BOLD:AAF2711) n=1: NOR
Monochroa palustrellus (BOLD:AAF2711) n=3: FIN
Monochroa sp.4 (BOLD:ACW2532) n=2: HRV
Monochroa ferrea (BOLD:AAF2721) n=7: CHE, FIN, GER
Monochroa sp.5 (BOLD:ADL9322) n=1: ESP
Monochroa bronzella (BOLD:ACA9721) n=2: ITA
Monochroa nomadella A (BOLD:ACU4919) n=7: AUT
Monochroa nomadella B (BOLD:AAU1638) n=2: ITA
Monochroa nomadella C (BOLD:ADR2662) n=3: HRV
Monochroa nomadella D (BOLD:AAG0220) n=5: ITA
Monochroa nomadella E (BOLD:ACB0708) n=1: RUS
0,020

NJ tree 42

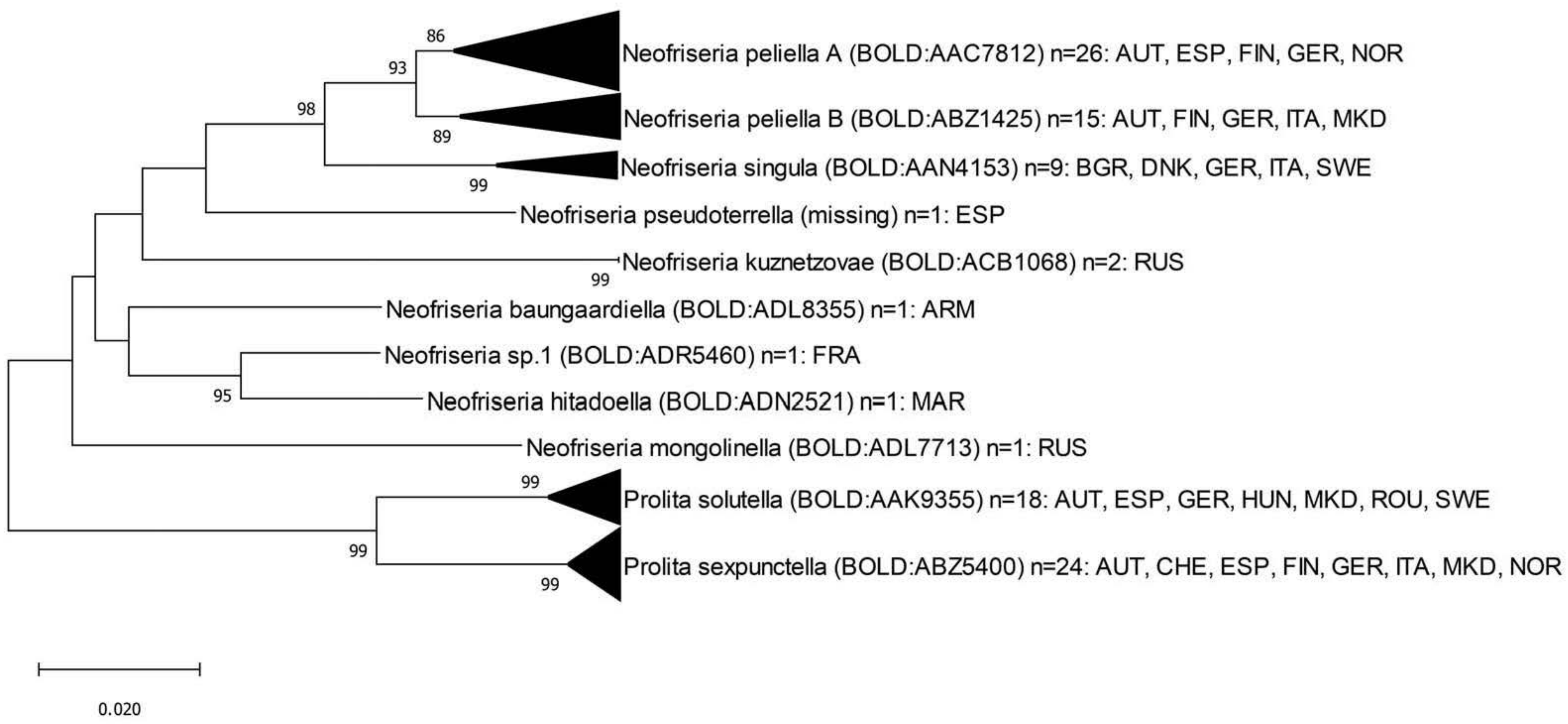
86
93
98
89
99
99
95
99
99
99
Neofriseria peliella A (BOLD:AAC7812) n=26: AUT, ESP, FIN, GER, NOR
Neofriseria peliella B (BOLD:ABZ1425) n=15: AUT, FIN, GER, ITA, MKD
Neofriseria singula (BOLD:AAN4153) n=9: BGR, DNK, GER, ITA, SWE
Neofriseria pseudoterrella (missing) n=1: ESP
Neofriseria kuznetzovae (BOLD:ACB1068) n=2: RUS
Neofriseria baungaardiella (BOLD:ADL8355) n=1: ARM
Neofriseria sp.1 (BOLD:ADR5460) n=1: FRA
Neofriseria hitadoella (BOLD:ADN2521) n=1: MAR
Neofriseria mongolinella (BOLD:ADL7713) n=1: RUS
Prolita solutella (BOLD:AAK9355) n=18: AUT, ESP, GER, HUN, MKD, ROU, SWE
Prolita sexpunctella (BOLD:ABZ5400) n=24: AUT, CHE, ESP, FIN, GER, ITA, MKD, NOR
0.020

NJ tree 43

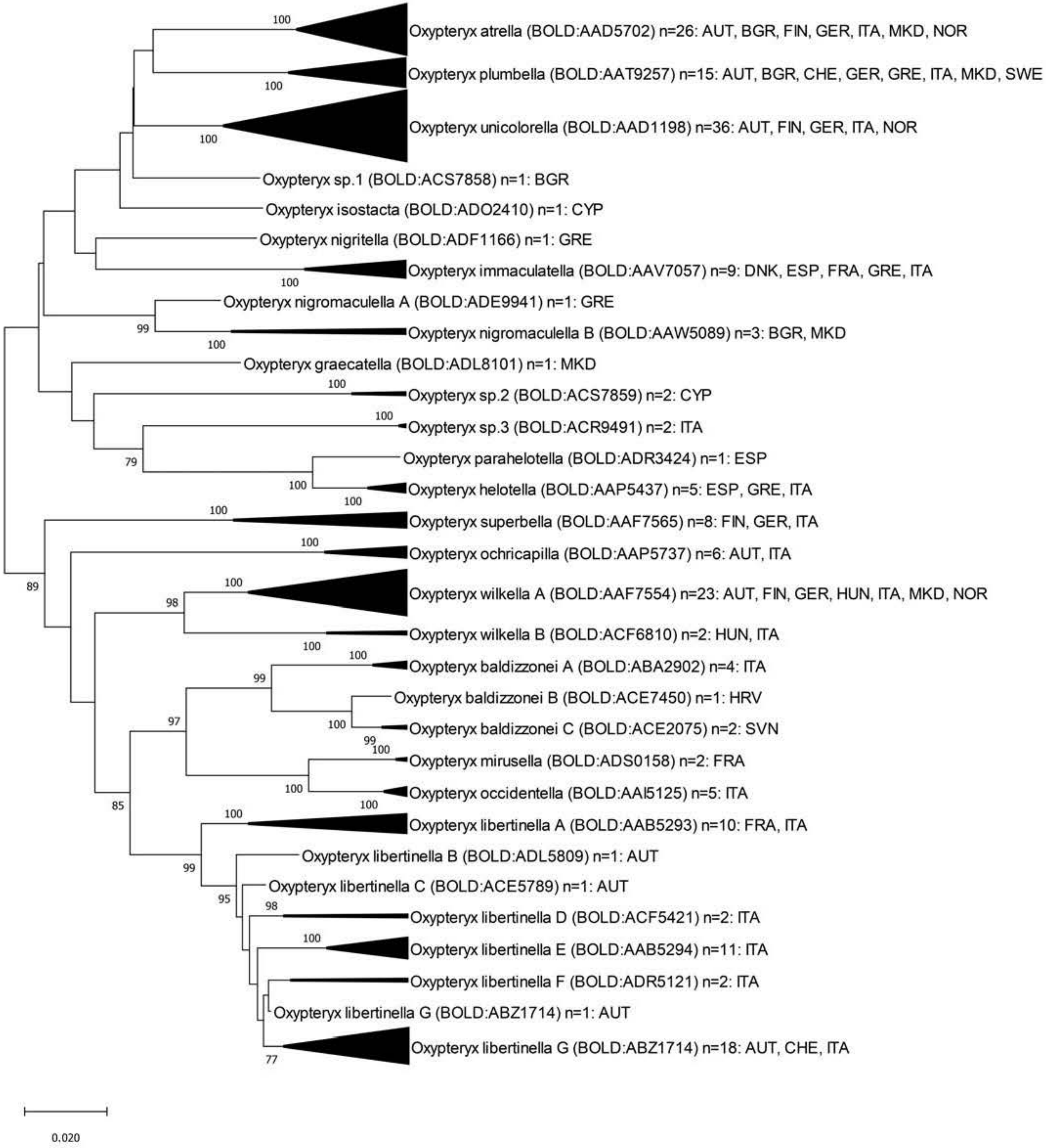

100
Oxypteryx atrella (BOLD:AAD5702) n=26: AUT, BGR, FIN, GER, ITA, MKD, NOR
100
Oxypteryx plumbella (BOLD:AAT9257) n=15: AUT, BGR, CHE, GER, GRE, ITA, MKD, SWE
100
Oxypteryx unicolorella (BOLD:AAD1198) n=36: AUT, FIN, GER, ITA, NOR
Oxypteryx sp.1 (BOLD:ACS7858) n=1: BGR
Oxypteryx isostacta (BOLD:ADO2410) n=1: CYP
Oxypteryx nigritella (BOLD:ADF1166) n=1: GRE
100
Oxypteryx immaculatella (BOLD:AAV7057) n=9: DNK, ESP, FRA, GRE, ITA
Oxypteryx nigromaculella A (BOLD:ADE9941) n=1: GRE
99
100
Oxypteryx nigromaculella B (BOLD:AAW5089) n=3: BGR, MKD
Oxypteryx graecatella (BOLD:ADL8101) n=1: MKD
100
Oxypteryx sp.2 (BOLD:ACS7859) n=2: CYP
100
Oxypteryx sp.3 (BOLD:ACR9491) n=2: ITA
79
Oxypteryx parahelotella (BOLD:ADR3424) n=1: ESP
100
100
Oxypteryx helotella (BOLD:AAP5437) n=5: ESP, GRE, ITA
100
Oxypteryx superbella (BOLD:AAF7565) n=8: FIN, GER, ITA
100
Oxypteryx ochricapilla (BOLD:AAP5737) n=6: AUT, ITA
89
100
98
Oxypteryx wilkella A (BOLD:AAF7554) n=23: AUT, FIN, GER, HUN, ITA, MKD, NOR
Oxypteryx wilkella B (BOLD:ACF6810) n=2: HUN, ITA
100
100
Oxypteryx baldizzonei A (BOLD:ABA2902) n=4: ITA
99
Oxypteryx baldizzonei B (BOLD:ACE7450) n=1: HRV
97
100
Oxypteryx baldizzonei C (BOLD:ACE2075) n=2: SVN
99
100
Oxypteryx mirusella (BOLD:ADS0158) n=2: FRA
100
Oxypteryx occidentella (BOLD:AAI5125) n=5: ITA
100
85
100
Oxypteryx libertinella A (BOLD:AAB5293) n=10: FRA, ITA
Oxypteryx libertinella B (BOLD:ADL5809) n=1: AUT
99
Oxypteryx libertinella C (BOLD:ACE5789) n=1: AUT
95
98
Oxypteryx libertinella D (BOLD:ACF5421) n=2: ITA
100
Oxypteryx libertinella E (BOLD:AAB5294) n=11: ITA
Oxypteryx libertinella F (BOLD:ADR5121) n=2: ITA
Oxypteryx libertinella G (BOLD:ABZ1714) n=1: AUT
Oxypteryx libertinella G (BOLD:ABZ1714) n=18: AUT, CHE, ITA
77
0.020

NJ tree 44

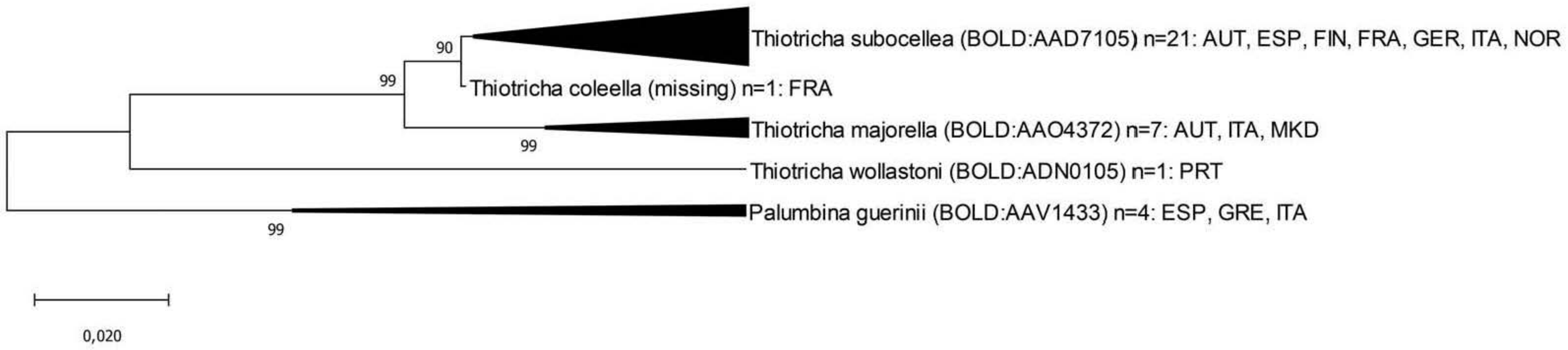

Thiotricha subocellea (BOLD:AAD7105) n=21: AUT, ESP, FIN, FRA, GER, ITA, NOR
90
99
Thiotricha coleella (missing) n=1: FRA
Thiotricha majorella (BOLD:AAO4372) n=7: AUT, ITA, MKD
99
Thiotricha wollastoni (BOLD:ADN0105) n=1: PRT
Palumbina guerinii (BOLD:AAV1433) n=4: ESP, GRE, ITA
99
0,020

NJ tree 45

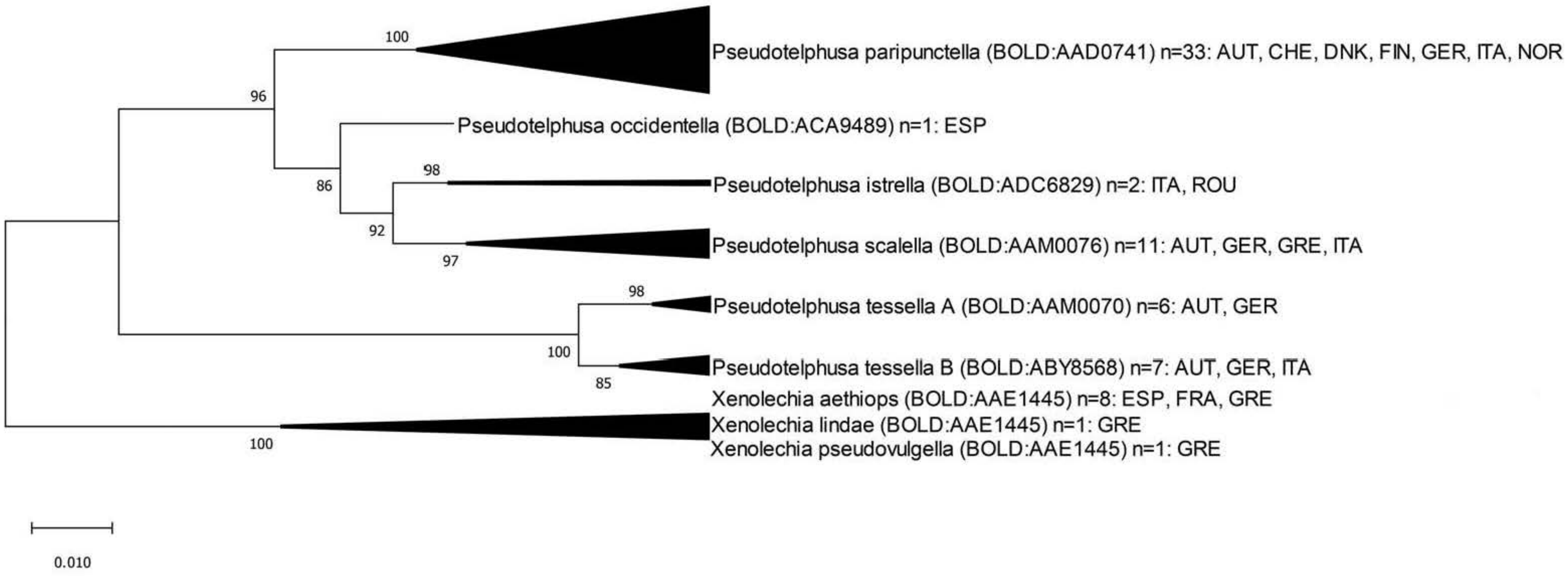
Pseudotelphusa paripunctella (BOLD:AAD0741) n=33: AUT, CHE, DNK, FIN, GER, ITA, NOR
Pseudotelphusa occidentella (BOLD:ACA9489) n=1: ESP
Pseudotelphusa istrella (BOLD:ADC6829) n=2: ITA, ROU
Pseudotelphusa scalella (BOLD:AAM0076) n=11: AUT, GER, GRE, ITA
Pseudotelphusa tessella A (BOLD:AAM0070) n=6: AUT, GER
Pseudotelphusa tessella B (BOLD:ABY8568) n=7: AUT, GER, ITA
Xenolechia aethiops (BOLD:AAE1445) n=8: ESP, FRA, GRE
Xenolechia lindae (BOLD:AAE1445) n=1: GRE
Xenolechia pseudovulgella (BOLD:AAE1445) n=1: GRE
100
96
86
98
92
97
98
100
85
100
0.010

NJ tree 46

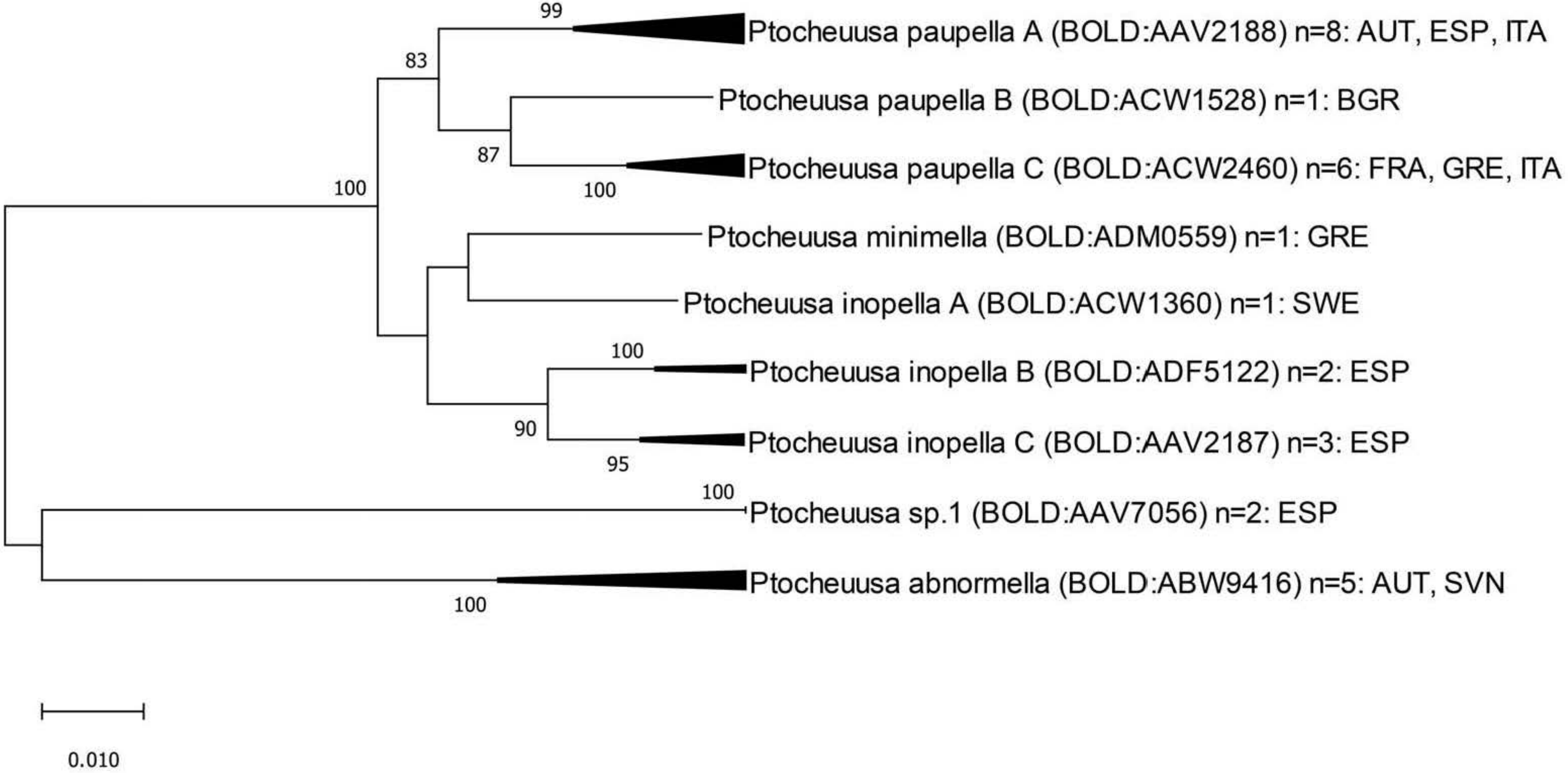
99
83
Ptocheuusa paupella A (BOLD:AAV2188) n=8: AUT, ESP, ITA
Ptocheuusa paupella B (BOLD:ACW1528) n=1: BGR
87
Ptocheuusa paupella C (BOLD:ACW2460) n=6: FRA, GRE, ITA
100
100
Ptocheuusa minimella (BOLD:ADM0559) n=1: GRE
Ptocheuusa inopella A (BOLD:ACW1360) n=1: SWE
100
Ptocheuusa inopella B (BOLD:ADF5122) n=2: ESP
90
Ptocheuusa inopella C (BOLD:AAV2187) n=3: ESP
95
100
Ptocheuusa sp.1 (BOLD:AAV7056) n=2: ESP
Ptocheuusa abnormella (BOLD:ABW9416) n=5: AUT, SVN
100
0.010

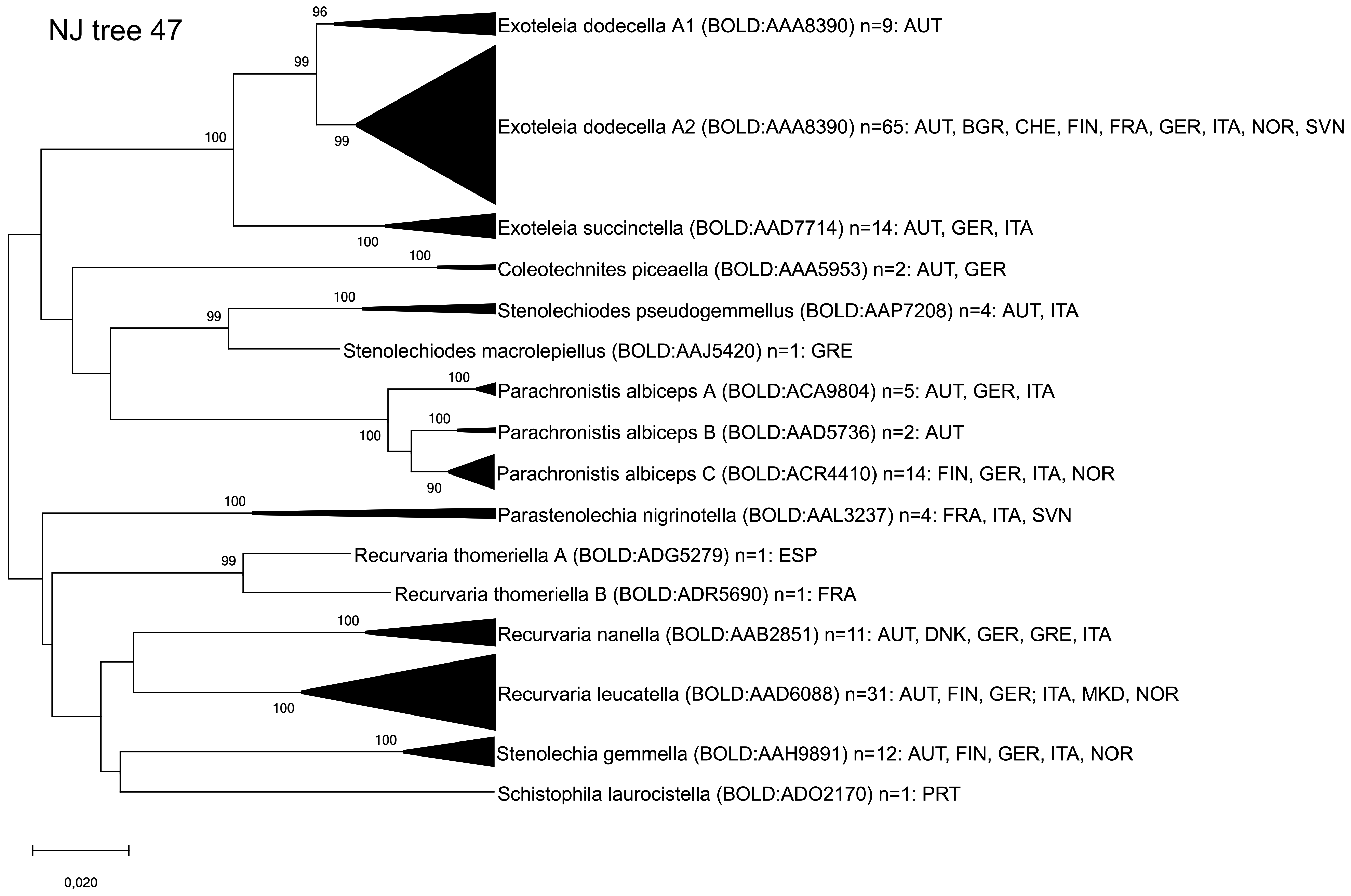
NJ tree 47
96
99
100
99
100
100
99
100
100
100
100
90
100
99
100
100
100
Exoteleia dodecella A1 (BOLD:AAA8390) n=9: AUT
Exoteleia dodecella A2 (BOLD:AAA8390) n=65: AUT, BGR, CHE, FIN, FRA, GER, ITA, NOR, SVN
Exoteleia succinctella (BOLD:AAD7714) n=14: AUT, GER, ITA
Coleotechnites piceaella (BOLD:AAA5953) n=2: AUT, GER
Stenolechiodes pseudogemmellus (BOLD:AAP7208) n=4: AUT, ITA
Stenolechiodes macrolepiellus (BOLD:AAJ5420) n=1: GRE
Parachronistis albiceps A (BOLD:ACA9804) n=5: AUT, GER, ITA
Parachronistis albiceps B (BOLD:AAD5736) n=2: AUT
Parachronistis albiceps C (BOLD:ACR4410) n=14: FIN, GER, ITA, NOR
Parastenolechia nigrinotella (BOLD:AAL3237) n=4: FRA, ITA, SVN
Recurvaria thomeriella A (BOLD:ADG5279) n=1: ESP
Recurvaria thomeriella B (BOLD:ADR5690) n=1: FRA
Recurvaria nanella (BOLD:AAB2851) n=11: AUT, DNK, GER, GRE, ITA
Recurvaria leucatella (BOLD:AAD6088) n=31: AUT, FIN, GER; ITA, MKD, NOR
Stenolechia gemmella (BOLD:AAH9891) n=12: AUT, FIN, GER, ITA, NOR
Schistophila laurocistella (BOLD:ADO2170) n=1: PRT
0,020

NJ tree 48

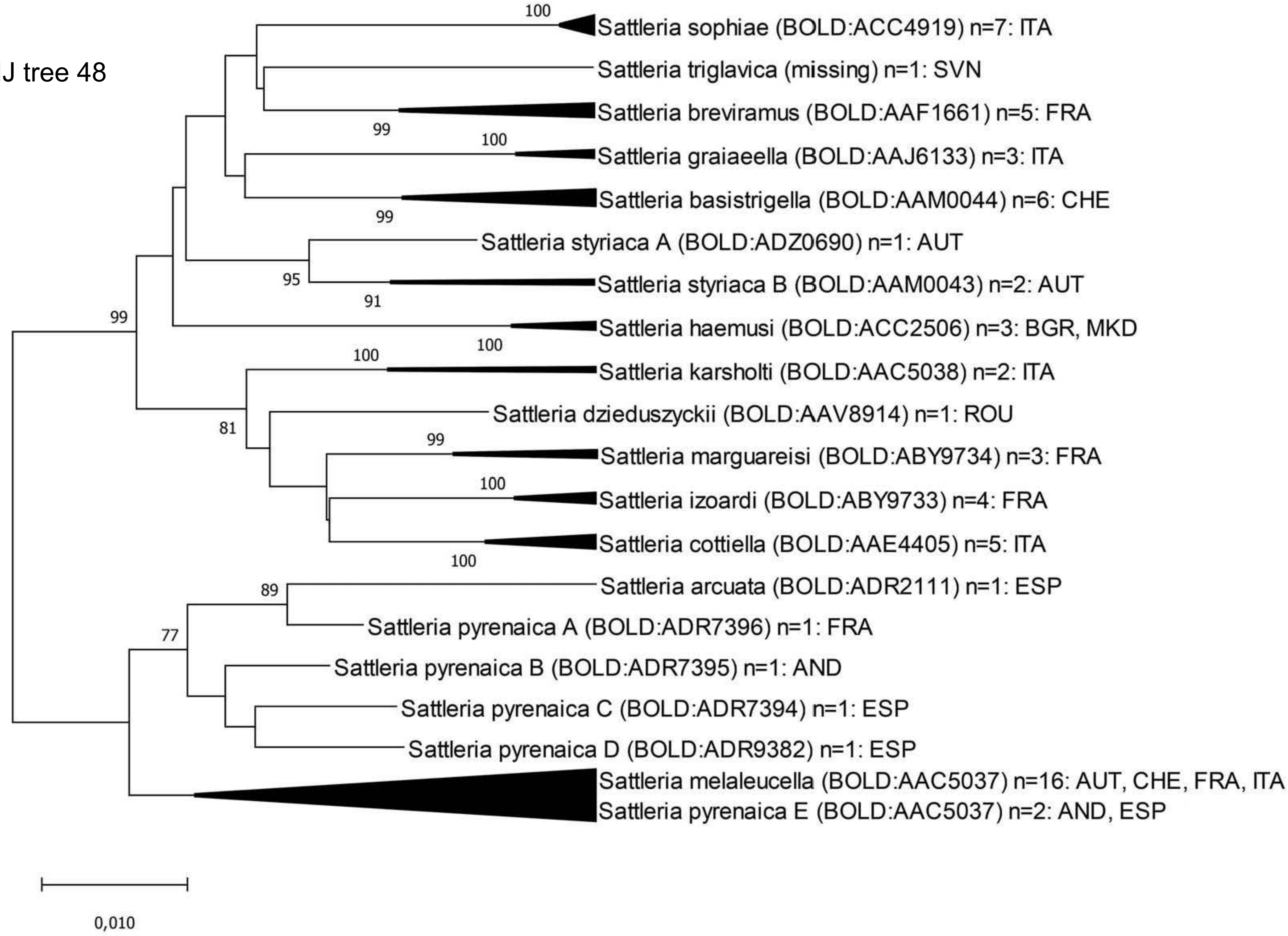

100
Sattleria sophiae (BOLD:ACC4919) n=7: ITA
Sattleria triglavica (missing) n=1: SVN
99
Sattleria breviramus (BOLD:AAF1661) n=5: FRA
100
Sattleria graiaeella (BOLD:AAJ6133) n=3: ITA
99
Sattleria basistrigella (BOLD:AAM0044) n=6: CHE
Sattleria styriaca A (BOLD:ADZ0690) n=1: AUT
95
91
Sattleria styriaca B (BOLD:AAM0043) n=2: AUT
99
100
Sattleria haemusi (BOLD:ACC2506) n=3: BGR, MKD
100
Sattleria karsholti (BOLD:AAC5038) n=2: ITA
81
Sattleria dzieduszyckii (BOLD:AAV8914) n=1: ROU
99
Sattleria marguareisi (BOLD:ABY9734) n=3: FRA
100
Sattleria izoardi (BOLD:ABY9733) n=4: FRA
100
Sattleria cottiella (BOLD:AAE4405) n=5: ITA
89
Sattleria arcuata (BOLD:ADR2111) n=1: ESP
77
Sattleria pyrenaica A (BOLD:ADR7396) n=1: FRA
Sattleria pyrenaica B (BOLD:ADR7395) n=1: AND
Sattleria pyrenaica C (BOLD:ADR7394) n=1: ESP
Sattleria pyrenaica D (BOLD:ADR9382) n=1: ESP
Sattleria melaleucella (BOLD:AAC5037) n=16: AUT, CHE, FRA, ITA
Sattleria pyrenaica E (BOLD:AAC5037) n=2: AND, ESP
0,010

NJ tree 49

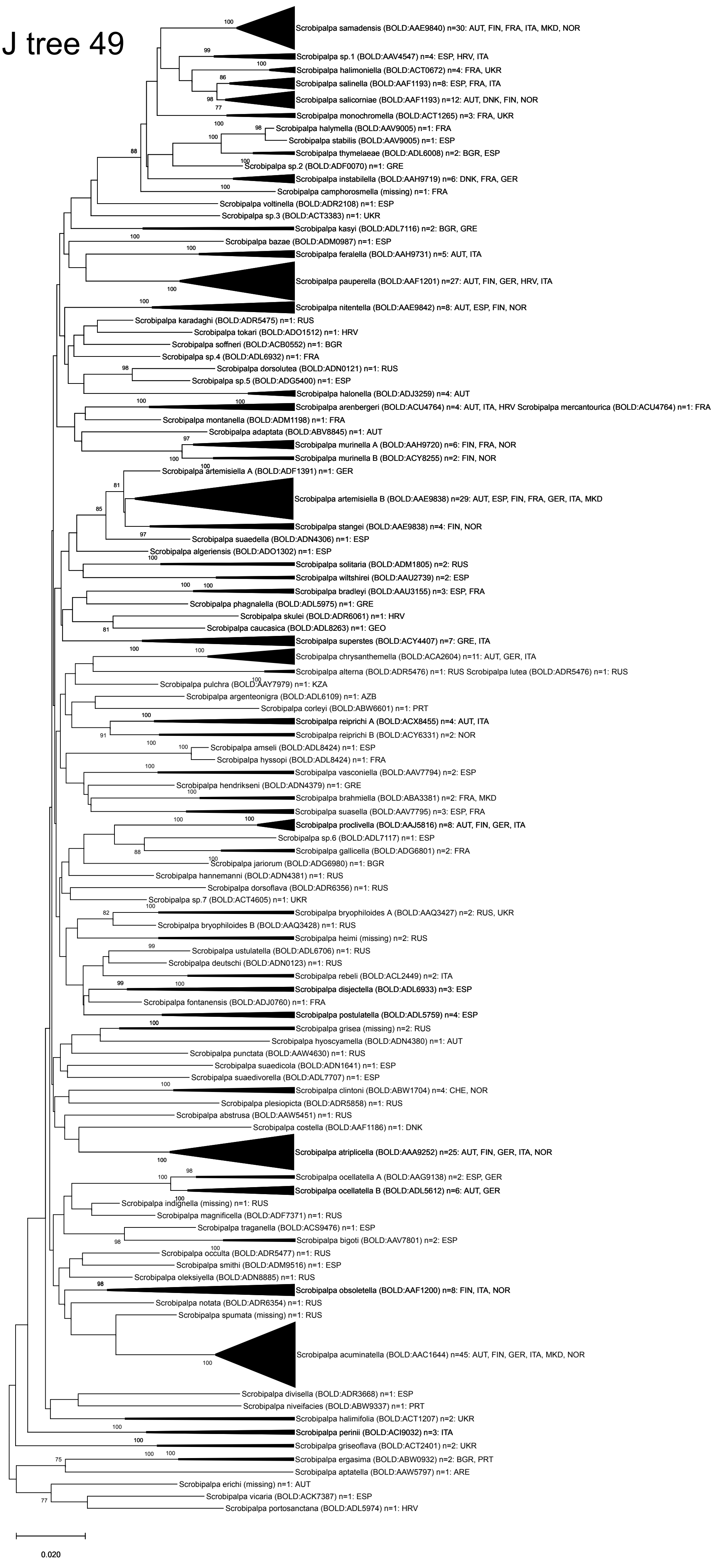
Scrobipalpa samadensis (BOLD:AAE9840) n=30: AUT, FIN, FRA, ITA, MKD, NOR
Scrobipalpa sp.1 (BOLD:AAV4547) n=4: ESP, HRV, ITA
Scrobipalpa halimoniella (BOLD:ACT0672) n=4: FRA, UKR
Scrobipalpa salinella (BOLD:AAF1193) n=8: ESP, FRA, ITA
Scrobipalpa salicorniae (BOLD:AAF1193) n=12: AUT, DNK, FIN, NOR
Scrobipalpa monochromella (BOLD:ACT1265) n=3: FRA, UKR
Scrobipalpa halymella (BOLD:AAV9005) n=1: FRA
Scrobipalpa stabilis (BOLD:AAV9005) n=1: ESP
Scrobipalpa thymelaeae (BOLD:ADL6008) n=2: BGR, ESP
Scrobipalpa sp.2 (BOLD:ADF0070) n=1: GRE
Scrobipalpa instabilella (BOLD:AAH9719) n=6: DNK, FRA, GER
Scrobipalpa camphorosmella (missing) n=1: FRA
Scrobipalpa voltinella (BOLD:ADR2108) n=1: ESP
Scrobipalpa sp.3 (BOLD:ACT3383) n=1: UKR
Scrobipalpa kasyi (BOLD:ADL7116) n=2: BGR, GRE
Scrobipalpa bazae (BOLD:ADM0987) n=1: ESP
Scrobipalpa feralella (BOLD:AAH9731) n=5: AUT, ITA
Scrobipalpa pauperella (BOLD:AAF1201) n=27: AUT, FIN, GER, HRV, ITA
Scrobipalpa nitentella (BOLD:AAE9842) n=8: AUT, ESP, FIN, NOR
Scrobipalpa karadaghi (BOLD:ADR5475) n=1: RUS
Scrobipalpa tokari (BOLD:ADO1512) n=1: HRV
Scrobipalpa soffneri (BOLD:ACB0552) n=1: BGR
Scrobipalpa sp.4 (BOLD:ADL6932) n=1: FRA
Scrobipalpa dorsolutea (BOLD:ADN0121) n=1: RUS
Scrobipalpa sp.5 (BOLD:ADG5400) n=1: ESP
Scrobipalpa halonella (BOLD:ADJ3259) n=4: AUT
Scrobipalpa arenbergeri (BOLD:ACU4764) n=4: AUT, ITA, HRV Scrobipalpa mercantourica (BOLD:ACU4764) n=1: FRA
Scrobipalpa montanella (BOLD:ADM1198) n=1: FRA
Scrobipalpa adaptata (BOLD:ABV8845) n=1: AUT
Scrobipalpa murinella A (BOLD:AAH9720) n=6: FIN, FRA, NOR
Scrobipalpa murinella B (BOLD:ACY8255) n=2: FIN, NOR
Scrobipalpa artemisiella A (BOLD:ADF1391) n=1: GER
Scrobipalpa artemisiella B (BOLD:AAE9838) n=29: AUT, ESP, FIN, FRA, GER, ITA, MKD
Scrobipalpa stangei (BOLD:AAE9838) n=4: FIN, NOR
Scrobipalpa suaedella (BOLD:ADN4306) n=1: ESP
Scrobipalpa algeriensis (BOLD:ADO1302) n=1: ESP
Scrobipalpa solitaria (BOLD:ADM1805) n=2: RUS
Scrobipalpa wiltshirei (BOLD:AAU2739) n=2: ESP
Scrobipalpa bradleyi (BOLD:AAU3155) n=3: ESP, FRA
Scrobipalpa phagnalella (BOLD:ADL5975) n=1: GRE
Scrobipalpa skulei (BOLD:ADR6061) n=1: HRV
Scrobipalpa caucasica (BOLD:ADL8263) n=1: GEO
Scrobipalpa superstes (BOLD:ACY4407) n=7: GRE, ITA
Scrobipalpa chrysanthemella (BOLD:ACA2604) n=11: AUT, GER, ITA
Scrobipalpa alterna (BOLD:ADR5476) n=1: RUS Scrobipalpa lutea (BOLD:ADR5476) n=1: RUS
Scrobipalpa pulchra (BOLD:AAY7979) n=1: KZA
Scrobipalpa argenteonigra (BOLD:ADL6109) n=1: AZB
Scrobipalpa corleyi (BOLD:ABW6601) n=1: PRT
Scrobipalpa reiprichi A (BOLD:ACX8455) n=4: AUT, ITA
Scrobipalpa reiprichi B (BOLD:ACY6331) n=2: NOR
Scrobipalpa amseli (BOLD:ADL8424) n=1: ESP
Scrobipalpa hyssopi (BOLD:ADL8424) n=1: FRA
Scrobipalpa vasconiella (BOLD:AAV7794) n=2: ESP
Scrobipalpa hendrikseni (BOLD:ADN4379) n=1: GRE
Scrobipalpa brahmiella (BOLD:ABA3381) n=2: FRA, MKD
Scrobipalpa suasella (BOLD:AAV7795) n=3: ESP, FRA
Scrobipalpa proclivella (BOLD:AAJ5816) n=8: AUT, FIN, GER, ITA
Scrobipalpa sp.6 (BOLD:ADL7117) n=1: ESP
Scrobipalpa gallicella (BOLD:ADG6801) n=2: FRA
Scrobipalpa jariorum (BOLD:ADG6980) n=1: BGR
Scrobipalpa hannemanni (BOLD:ADN4381) n=1: RUS
Scrobipalpa dorsoflava (BOLD:ADR6356) n=1: RUS
Scrobipalpa sp.7 (BOLD:ACT4605) n=1: UKR
Scrobipalpa bryophiloides A (BOLD:AAQ3427) n=2: RUS, UKR
Scrobipalpa bryophiloides B (BOLD:AAQ3428) n=1: RUS
Scrobipalpa heimi (missing) n=2: RUS
Scrobipalpa ustulatella (BOLD:ADL6706) n=1: RUS
Scrobipalpa deutschi (BOLD:ADN0123) n=1: RUS
Scrobipalpa rebeli (BOLD:ACL2449) n=2: ITA
Scrobipalpa disjectella (BOLD:ADL6933) n=3: ESP
Scrobipalpa fontanensis (BOLD:ADJ0760) n=1: FRA
Scrobipalpa postulatella (BOLD:ADL5759) n=4: ESP
Scrobipalpa grisea (missing) n=2: RUS
Scrobipalpa hyoscyamella (BOLD:ADN4380) n=1: AUT
Scrobipalpa punctata (BOLD:AAW4630) n=1: RUS
Scrobipalpa suaedicola (BOLD:ADN1641) n=1: ESP
Scrobipalpa suaedivorella (BOLD:ADL7707) n=1: ESP
Scrobipalpa clintoni (BOLD:ABW1704) n=4: CHE, NOR
Scrobipalpa plesiopicta (BOLD:ADR5858) n=1: RUS
Scrobipalpa abstrusa (BOLD:AAW5451) n=1: RUS
Scrobipalpa costella (BOLD:AAF1186) n=1: DNK
Scrobipalpa atriplicella (BOLD:AAA9252) n=25: AUT, FIN, GER, ITA, NOR
Scrobipalpa ocellatella A (BOLD:AAG9138) n=2: ESP, GER
Scrobipalpa ocellatella B (BOLD:ADL5612) n=6: AUT, GER
Scrobipalpa indignella (missing) n=1: RUS
Scrobipalpa magnificella (BOLD:ADF7371) n=1: RUS
Scrobipalpa traganella (BOLD:ACS9476) n=1: ESP
Scrobipalpa bigoti (BOLD:AAV7801) n=2: ESP
Scrobipalpa occulta (BOLD:ADR5477) n=1: RUS
Scrobipalpa smithi (BOLD:ADM9516) n=1: ESP
Scrobipalpa oleksiyella (BOLD:ADN8885) n=1: RUS
Scrobipalpa obsoletella (BOLD:AAF1200) n=8: FIN, ITA, NOR
Scrobipalpa notata (BOLD:ADR6354) n=1: RUS
Scrobipalpa spumata (missing) n=1: RUS
Scrobipalpa acuminatella (BOLD:AAC1644) n=45: AUT, FIN, GER, ITA, MKD, NOR
Scrobipalpa divisella (BOLD:ADR3668) n=1: ESP
Scrobipalpa niveifacies (BOLD:ABW9337) n=1: PRT
Scrobipalpa halimifolia (BOLD:ACT1207) n=2: UKR
Scrobipalpa perinii (BOLD:ACI9032) n=3: ITA
Scrobipalpa griseoflava (BOLD:ACT2401) n=2: UKR
Scrobipalpa ergasima (BOLD:ABW0932) n=2: BGR, PRT
Scrobipalpa aptatella (BOLD:AAW5797) n=1: ARE
Scrobipalpa erichi (missing) n=1: AUT
Scrobipalpa vicaria (BOLD:ACK7387) n=1: ESP
Scrobipalpa portosanctana (BOLD:ADL5974) n=1: HRV
0.020

NJ tree 50

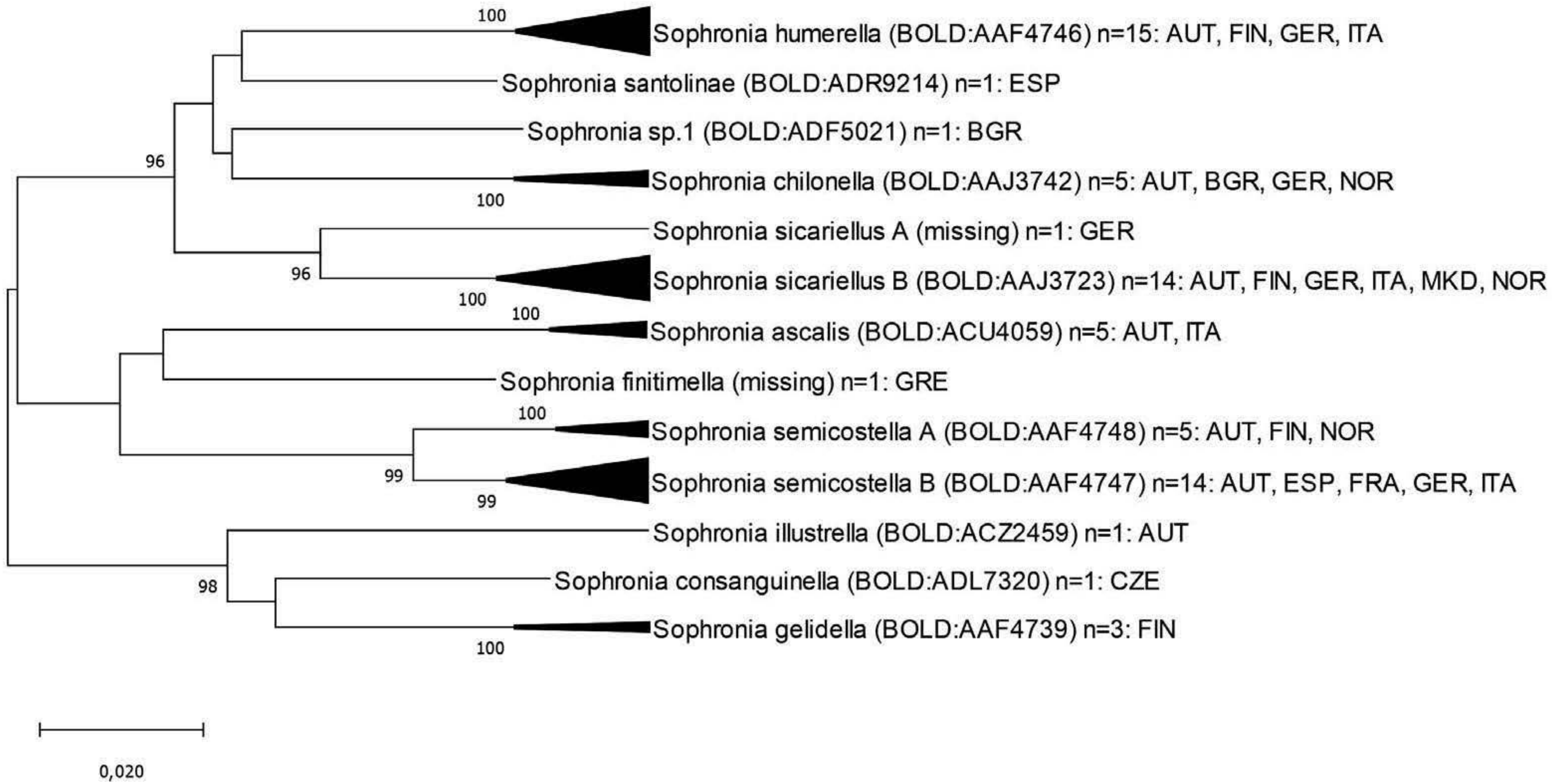
100
Sophronia humerella (BOLD:AAF4746) n=15: AUT, FIN, GER, ITA
Sophronia santolinae (BOLD:ADR9214) n=1: ESP
Sophronia sp.1 (BOLD:ADF5021) n=1: BGR
96
Sophronia chilonella (BOLD:AAJ3742) n=5: AUT, BGR, GER, NOR
100
Sophronia sicariellus A (missing) n=1: GER
96
Sophronia sicariellus B (BOLD:AAJ3723) n=14: AUT, FIN, GER, ITA, MKD, NOR
100
100
Sophronia ascalis (BOLD:ACU4059) n=5: AUT, ITA
Sophronia finitimella (missing) n=1: GRE
100
Sophronia semicostella A (BOLD:AAF4748) n=5: AUT, FIN, NOR
99
Sophronia semicostella B (BOLD:AAF4747) n=14: AUT, ESP, FRA, GER, ITA
99
Sophronia illustrella (BOLD:ACZ2459) n=1: AUT
98
Sophronia consanguinella (BOLD:ADL7320) n=1: CZE
Sophronia gelidella (BOLD:AAF4739) n=3: FIN
100
0,020

NJ tree 51

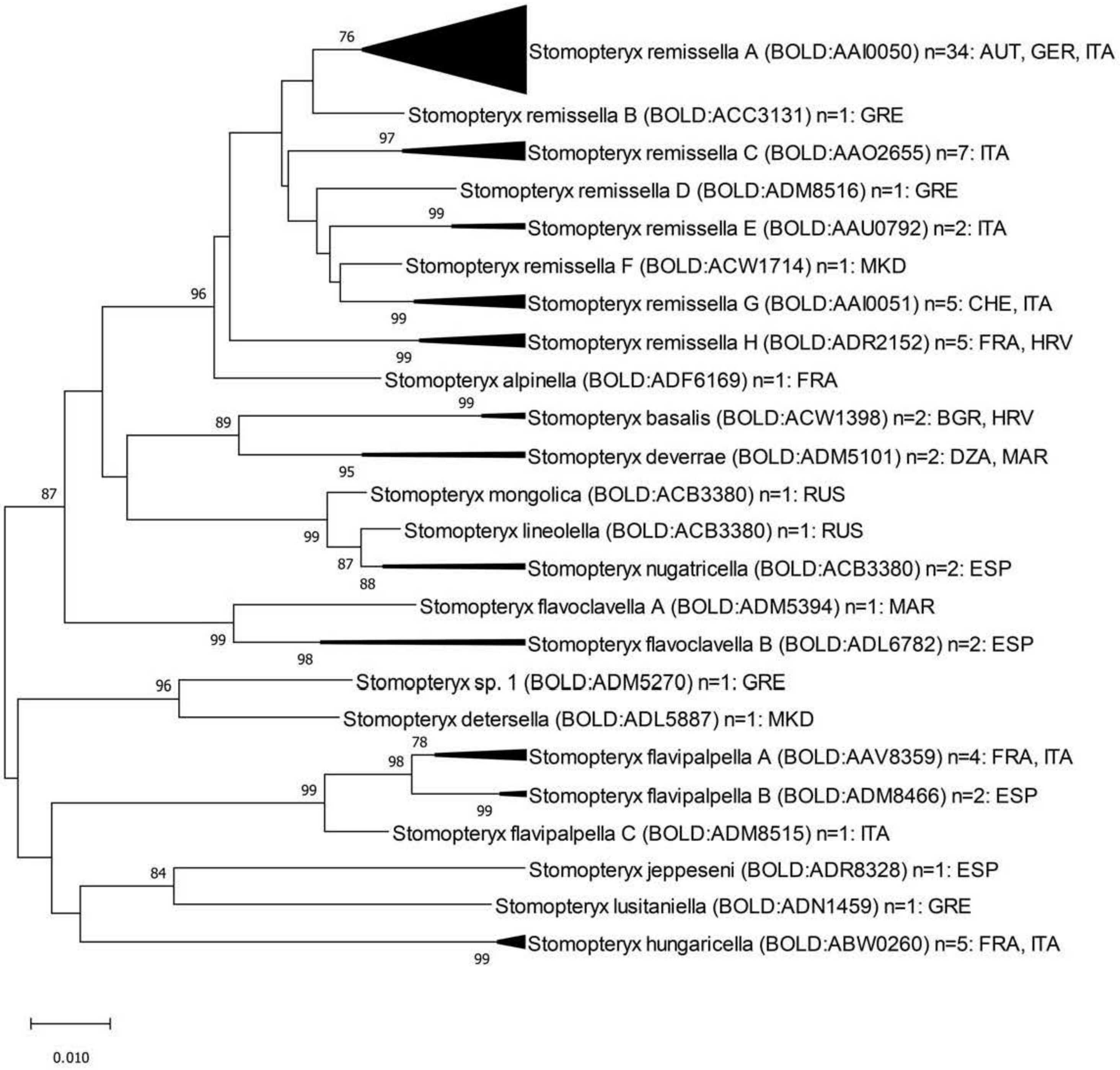
Stomopteryx remissella A (BOLD:AAI0050) n=34: AUT, GER, ITA
Stomopteryx remissella B (BOLD:ACC3131) n=1: GRE
Stomopteryx remissella C (BOLD:AAO2655) n=7: ITA
Stomopteryx remissella D (BOLD:ADM8516) n=1: GRE
Stomopteryx remissella E (BOLD:AAU0792) n=2: ITA
Stomopteryx remissella F (BOLD:ACW1714) n=1: MKD
Stomopteryx remissella G (BOLD:AAI0051) n=5: CHE, ITA
Stomopteryx remissella H (BOLD:ADR2152) n=5: FRA, HRV
Stomopteryx alpinella (BOLD:ADF6169) n=1: FRA
Stomopteryx basalis (BOLD:ACW1398) n=2: BGR, HRV
Stomopteryx deverrae (BOLD:ADM5101) n=2: DZA, MAR
Stomopteryx mongolica (BOLD:ACB3380) n=1: RUS
Stomopteryx lineolella (BOLD:ACB3380) n=1: RUS
Stomopteryx nugatricella (BOLD:ACB3380) n=2: ESP
Stomopteryx flavoclavella A (BOLD:ADM5394) n=1: MAR
Stomopteryx flavoclavella B (BOLD:ADL6782) n=2: ESP
Stomopteryx sp. 1 (BOLD:ADM5270) n=1: GRE
Stomopteryx detersella (BOLD:ADL5887) n=1: MKD
Stomopteryx flavipalpella A (BOLD:AAV8359) n=4: FRA, ITA
Stomopteryx flavipalpella B (BOLD:ADM8466) n=2: ESP
Stomopteryx flavipalpella C (BOLD:ADM8515) n=1: ITA
Stomopteryx jeppeseni (BOLD:ADR8328) n=1: ESP
Stomopteryx lusitaniella (BOLD:ADN1459) n=1: GRE
Stomopteryx hungaricella (BOLD:ABW0260) n=5: FRA, ITA
76
97
99
96
99
99
99
89
95
87
99
87
88
99
98
96
78
98
99
99
84
99
0.010

NJ tree 52

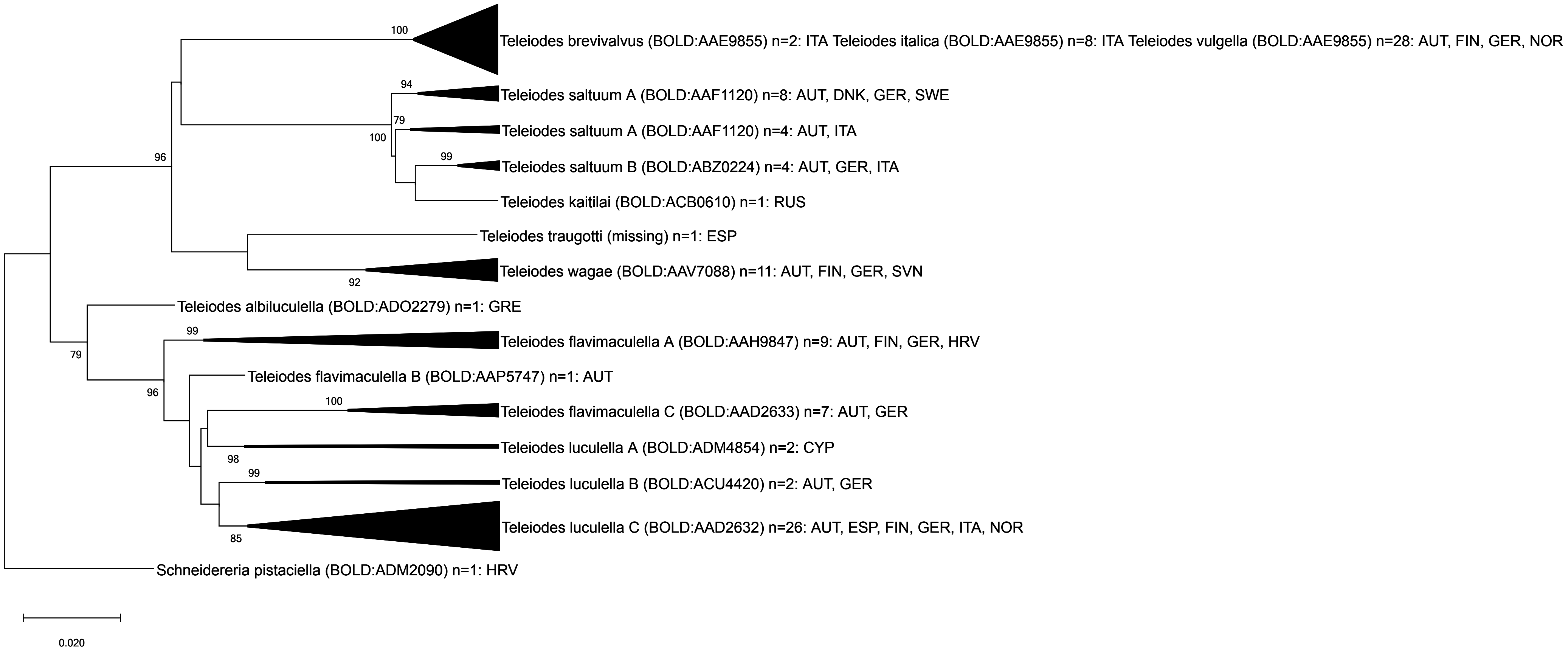

100
Teleiodes brevivalvus (BOLD:AAE9855) n=2: ITA Teleiodes italica (BOLD:AAE9855) n=8: ITA Teleiodes vulgella (BOLD:AAE9855) n=28: AUT, FIN, GER, NOR
94
Teleiodes saltuum A (BOLD:AAF1120) n=8: AUT, DNK, GER, SWE
79
100
Teleiodes saltuum A (BOLD:AAF1120) n=4: AUT, ITA
96
99
Teleiodes saltuum B (BOLD:ABZ0224) n=4: AUT, GER, ITA
Teleiodes kaitilai (BOLD:ACB0610) n=1: RUS
Teleiodes traugotti (missing) n=1: ESP
Teleiodes wagae (BOLD:AAV7088) n=11: AUT, FIN, GER, SVN
92
Teleiodes albiluculella (BOLD:ADO2279) n=1: GRE
99
Teleiodes flavimaculella A (BOLD:AAH9847) n=9: AUT, FIN, GER, HRV
79
Teleiodes flavimaculella B (BOLD:AAP5747) n=1: AUT
96
100
Teleiodes flavimaculella C (BOLD:AAD2633) n=7: AUT, GER
Teleiodes luculella A (BOLD:ADM4854) n=2: CYP
98
99
Teleiodes luculella B (BOLD:ACU4420) n=2: AUT, GER
Teleiodes luculella C (BOLD:AAD2632) n=26: AUT, ESP, FIN, GER, ITA, NOR
85
Schneidereria pistaciella (BOLD:ADM2090) n=1: HRV
0.020

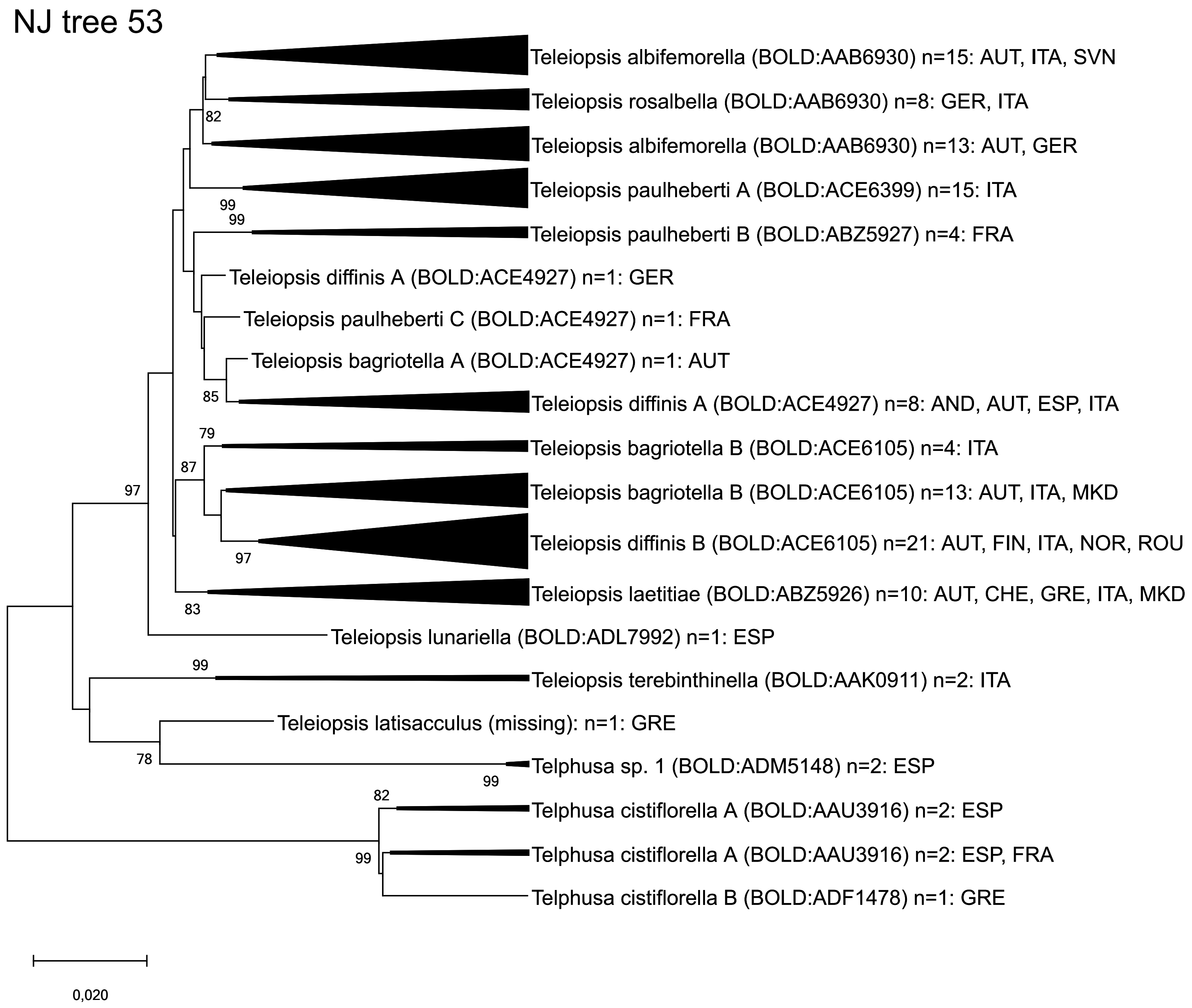
NJ tree 53
Teleiopsis albifemorella (BOLD:AAB6930) n=15: AUT, ITA, SVN
Teleiopsis rosalbella (BOLD:AAB6930) n=8: GER, ITA
Teleiopsis albifemorella (BOLD:AAB6930) n=13: AUT, GER
Teleiopsis paulheberti A (BOLD:ACE6399) n=15: ITA
Teleiopsis paulheberti B (BOLD:ABZ5927) n=4: FRA
Teleiopsis diffinis A (BOLD:ACE4927) n=1: GER
Teleiopsis paulheberti C (BOLD:ACE4927) n=1: FRA
Teleiopsis bagriotella A (BOLD:ACE4927) n=1: AUT
Teleiopsis diffinis A (BOLD:ACE4927) n=8: AND, AUT, ESP, ITA
Teleiopsis bagriotella B (BOLD:ACE6105) n=4: ITA
Teleiopsis bagriotella B (BOLD:ACE6105) n=13: AUT, ITA, MKD
Teleiopsis diffinis B (BOLD:ACE6105) n=21: AUT, FIN, ITA, NOR, ROU
Teleiopsis laetitiae (BOLD:ABZ5926) n=10: AUT, CHE, GRE, ITA, MKD
Teleiopsis lunariella (BOLD:ADL7992) n=1: ESP
Teleiopsis terebinthinella (BOLD:AAK0911) n=2: ITA
Teleiopsis latisacculus (missing): n=1: GRE
Telphusa sp. 1 (BOLD:ADM5148) n=2: ESP
Telphusa cistiflorella A (BOLD:AAU3916) n=2: ESP
Telphusa cistiflorella A (BOLD:AAU3916) n=2: ESP, FRA
Telphusa cistiflorella B (BOLD:ADF1478) n=1: GRE
82
99
99
85
79
87
97
97
83
99
78
99
82
99
0,020
